# Isotope-labeled growth histories reveal persistent chirality in individual carbon nanotubes despite catalyst evolution

**DOI:** 10.1038/s41467-026-77101-2

**Published:** 2026-09-01

**Authors:** Keigo Otsuka, Ryuji Fujiwara, Shigeo Maruyama

**Affiliations:** 1 Department of Mechanical Engineering, The University of Tokyo, Tokyo, Japan; 2 State Key Laboratory of Fluid Power and Mechatronic Systems, School of Mechanical Engineering, Zhejiang University, Hangzhou, China; 3 Institute of Materials Innovation, Nagoya University, Nagoya, Japan

**Keywords:** Carbon nanotubes and fullerenes, Synthesis and processing

## Abstract

In catalytic growth of carbon nanotubes (CNTs), the stability of catalyst structures is critical for achieving structural uniformity and scalability. Continuous synthesis configurations often expose catalysts to spatially varying temperatures and gas compositions along the reactor. The structural integrity along CNTs growing under such dynamic environments remains largely unexplored, due to the inaccessibility of transient catalyst evolution. Here, we use isotope labeling to indirectly read out, from the growth history recorded within each CNT, how its catalyst evolves in a substrate-supported system. The growth rates exhibit hysteresis, varying up to twofold at the same temperature, indicating irreversible catalyst coarsening; nevertheless, the same nanotubes retain atomically identical lattice structures over hundreds of micrometers. This structural robustness, corroborated by molecular dynamics simulations under evolving catalyst conditions, calls for caution against the interpretation of catalyst–CNT structural relationships from static ex situ snapshots. The contrast between structural memory and adaptive growth kinetics further provides a mechanistic design principle of multiple-stage strategies, toward overcoming the conventional quality-quantity trade-off.

## Abstract (web summary)

The carbon nanotube structure is often assumed to be dictated by the size of the catalyst nanoparticle. Here, the authors show that each nanotube retains its original structure over hundreds of micrometres, even when the catalyst is coarsened and its growth rate changes.

## Introduction

Dry synthesis of nanomaterials can be broadly classified into substrate-supported processes (e.g., semiconductor epitaxy)^1^ or gas-phase processes (e.g., flame aerosol synthesis)^2^. Chemical vapor deposition (CVD) growth of carbon nanotubes (CNTs) has been investigated using both approaches. Supported growth enables highly ordered architectures through synergistic interactions among the substrate, catalyst, and neighboring nanotubes^3–5^. In contrast, gas-phase growth offers greater scalability^6,7^ and compatibility with dispersion and liquid-phase separation processes^8^. Consequently, single-walled CNTs (SWCNTs) are commercially produced primarily via floating-catalyst CVD (FCCVD), such as HiPco and eDIPS^9,10^, providing source materials for semiconductor applications, including transistors^11^ and single-photon emitters^12^.

**Fig. 1:**
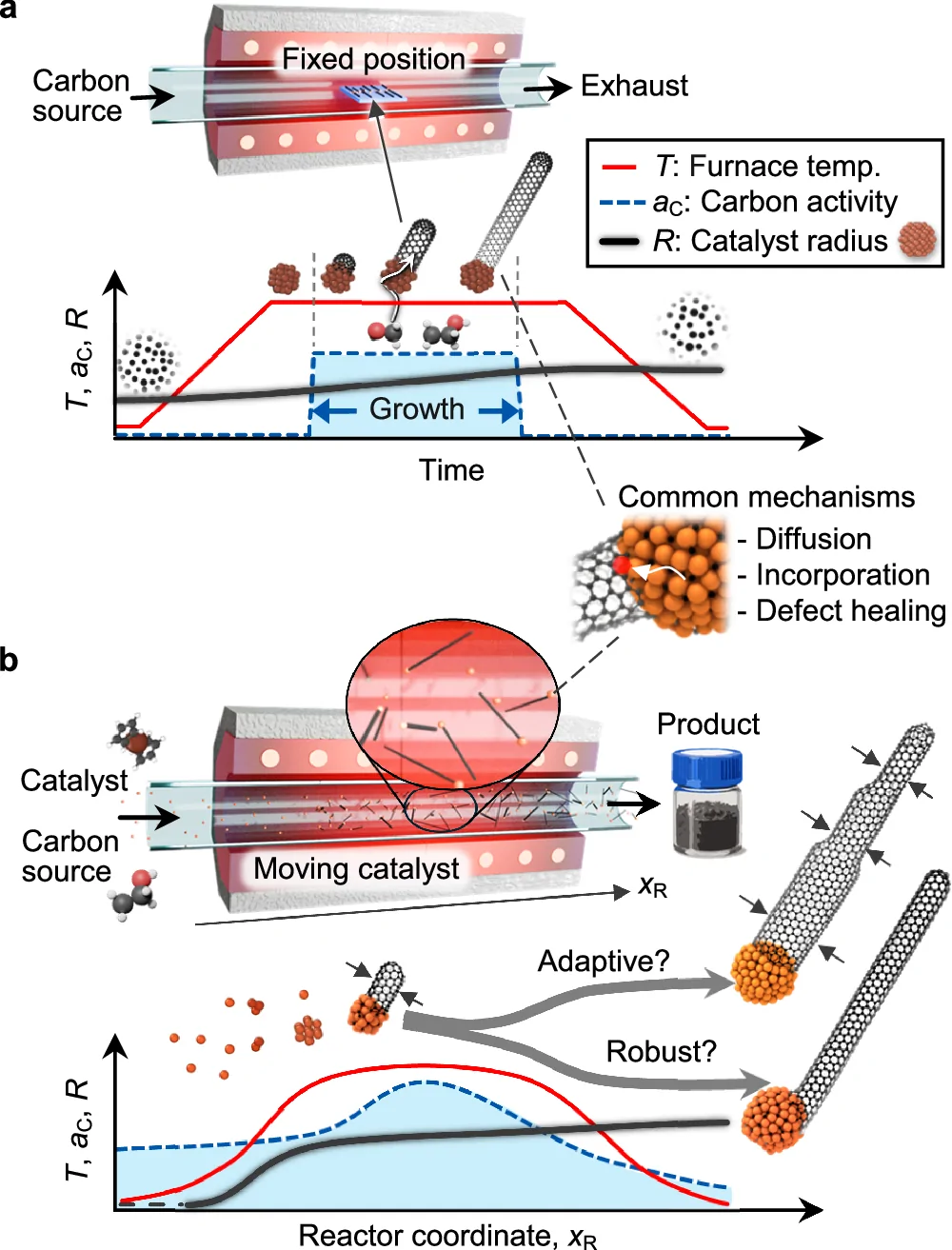
Schematic comparison of catalyst configurations for CNT synthesis. **a** Substrate-supported catalyst system for CNT growth. Growth typically proceeds at a constant temperature *T* (red), while carbon precursors are supplied for a defined duration to increase the gas-phase carbon activity *a*_C_ (blue). Meanwhile, average catalyst radius *R* (black) typically increases with time. **b** Floating-catalyst system, in which catalyst and carbon precursors are continuously introduced in the gas phase, producing spatially varying *T* and *a*_C_ as catalyst particles undergo coarsening along the reactor coordinate *x*_R_. The two systems are expected to share similar tube–catalyst interfacial dynamics.

A fundamental distinction between the two CNT growth modes lies in catalyst nanoparticle evolution. In supported systems, catalyst particles primarily grow via Ostwald ripening mediated by surface diffusion of atoms^13^, with possible subsurface diffusion of atoms into supports^14^. In contrast, in floating-catalyst reactors, catalysts usually nucleate from volatile metal precursors and enlarge predominantly through particle coalescence at relatively low temperatures, while evaporation-driven redistribution also plays a crucial role at higher temperatures^15,16^. Growth promoters commonly employed in these systems, such as sulfur^17^ and CO_2_^18^, may also influence the catalyst evolution processes. The fixed catalyst positions in supported systems enable precise temporal control of growth conditions: precursors are supplied for a defined duration once the temperature *T* reaches the set point (Fig. 1a). This controlled environment permits simplified descriptions of elongation kinetics^19,20^. Conversely, FCCVD processes involve dynamic fluctuations, as catalyst particles continuously coarsen under spatially varying temperature and feedstock composition along the reactor coordinate (Fig. 1b)^16^. Such evolving conditions complicate precise structural control in gas-phase synthesis.

Thermal decomposition and depletion of feedstock species in CVD reactors generate spatial variations in gas-phase composition and carbon activity *a*_C_^21^, leading to position-dependent growth behavior^22,23^. An ex situ transmission electron microscopy (TEM) study has suggested that increasing carbon chemical potential reduces nanotube diameter^24^ via growth mode changes^25^. Diameter modulation within a single SWCNT has also been reported upon temperature modulation^26^. Because floating catalysts experience evolving chemical potential and temperature, the resulting SWCNT diameter is expected to vary accordingly. Molecular dynamics (MD) simulations further show that nanotube diameters can dynamically adjust to catalyst size^27^, reinforcing this expectation.

Since any change in nanotube diameter requires topological defect incorporation, consistency with the literature on high-crystallinity SWCNT growth warrants examination. Defect formation is in practice governed by kinetics rather than equilibrium thermodynamics^28^. Under steady growth conditions, chirality preservation along SWCNTs over hundreds of micrometers has been well reported^29–31^, further supported by recent MD simulations through the modeling of defect-healing kinetics^32,33^. These supports for robustness, however, do not rule out structural adaptation under dynamically evolving conditions: these models assume planar interfaces^28^ or fixed interfaces^32^, leaving the effects of tube–catalyst mutual curvature and contact geometry unresolved.

Whether the structure of an individual SWCNT is adaptive or robust as temperature and catalyst size vary during elongation, therefore, calls for direct experimental resolution. Ensemble measurements inherently lack information on structural continuity along individual nanotubes, while ex situ analyses of isolated SWCNTs cannot directly link environmental perturbations to specific growth locations. Although in situ TEM can probe catalyst nanoparticles during active nanotube growth^34–38^, its intrinsic constraints, including the general requirement for low pressure/temperature and electron-beam-induced damage, render it incompatible with the growth of well-crystallized SWCNTs under practical conditions.

Here, we combine digital isotope labeling^39^ with controlled thermal cycling to investigate chirality preservation in individual SWCNTs under dynamic environments, with the growth rate history serving as a probe of catalyst-size evolution over hundreds of micrometers. The experimental observations are integrated with kinetic Monte Carlo simulations of stochastic catalyst coarsening and MD simulations of SWCNT growth, providing complementary perspectives across length and time scales. Our results reveal robust structural memory alongside adaptive kinetics, establishing a rigorous baseline for mechanistic discussion of chirality determination and a foundation for practical strategies that integrate structural control with scalable growth.

## Results and Discussion

### Tracking individual growth under temperature modulation

**Fig. 2:**
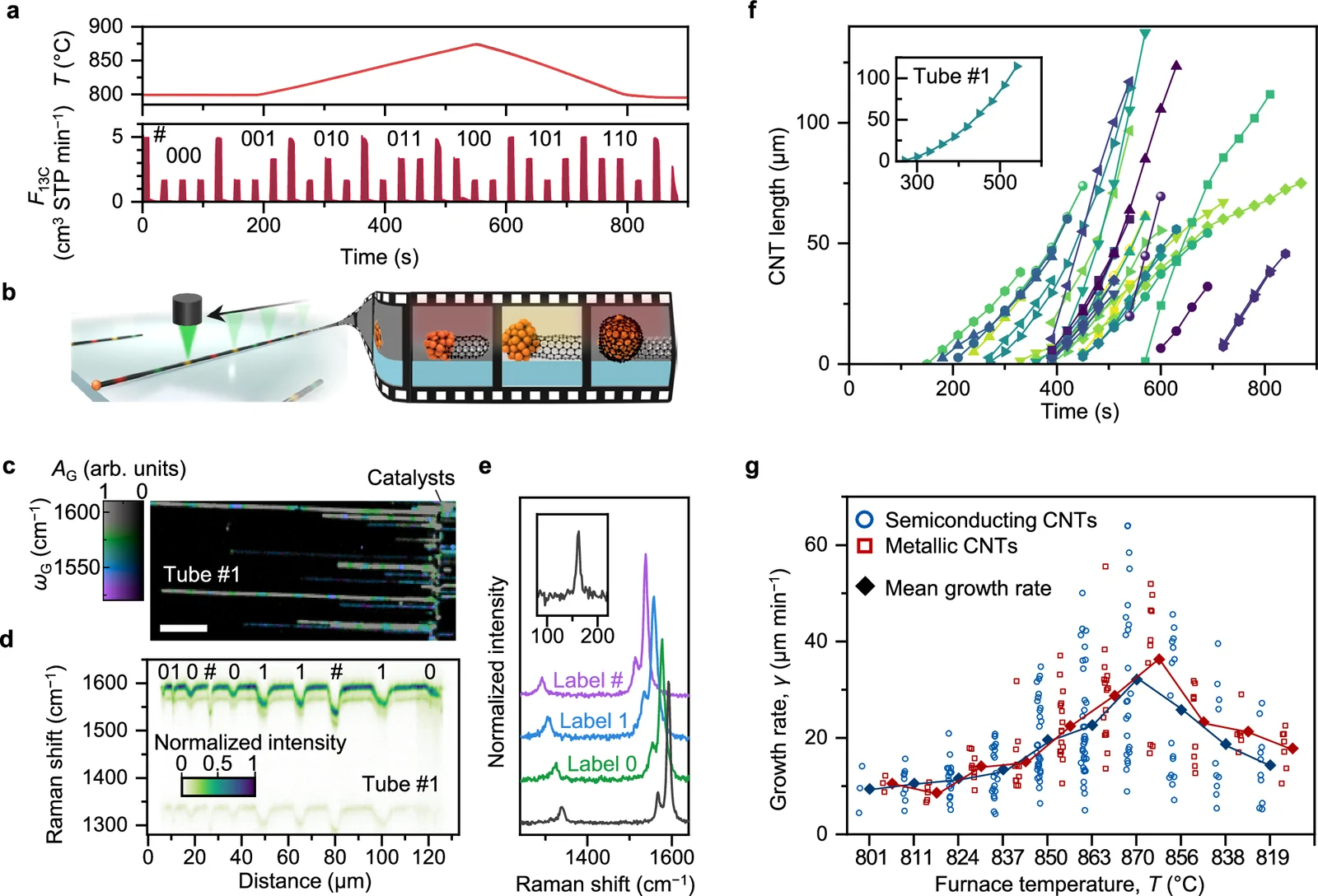
Individual growth profiles of substrate-supported CNTs under temperature modulation. **a** Time profiles of furnace temperature (top) and flow rate *F*_13C_ of ^13^C-enriched ethanol pulses (bottom). The total ethanol flow rate (^12^C + ^13^C) was maintained at 5 cm^3^ STP min^−1^. **b** Conceptual illustration of isotope-encoded growth-history readout from individual CNTs. **c** Raman spectral map, where brightness and color represent the integrated G-mode area *A*_G_ and peak frequency *ω*_G_, respectively. Scale bar, 20 μm. **d** Raman spectral profile acquired along the horizontal axis of tube #1 indicated in **c**. **e** Raman spectra showing the G-mode of the ^12^C-grown segment (gray) and three isotope-labeled segments (0, 1, and # in green, blue, and purple, respectively). The inset displays the radial breathing mode spectrum of the ^12^C-grown segment. **f** Length evolution of representative CNTs; the growth history of tube #1 is shown in the inset. **g** Growth-rate distributions (open symbols) for semiconducting (blue) and metallic (red) CNTs, with corresponding mean values (filled diamonds), plotted as a function of temperature *T*. Source data are provided as a Source Data file.

Among various key distinctions between supported- and floating-catalyst systems, we focus on a few variables specific to FCCVD—temperature and carbon activity—and impose them in a controlled manner on substrate-supported CNTs. We should note that growth rates reported for FCCVD and supported systems fall on a common scaling (Supplementary Fig. 1), indicating a unified kinetic framework among different configurations. Here, we focus on programmed temperature variation during CNT elongation, as the effects of carbon activity fluctuations have been partially examined through modulation of carbon feedstocks and etching agents^40,41^. Aligned CNTs were synthesized on quartz substrates (Supplementary Fig. 2). The furnace temperature was programmed to follow a heating–cooling sequence between 800 and 873 °C (Fig. 2a, top). Three-bit digital isotope labeling^39^ was applied at 30 s intervals throughout growth (Fig. 2a, bottom) to track time-resolved elongation of individual nanotubes (Fig. 2b), which also serves as an indirect readout of the catalyst state during growth.

Fig. 2c presents a Raman map of the G-mode intensity for CNT arrays transferred onto a Si/SiO_2_ substrate. The color scale represents the G-mode frequency *ω*_G_, and the brightness corresponds to the peak area *A*_G_. Raman spectra acquired along a representative nanotube (tube #1) are shown in Fig. 2d, displaying spatially correlated G- and D-mode features. Distinct frequency downshifts mark the positions of the inserted isotope labels. Representative spectra from each labeled segment and from unlabeled ^12^C-derived regions are shown in Fig. 2e. The nanotube length at each labeling time can be reconstructed from the spacing between isotope markers (Fig. 2f). Pronounced variability is observed in incubation time, growth termination, and elongation rate. For tube #1, continuous growth occurs from ≈300 to 550 s, with a rate that varies in response to the programmed temperature profile (Fig. 2f, inset).

In the present sample configurations, metallic and semiconducting CNTs (m- and s-CNTs) can be clearly distinguished based on their G-mode features over their entire lengths, although Raman features are influenced by local fluctuations, including doping and strain^42^. We briefly compared the growth-rate distributions of each type within individual temperature segments. Across the examined temperature range, no significant metallicity dependence was observed (Fig. 2g).

### Hysteretic behavior in growth termination and kinetics

**Fig. 3:**
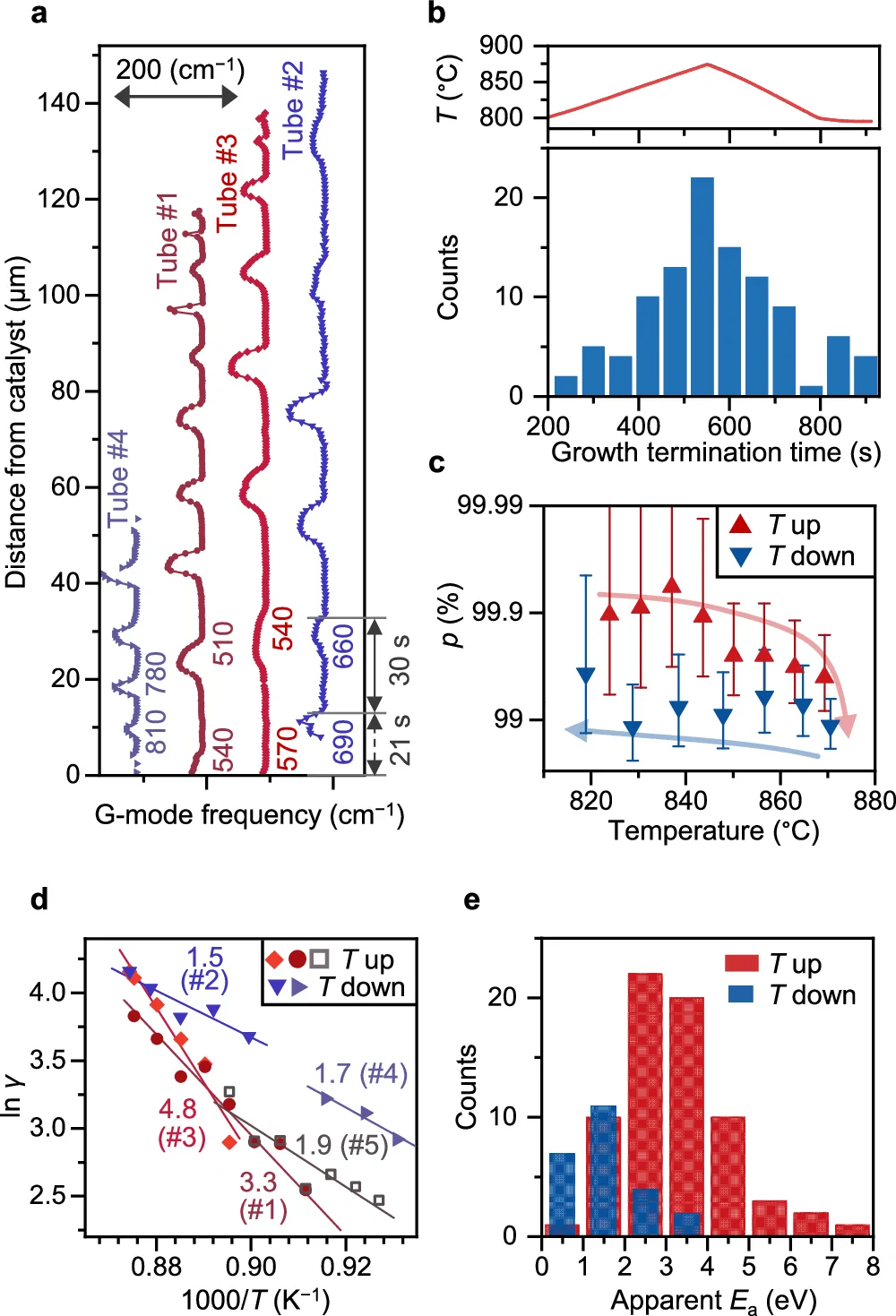
Growth termination and kinetic hysteresis under thermal modulation. **a** Axial profiles of the G-mode frequency *ω*_G_ in the Raman spectra of four CNTs. Numbers to the right of each trace denote the onset time of the corresponding isotope labels. **b** Histogram of growth-termination times for all analyzed CNTs. The temporal profile of furnace temperature *T* is shown above for reference. **c** Survival probability *p* over a 1 s interval for growing CNTs plotted as a function of temperature. Red and blue symbols represent point estimates of *p* calculated from the pooled counts of actively growing CNTs and growth-termination events within each temperature bin during temperature ramp-up and ramp-down, respectively. Error bars represent 95% confidence intervals assuming Poisson statistics. The number of independent CNTs counted at each data point is provided in the Source Data file. Arrows indicate the direction of temperature modulation. **d** Arrhenius plots derived from growth rates of five representative CNTs. Values in parentheses identify individual CNTs; values outside parentheses denote the corresponding activation energies (*E*_a_, in eV). **e** Distributions of apparent *E*_a_ extracted separately for temperature ramp-up (*T* up, red) and ramp-down (*T* down, blue) periods. Source data are provided as a Source Data file.

Growth lifetime and elongation rate *γ* jointly determine the final CNT length. To quantify the temperature dependence of growth termination, we approximated the termination time for each CNT by linearly extrapolating the growth rate defined between the last two isotope labels to the catalyst positions (Fig. 3a). The resulting termination-time histogram peaks at ≈540 s (Fig. 3b), corresponding to the maximum furnace temperature. This observation indicates that elevated temperature shortens the growth lifetime, revealing a tradeoff between elongation rate and lifetime. No discernible difference in termination timing was observed between metallic and semiconducting CNTs. Accordingly, the s-CNT:m-CNT yield ratio remained constant across the examined temperature range (Supplementary Fig. 4).

To further quantify the temperature dependence, we define the survival probability *p* over a unit time interval *τ* (= 1 s) as

1 $$p\left(t,T\right)=\exp (-q\tau )\cong 1-q\left(t,T\right)\tau,$$

where $q(t,T)\equiv D(t)/(N\left(t\right)\Delta t)$ is the instantaneous growth-termination rate, *N*(*t*) is the number of actively growing nanotubes, and *D*(*t*) is the number of termination events within the sampling interval Δ*t* (see Supplementary Note 5). Plotting *p* as a function of furnace temperature reveals an increased termination frequency at elevated temperatures (Fig. 3c). The survival probability *p* exhibits clear hysteresis: for the same temperature, *p* is systematically lower during the ramp-down stage than during ramp-up. Consistently, most CNTs had already terminated growth before the carbon feed was stopped (Fig. 2f), indicating that accelerated elongation is accompanied by shortened growth lifetime.

The temperature-dependent growth rate *γ* provides further insight into the rate-limiting steps. Arrhenius plots (Fig. 3d) enable extraction of the apparent activation energy *E*_a_ from the slope. Two key features emerge: (i) *E*_a_ varies substantially among individual nanotubes, and (ii) it differs systematically between temperature ramp-up and ramp-down stages. Fig. 3e summarizes these trends for CNTs containing at least three segments between isotope labels, thereby minimizing fitting uncertainty. During temperature ramp-down, *E*_a_ predominantly falls within 1–2 eV, consistent with previous reports^43,44^. In contrast, *E*_a_ is significantly larger during the heating stage and exhibits pronounced scatter (Supplementary Fig. 6). These values substantially exceed those typically reported for catalytic CVD growth, including our prior isotope-labeling study conducted at ≈800 °C^39^.

The unusually large *E*_a_ values during temperature ramp-up warrant attention. In a simple serial-rate (series-resistance) framework, the apparent activation energy of the rate-limiting step would be expected to decrease as temperature increases. The opposite trend observed here suggests two possible scenarios: (i) growth proceeds via parallel kinetic pathways whose relative contributions vary with temperature, for example, a shift in the dominant carbon source from ethanol to its pyrolysis products (e.g., acetylene). (ii) The growth rate is constrained by an irreversible, non-thermal factor that evolves with time, such as progressive catalyst coarsening at elevated temperatures. In such a case, projecting the growth rate evolution onto an Arrhenius plot requires caution, and we therefore refer to the extracted *E*_a_ values as apparent activation energies.

### Explanation of observed hysteresis

**Fig. 4:**
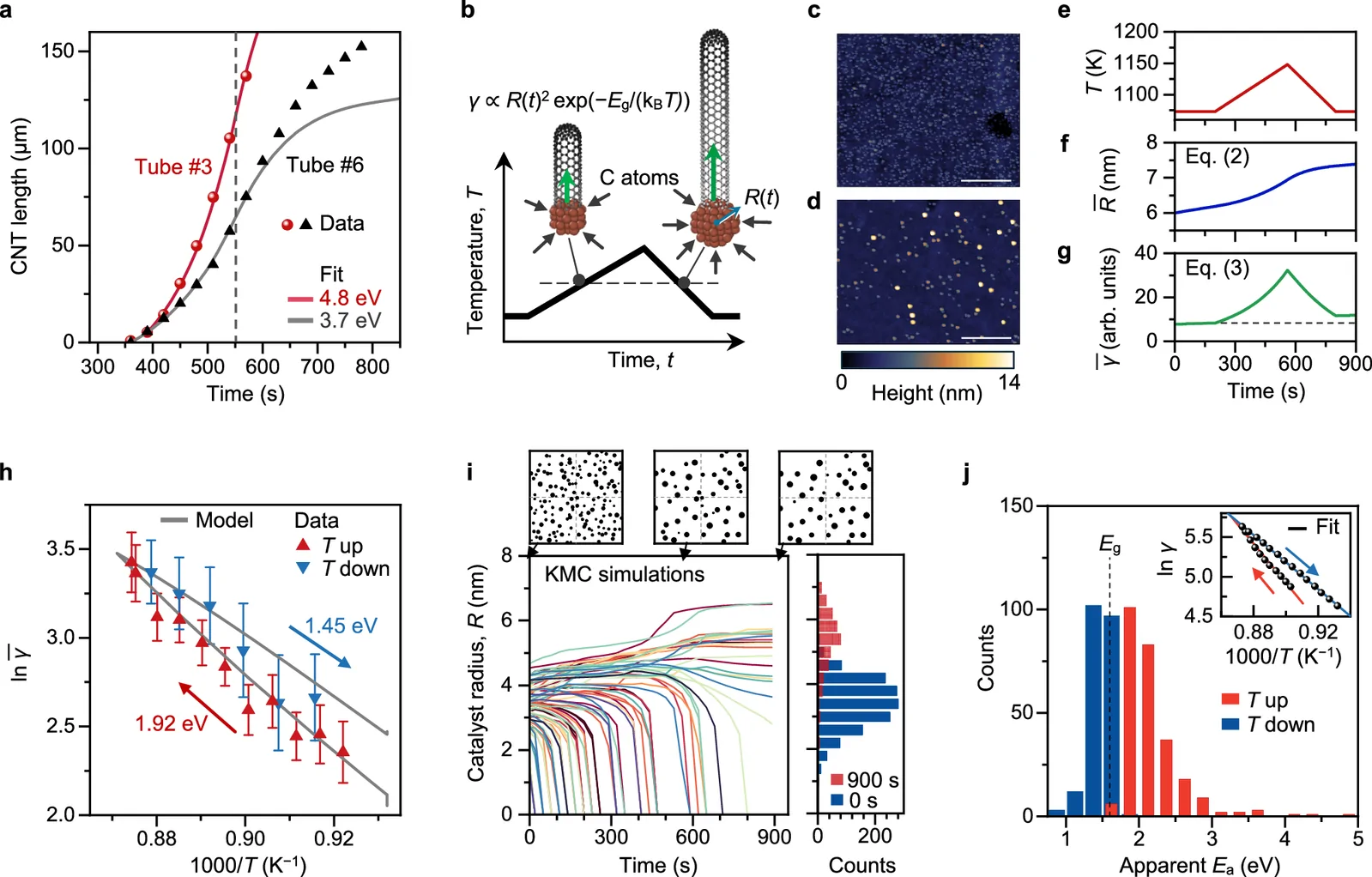
Ensemble and single-tube evidence for catalyst coarsening during CNT growth. **a** Time evolution of CNT length for representative nanotubes that terminated during the temperature ramp-up stage (tube #3, red) and those that continued growing into the ramp-down stage (tube #6, black). A vertical dashed line represents the highest temperature point. Solid curves show predictions based on a single activation energy *E*_a_ fitted to early-stage growth rates. **b** Schematic illustration of deviations between experimental data and single-*E*_a_ fits when the growth rate scales with catalyst surface area. **c**, **d** Atomic force microscopy (AFM) images of catalyst nanoparticles after CNT growth at 800 °C (**c**) and 850 °C (**d**) for 20 min. Scale bars, 500 nm. The sample shown in **c** was prepared once. **e–g** Programmed temperature profile (**e**), average catalyst radius $\bar{R}$ (**f**), and corresponding growth rate $\bar{\gamma }$ calculated using Eq. (3) (**g**). **h** Arrhenius plots comparing calculated $\bar{\gamma }$ with experimental mean values measured for individual CNTs. Error bars denote the relative standard errors of the mean. The number of independent CNTs averaged at each data point is provided in the Source Data file. **i** Evolution of individual catalyst sizes obtained from kinetic Monte Carlo (KMC) simulations under a temperature profile similar to that in (**e**). Top: snapshots of surviving par*t*icles at *t* = 0, 570, and 900 s (left to right) in 2 × 2 unit cells. Right: Distribution of particle size at *t* = 0 s (blue) and 900 s(red). **j** Distributions of apparent activation energies *E*_a_ extracted from ramp-up (red) and ramp-down (blue) stages in KMC simulations. Inset: representative Arrhenius plot derived from a simulated growth trajectory. Source data are provided as a Source Data file.

To distinguish between the two proposed mechanisms underlying the hysteresis, we examined CNTs that remained active during both the temperature ramp-up and ramp-down stages. This approach enables direct comparison of the growth behavior of the same nanotube at identical temperatures but at different stages of catalyst evolution. Fig. 4a presents the time-dependent length evolution for two representative CNTs. For a nanotube that terminated during ramp-up (tube #3; *E*_a_ = 4.8 eV), the growth profile is well described by a single-activation-energy model. In contrast, the same model fails to reproduce the behavior of a nanotube that continued growing beyond the peak temperature into the ramp-down stage (tube #6; *E*_a_ = 3.7 eV), significantly underestimating the observed elongation.

The observed deviation cannot be reconciled within simple series or parallel kinetic analogies, in which the growth rate depends solely on temperature-dependent reaction barriers. At ≈837 °C, the growth rate during ramp-down is enhanced by a factor of 1.9 relative to ramp-up at the same temperature, indicating an additional irreversible change induced by prior high-temperature exposure. This behavior can be rationalized if the elongation rate scales with catalyst surface area, so that catalyst size evolution (Fig. 4b) produces different rates at the same temperature. Such scaling nearly holds in the weak-etching regime at the low ethanol partial pressure ( ≈135 Pa) used here^40,45^ (see Supplementary Fig. 7). In this scenario, CNTs grown at the same temperature exhibit different growth rates depending on the catalyst’s thermal history. We note that a transition from tangential to perpendicular growth modes^24^ alone cannot account for the growth rate change, because such a transition presupposes a corresponding change in catalyst size as long as tube chirality remains unchanged.

To assess whether significant catalyst coarsening occurred, we analyzed the average behavior of Fe nanoparticles, irrespective of whether they nucleated CNTs. Catalyst particles were characterized by atomic force microscopy (AFM) following CNT growth at 800 and 850 °C for 20 min, after removal of CNTs by oxygen plasma treatment. At 800 °C, the catalyst-size distribution remained largely unchanged over time (Fig. 4c). In contrast, growth at 850 °C resulted in the emergence of larger particles (10–15 nm) via Ostwald ripening, yielding a bimodal size distribution^46^, while the main population remained near 4–5 nm (Fig. 4d). These observations indicate substantial catalyst evolution at elevated temperatures. Consistently, AFM measurements on as-grown samples showed catalyst heights larger than the corresponding CNT diameters (Supplementary Fig. 9).

To estimate the impact of catalyst coarsening on CNT growth rates, we adopted a Lifshitz–Slyozov–Wagner (LSW)-type description of Ostwald ripening, extended to sparsely distributed, surface-supported nanoparticles. In this framework, the average particle radius $\bar{R}(t)$ for diffusion-limited coarsening empirically follows^47,48^

2 $${\bar{R}}^{4}\left(t\right)-{\bar{R}}^{4}\left(0\right)={Kt},$$

where $\bar{R}(0)$ is the initial radius. The ripening rate coefficient is expressed as $K(T)={K}_{0}\exp (-{E}_{{{{\rm{r}}}}}/({{{{\rm{k}}}}}_{{{{\rm{B}}}}}T))$, with *E*_r_ denoting the effective activation barrier for ripening—primarily governed by surface diffusion barriers, cohesive energies, and adsorption energies^48^. The prefactor *K*_0_ was selected based on prior measurements indicating a ≈4% min^−1^ increase in growth rate at 800 °C^49^. We employed the differential form of the Ostwald ripening model (Eq. 2), ${{{\rm{d}}}}\bar{R}/{{{\rm{d}}}}t=K(T)/4{\bar{R}}^{3}$ to obtain the evolution of the average catalyst radius $\bar{R}$ under temperature modulation. Based on this relation, we constructed a deterministic ensemble model to quantify the impact of catalyst coarsening on CNT growth kinetics. The model incorporates the programmed temperature profile (Fig. 4e), the evolution of $\bar{R}$ derived from the LSW-type scaling (Fig. 4f), and the average CNT growth rate $\bar{\gamma }$ (Fig. 4g). The latter is expressed as the product of a surface-area term $\bar{A}\propto {\bar{R}}^{2}$ and a thermally activated kinetic factor with effective activation energy *E*_g_ = 1.6 eV. Accordingly,

3 $$\bar{\gamma }\left(T,\bar{R}\right)={\varGamma }_{0}{\bar{R}(t)}^{2}\exp \left(-\frac{{E}_{{{{\rm{g}}}}}}{{{{{\rm{k}}}}}_{{{{\rm{B}}}}}T}\right),$$

where the proportionality constant *Γ*_0_ is chosen such that $\bar{\gamma }$ matches the experimentally observed magnitude. When recast as Arrhenius plots, the predicted $\bar{\gamma }$ values naturally exhibit hysteresis between ramp-up and ramp-down branches (Fig. 4h). This ensemble model reproduces the average growth rate of s-CNTs (Fig. 2g) when *E*_r_ = 2.6 eV is used, which reasonably agrees with a first-principles kinetic study for Ni on TiO_2_^50^. These simulations thus indicate that catalyst coarsening can account for the large apparent activation energies and the observed hysteresis.

### Stochastic catalyst coarsening explains inter-tube scatter

The deterministic ensemble model adequately describes average behavior but does not capture the unusually large apparent *E*_a_ observed for individual nanotubes (Fig. 4a) or the pronounced inter-tube variability (Fig. 3d). In practice, catalyst populations evolve heterogeneously: a minority of particles grow, whereas others shrink or disappear. To account for this stochastic evolution, we employed kinetic Monte Carlo (KMC) simulations. Specifically, we implemented the three-dimensional surface Ostwald ripening model developed by Prévot^48^, which explicitly tracks the time-dependent radii *R* of individual particles (see Supplementary Fig. 8). The simulated size evolution of randomly selected particles under the same temperature modulation is presented in Fig. 4i. As expected, most particles vanished over time, while a small fraction continued to grow, leading to a reduced total particle number and a shift in the radius distribution.

To gain further insight into stochastic CNT elongation, we simulated CNT growth from the KMC-derived nanoparticles. Since our measurements resolve only CNTs whose length and growth rate exceed detection thresholds, we selected a relatively large subset of catalysts to calculate an instantaneous growth rate *γ* described by Eq. (3) (Supplementary Note 7 for details). The same activation energy (*E*_g_ = 1.6 eV) used in the ensemble model was retained, while growth initiation time and lifetime were randomized to reflect experimental variability. Despite this minimal parameterization, the resulting KMC-derived *E*_a_ distributions (Fig. 4j) are qualitatively consistent with the experimental results (Fig. 3e), supporting that stochastic catalyst coarsening governs both the magnitude and hysteresis of the apparent activation energies. The simulated Arrhenius plots (Fig. 4j, inset) reproduce the broad distribution of apparent *E*_a_ values and the systematic enhancement during temperature ramp-up relative to ramp-down observed experimentally. The comparable hysteresis in growth lifetime (Fig. 3c) may reflect the increase in carbon chemical potential of catalysts, providing independent support for irreversible catalyst coarsening (see Supplementary Note 6 for modeling).

### Chirality preservation under dynamic growth environments

**Fig. 5:**
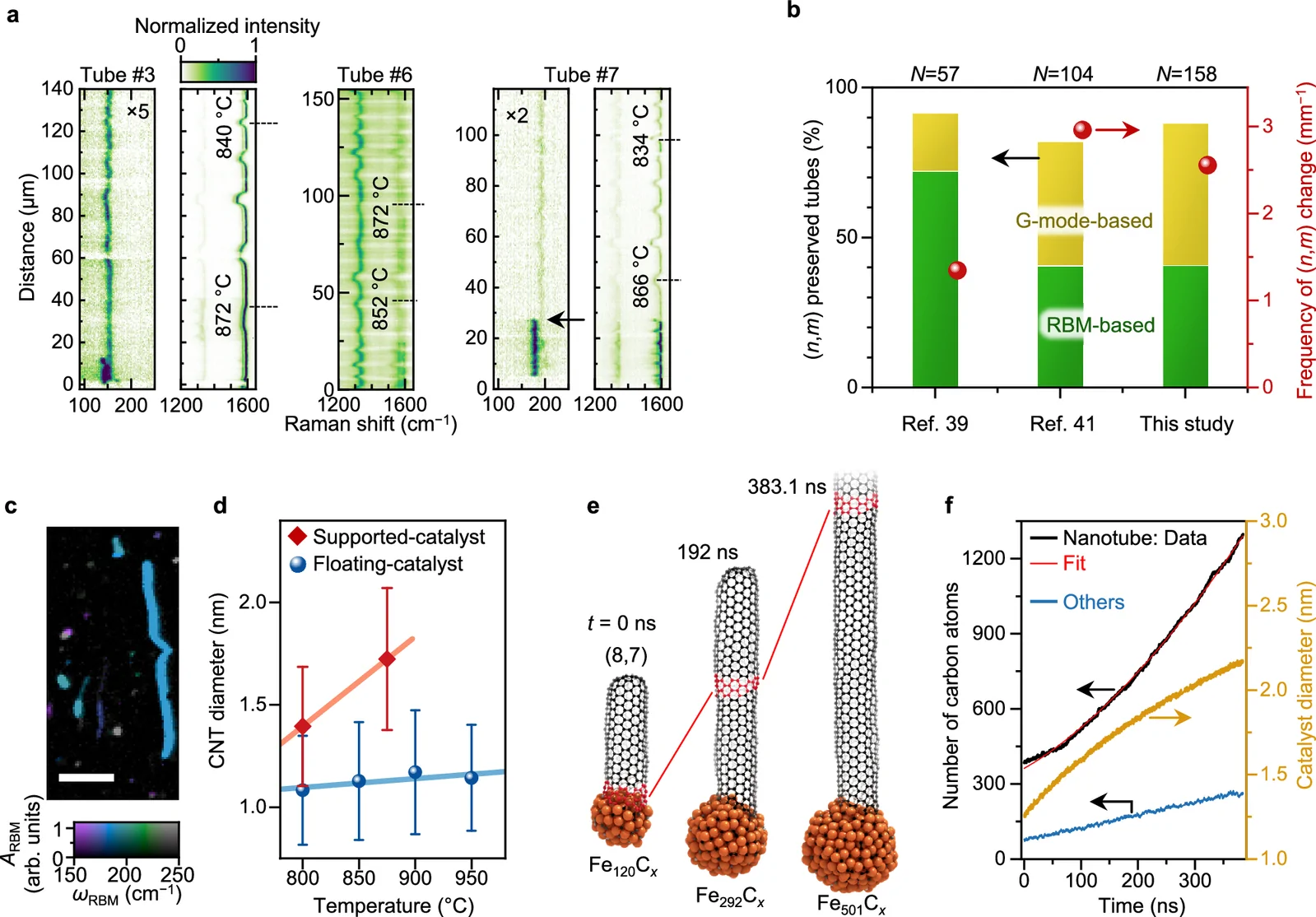
Robust chirality preservation under evolving environments. **a** Raman spectral profiles acquired along CNTs, showing two tubes that preserved chirality (left and center) and one tube exhibiting a localized chirality change (right). **b** Count-based fraction of chirality-preserved CNTs (bars), as confirmed by radial breathing mode (RBM) profiles (green) or inferred from G-mode features (yellow). Results from constant-temperature growth^39^ and a growth-interruption–etching protocol^41^ are included for comparison. The length-normalized frequencies of (*n,m*) transitions are indicated by red symbols. **c** Raman RBM mapping of floating-catalyst chemical vapor deposition (FCCVD)-derived CNTs directly deposited onto a Si/SiO_2_ substrate. Scale bar, 5 µm. **d** Temperature dependence of CNT diameter for substrate-supported growth (red) and FCCVD growth (blue). Red symbols represent the mean diameters measured for individual CNTs. The number of independent CNTs counted at each data point is provided in the Source Data file. Blue symbols represent diameters estimated from the centroid of the ensemble RBM spectra. Error bars denote the within-sample standard deviation of CNT diameter. **e** Snapshots of the growth trajectories in molecular dynamics (MD) simulation for a representative (8,7) CNT growing from an iron nanoparticle that gradually grows in size. Atoms in red represent the hexagons located at the tube edge at *t* = 0. **f** Time evolution of carbon atoms incorporated into the nanotube (black), non-CNT carbon atoms (blue), and catalyst diameter (yellow), showing increasing catalyst size and accelerated growth while the SWCNT chirality remains fixed. The red line is a fitting result, assuming that the growth rate is proportional to the catalyst surface area. Source data are provided as a Source Data file.

A final key question is whether CNTs retain their diameter and chirality during elongation from catalysts undergoing Ostwald ripening. Fig. 5a presents Raman spectral profiles acquired along three representative CNTs. For tube #3, the radial breathing mode (RBM) response and the continuity of the G- and D-mode features confirm that the chiral indices (*n*,*m*) remain unchanged along the measured length. In metallic tube #6, although RBM features are absent, the homogeneous evolution of G- and D-mode intensity, lineshape, and peak positions supports chirality preservation. In contrast, tube #7 exhibits abrupt spectral changes in both the G-mode and RBM, indicating a structural transition. Additional full-length Raman traces (eight tubes per category) are provided in Supplementary Fig. 10. Tube #3 preserves its chirality despite an ≈3.5-fold increase in growth rate *γ* over a temperature rise of only ≈30 °C, which reflects substantial catalyst coarsening. Similarly, tube #6 grows from the maximum temperature down to ≈800 °C without any detectable change in chirality.

CNTs classified as chirality-preserved based on RBM or G-mode criteria constituted the clear majority of the dataset. Among 158 analyzed CNTs, 139 tubes (88%) maintained their chiral indices (*n*,*m*) over the entire measured length. The length-normalized frequency of chirality-change events was 2.6 mm^−1^ (total inspected length, 9.39 mm; 24 (*n*,*m*) transitions), corresponding to one event every 391 µm. These results demonstrate that tube chirality and diameter are robustly preserved despite concurrent temperature modulation and catalyst-size evolution. Fig. 5b compares this robustness with our previous studies under steady growth at 800 °C^39^ and under alternating growth and H_2_O-induced etching^41^. We note that changes in Raman resonant conditions can be overlooked and may lead to overestimation of these values (Supplementary Fig. 12). However, the overall level of chirality preservation is comparable—though classification may be modestly influenced by CNT array density—indicating that dynamically evolving environments do not measurably increase the incidence of chirality transitions. The residual transitions are consistent with the stochastic, kinetically governed defect formation that persists even under steady growth.

Taken together, the results support a straightforward interpretation: CNT chirality is primarily established during nucleation and remains unchanged throughout subsequent elongation, even under dynamically modulated growth conditions. Because most CNTs initiate growth near the initial 800 °C stage of the 800 °C–873 °C–800 °C temperature protocol, the resulting diameter distribution would be expected to resemble that obtained under constant growth at 800 °C. To test this expectation, we determined the diameter distribution by counting RBM peaks from individual CNTs. The resulting distribution closely matches that measured under steady growth at 800 °C (Supplementary Fig. 4). This agreement reinforces the conclusion that the diameter distribution is governed by the catalyst temperature history prior to nucleation, whereas CNT length is determined by the temperature history during post-nucleation elongation.

Although we observed robust structural memory in the substrate-supported system, tube-substrate interactions can enhance local curvature^42^ that lowers the kinetic barrier for defect formation^28^ (see Supplementary Figs. 15, 16). Chirality preservation in floating-catalyst systems is therefore expected to be at least comparably robust. To examine this expectation, we sparsely deposited CNTs derived from FCCVD onto Si/SiO_2_ substrates using a thermophoretic deposition method^51^, thereby preserving the as-grown structures as much as possible. Raman spectral mapping (Fig. 5c, Supplementary Fig. 3) shows RBM continuity along individual nanotubes, suggesting chirality preservation in the FCCVD system. Unlike substrate-supported growth, FCCVD lacks a clearly defined spatial growth-onset point along the reactor (Fig. 1), implying that the furnace set temperature, or the maximum temperature reached by the catalyst, is not the dominant factor determining nanotube diameter. CNTs were synthesized by FCCVD at four temperatures between 800 and 950 °C and collected on filter paper. Tube diameters were estimated from RBM features in the Raman spectra and compared with the count-based diameter distributions obtained for substrate-supported CNTs (Fig. 5d; Supplementary Figs. 4, 13). FCCVD samples exhibited only minor diameter variation, with an average diameter below 1.2 nm even at 950 °C. This contrasts with the pronounced temperature sensitivity observed for substrate-supported CNTs and supports the proposed interpretation.

As a complementary approach, we performed MD simulations using a machine-learning force field (DeepCNT-22^32,52^), in which an SWCNT with a pre-defined chirality (8,7) is grown from an iron catalyst particle (initially Fe_120_) whose size is allowed to evolve during tube elongation (Fig. 5e, f). In this model, Fe atoms are incrementally added to the catalyst, while carbon atoms are supplied to the catalyst interior at a rate proportional to the catalyst surface area, with unstable carbon atoms being stochastically removed. As a result, the nanotube chirality remains unchanged throughout the simulation, while the catalyst particle grows in size over time (see also Supplementary Movie 1). The time evolution of the system (Fig. 5f) shows that the number of carbon atoms incorporated into the SWCNT (black line) increases in a convex manner, indicating an acceleration of the growth rate as the catalyst grows in size as assumed earlier. Consistent results are obtained for the cases of a (13,0) CNT and a (10,5) CNT under otherwise identical conditions (Supplementary Fig. 18). These results provide mechanistic support that, even under catalyst coarsening, the nanotube diameter does not follow the evolving catalyst size but robustly retains the initially established structure, which is consistent with the experimental observations. Further systematic simulations across different diameters, carbon-supply rates, and temperatures are needed to define the quantitative limits of this structural robustness.

### Structural memory at the single-tube level and implications

The helical topology of the CNT–catalyst interface enables screw-dislocation-like growth^53^ without introducing crystallographic defects, which may underlie the observed contrast between growth kinetics and structural identity. This behavior contrasts with crystal growth in higher dimensions, where temperature and chemical potential typically influence both growth rate and morphology through repeated nucleation events^54^. Even vapor-liquid-solid 1D nanowires adapt their diameter to the evolving catalyst geometry^55^, indicating the importance of interface structure. In this sense, CNT growth may share a few features with biochemical processes. For example, during DNA replication, rapid one-dimensional elongation proceeds strictly at the enzyme-substrate interface, where structural fidelity is maintained because incorrect intermediates are removed before irreversible extension occurs^56,57^. In catalytic CNT growth, elongation occurs at a dynamically reconfigurable CNT–catalyst interface, where transient non-hexagonal defects increase the barrier of subsequent reactions and can be eliminated before being permanently incorporated into the tube wall^28,32,58^ (see Supplementary Fig. 17 for MD simulations). Such dynamic behavior may account for efficient defect healing during one-dimensional crystal growth of CNTs.

**Fig. 6:**
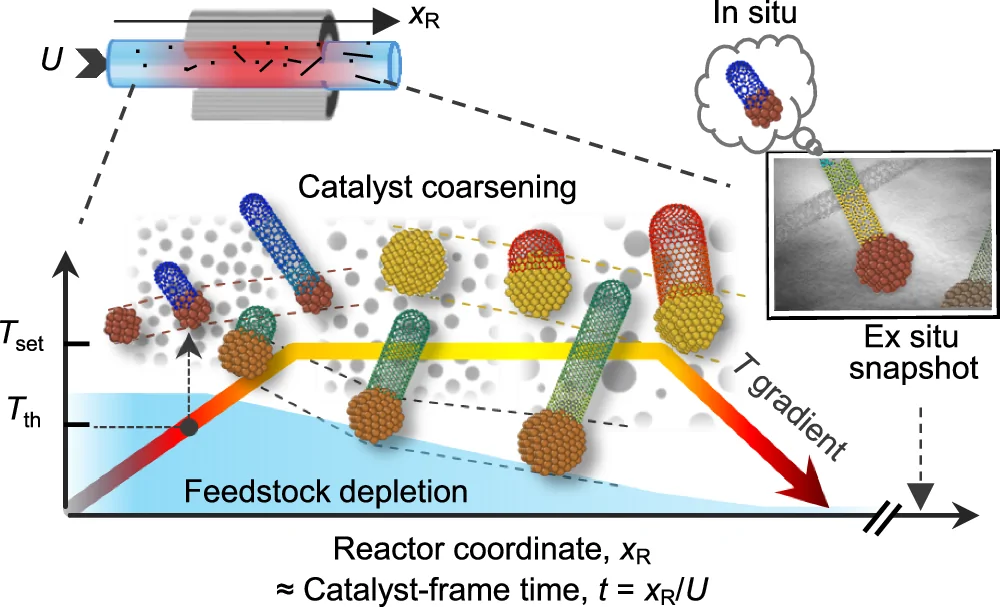
Implications for floating-catalyst systems. Schematic illustration of the proposed growth scenario in a floating-catalyst reactor. Catalyst particles are carried at a flow velocity *U* along the reactor coordinate *x*_R_, so that the time in the catalyst frame is *t* ≈ *x*_R_/*U*. The thick line shows the temperature *T* experienced by the catalyst, which passes a threshold temperature *T*_th_ for nucleation before approaching the furnace set temperature *T*_set_. As catalyst particles coarsen continuously, an ex situ snapshot acquired after growth (right, schematic) compares a carbon nanotube of fixed diameter with a catalyst that has evolved.

Fig. 6 provides a conceptual framework for understanding CNT diameter determination and potential control in dynamic growth systems. In FCCVD, catalyst particles traverse finite axial temperature gradients at the furnace inlet, and nucleation can occur upstream once the gas-phase environment reaches a threshold temperature *T*_th_, independent of the furnace setpoint *T*_set_. As a result, the effective nucleation temperature—and the corresponding catalyst size—can remain effectively constant. This framework explains the weak temperature dependence of the diameter distribution observed in Fig. 5d. Moreover, the diameter increase induced by CO_2_ addition^18^ can be interpreted as delayed nucleation under reduced carbon chemical potential, allowing greater catalyst coarsening before nucleation—analogous to the diameter decrease observed upon addition of a secondary precursor in the eDIPS process^10^. This view is also consistent with the framework of Navas et al., where the diameter distribution of growing nanotubes reflects the active catalyst size distribution as Ostwald ripening continuously reshapes the catalyst population under a given carbon feeding condition^59^.

These considerations suggest that intentional perturbations of the growth environment at the reactor inlet, including precursor concentration and temperature gradients, could be used to confine the nucleation zone, thereby limiting the range of catalyst sizes sampled at nucleation and potentially narrowing the diameter distribution. Complementarily, the robust structural memory allows for independent optimization of the gas-phase carbon activity profile at the subsequent elongation zone to maximize CNT length without disturbing the established diameter. We have seen that the catalyst chemical potential *µ*, which depends on its size and the carbon activity, governs both the growth rate *γ* and the intrinsic growth lifetime in opposite directions (Fig. 3, Supplementary Note 6). Final CNT length is then *γ* integrated over this lifetime, also limited by the extrinsic residence time. An optimal carbon-activity profile therefore depends on the reactor geometry, as well as temperature profile and catalyst evolution along the flow, reflecting a design constraint distinct from that in substrate-supported growth.

Beyond FCCVD-specific implications, the separation of structural determination from kinetic optimization identified here provides a physical rationale for two-stage or seeded growth strategies, in which nucleation and elongation are deliberately decoupled^60–62^. The relationship between CNT diameter and catalyst size, when measured as a snapshot after growth, must be interpreted with caution. CNT–catalyst diameter ratios have been extensively studied using both in situ^37,38^ and ex situ^24,25,63,64^ approaches to establish a foundation for chirality-controlled growth. Ex situ measurements effectively compare a CNT with a fixed diameter to a catalyst particle whose size has continued to evolve over time. Consequently, it is essential to account explicitly for how significantly catalyst coarsening occurs under given growth conditions and the time elapsed since nanotube nucleation when discussing CNT–catalyst diameter relationships.

In conclusion, by tracking individual CNTs under dynamically evolving environments, we have established that diameter and chirality, set at nucleation, are robustly preserved during subsequent elongation, while growth rate responds sensitively to environmental fluctuations. The pronounced temperature dependence and hysteresis of growth rates reflect irreversible catalyst Ostwald ripening rather than intrinsic changes in the growth mechanism, as supported by MD simulations of SWCNT growth from enlarging catalysts. This contrast between structural robustness and adaptive kinetics carries three consequences. First, it provides a design principle that decouples chirality control from yield optimization across separate growth stages. Second, this finding requires reinterpretation of ex situ CNT–catalyst diameter ratios in terms of the catalyst’s temporal evolution rather than static post-growth snapshots. Finally, the robust structural memory suggests a strategy for catalyst-mediated 1D crystal growth: preserving structural fidelity at a reconfigurable interface while allowing kinetic flexibility during elongation. These consequences establish a framework for connecting atomic-scale structural understanding to scalable strategies for structure-controlled synthesis.

## Methods

### Isotope-labeled CNT growth

Aligned CNTs were grown on r-cut quartz substrates patterned with lithographically defined Fe catalysts (nominal thickness 0.1 nm) using alcohol CVD. The catalyst was first reduced at 800 °C in Ar/H_2_ (3%). CNT growth was then initiated with ethanol (FUJIFILM Wako Pure Chemical Corporation, 99.5%) at 5 cm^3^ STP min^−1^ as the carbon source and Ar/H_2_ as the carrier gas. The total pressure was 1480 Pa, with an ethanol partial pressure of ≈135 Pa. Individual CNT growth histories were tracked using a digital isotope-labeling method^39^. Pulses of ^13^C-enriched ethanol (Cambridge Isotope Laboratories, Inc., 1,2-^13^C_2_, 99%, <6% H_2_O) were introduced for 10 s during a programmed heating/cooling sequence of the furnace (see Supplementary Fig. 5 for temperature response of the substrates). Three different ^13^C fractions (33%, 67%, and 100%) were assigned as binary codes of 0 and 1, and a delimiter symbol (#), respectively. In this study, 3-bit codes such as ‘#000’ and ‘#011’ represent the order of introduced isotope labels, enabling reconstruction of CNT elongation with a temporal resolution of 30 s.

### Raman-based reconstruction of growth histories

After growth, CNT arrays were transferred onto Si substrates with a 100 nm-thick oxide layer and analyzed by Raman spectral mapping with a 532 nm excitation wavelength using either a commercial Raman spectrometer (Renishaw, inVia) or a custom-built Raman system. Isotope-labeled segments were identified from corresponding downshifts in the G-mode frequency caused by the heavier carbon isotopes. Raman spectra collected along the same tubes were fitted with Lorentzian functions, yielding the peak frequencies *ω*_G_(*x*), peak heights *h*_G_(*x*), and peak widths *Γ*_G_(*x*) as functions of the distance from the tube root *x*. To determine the precise position of isotope labels, we fitted *ω*_G_(*x*) at the transition from the ^12^C segment to the ^13^C-labeled segments using error functions from the large-*x* side. This procedure allows the onset of isotope labels *x*_*i*_, corresponding to growth times of 30 s, 60 s, etc., to be determined with minimized ambiguity. Distances between adjacent isotope labels (Δ*x*_*i*_ = *x*_*i*_−*x*_*i*+1_) defined the instantaneous growth rates *γ* at a 30 s time resolution. In the displayed data, background signals obtained from Si/SiO_2_ substrates without CNTs under an identical condition were subtracted from the raw spectra to improve clarity and fitting accuracy.

### Floating-catalyst CVD growth of CNTs

CNTs were synthesized by FCCVD for comparison with substrate-supported growth. Ferrocene (Sigma-Aldrich Co. LLC, 98%) and thiophene (Sigma-Aldrich Co. LLC, ≥99%) were dissolved in ethanol (99.5%) at 0.11 and 0.01 wt.%, respectively. The ethanol solution was continuously supplied at a rate of 6 μL min^−1^. The vaporized precursors were introduced into a heated quartz-tube reactor with an inner diameter of 26 mm ( ≈ 130 kPa) with an Ar/H_2_ (3%) carrier gas of 500 cm^3^ STP min^−1^. The quartz tube was heated (800–950 °C) by an electric furnace with a total heated length of 600 mm. CNTs were continuously collected downstream using glass-fiber filter paper for ensemble characterization, with a deposition time of 30 s for each condition.

### Catalyst characterization

To investigate the catalyst size distribution, iron catalysts were deposited on r-cut quartz substrates without spatial patterning. CNTs were grown at different temperatures for the same duration and then burned via oxygen plasma at room temperature so that the catalyst size could be analyzed by atomic force microscopy (AFM). Catalyst heights were analyzed using the grain characterization tool of the Gwyddion software (version 2.60).

### Kinetic Monte Carlo simulations of Ostwald ripening

The stochastic evolution of catalyst nanoparticles was modeled using KMC simulations based on a three-dimensional surface Ostwald ripening model^48^. The simulations explicitly track the size evolution of individual catalyst particles under time-dependent temperature profiles based on the following detachment rate from island *i* with radius *R*_*i*_,

4 $$F\left({R}_{i},{L}_{1,i}\right)=\frac{2{{{\rm{\pi }}}}{v}_{{{{\rm{e}}}}}{a}_{{{{\rm{s}}}}}}{{a}_{{{{\rm{p}}}}}{{\mathrm{ln}}}({L}_{1,i}/{R}_{i})}\exp \left[\frac{{E}_{{{{\rm{c}}}}}-{E}_{{{{\rm{ad}}}}}+2{\gamma }_{{{{\rm{s}}}}}\varOmega /{R}_{i}-{E}_{{{{\rm{d}}}}}}{{{{{\rm{k}}}}}_{{{{\rm{B}}}}}T}\right],$$

where *L*_1,*i*_ is the distance from the island center to the nearest neighboring island edge, *v*_e_ is the attempt frequency of atom detachment, *a*_s_ is sublattice spacing, *a*_p_ is the atomic spacing at island perimeter, *E*_c_ is the cohesive energy of island material, *E*_ad_ is the adsorption energy of adatom on substrate, γ_s_ is the surface energy of the island, *Ω* is the atomic volume, and *E*_d_ is the diffusion barrier on substrate. After detachment, adatom diffusion on the substrate is treated using a first-passage coarse-graining scheme. Temperature varies over time using linear interpolation of a programmed *T*(*t*) profile. With a nominal thickness of 0.1 nm, 100 islands were randomly placed in a 140 × 140 nm^2^ region, with an initial size distribution of 20% relative to the mean size. The same simulation protocol was repeated until sufficient statistics were obtained.

### Molecular dynamics simulations for SWCNT growth

MD simulations were performed using a machine learning force field for iron and carbon atoms, which has been developed by Hedman et al.^32^, implemented through DeePMD-kit^52^ (version 3.1.2) within the LAMMPS (version 29 Aug 2024) framework^65^. To model continuous growth of an SWCNT from an iron catalyst particle under carbon supply, an initial structure was prepared as follows. A short nanotube segment with a predefined chirality was placed with its open edge near the surface of an Fe_120_ nanoparticle, and the system was equilibrated at 1400 K until dissolution of the tube edge into the catalyst had subsided. Starting from this structure, growth was simulated with carbon supply at a given rate and stochastic removal of unstable carbon atoms. Catalyst coarsening was explicitly incorporated by incrementally adding Fe atoms on the catalyst surface during growth. Carbon supply was implemented as a surface-area-dependent insertion process within the catalyst, where ≈4.3 atoms ns^−1^ were added at *t* = 0. Each of unstable carbon atoms was removed stochastically with a rate $A\min [1,\,\exp \left(\frac{E-\mu }{{{{{\rm{k}}}}}_{{{{\rm{B}}}}}T}\right)],$ where *A* (0.069 ns^−1^) is a kinetic prefactor, *E* is the potential energy of each non-CNT carbon atom, and *μ* (−6.6 eV) represents the effective chemical potential of the environment. SWCNT growth simulations were performed at a temperature of 1400 K. OVITO (version 3.15.3) was used to visualize parts of atomic structures and render movies^66^.

### Reporting summary

Further information on research design is available in the Nature Portfolio Reporting Summary linked to this article.

## Supplementary information


Supplementary Information
Description of Additional Supplementary Files
Supplementary Movie 1
Supplementary Movie 2
Supplementary Movie 3
Reporting Summary
Transparent Peer Review file


## Source data


Source Data


## Data Availability

The data that support the findings of this study, including unprocessed raw data, are available from Zenodo^67^ and from the corresponding author upon request. Source data are provided with this paper.

## Code availability

The custom code used in this study is available from Zenodo^67^ and from the corresponding author upon request.
