# Supplementary material for: Isotope-labeled growth histories reveal persistent chirality in individual carbon nanotubes despite catalyst evolution
Source: Nat Commun. 2026 Sep 1;17:8898. doi: 10.1038/s41467-026-77101-2 (PMC13534721; doi:10.1038/s41467-026-77101-2)
Supplement: Supplementary file 1 [file 41467_2026_77101_MOESM1_ESM.pdf]

Isotope-labeled growth histories reveal persistent chirality in individual carbon nanotubes despite catalyst evolution

Keigo Otsuka[1]*†, Ryuji Fujiwara[1]†, Shigeo Maruyama[1,2,3]

[1]Department of Mechanical Engineering, The University of Tokyo, Tokyo, 113-8656, Japan

[2]School of Mechanical Engineering, Zhejiang University, Hangzhou 310027, China

[3]Institute of Materials Innovation, Nagoya University, Nagoya 464-8603, Japan

†These authors contributed equally to this work

* email: otsuka@photon.t.u-tokyo.ac.jp

Supplementary Note 1: Kinetics of floating-catalyst and supported-catalyst growth

To extend the insights obtained from the present supported-catalyst CVD experiments to FCCVD systems, it is necessary that both the chirality determination during nucleation and the subsequent elongation kinetics can be described within a common kinetic framework. To examine this point, we compiled literature data in which CNT growth rates were quantitatively evaluated and compared the growth kinetics between FCCVD and supported-catalyst CVD systems. The dataset includes various growth configurations, such as aligned growth parallel to substrates[4,5], vertically aligned growth[6], kite growth[7], and FCCVD[8–11], as summarized in Supplementary Fig. 1. To account for differences in growth temperature, the reported growth rates were plotted in the form of an Arrhenius plot ($\ln \gamma$ vs $1/T$). The red circles represent the average growth rates obtained in the present supported-catalyst experiments.

As shown in Supplementary Fig. 1, the collected data fall approximately along a single band over a wide temperature range of ≈500–1200 °C. The Arrhenius representation further indicates that these data can be approximately described by a common effective activation energy. These results suggest that, although the absolute growth rates vary depending on experimental conditions, CNT growth in both FCCVD and supported-catalyst CVD systems can be described, at least to a first approximation, within a similar kinetic framework for the rate-limiting process of elongation. At the same time, some scatter is observed in the absolute growth rates at a given temperature. Such variations can be attributed to differences in catalyst composition and size, as well as to the reactivity and partial pressure of carbon source gases and the presence of additives such as sulfur or oxygen. For example, we have reported that the addition of a small amount of acetylene (≈1% relative to ethanol) in alcohol CVD can enhance the growth rate by a factor of 5–10. This type of variation can be interpreted as an effective increase in incoming carbon flux and can be consistently described within the same kinetic framework[5].

We also note that care must be taken when comparing reported growth rates across different studies. In some previous reports[8,12], growth rates are presented in a normalized form assuming a fixed carbon source pressure (e.g., 1 atm), which may lead to overestimation by several orders of magnitude, if the growth rate values are adopted

without recognizing this normalization. In the present comparison, we focus on experimentally measured values to ensure a consistent interpretation. Furthermore, this apparent universality in temperature dependence implies that CNT growth is governed by a common rate-limiting step among multiple elementary processes, such as carbon supply, surface diffusion, and precipitation at the catalyst interface.

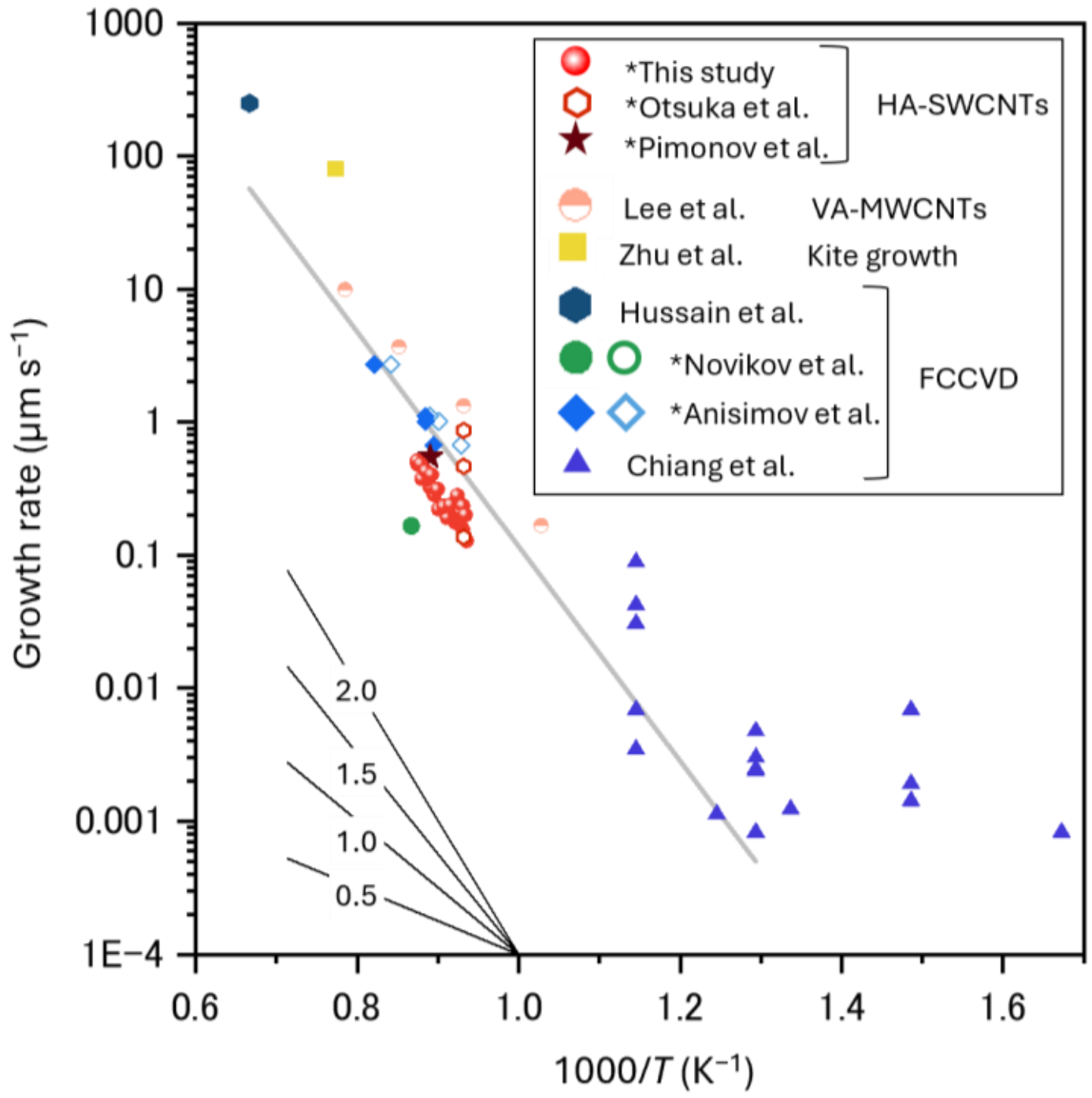

Supplementary Fig. 1 | Comparison of reported CNT growth rates across different synthesis methods. Literature denoted with a star (*) reported the growth of single-walled carbon nanotubes (SWCNTs), otherwise multi-walled carbon nanotubes (MWCNTs) were grown. Despite the differences in growth methods, overall growth rates can be fitted with a single slope, suggesting that the observed growth rates fall within a similar kinetic regime. In the study by Otsuka et al.[5] (open hexagons), a small amount of acetylene was gradually added along with a primary carbon source (ethanol), while maintaining the temperature. In the studies by Novikov et al.[9] and Anisimov et al.[10], upper-bound values are also shown in open symbols, because growth rates and temperatures cannot be uniquely determined. In the study by Chiang et al.[8], growth rates were estimated using dynamic mobility analyzer (DMA), instead of directly measuring the length by microscopic means, potentially leading to overestimation of growth rates obtained for low-temperature growth, where catalyst size cannot be ignored compared with CNT length at growth rates of ≈1 nm s^{-1} or lower.

Supplementary Note 2: Transfer and deposition of CNTs for Raman analysis

CNTs grown on quartz substrates (Supplementary Fig. 2a) are not suitable for Raman analysis of isotope labels for several reasons. First, residual thermal stress induces non-uniform shifts in the Raman spectra[13]. Second, the Raman scattering intensity is relatively weak on quartz. Third, the quartz substrate itself exhibits strong Raman peaks in the radial breathing mode (RBM) region. To mitigate these issues, the CNTs were transferred onto a Si/SiO_2 (100 nm) substrate pre-patterned with metal markers using a thin PMMA film (Supplementary Fig. 2b). A representative Raman map and the corresponding SEM image of the same area are shown in Supplementary Figs. 2c and 2d, respectively. Supplementary Fig. 2e shows the G^+-mode peak frequencies extracted from more than 100 points along the same CNT (tube #1 in Fig. 2d). Except for obvious isotope-induced downshifts, each tube segment shows highly uniform peak frequencies and linewidths owing to the transfer process and a minimum doping effect from Si/SiO_2 substrates. These features enable reliable and spatially resolved identification of chirality preservation or change. In contrast, conventional measurements on as-grown substrates are often affected by residual thermal stress and limited spatial sampling, making it difficult to distinguish extrinsic spectral variations from intrinsic structural changes.

Such quasi-intrinsic states observed for semiconducting CNTs then facilitate the assignment of metallic CNTs only from G-mode features without clear RBM peaks. As shown in Supplementary Fig. 2f, one of the CNTs (at 808 μm within tube #s11 in Supplementary Fig. 10c) shows a Breit-Wigner-Fano peak centered at 1560–1570 cm^{-1}, as well as an RBM peak within the M_{22} region, which is likely to be a metallic (19,10) or (23,5) CNT. Although RBM features are absent in another CNT (at 1404 μm within tube #s4 in Supplementary Fig. 10c), a similar BWF peak is observed across the length over ≈100 μm. Considering the negligible extrinsic perturbation in our samples and uniform spectra, this CNT is very likely to be a metallic CNT.

For FCCVD-derived CNTs, we performed similar Raman mapping measurements. Typically, CNTs synthesized via FCCVD are collected using membrane or glass-fiber filters; the ensemble Raman spectra shown in Supplementary Fig. 13 were obtained from such samples. However, as this study focuses on whether chirality remains continuous along a single CNT, it is essential to observe the nanotubes in an isolated morphology.

Although isolation can be achieved by dispersing bundled samples from thick films into a solvent and then depositing them onto a substrate, this process involves a high possibility of cutting the CNTs, making them unsuitable for intended analysis along a single CNT. We therefore employed a direct dry deposition method via thermophoresis[14] during low-concentration CNT synthesis. Supplementary Fig. 3a shows an SEM image of the as-deposited CNTs. The G-mode intensity map for the region enclosed by the yellow rectangle is presented in Supplementary Fig. 3b. By tracing the Raman spectra along the axis of the CNT(s) indicated by the arrows, we observed approximately four distinct RBM peaks. Notably, the peak at 174 cm^{-1} remains continuous from one end to the other, indicating that the chirality of this long CNT is preserved throughout its length (Supplementary Fig. 3c). The other RBM peaks likely originate from short CNTs that are locally bundled or crossed with the long nanotube.

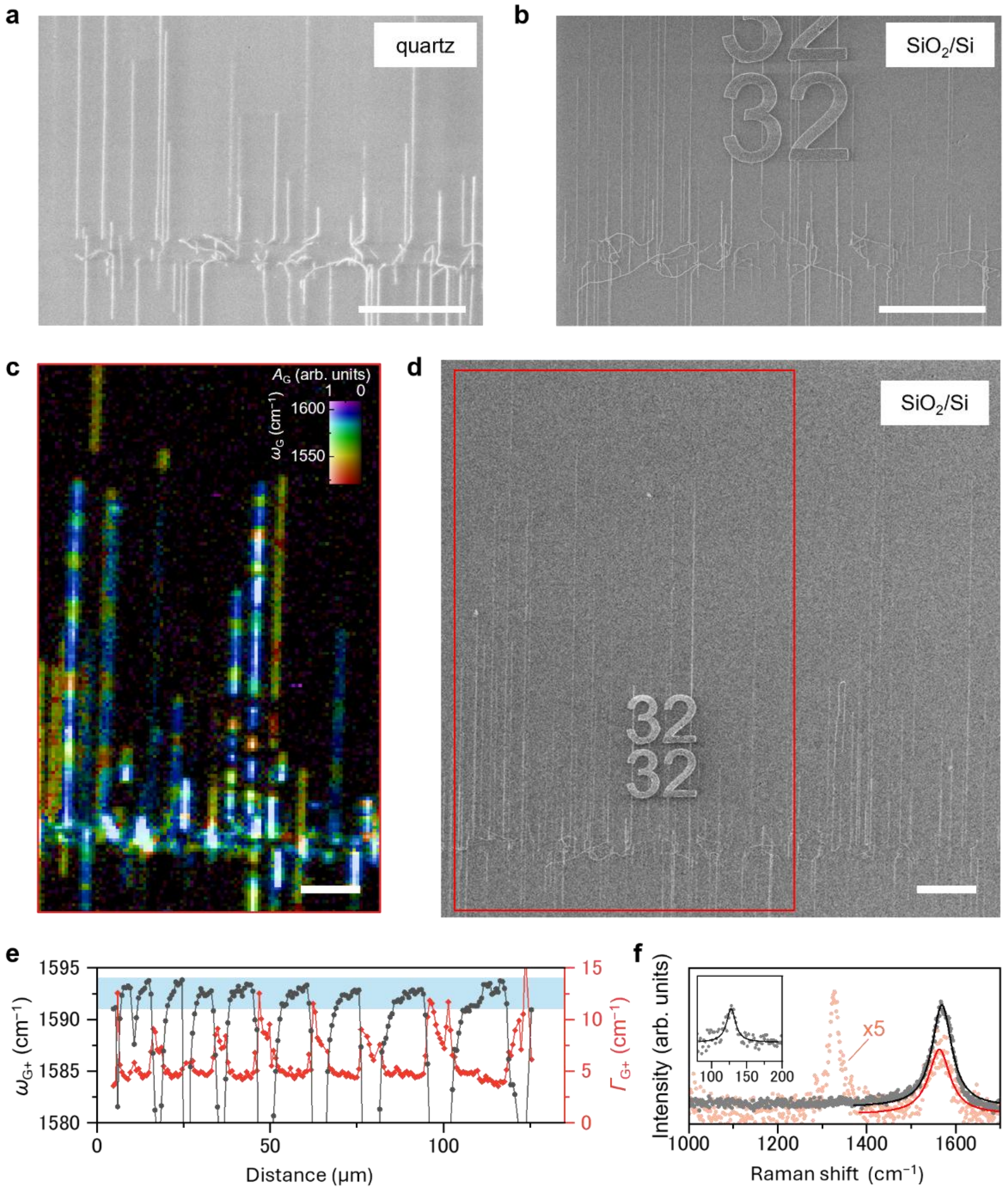

Supplementary Fig. 2 | Aligned, isolated CNTs for isotope labeling analysis. a SEM image of as-synthesized carbon nanotubes (CNTs) obtained from an experiment with a temperature profile of 800-873-800 °C. **b** SEM image of the CNTs on the same substrate after transfer onto a Si/SiO_2 substrate for Raman analysis. **c**, **d** Corresponding Raman mapping image (**c**) and SEM image (**d**) of the identical area. In the Raman map, the color represents the G-mode peak frequency, and the brightness indicates its intensity. **e** G^+-mode peak frequencies ω_{G+} and linewidths Γ_{G+} acquired along tube #1 in the main text (Fig. 2). Despite the mixed supply of ^{13}C-enriched ethanol, most of measured spectra peak within the blue shaded region, which indicates a 3 cm^{-1} width. **f** Raman spectra obtained

from a metallic CNT (gray, tube #s11 in Supplementary Fig. 10), which shows an RBM peak at 127 cm^{-1} (diameter of ≈2.03 nm), and from a metallic-like CNT (light red, tube #s4 in Supplementary Fig. 10) judged from G-mode shape. These spectra are averaged over 4.2 (7 points) and 9 µm (15 points) along their axis, respectively. Excitation wavelength of 532 nm was used. All scale bars are 10 µm.

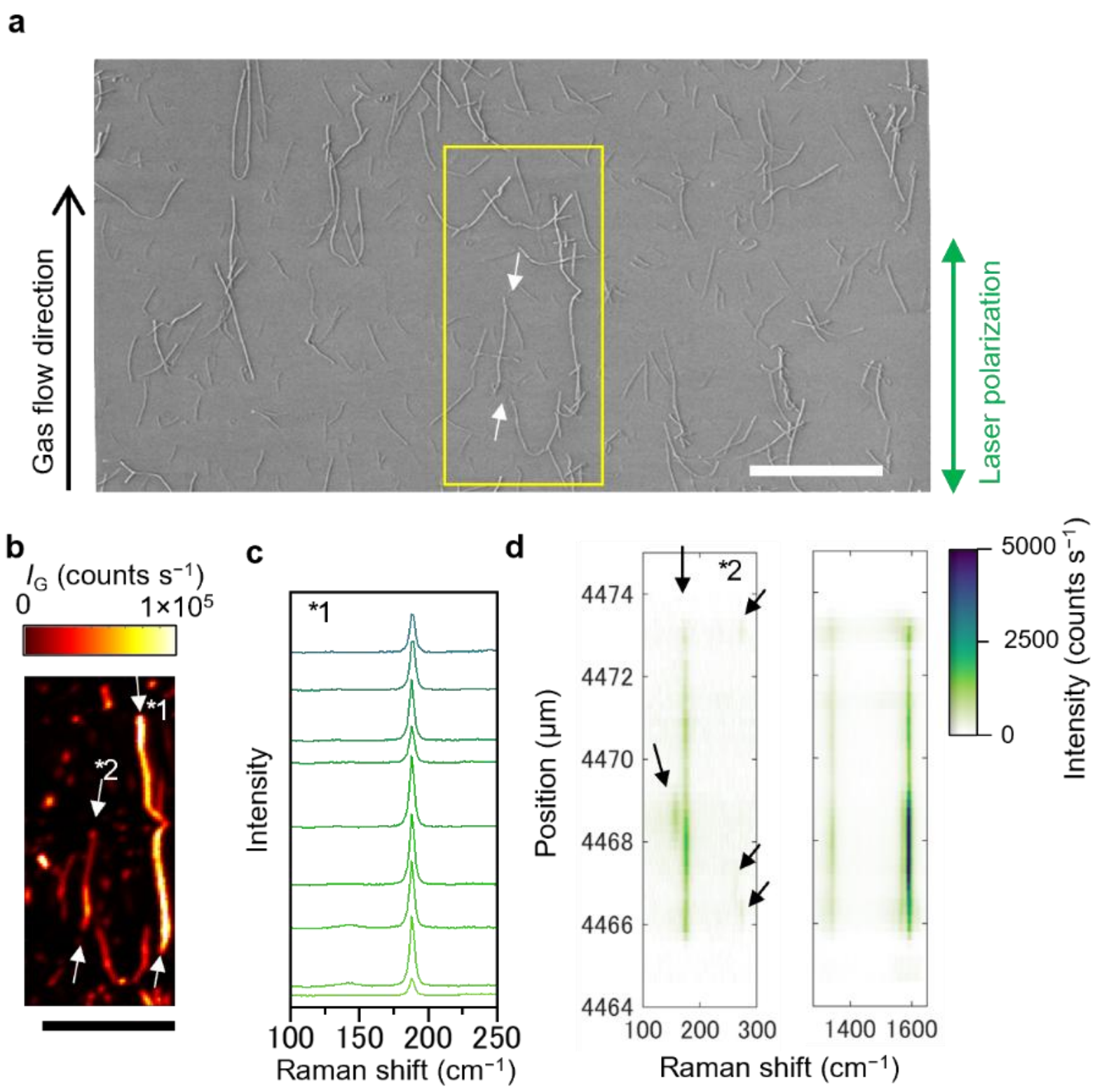

Supplementary Fig. 3 | As-grown CNTs on a substrate derived from FCCVD. a SEM image of CNTs synthesized by FCCVD and directly deposited onto a Si/SiO_2 substrate via thermophoresis. The gas flow direction is from the bottom to the top of the image. Scale bar is 10 µm. **b** Raman mapping image (integrated G-mode intensity I_G) of the region enclosed by the yellow rectangle in panel **a**. The area corresponds to that shown in Fig. 5d of the main text. Excitation laser was linearly polarized parallel to the gas flow direction. **c** Raman spectra measured at different points along the CNT denoted with arrow *1 in panel **b**. **d** Raman spectra acquired along the axis of the CNT indicated by the arrow *2 in panel **b**. The spectra reveal several short CNTs (approximately 1 µm) in close proximity to a longer CNT (>7 µm). Excitation wavelength of 532 nm was used.

Supplementary Note 3: Estimation of semiconducting-to-metallic CNT ratios

To assess whether the growth behaviors discussed in the main text exhibit any dependence on the electronic type of CNTs, we first compared the abundance of semiconducting (s-) and metallic (m-) CNTs by scanning the excitation laser perpendicular to the CNT arrays separated 10 μm away from the catalyst stripe. The electronic type was assigned based on the detected counts of RBM peaks using the Kataura plot for samples grown at fixed temperatures of 800 °C and 875 °C. Using the dataset shown in Supplementary Fig. 4a,b using different excitation wavelengths (532, 633, and 785 nm), CNTs were assigned as semiconducting or metallic within each diameter range based on their RBM frequencies. For each excitation wavelength, the fraction of semiconducting CNTs is indicated in the figure. Furthermore, by weighing the number density of CNTs detected per scan length with these excitation wavelengths, the overall semiconducting-to-metallic ratio was estimated. This analysis yields ratios of 64:36% at 800 °C and 63:37% at 875 °C, corresponding closely to the expected 2:1 ratio and showing no discernible temperature dependence. To examine the effect of temperature profiles, the diameter distribution of CNTs grown under modulated temperature (800–873–800 °C protocol in Fig. 2) is compared with those under constant temperatures (Supplementary Fig. 4c). Synthesis with the modulated temperature yielded a comparable diameter to the 800 °C case.

Since the growth rates of m-CNTs and s-CNTs, as well as their temperature dependence, are also almost identical within the temperature range examined (Fig. 2g), the unchanged semiconducting-to-metallic ratio suggests that other features, such as growth lifetime, are not expected to depend on the tube metallicity within this temperature range. We thus separately analyzed the growth termination timing in Supplementary Fig. 4d. Although the distributions of m- and s-CNTs are not identical, the differences may arise from sampling biases related to Raman intensity and finite tube length. In addition, apparent E_a values extracted for T ramp-up and ramp-down periods are separately analyzed for m-CNTs and s-CNTs (Supplementary Fig. 4e), exhibiting a similar hysteretic behavior. These results are fully consistent with the observation that both growth rate and growth lifetime exhibit negligible metallicity-dependent differences in this temperature range.

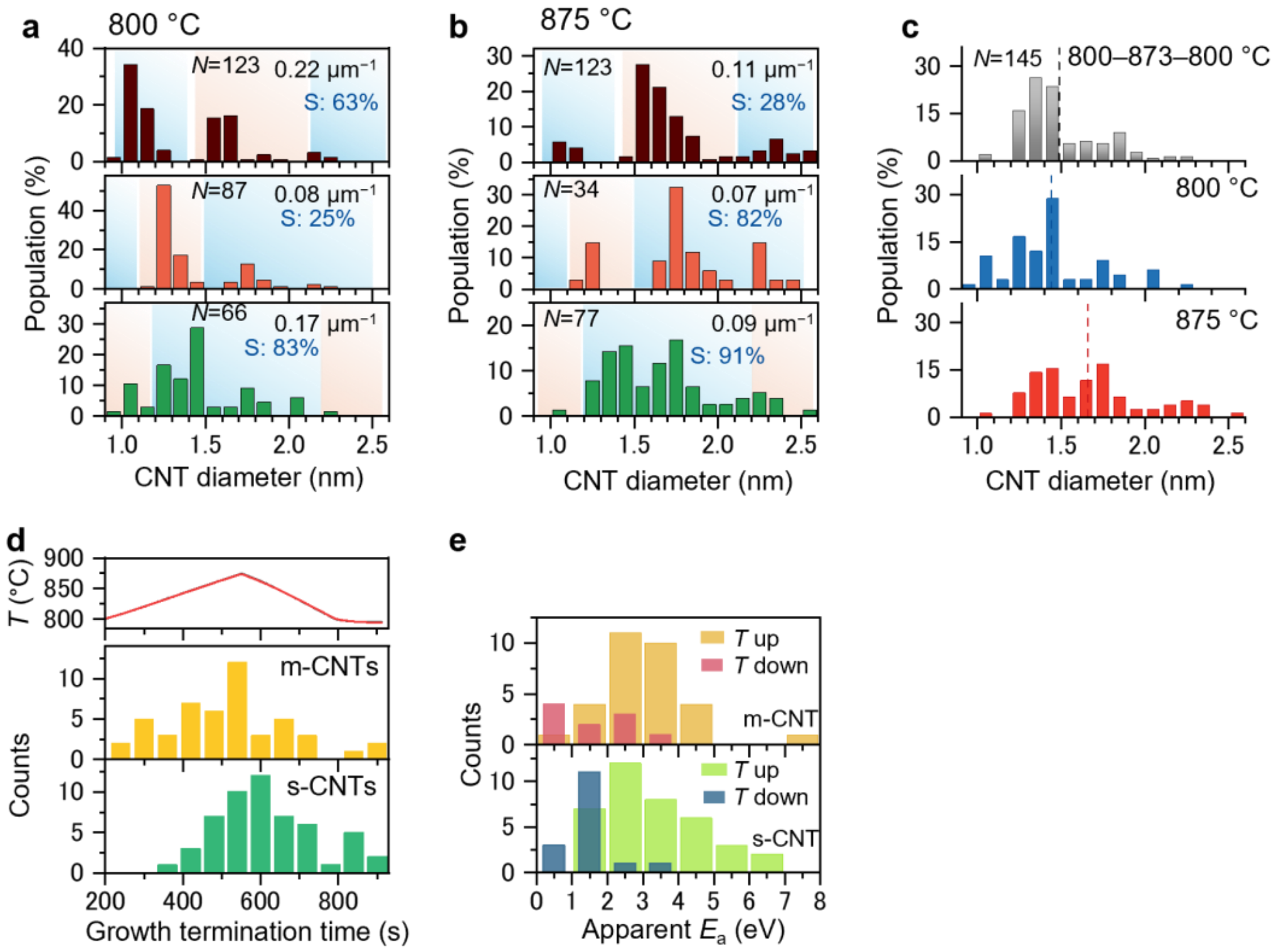

Supplementary Fig. 4 | Electronic-type distributions from RBM data. a,**b** Diameter distributions of CNTs derived from Raman RBM counts for samples synthesized at 800 °C (**a**) and 875 °C (**b**). Metallic and semiconducting diameter regions are indicated by red and blue shaded backgrounds, respectively. The fraction of semiconducting CNTs is shown for each excitation wavelength (785, 633, and 532 nm from top to bottom). The density (μm^{-1}) of parallel CNT arrays is shown inside the graphs. **c** Diameter distribution of CNTs grown under three different temperature profiles. Excitation wavelength of 532 nm is used. Average diameter is indicated by the vertical dashed lines. **d**,**e** Histograms of growth termination times (**d**) and apparent activation energy E_a (**e**) for m- and s-CNTs. E_a is separately extracted during T ramp-up and ramp-down periods.

Supplementary Note 4: Temperature response of substrates inside the furnace

To assess the potential influence of finite thermal response time on the temperature dependence of CNT growth rates under dynamically modulated temperatures, we constructed a thermal model that captures the heat-transfer processes in our experimental configuration. Supplementary Fig. 5a illustrates the experimental setup, where the substrate is placed in the central region of the first furnace. Thermocouples positioned near each furnace monitor the nominal furnace temperatures, while the actual temperature experienced by catalyst nanoparticles deposited on the substrate surface may deviate to some extent because of finite heat-transfer rates. To evaluate this deviation, we model the system as a planar substrate exposed symmetrically to a heat source with temperature of $T_F(t)$ (Supplementary Fig. 5b). Heat transfer is assumed to occur via (i) radiative exchange with the heat source and (ii) gas-phase heat transfer, as well as one-dimensional heat conduction across a solid substrate. The furnace interior is approximated as an effective blackbody (ε=1), which is justified by multiple reflections within the confined geometry and the relatively small size of the substrate compared to the enclosure. The substrate consists of a quartz plate (thickness 500 μm) placed on a heavily doped silicon substrate (thickness 525 μm). Based on literature values[15], the effective emissivity of silicon at elevated temperatures is taken as ε=0.7, while that of quartz is treated as a parameter (ε=0.1 for a conservative case).

Supplementary Fig. 5c (bottom panel) shows the simulated time evolution of the quartz surface temperature compared to the programmed furnace temperature. Despite the imposed temperature modulation, the surface temperature closely follows the furnace temperature. The corresponding temperature difference $\Delta T = T_F - T_{surface}$ is plotted in Supplementary Fig. 5c (top panel). Three representative cases are considered: (1) a conservative scenario including the silicon substrate (gas-phase heat transfer neglected), (2) a case without the silicon support (lower heat capacity, but low absorption), and (3) an optimistic case including gas-phase heat transfer and a higher absorption by quartz (ε=0.4). The use of silicon support improves the thermal response of quartz substrates, with maximum temperature deviation less than 3 K. The internal thermal conduction within the substrate occurs on a sub-second timescale due to the small thickness, and the overall response is governed by external heat transfer, leading to a characteristic timescale

on the order of a few seconds. This timescale is substantially shorter than both the temperature modulation period (~100–300 s) and the isotope-labeling interval (30 s).

To directly evaluate the impact of such thermal lag on the extracted growth kinetics, we computed Arrhenius plots with and without incorporating the thermal response delay (Supplementary Fig. 5d). The comparison shows that the inclusion of a conservative delay leads to only a minor shift in the data, without altering the overall slope corresponding to the apparent activation energy (E_g=1.6 eV). Importantly, the key observation in this study—the hysteresis between temperature ramp-up and ramp-down processes (Fig. 3c–e)—cannot be explained by such a small and uniform temporal lag. A simple delay would affect both heating and cooling branches in a similar manner, whereas the experimentally observed asymmetry reflects an intrinsic evolution of the catalyst state.

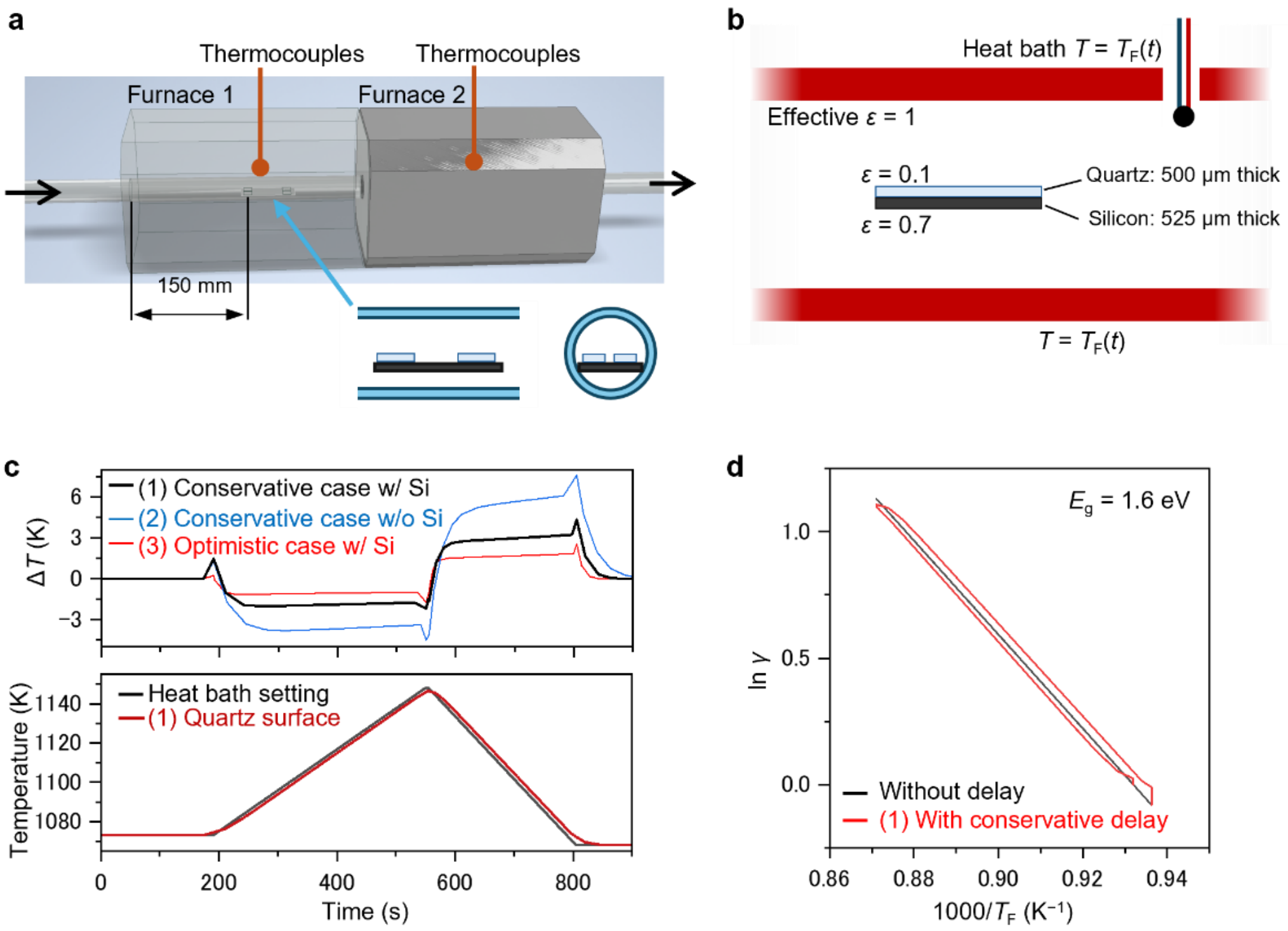

Supplementary Fig. 5 | Thermal response of the substrate under programmed furnace temperature. **a** Schematic of the two-zone furnace setup and substrate placement inside the quartz tube. **b** Simplified thermal model describing radiative and gas-mediated heat transfer between the furnace and a quartz/Si substrate stack. **c** Simulated temperature deviation (top) between the furnace setpoint and the quartz surface (bottom) for three different assumptions on heat transfer. **d** The corresponding Arrhenius plots comparing cases with and without thermal delay.

Supplementary Table 1 | Parameters used for three different scenarios. ε, κ, c_p, t_{sub} represent emissivity, which is equal to absorptivity within the temperature range considered, thermal conductivity, specific heat capacity, and thickness of quartz and silicon substrates, respectively.

Case	Quartz				Silicon				
	ε	κ (W m^{-1} K^{-1})	c_p (J kg^{-1} K^{-1})	t_{sub} (μm)	ε	κ (W m^{-1} K^{-1})	c_p (J kg^{-1} K^{-1})	t_{sub} (μm)	h (W m^{-2} K^{-1})
(1)	0.1	3.0	1000	500	0.7	20	950	525	0.0
(2)	0.1	3.0	1000	500	-	-	-	-	0.0
(3)	0.4	4.0	1000	500	1.0	30	950	525	5.0

Supplementary Note 5: Temperature dependence of growth rates and lifetime

If the anomalous acceleration of growth rate and the observed hysteresis can be attributed to catalyst coarsening at elevated temperatures, the Arrhenius plot of the growth rate is expected to deviate from linearity and become downward-convex during temperature ramp-up. This is because Ostwald ripening is accelerated at higher temperatures, which should enhance the growth rate more significantly.

Supplementary Fig. 6a presents Arrhenius plots of the growth rate for a larger number of individual CNTs. First, all the CNTs displayed in the bottom right panel grew faster during T ramp-down than during ramp-up when compared at comparable temperatures, indicating the coarsening of the corresponding catalyst. Since the intrinsic E_a is estimated to be ≈1.6 eV (Fig. 4h), more than 80% of CNTs in the upper three panels suggest the coarsening of catalysts (apparent E_a > 2 eV). Second, a weak tendency is observed in which the slope becomes slightly steeper on the high-temperature side. To further extract this feature, we focused on the heating process and plotted the apparent activation energy E_a of each CNT against the average temperature of its corresponding growing duration (Supplementary Fig. 6b). Although the data show considerable scatter, CNTs growing at higher temperatures tend to exhibit slightly larger apparent activation energies, whereas apparent E_a during T ramp-down is much smaller at high temperatures.

It should be noted, however, that the increase in the average catalyst particle radius $\bar{R}$ due to Ostwald ripening follows a sublinear power-law dependence on time, with an exponent of 1/4 or smaller, as discussed later. Consequently, growth acceleration in the later high-temperature stage is not expected to be abrupt but is instead governed by stochastic variations among individual CNTs. Supplementary Fig. 6c compares the apparent activation energies of CNTs that grew across both T ramp-up and ramp-down stages, evaluated separately for each stage.

Supplementary Fig. 6d shows the number of growth-termination events D(t) and the number of actively growing CNTs $N(t)$ as a function of time for a control experiment in which the temperature was held constant at 800 °C for 24 min. Using this data, the survival probability p over a unit time of τ = 1 s was calculated in the same manner as in the main text, and its temporal evolution is plotted in Supplementary Fig. 6e. For comparison, Fig. 3c is replotted with time as the horizontal axis. In the initial stage at

800 °C, both experiments show similar values of $p \approx 99.9\%$, corresponding to a growth lifetime of ≈15 min. While this high survival probability is maintained under constant-temperature conditions, a pronounced decrease in p is observed when the temperature is increased, highlighting the strong impact of high temperatures on growth termination. In addition, the survival probability obtained for the T=800 °C for 24 min, but with 5-cm^3 STP min^{-1} CO_2 addition from 6 min[16], is compared (green circles). Growth lifetime extension by CO_2 is also confirmed in the form of survival probability.

Under constant temperature conditions, one might also expect that CNTs should exhibit changes in growth rates. Supplementary Fig. 6f shows growth rate evolution of representative CNTs grown at 800 °C[16], indicating a gradual acceleration of the growth rate for some CNTs. To quantify this behavior, the change in growth rate evaluated within each nanotube over one-minute intervals is averaged across all analyzed CNTs (Supplementary Fig. 6g). The resulting distribution shows a finite fraction of positive rate changes, indicating that non-negligible growth acceleration occurs even under constant temperature conditions.

Because the confidence interval of any single 30-s bin is too wide to establish a trend on its own, we instead compared the systematic difference in the mean survival probability p between the ramp-up and ramp-down stages by pooling the termination statistics over a common temperature band. For each stage, we summed the termination events D and the numbers of growing nanotubes N over the bins, evaluated a pooled rate $q_{pool} = \Sigma D/(\Delta t \cdot \Sigma N)$, and obtained the mean survival probability $p = \exp(-q_{pool} \cdot \tau)$ with an exact 95% confidence interval (CI) on the Poisson count ΣD. The two branches give clearly non-overlapping intervals, p_{up} = 99.87% (95% CI 99.76–99.94%) and p_{down} = 99.19% (95% CI 98.76–99.50%) over 818.9–850.1 °C, and the separation is preserved when the band is narrowed (Supplementary Table 3). This significant difference in growth lifetime indicates that an irreversible change occurred between the two stages; together with the concurrent hysteretic increase in growth rate, it is most reasonably attributed to catalyst coarsening.

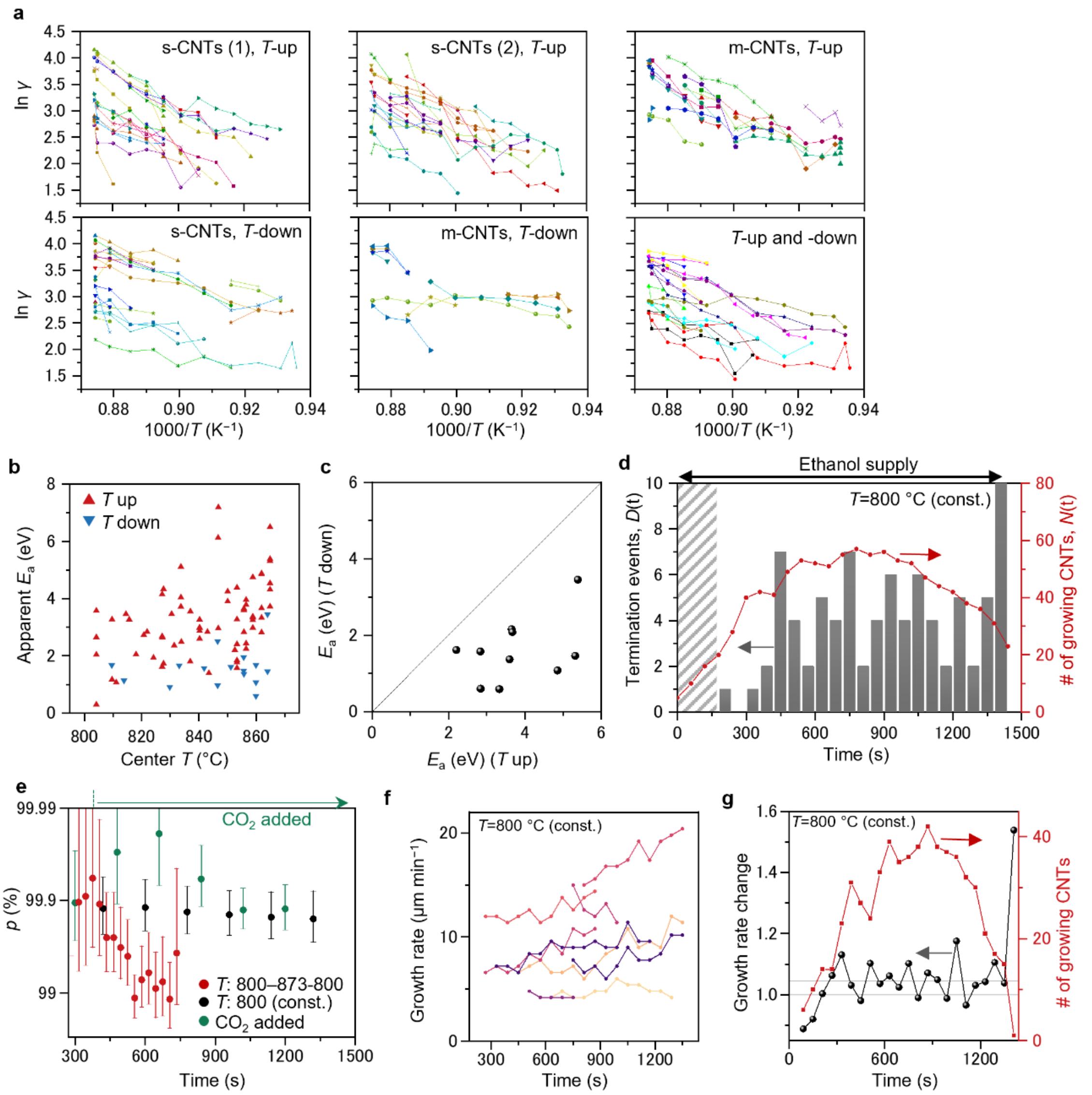

Supplementary Fig. 6 | Arrhenius and survival analyses supporting kinetic interpretations. a Arrhenius plots of the growth rate for a large number of individual CNTs. For clarity, panels are separated for s-CNTs and m-CNTs, as well as for *T* ramp-up and *T* ramp-down. All CNTs whose apparent E_a for both *T* ramp-up and ramp-down are evaluated are plotted in the bottom right panel. **b** Apparent activation energy E_a plotted against the average temperature of each growth segment during *T* ramp-up (red) and ramp-down (blue) periods. **c** Comparison of apparent activation energies E_a extracted from the *T* ramp-up and ramp-down periods for CNTs that grew across both stages. **d** Time evolution of the number of growth-termination events $D(t)$ and actively growing CNTs $N(t)$ for growth at a constant temperature of 800 °C. Δt is 60 s for this experiment. **e** Temporal evolution of the survival probability p calculated from the data in panel **d**,

together with a time-replotted version of Fig. 3c and data obtained with CO_2 addition from 360 s for comparison. Error bars represent 95% confidence intervals. **f** Growth rate evolution of representative CNTs grown under a constant temperature[16]. **g** Ensemble average of growth rate change per one minute within the same CNTs[16].

Supplementary Table 2 | Data used for Fig. 3c, including *t*, *T*, *D*, *N* for each time bin (30 s).

**Time** t (s)	**Temperature** T (°C)	**Termination counts in 30 s** $D(t)$	**Growing CNT counts** $N(t)$
15	799.2	-	-
45	799.1	-	-
75	799.1	-	-
105	799.1	-	-
135	799.1	0	5
165	799.1	0	8
195	801.0	0	11
225	805.6	0	15
255	811.4	0	22
285	817.5	0	31
315	823.9	1	32
345	830.4	1	37
375	837.1	1	58
405	843.6	2	61
435	850.1	5	66
465	856.6	5	66
495	863.0	6	62
525	869.3	7	58
555	870.5	15	44
585	864.8	8	37
615	856.7	6	33
645	847.9	7	26
675	838.5	5	22
705	828.8	7	20
735	818.9	2	18
765	808.9	0	18
795	801.1	-	-
825	797.2	-	-
855	795.6	-	-
885	795.0	-	-

Supplementary Table 3 | Pooled growth-survival statistics for the ramp-up and ramp-down stages. CI represents the confidence interval.

Temperature band	branch	Σ*D*	Σ*N*	Mean *p*	95% CI
818.9–850.1 °C	Ramp-up	10	254	99.87%	99.76%–99.94%
	Ramp-down	21	86	99.19%	98.76%–99.50%
823.9–847.9 °C	Ramp-up	5	188	99.91%	99.79%–99.97%
	Ramp-down	19	68	99.07%	98.56%–99.44%

Supplementary Note 6: Modeling hysteresis of growth lifetime

In the main text, we attributed the hysteresis observed in the growth rate of individual CNTs to irreversible coarsening of the catalyst particle. Importantly, the same physical picture can also account for hysteresis in the ensemble growth lifetime. Here, we examine the mechanisms responsible for CNT growth termination and show that catalyst coarsening provides a unified explanation for the hysteretic behavior observed in both the growth rate and the growth lifetime. We begin by considering the possible processes that can terminate CNT growth on a metal catalyst particle. In general, growth termination occurs through several distinct mechanisms, including (i) encapsulation of the catalyst surface with graphene-like overlayer, (ii) detachment of the CNT from the catalyst particle accompanied by defect formation and a loss of diameter matching, and (iii) disappearance of the catalyst particle itself, for example due to evaporation or Ostwald ripening. Introducing the characteristic time constant τ_i associated with each process, the overall catalyst lifetime τ can be expressed as,

$$\tau_{\mathrm{life}}^{-1} = \sum_i \tau_i^{-1}. \qquad \cdots (2)$$

In the present experiments, abrupt changes in CNT diameter are rarely observed, indicating that CNT detachment associated with significant structural reconstruction plays a negligible role compared with other mechanisms. In addition, since defect healing is kinetically enhanced at higher temperatures[17,18], defect-induced termination is unlikely to be the dominant origin for the lower survival probability p observed at higher temperatures (Fig. 3c). Furthermore, we suppose CNTs analyzed in this study to nucleate and grow from relatively large catalyst particles, for which complete catalyst disappearance through Ostwald ripening can be ignored. Under these conditions, the catalyst lifetime is dominated by surface passivation by graphene, and the growth termination rate q can be approximated as

$$q = \tau_{\mathrm{life}}^{-1} \simeq \tau_{\mathrm{pass}}^{-1}. \qquad \cdots (3)$$

In the following, we associate the passivation rate $\tau_{\mathrm{pass}}^{-1}$ with the nucleation frequency J of a graphene overlayer on the catalyst surface.

$$\tau_{\mathrm{pass}}^{-1} \simeq J. \qquad \cdots (4)$$

To estimate J, we model the formation of a graphene overlayer as a two-dimensional nucleation process occurring on the catalyst surface. Within this framework, the nucleation frequency can be expressed as

$$J = \nu_0 N_s \exp(-\frac{\Delta G^*}{k_B T}), \quad \cdots (5)$$

where ν_0 is an attempt frequency, N_s is the number of available nucleation sites on the catalyst surface. ΔG^* is the nucleation barrier and can be given by

$$\Delta G^* = \pi \frac{\sigma^2 \Omega}{\Delta\mu}, \quad \cdots (6)$$

where σ is the graphene edge free energy, Ω is the atomic area of graphene, and $\Delta\mu$ is the chemical potential difference driving graphene nucleation on the catalyst surface. Assuming that the density of nucleation sites is approximately uniform, N_s is taken to scale with the effective catalyst surface area A as $N_s = A/\Omega$. Compared with the chemical potential of graphene μ_{gr}, we defined that of a CNT as $\mu_{cnt} = \mu_{gr} + \Delta\mu_{cnt-gr}$, where $\Delta\mu_{cnt-gr}$ (0.07 eV) reflects the curvature-induced destabilization of CNTs relative to graphene, corresponding to 1-nm-diameter tubes.

To connect this thermodynamic convention to experimentally accessible quantities, we use the framework introduced in our previous study[5], where carbon concentration N within the catalyst particle and equilibrium concentration N_{eq} are used to describe the driving force of CNT growth ($\gamma = k_g(N - N_{eq})$) as,

$$N = \frac{A k_{ad} P_C + N_{eq} k_g D}{k_g D + A k_{de} P_E}, \quad \cdots (7)$$

where A is the active area of the catalyst surface, D is the number of carbon atoms per unit length ($\propto$ tube diameter). P_C and P_E are effective concentration of carbon sources and etching agents in gas phase, respectively, and k_{ad}, k_{de}, and k_g are the kinetic constants for carbon adsorption, desorption, and precipitation as CNTs, respectively. At low-pressure CVD processes, where P_E is small, the driving force for CNT growth ($N - N_{eq}$) is roughly proportional to A as assumed in the main text (Supplementary Fig. 7a). Here, "low P_E" corresponds to the growth condition with 5 cm^3 STP min^{-1} ethanol (partial pressure of ≈135 Pa). The other two conditions use 10× smaller or larger P_E, while P_C is chosen so that γ becomes 10 μm min^{-1} at A=A_0. Note that in the low-P_E limit, the

sensitivity $S_A \equiv \partial \ln\gamma / \partial \ln A = k_g D/(k_g D + A k_{de} P_E)$ approaches unity, indicating that the growth rate scales with the catalyst surface area, and the assumed proportional relationship is roughly valid for the present experimental conditions. Note that when the sensitivity to k_g is defined ($S_g \equiv \partial \ln\gamma / \partial \ln k_g$), $S_A + S_g = 1$, indicating the complementary features that depend only on dimensionless ratio $A k_{de} P_E / k_g D$.

Here, we simply assume that $\mu_{cnt} = \mu^o + k_B T \ln N_{eq}$ and chemical potential on the catalyst during CNT growth is written as

$$\mu(N) = \mu^o + k_B T \ln N = \mu_{gr} + k_B T \ln(N/N_{eq}) + \Delta\mu_{cnt-gr}. \quad \cdots (8)$$

Therefore, driving force for the nucleation of graphene on the catalyst surface is

$$\Delta\mu = k_B T \ln(N/N_{eq}) + \Delta\mu_{cnt-gr}. \quad \cdots (9)$$

In the present experiment, average growth rates increased from ≈10 to ≈14 μm min^{-1} at 800 °C before T ramp-up and after T ramp-down, indicating the catalyst coarsening. The $\Delta\mu$ values are plotted as a function of normalized surface area of the catalyst A/A_0 in Supplementary Fig. 7b. Because the experimental growth termination probability at 1 s intervals (= τ) was ≈0.1% (Supplementary Fig. 6e), we adjusted σ to be 0.1954 eV Å^{-1}, which is smaller than the DFT-based value (≈0.4 eV Å^{-1} for graphene on Fe surface). By substituting Supplementary Equations 6 and 9 into Supplementary Equation 5, we can estimate the J and p (=e$^{-J\tau}$) values as shown in Supplementary Fig. 7c,d, respectively. In order to estimate the average CNT length, we can also implement other time constants τ_2 (e.g., 900 s and 10 s) imposed by other mechanisms for growth termination, including the nanotube detachment from catalysts and residence time in FCCVD systems (Supplementary Fig. 7e). Furthermore, by considering increase in $\bar{R}$ due to Ostwald ripening (Supplementary Note 7), we can replot the p evolution as a function of elapsed time (Supplementary Fig. 7f). In contrast to such spontaneous catalyst coarsening, changes in gas composition provide an experimentally accessible perturbation to the growth environments. We therefore revisit the effect of CO_2 on the growth lifetime[16]. To estimate the impact of CO_2-induced lifetime extension and compare it with experimental observations, the evolution of p is plotted for a representative case in which CO_2 was introduced at 6 min in a manner similar to Supplementary Fig. 6e. The change in $\Delta\mu$ by CO_2 addition is estimated from the experimental change in the growth rate, further indicating the reasonable consistency of the present rate-lifetime model. In

addition, this framework is consistent with the TEM observation by Navas et al.[19], where a significant evolution of the particle size distribution and encapsulation of large catalyst particles by carbon layers were observed.

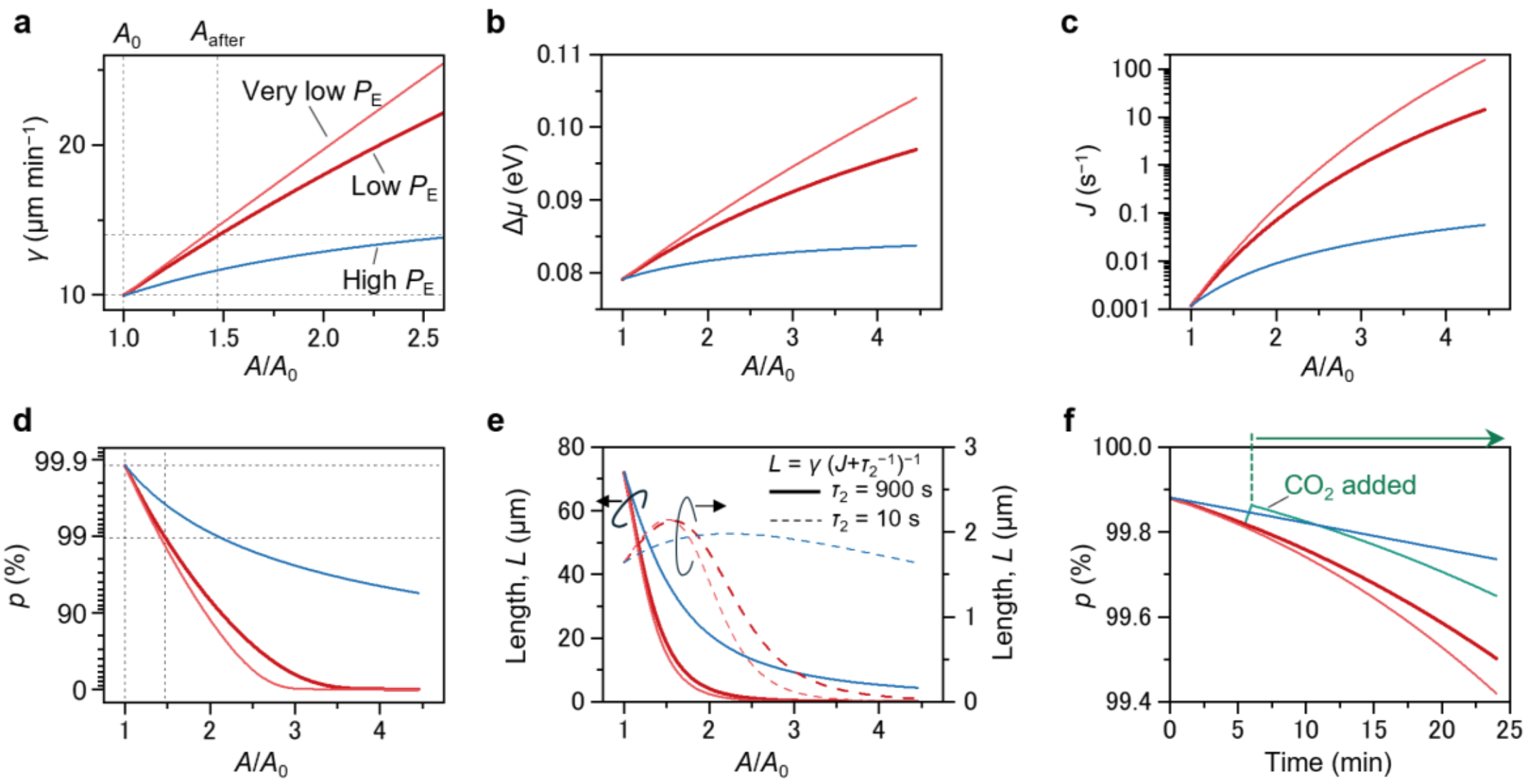

Supplementary Fig. 7 | Modeling of growth rates and lifetime. a Growth rate γ derived from Supplementary Eq. 7 as a function of normalized area A/A_0 of catalyst nanoparticles. Three different levels of P_E are considered. **b** Driving force for graphene nucleation $\Delta\mu$ on the catalyst surface. **c** Nucleation frequency of graphene J on catalyst nanoparticles. **d** Survival probability p at 1 s intervals. **e** Predicted CNT lengths as the product of growth rates and growth lifetime for different τ_2. **f** Time evolution of p when considering catalyst coarsening via Ostwald ripening at 1073 K. The case corresponding to constant T, but with 5-cm^3 STP min^{-1} CO_2 addition from 6 min, is also plotted (green).

Supplementary Table 4 | Parameters used to connect growth rates to growth lifetime. The values with “*” can be adjusted depending on each other, and only their relative values should be considered.

**Symbol / Parameter**	**Value**	**Unit**	**Physical meaning**
T	1073	K	Temperature
D	1.0*	nm^{-1}	Number of atoms per unit tube length (proportional to diameter)
N		nm^{-2}	Surface carbon concentration on the catalyst nanoparticle
N_{eq}	30*	nm^{-2}	Equilibrium surface carbon concentration on the catalyst
A_0	14.14*	nm^2	Initial surface area of catalyst
k_{ad}	1.6*	$nm^{-2}\cdot s^{-1}\cdot Pa^{-1}$	Kinetic constant for carbon adsorption
k_{de}	0.027*	$s^{-1}\cdot Pa^{-1}$	Kinetic constant for carbon desorption
k_g	3.2*	$nm^3\cdot s^{-1}$	Kinetic constant for CNT growth
P_C	1*	Pa	Effective pressure of carbon source (normalized)
P_E	1*	Pa	Effective pressure of etching agent (normalized)
$\Delta\mu_{cnt\text{-}gr}$	0.07	eV	Chemical potential difference between CNT and graphene
μ_{cnt}		eV	Chemical potential of CNT (equal to that on catalyst surface at equilibrium)
$\Delta\mu$		eV	Chemical potential difference between graphene and carbon on catalyst surface
Ω	2.62	Å^2	Atomic area per carbon atom
σ	0.1954	eV Å	Edge energy of graphene (adjusted to roughly reproduce experimental trend)
ν_0	1×10^{13}	s^{-1}	Prefactor for Arrhenius expression

Supplementary Note 7: Temperature-dependent Monte Carlo simulations

To verify the model for our kinetic Monte Carlo simulations, we run simple simulations at constant temperatures. Most of the simulation parameters are adopted from the literature[3], which investigated the Ostwald ripening of Co nanoparticles on graphite surfaces, while a few parameters are adjusted to achieve better consistency with the AFM observations shown in Fig. 4c,d and the ensemble growth rate shown in Fig. 4. All parameters used in this study are summarized in Supplementary Table 5.

Each simulation initially contains 100 particles randomly distributed within a 140×140 nm unit cell under periodic boundary conditions, and each condition is simulated multiple times to obtain statistical averages. Supplementary Fig. 8a shows time evolution of average particle radius $\bar{R}$ and the total number of all remaining nanoparticles for four different temperatures. As described by Eq. (2) in the main text, Ostwald ripening is expected to follow power-law growth behavior once the system reaches a sufficiently dilute regime. For Ostwald ripening of three-dimensional nanoparticles on surfaces, a $t^{1/4}$ growth law is widely known to hold, but it has also been reported that, in earlier stages, before particles become sufficiently sparse, the effective exponent can be smaller than 1/4. To assess the validity of our simulation model under the present conditions, where particles grow from a nominal thickness of 0.1 nm to average diameters of approximately 3–6 nm, we examined the generalized ripening relation $\bar{R}^n - \bar{R}_0^n = Kt$. Supplementary Fig. 8b plots $\bar{R}^n - \bar{R}_0^n$ as a function of time, where a value of n = 5.5 yields an approximately linear relationship over the relevant size range. This exponent is in good agreement with previous reports for the early-stage ripening regime[3]. Furthermore, the temperature dependence of the proportionality constant K follows Arrhenius behavior, with an extracted activation energy E_r of approximately 2.6 eV. We note that the value of E_r = 2.6 eV was first determined from the temperature dependence and hysteresis of the experimentally measured CNT growth rates based on comparison with the deterministic ensemble model for Ostwald ripening (Fig. 4h). Since the previous study[3] considers a different system (Co on graphite) from our experiments, we then slightly adjusted a few parameters in Supplementary Table 5, so that the independently performed KMC simulations yield a consistent E_r through the analysis shown in the inset of Supplementary Fig. 8b.

Using the same KMC model, we then simulated the size evolution of individual nanoparticles under time-varying temperature conditions, analogous to those employed in the experiments. Each condition was simulated 36 times with 100 particles in each run, corresponding to 3600 initial particles in total. Ensemble behaviors, such as $\bar{R}$, $\bar{R}^n - \bar{R}_0^n$, and the total number of nanoparticles are summarized in Supplementary Figs. 8c,d. In addition, the trajectories for individual nanoparticles are shown in Supplementary Fig. 8e. Because CNTs were grown from relatively large catalyst particles (Supplementary Fig. 9), in the simulation for length evolution of CNTs, we simply assumed that the 185 largest nanoparticles among the 388 particles that survived until t = 900 s contributed to CNT growth. The remaining particles were excluded, as they predominantly disappear during the ripening process. CNT growth was then simulated using a simplified scheme in which growth initiates and terminates at random times for each selected particle. Note that this latter assumption is not strictly accurate, because, as discussed in Fig. 3c, the probability of growth termination depends on temperature and its prior history. The nanoparticle size evolution is shown by black lines in Supplementary Fig. 8e, while the time intervals assigned as active CNT growth are indicated by colored lines and symbols. The corresponding temporal evolution of CNT lengths obtained from this model is shown in Supplementary Fig. 8f.

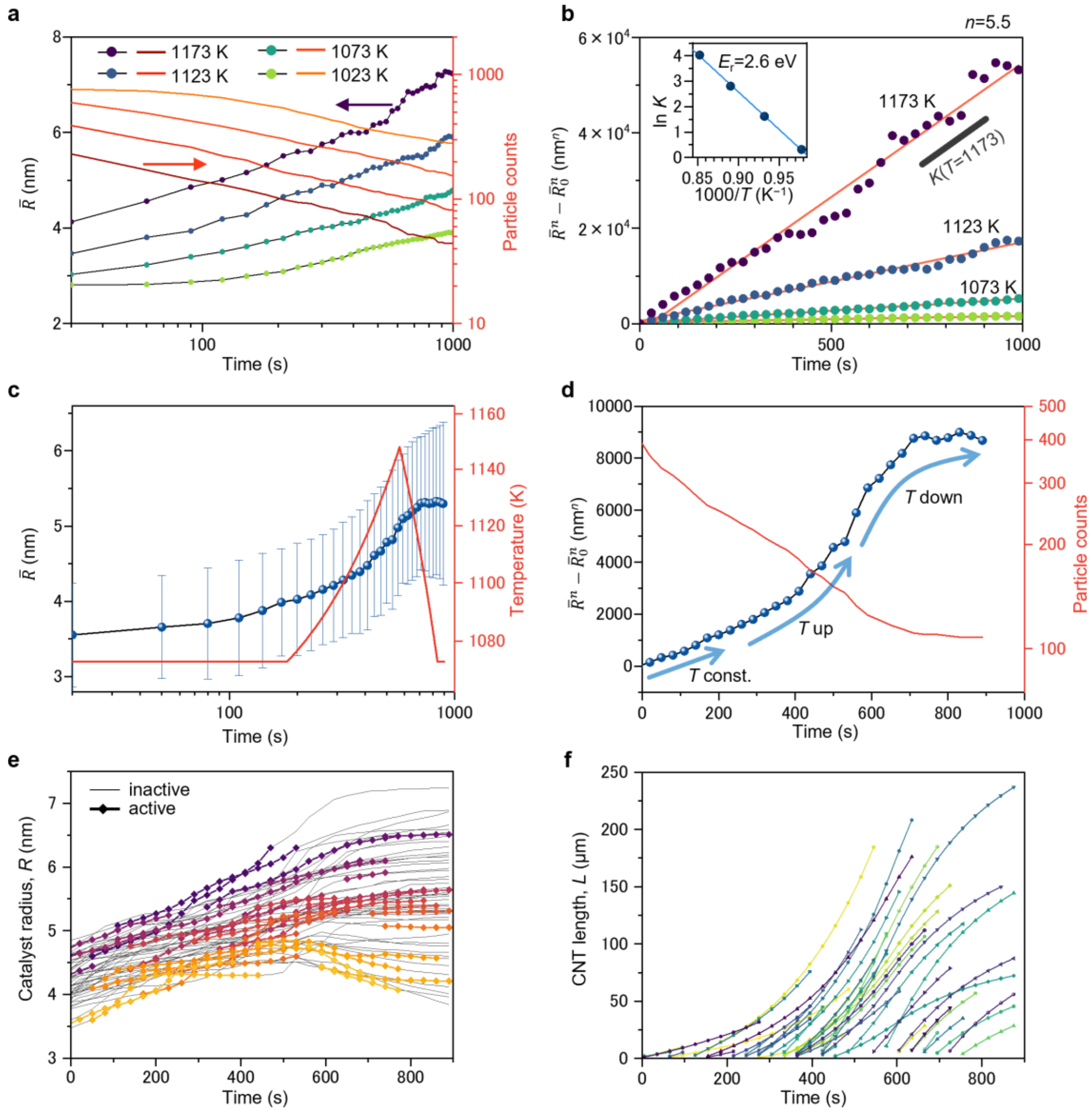

Supplementary Fig. 8 | KMC validation and connection to simulating CNT length evolution. **a** Time evolution of average radius $\bar{R}$ and the total number of nanoparticles obtained from the KMC simulation at different temperatures. **b** Time evolution of $\bar{R}^n - \bar{R}_0^n$, where $n = 5.5$ provides an approximately linear relation over a given size range. Inset: Arrhenius plot of the prefactor K in the Ostwald ripening model, yielding an activation energy of 2.6 eV. **c** Time evolution of $\bar{R}$ during temperature modulation (right-hand axis). Error bars represent the standard deviation of individual particle size. **d** Time evolution of $\bar{R}^n - \bar{R}_0^n$ and the total number of nanoparticles. **e**,**f** Time evolution of individual nanoparticles that we assume to grow CNTs in Fig. 4j (black lines) (**e**) and of lengths of individual CNTs (**f**). The periods assigned as active CNT growth in panel **e** are indicated with colored lines and symbols.

Supplementary Table 5 | Parameters used in the KMC simulations and their physical meaning.

**Symbol / Parameter**	**Value**	**Unit**	**Physical meaning**
L_x, L_y	140×140	nm^2	Simulation cell size in x and y (periodic)
n_{int}	100	–	Initial number of islands
nominal thickness	0.1	nm	Equivalent mean film thickness
θ	π/2	rad	Contact angle of spherical-cap islands
Ω	0.25^3	nm^3	Atomic volume
γ_s	2.5	J m^{-2}	Surface free energy of the island
ν_e	1×10^{14}	s^{-1}	Attempt frequency for atom detachment
a_s	0.246	nm	Substrate lattice spacing
a_p	0.25	nm	Atomic spacing at island perimeter
E_c	−4.386	eV	Cohesive energy of island material
E_{ad}	−1.6	eV	Adsorption energy of adatom on substrate
E_d	0.1	eV	Diffusion barrier on substrate
d_{capt}	0.246	nm	Capture radius for adatom attachment

Supplementary Note 8: CNT-catalyst size correlation from post-growth AFM

Supplementary Fig. 9a,b shows AFM images of sparsely deposited catalyst nanoparticles and CNTs grown on quartz at 800 °C, together with the corresponding height correlation. The analysis was performed by identifying CNTs and their associated catalyst particles within the same field of view. As shown in Supplementary Fig. 9c, the catalyst-CNT height ratio exhibits a broad distribution and does not converge to commonly presupposed values such as 1:1. In many cases, catalyst particles are several times larger than the corresponding CNT diameters. Note that AFM characterization does not necessarily reflect the actual diameter of CNTs, resulting in physically unlikely height profiles for some nanotubes. Because this analysis is based on post-growth snapshots, the measured catalyst sizes reflect their state after growth. Under such conditions, catalyst particles may evolve during nanotube elongation, including coarsening and disappearance, as reported in previous studies[19,20]. In contrast, when catalyst size evolution is suppressed, a more defined diameter ratio (1:1.6) has been reported based on ensemble-averaged quantities[21]. However, analyses based on individual tube-catalyst pairing generally show substantial variability in the size ratio[19]. These observations indicate that the post-growth catalyst-CNT diameter ratio does not converge to a single characteristic value in general, but can be strongly influenced by growth history after nucleation, including catalyst size evolution during elongation.

Furthermore, to directly correlate a CNT growth profile with its corresponding catalyst nanoparticle, we analyzed isotope-labeled CNTs on quartz substrates. Because catalysts cannot usually be transferred via PMMA thin films, any transfer or lithography processes were avoided to maintain the as-grown structures of catalyst nanoparticles. Using the labeling protocol shown in Supplementary Fig. 9d, we measured Raman spectra along a representative metallic CNT and reconstructed its growth history (Supplementary Fig. 9e,f), from which an apparent activation energy of ≈2.3 eV was obtained. We then traced the same CNT to its catalyst nanoparticle by SEM and AFM measurements (Supplementary Fig. 9g), yielding CNT and catalyst diameters of 1.6 nm and 3.4 nm, respectively, consistent with a perpendicular-growth-like geometry shown in panel b. However, this post-growth static pairing still does not reveal how the catalyst size evolved during elongation, underscoring the importance of growth-history encoding using isotope

labeling. We also note that the iron loading was substantially reduced in this experiment to allow unambiguous CNT-particle pairing, which may affect the extent of Ostwald ripening compared with the main-text experiments.

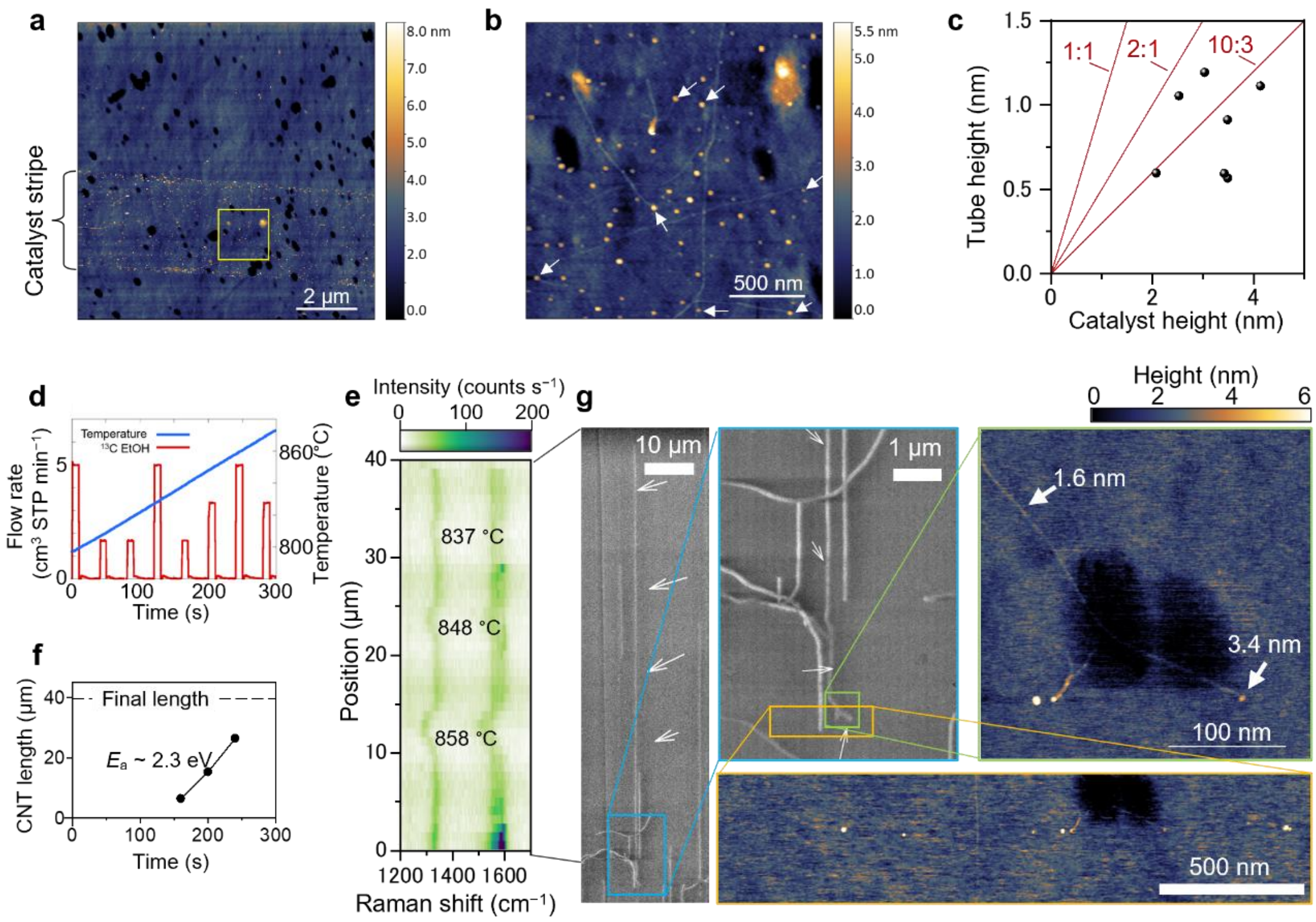

Supplementary Fig. 9 | AFM characterization on quartz. a, b AFM images of catalyst nanoparticles and CNTs grown at 800 °C. Panel b was measured within the catalyst stripe (yellow box) in a. Scale bars are 2 μm and 500 nm in panels a and b, respectively. **c** Correlation of the heights of catalyst nanoparticles and the CNTs that grew from the catalysts. Solid lines represent the 1:1, 2:1, and 10:3 catalyst-tube height ratios. **d** Temperature and isotope labeling pattern for the growth analysis on a quartz substrate. **e** Raman spectra along a metallic CNT. Temperatures at which each isotope label were incorporated are shown. **f** Reconstructed length evolution of the CNT, indicating the extracted activation energy to be ≈2.3 eV for this particular case. **g** SEM and AFM images to trace the catalyst nanoparticle responsible for the growth of the m-CNT.

Supplementary Note 9: 1D Raman spectra along CNTs

According to the definition by Gao et al.[22], where a generation is defined as the height of a single atomic layer growth (≈0.12–0.14 nm), it is estimated that the CNTs maintain their chirality for an average of three million generations, even under fluctuating environmental conditions such as variations in catalyst size and temperature. This chirality preservation along individual nanotubes was investigated by Raman spectroscopy measured along the same nanotubes in 600 nm intervals using an excitation wavelength of 532 nm, covering >15,000 points in total (9.39 mm). Supplementary Fig. 10 presents representative Raman maps for 24 CNTs, which are classified into three categories based on the confidence level of chirality assignment. The first group consists of CNTs that show clear chirality preservation over their entire lengths, as evidenced by the continuity of RBM features (panel a). The second group exhibits chirality preservation with lower confidence, where continuity is inferred mainly from the G- and D-mode features in the absence of clearly resolved RBMs (panel b). The third group includes CNTs that display apparent intratube chirality changes, manifested as discontinuities in RBM frequencies or concomitant changes in the G- and D-mode spectra (panel c).

For the CNT that exhibits a chirality change (tube #7), we examined the timing of the transition, the associated diameter change, and its impact on the growth rate (Supplementary Fig. 11a). The chirality change occurs near the maximum temperature and is most plausibly assigned to a transition from (15,2) to (15,5). Immediately after the chirality change, the growth rate decreases markedly; nevertheless, within each segment before and after the transition, the temperature dependence of the growth rate remains consistent with the trends discussed throughout the main text (Supplementary Fig. 11b). Supplementary Fig. 11c summarizes the chirality-change timing for ten CNTs together with the apparent activation energy extracted from the growth segment preceding the transition, which is related to the degree of catalyst coarsening. Although the number of samples is limited, chirality-change events appear to occur more frequently near the maximum temperature of the modulation profile. No clear correlation between the apparent activation energy and the transition timing is observed within the present dataset.

Supplementary Fig. 12 shows the SEM images of CNTs transferred on a Si/SiO_2 substrate, together with Raman mapping images of CNTs used to evaluate the degree of

chirality preservation. Some segments that are absent in Raman maps are also missing in the corresponding SEM images, indicating that they originate from physical cutting during the transfer process rather than from loss of resonance. Except for a few local mismatches (marked by *), the SEM and Raman images show consistent one-to-one correspondence, suggesting that undetected chirality changes due to resonance mismatch are not a dominant source of error in the analysis. A recent study observed chirality changes from (*n*,*m*) to (*n*+*i*,*m*−*i*) most frequently within s-CNTs[23], where minimal diameter changes and moderate changes in transition energies are expected for different 2*n*+*m* families. This suggests that the probability of fully changing resonant conditions upon intramolecular junctions is lower than that for randomly selected chiral index pairs, which may explain the reasonable consistency of SEM and Raman images. In addition, the majority (12 cases) among 19 observed cases of chirality change events show RBM spectra either before or after the chirality changes, likely supporting such a scenario.

Supplementary Fig. 12b highlights the CNT (tube #7 in Fig. 5a, tube #s7 in Supplementary Fig. 10c), which exhibits a rare chirality change event. To exclude the possibility that the observed spectral change arises from bundling of another CNT near the catalyst stripe, we compared RBM spectra before and after the transition point indicated by the blue dashed line. The upper segment exhibits a weak RBM peak at 191.7 cm^{-1}, whereas the lower segment shows a distinct peak at 177.7 cm^{-1}. Importantly, deviation of the measured data (gray circles) from Lorentzian fits (black line) is much smaller than the signal of the upper segment (red diamonds). This indicates that these two discrete RBM peaks originate from the same CNT, but with different chirality (*n*,*m*), rather than from two different CNTs. This confirms that the observed transition corresponds to an intrinsic chirality change within a single CNT.

Note that we synthesized low-density CNTs to minimize the probability of detecting multiple CNTs within a single laser spot. As a result, the density of CNTs resonant with a given excitation wavelength was very low over most regions of the substrate (Supplementary Fig. 12). Raman measurements were performed using a custom-built Raman system equipped with a 532-nm laser for low-density regions. This measurement system enabled efficient automated mapping over a series of selected areas, which was created through initial rough scans over a large area. The excitation

wavelength was therefore fixed at 532 nm for decoding isotope labels and reconstructing the growth process of individual CNTs.

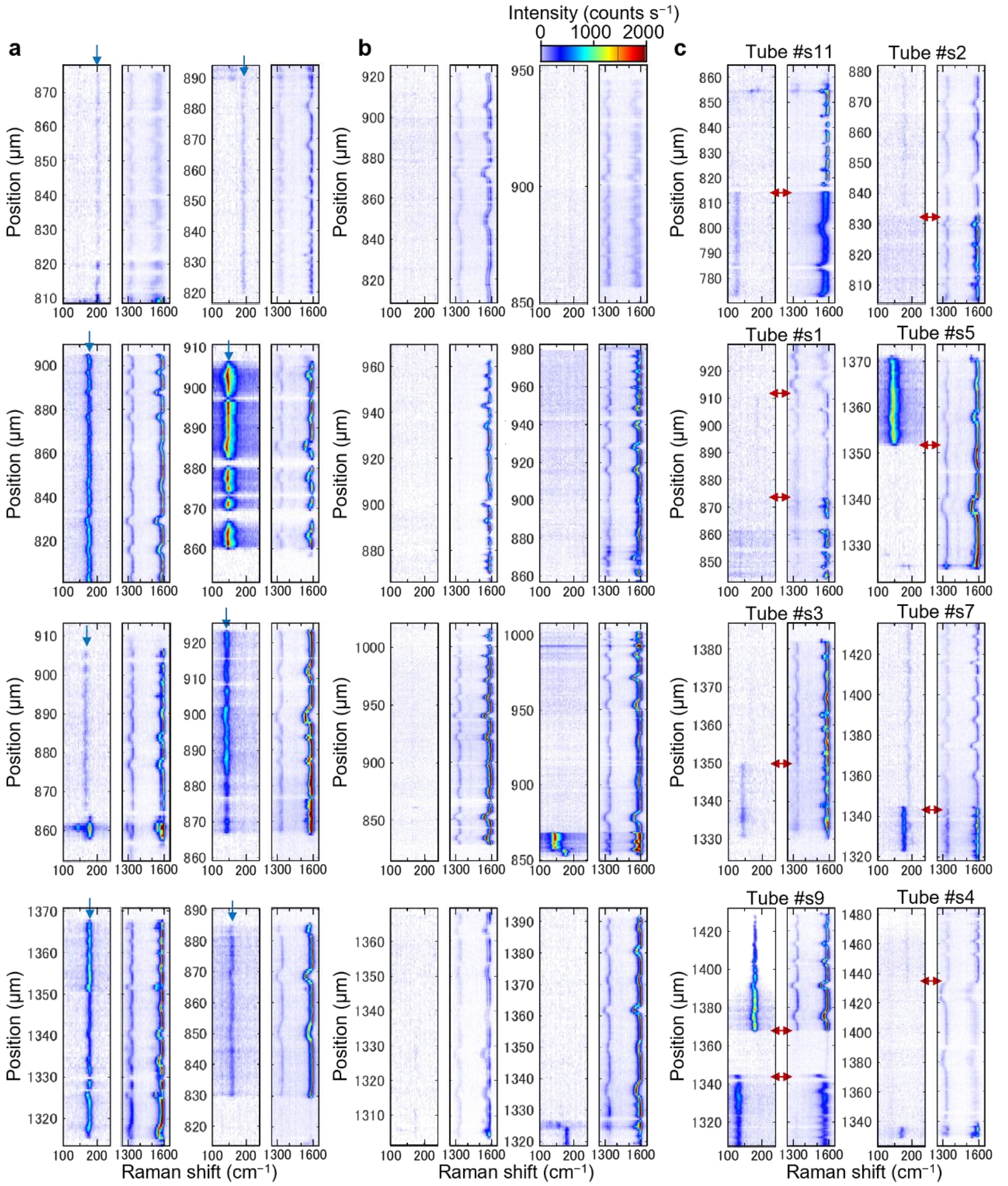

Supplementary Fig. 10 | Raman-mapping criteria for chirality preservation. Raman spectra measured along individual CNTs at 600 nm intervals using an excitation wavelength of 532 nm. **a** CNTs exhibiting clear chirality preservation over their entire lengths, confirmed by the continuity of RBM features. **b** CNTs showing chirality preservation inferred with lower confidence, based primarily on the continuity of G- and

D-mode features. **c** CNTs exhibiting apparent intratube chirality changes, as evidenced by discontinuities in RBM frequencies, accompanied in some cases by changes in the G- and D-mode spectra. In all panels, Raman signals originating from the substrate (regions without CNTs) have been subtracted.

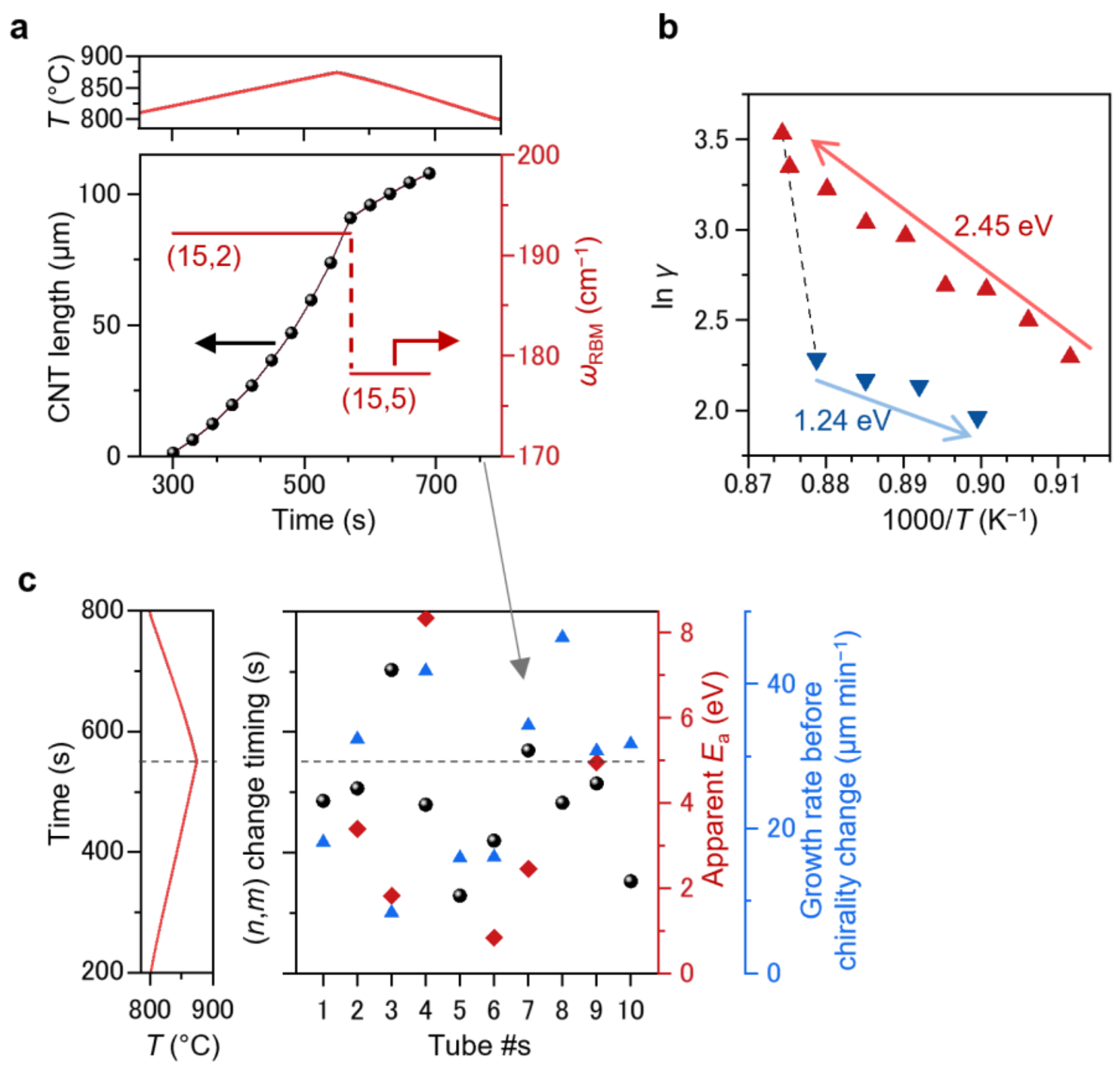

Supplementary Fig. 11 | Timing and kinetics of rare chirality-change events. a Length evolution of the CNT (tube #7 in Fig. 5a) that shows a chirality change (bottom). Temperature profile is shown on top for comparison. **b** Arrhenius plots for this CNT, showing the different slopes and a large offset of growth rate γ. **c** Chirality-change timing (black), apparent activation energy (red), and instantaneous growth rate (blue) before the transition for ten CNTs.

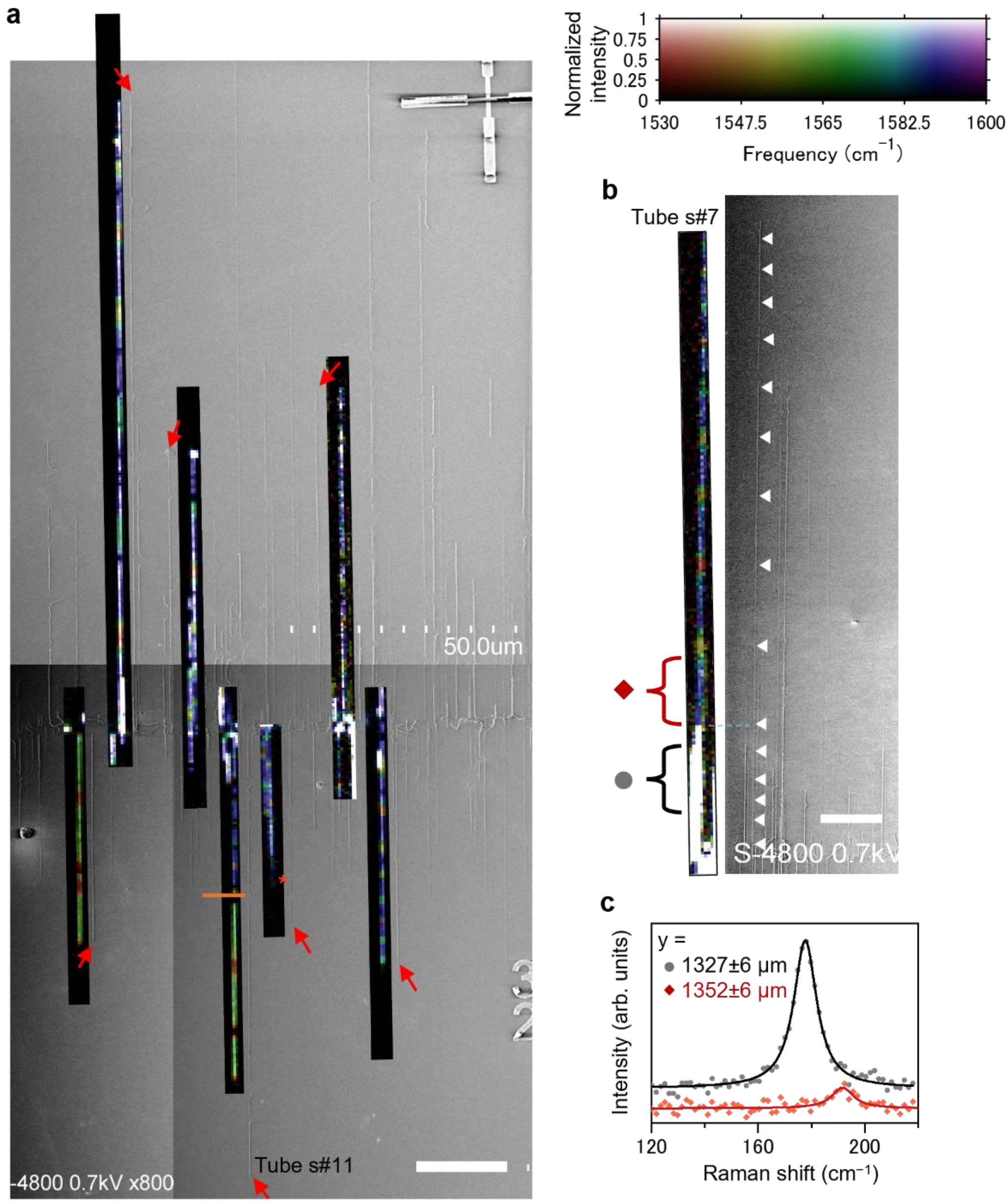

*Continues to next page

d

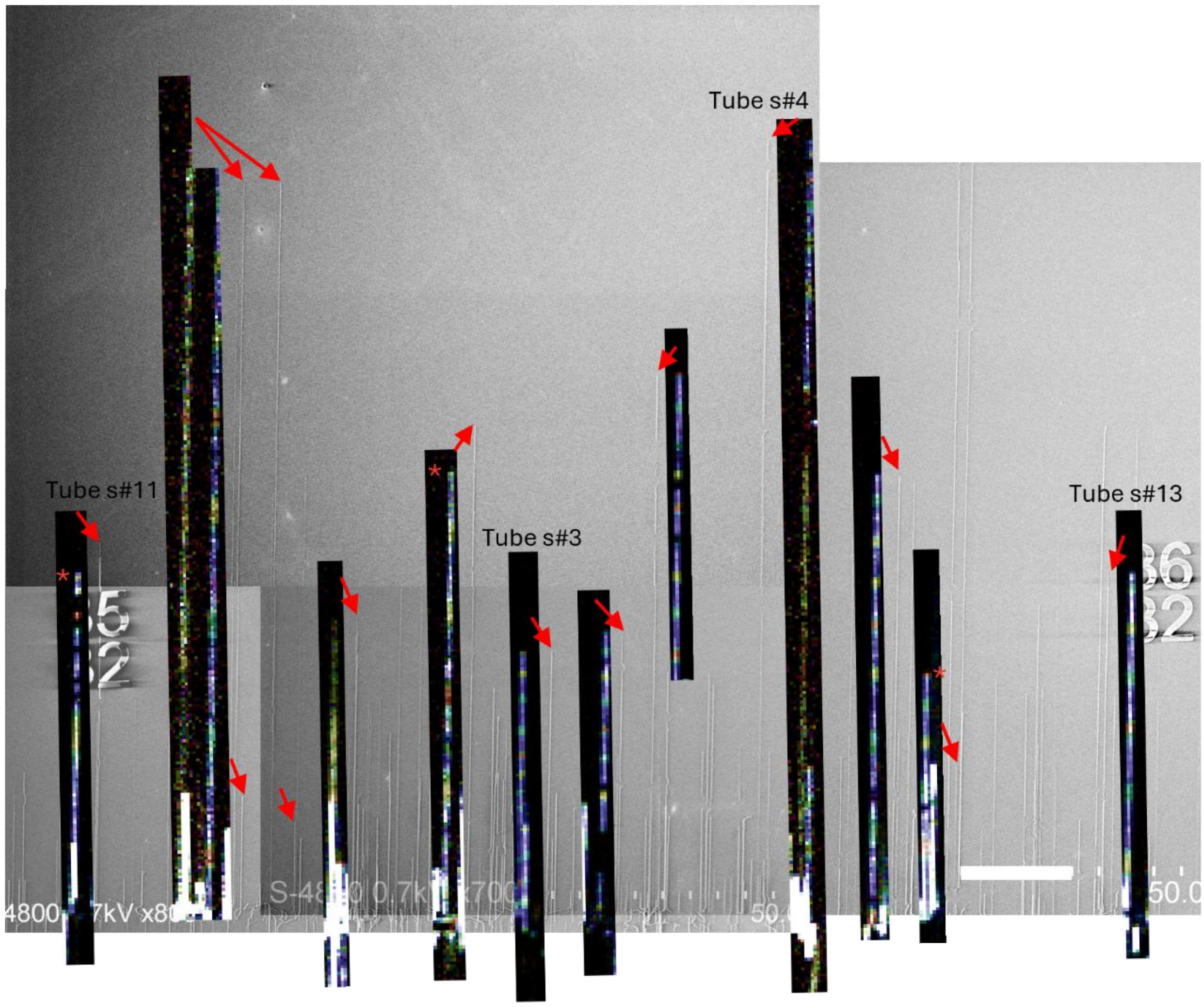

Supplementary Fig. 12 | Comparison of Raman mapping and SEM images. a SEM images of CNT transferred onto a Si/SiO_2 substrate for Raman measurements. After roughly conducting Raman mapping measurements over a very large area, CNTs indicated by red arrows (resonant to a 532 nm laser) were characterized by Raman spectroscopy, and the corresponding Raman mapping images are shown adjacent to the SEM image. Horizontal orange lines indicate the chirality change positions. Stars (*) indicate the inconsistency between SEM and Raman images, which suggests some changes in resonance condition. **b** SEM and Raman mapping images of tube #7 in Fig. 5a (tube #s7 in Supplementary Fig. 10). White triangles indicate the isotope labels, and a horizontal dashed line in light blue indicates the junction where the Raman features abruptly change. Raman mapping image shares the same scale bars with the SEM image. **c** RBM spectra of the CNT before (y = 1352±6 μm in Supplementary Fig. 10) and after (y = 1327±6 μm) the chirality change event. **d** SEM and Raman mapping images of CNTs in other regions. Scale bars are 20, 10, and 20 μm in panels **a**, **b**, and **d**, respectively.

Supplementary Note 10: Floating-catalyst chemical vapor deposition

For FCCVD-based CNT growth, the synthesis temperature was initially set to T_{set} =950 °C, held for 48 min at each step, and subsequently decreased in 50 °C steps down to 800 °C. Raman spectroscopy was performed directly on the CNTs deposited on the glass filter paper without further processing. Supplementary Fig. 13b,c shows the G-mode (excitation wavelength of 532 nm) and RBM (excitation wavelengths of 785 nm, 633 nm, and 532 nm), respectively. In our experiments, the concentration of sulfur (thiophene) as growth promoter in the reactor is 0.4–0.5 ppm. In addition, we also synthesized CNTs without thiophene promoter at four different furnace set temperatures (Supplementary Fig. 13d,e). Although diameter distribution is different from the case with thiophene, which might be influenced by the total pressure and the condition of quartz tube wall, high-quality CNTs with G/D ratio of ≈50 were obtained, exhibiting a weak temperature dependence of average diameter (1.1 nm and 1.2 nm for T_{set}=800 °C and 950 °C cases, respectively).

In our reactor, a bulk residence time is on the order of 7–8 s , which is comparable to the lower end of the direct-spun FCCVD literature summarized by Weller et al. (5–240 s, median ≈20 s)[24] and to the residence-time window examined by Novikov et al. (3.8–12.5 s)[9]. Although the overall catalyst-size behavior differs among these systems—for example, Hoecker et al.[25] observed a maximum in the particle size distribution due to evaporation at the hottest zone of the reactor—the local behavior near the reactor entrance region is more relevant here. In both their system and ours, there should be a stage in which catalyst particles grow in size while CNT growth is initiated, as illustrated in Fig. 1. More generally, although the temperature range and residence time can vary significantly among different systems, it is natural to expect non-negligible catalyst evolution in size during CNT growth lifetime, because kinetics of catalyst growth and CNT elongation should be correlated, both depending on temperature and precursor concentration.

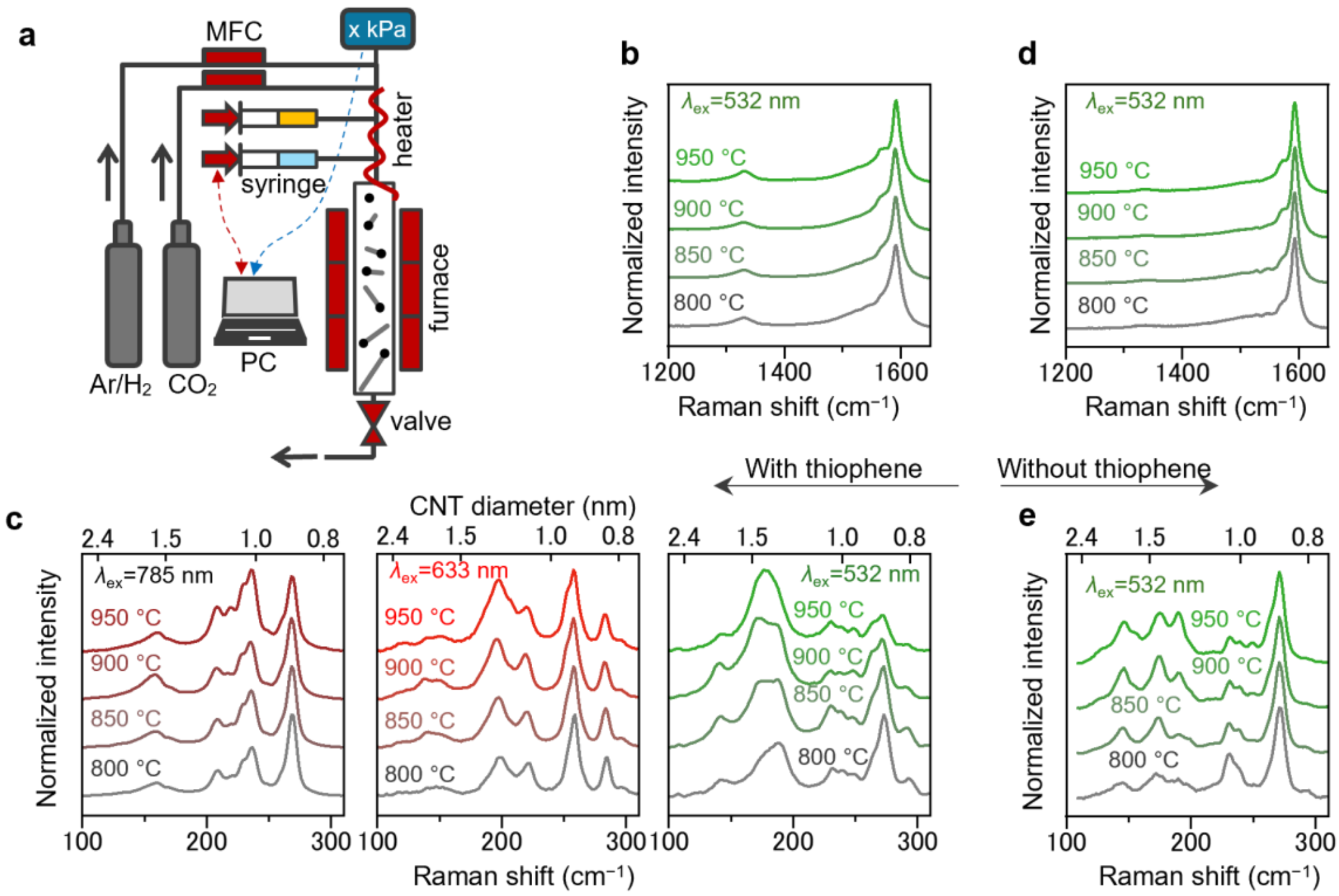

Supplementary Fig. 13 | FCCVD furnace and Raman analysis. a Schematic illustration of the FCCVD reactor used in this study. **b** Raman spectra of the G- and D-mode regions measured with an excitation wavelength of 532 nm. **c** RBM spectra measured with an excitation wavelength of 785 nm (left), 633 nm (center), and 532 nm (right) for CNTs synthesized at temperatures between 800 and 950 °C. **d**,**e** Raman spectra of CNTs grown without thiophene using an excitation wavelength of 532 nm.

Supplementary Note 11: Raman D mode and lattice defects

Non-negligible D-mode peaks are displayed in Figs. 2d and e. If the D mode originates from topological defects, i.e., introduction of pentagon-heptagon in the tube wall or vacancy, the chirality of CNTs may not be robustly preserved. Since our CNTs rarely changed their chirality during growth, we assume the D mode to be related to other disorders, such as sp^3 defects[26], surface contaminations[27], and interaction with substrates[28]. Among the abovementioned origins for Raman D mode, sp^3 functional groups and surface contamination, such as PMMA residue, are not associated with chirality change during growth and can be removed by post-growth annealing at temperatures much below that for growth (≈800 °C).

To clarify the origin of the Raman D mode observed in our samples, we performed in situ Raman spectroscopy on the same individual CNT upon heating in vacuum (Supplementary Fig. 14). The CNTs were synthesized at a fixed temperature of 800 °C, while all other growth and transfer conditions were identical to those used for the samples shown in Fig. 2. As the temperature increased, a noticeable improvement in the G/D intensity ratio was observed between 200 and 400 °C, suggesting the removal of surface functional groups[29] and residual contaminants introduced during the transfer and processing steps. After heating up to 800 °C and cooling to room temperature, a suppressed D-mode signal remained detectable, supporting the removal of functional groups or polymer residues. The small residual D mode can be attributed to substrate-induced symmetry breaking (as discussed in Supplementary Note 12) and/or dielectric inhomogeneity, rather than to irreversible lattice defects such as pentagon-heptagon pairs or vacancies. Combined with the fact that chirality changes were rarely observed during CNT growth, these results support the interpretation that our synthesis conditions suppressed the formation of topological defects capable of inducing chirality change. It should be noted, however, that this conclusion does not necessarily imply universal chirality preservation across all CNT synthesis conditions. In particular, CNTs grown using precursors, catalysts, or support prone to defect formation, as well as multi-walled CNT systems, may exhibit different defect landscapes and chirality stability[30,31]. Care should therefore be taken when extending the present conclusions to such systems.

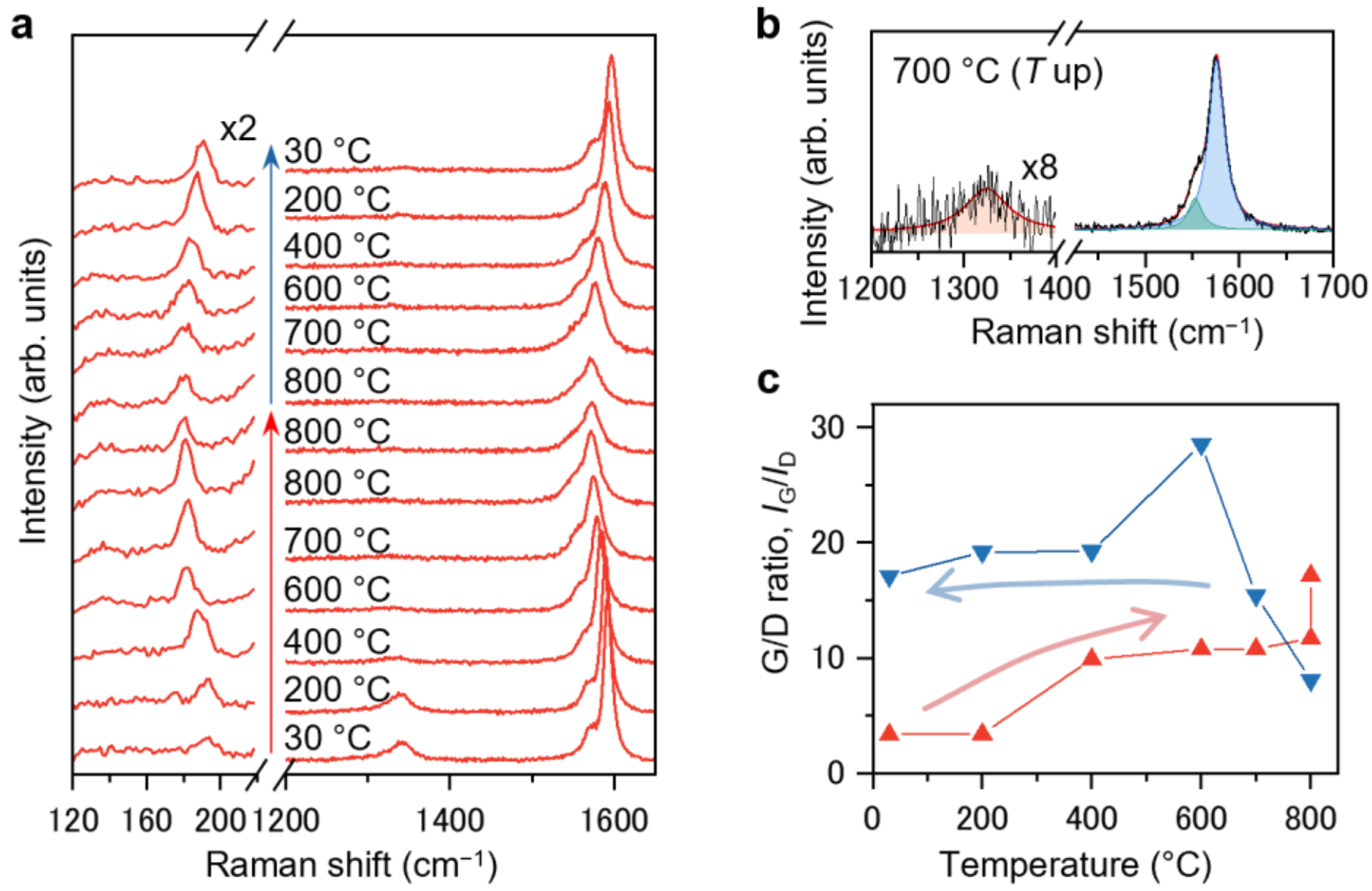

Supplementary Fig. 14 | D-mode peaks upon heating. **a** A series of Raman spectra of a single CNT measured in situ during heating and cooling in vacuum. Aligned CNTs were grown on a quartz substrate and transferred onto Si/SiO_2 substrate using a PMMA film. Excitation wavelength of 532 nm was used. **b** Raman spectra measured at 700 °C during *T* ramp-up and Lorentzian components corresponding to D, G^+, and G^- modes. Peak area of each mode is obtained as the product of peak height and peak width. **c** Temperature-dependent G/D intensity ratios (I_G/I_D) based on the fitting procedure shown in panel **b**.

Supplementary Note 12: Effects of substrates for topological defect formation

Single-crystal quartz, which was used as substrates for growth, is known to exhibit strong interactions with CNTs[32,33], sufficient to induce alignment[34]. One may therefore question whether the apparent suppression of chirality change observed in substrate-supported growth can be generalized to floating-catalyst systems. However, this situation is fundamentally different from CNT alignment on hexagonal substrates such as hexagonal boron nitride (hBN) or graphite, where crystallographic matching between the nanotube lattice and the substrate can produce chiral-angle-dependent alignment[35]. In contrast, alignment on quartz has been shown to be reproduced even when the CNT is modeled simply as a continuous cylinder with different diameters, indicating that the preferred growth direction is governed primarily by anisotropic substrate interactions rather than by hexagon-by-hexagon epitaxial matching[36]. Instead, we consider the other aspects of nanotube-substrate interactions for structural robustness in CNT growth.

To evaluate the influence of substrate-nanotube interactions on the structural stability of CNTs, we constructed a continuum model that couples elastic deformation with curvature-dependent defect energetics. Following the framework of Hertel et al.[37], the total energy of a nanotube interacting with a substrate is expressed as the sum of elastic bending energy and substrate interaction energy, $E_{\mathrm{tot}} = E_{\mathrm{bend}} + E_{\mathrm{sub}}$. The bending energy is written as

$$E_{\mathrm{bend}} = \frac{D_{\mathrm{b}}}{2} \oint (\kappa(s) - \kappa_0)^2 \, ds, \qquad \cdots (10)$$

where D_{b} is the bending rigidity, $\kappa(s)$ is the local curvature along the tube circumference, and $\kappa_0 = 2/D$ is the curvature of the undeformed circular tube with diameter D. The substrate interaction is represented by an effective adhesion energy,

$$E_{\mathrm{sub}} = \oint V(h(s)) \, ds, \qquad \cdots (11)$$

where $h(s)$ denotes the distance to the substrate and V is an effective interaction potential. To describe the deformation in a tractable manner, the tube cross-section is approximated by a perimeter-preserving ellipse, $x = a\cos\phi$, $y = b\sin\phi$ with

$$a = r_0(1 + \delta), b = r_0(1 - \delta), \qquad \cdots (12)$$

where δ is a dimensionless ellipticity parameter and r_0 is a reference radius determined such that the perimeter remains equal to that of the original circle ($L = \pi D$). For each tube diameter D, the equilibrium ellipticity $\delta(D)$ is obtained by minimizing E_{tot} as shown in Supplementary Fig. 15a. Note that the effective adhesion energy γ_{eff} between a nanotube and a substrate is determined so that the model reproduces the molecular mechanics calculations[37]. Representative cross-sectional shapes for selected diameters are shown in Supplementary Fig. 15b.

The local curvature of the deformed tube is given by

$$\kappa(s) = \frac{ab}{(a^2 \sin^2 \phi(s) + b^2 \cos^2 \phi(s))^{3/2}}. \quad \cdots (13)$$

Using this local curvature, an equivalent local diameter is defined as $D_{eq}(s) = 2/\kappa(s)$. The formation energy of a 5|7 defect is then assigned based on the diameter-dependent data reported by Yuan et al.[17]. By fitting the reported data using the following phenomenological expression constrained to the graphene limit $E_f(\infty) = 4.39$ eV,

$$E_f(D) = E_f(\infty) - \frac{\varepsilon_D}{(D + D_0)}, \quad \cdots (14)$$

we obtain ε_D =3.144 eV nm and D_0 = 0.511 nm (Supplementary Fig. 15c).

The local defect formation energy is thus evaluated as $E_f(s) = E_f\big(D_{eq}(s)\big)$. Within a simple thermal equilibrium approximation, the local defect probability is assumed to follow a Boltzmann factor,

$$p(s) \propto \exp\left(-\frac{E_f(s)}{k_B T}\right). \quad \cdots (15)$$

The effective defect density of the entire nanotube is obtained by integrating this quantity along the circumference L,

$$\langle p \rangle = \frac{1}{L} \oint \exp\left(-\frac{E_f(s)}{k_B T}\right) ds, \quad \cdots (16)$$

where a prefactor in $p(s)$ is assumed to be 1 for simplicity. For a representative diameter of D = 1.5 nm, the spatial variation of $E_f(s)$ and the resulting local Boltzmann factor $p(s)$ are shown in Supplementary Fig. 15d.

The above procedure was applied over different diameters, using the corresponding equilibrium ellipticity $\delta(D)$. The resulting defect densities are plotted in

Supplementary Fig. 15e, suggesting that the influence of substrates on topological defect formation is more significant in the case of larger-diameter CNTs. As single-crystal quartz is known to have strong interaction with CNTs, we varied the effective adhesion energy γ_{eff} by factors of 0.5 and 2 relative to the reference value (2.6 eV nm^{-2}) calibrated to the results by Hertel et al.[37]. The present analysis indicates that strong CNT-substrate interaction deformation enhances the probability for defect formation through the tube deformation. Therefore, the robustness of structural memory observed in our experiments is not a consequence of substrate stabilization, but is expected to persist even in floating-catalyst growth environments where such substrate interactions are absent. The calculations also show that increasing the interaction strength enhances the deformation and shifts the diameter at which defect density is minimized toward smaller diameters. These results may suggest that substrate interactions may impose a constraint on the accessible diameter range of horizontally aligned nanotubes grown on quartz substrates.

We should emphasize that the analysis above is based on a thermal equilibrium assumption and considers only curvature-dependent defect energetics. As a result, the estimated defect density for D =1.5 nm is lower than the present experimental observation (≈2.5 mm^{-1}). This can be attributed to kinetic processes, which dominate the defect formation for CNT growth at practical temperature range[17]. However, extending the present framework to include growth kinetics is nontrivial, as even the previous kinetic model[17] does not include curvature effects and uses a flat graphene edge as a model case.

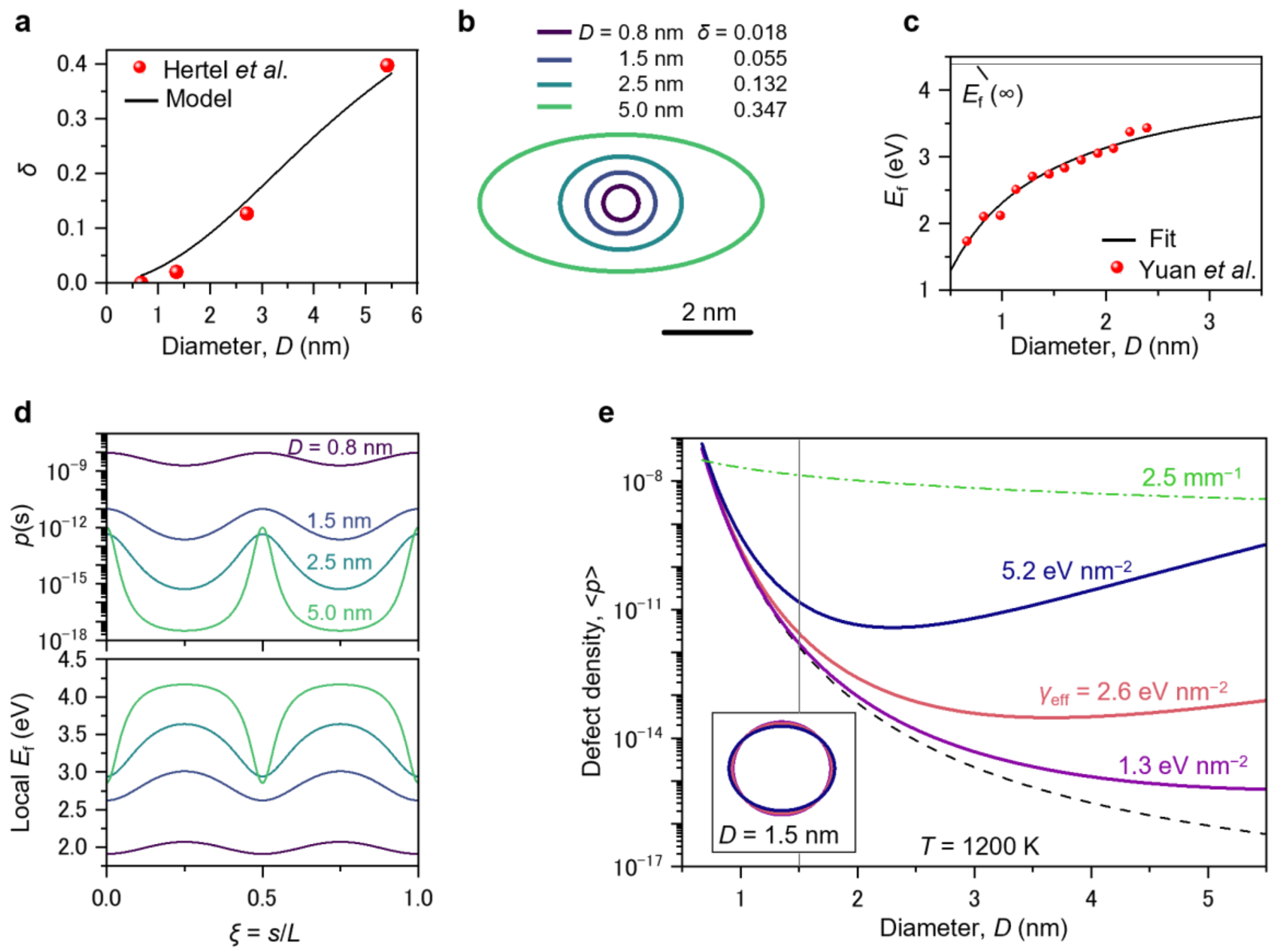

Supplementary Fig. 15 | Substrate-induced deformation and its impact on defect formation under thermal equilibrium. a Diameter dependence of the ellipticity δ obtained from the continuum model, with molecular mechanics results[37] shown for reference. **b** Cross-sectional shapes of nanotubes with different diameters. **c** Curvature-dependent defect formation energy based on the calculated data of Yuan et al.[17] and the fitted curve used in this work. **d** Spatial variation of the local defect formation energy and the corresponding Boltzmann factor along the tube circumference for selected diameters. **e** Circumferentially averaged defect density as a function of nanotube diameter at T = 1200 K for different substrate interaction strengths. The black dashed line indicates the undeformed case, the vertical line marks a representative diameter (D = 1.5 nm). The inset compares the cross-sectional shapes of a nanotube with different interaction strengths.

Supplementary Note 13: Molecular dynamics study on substrate effects

To explore kinetic effects of tube-substrate interactions on defect formation, we performed molecular dynamics simulations of CNT growth on a substrate with varying van der Waals interaction strength. Simulations were carried out using the LAMMPS package[38] with a hybrid interaction model. Fe–C interactions were described by a machine learning interatomic potential (DeePMD[39], DeepCNT-22[18]), applied to iron and carbon atoms. The substrate was modeled as a multilayer slab composed of artificial atoms (atomic mass = 20 amu) with a face-centered cubic structure, stabilized by strong Lennard–Jones (LJ) interactions to prevent evaporation. The time step for numerical integration was 2.0 fs. Interactions between the substrate and Fe/C atoms were described by weaker LJ potentials as summarized in Supplementary Table 6. The system consisted of an iron nanoparticle (Fe_{217}) attached to a (8,4) CNT segment placed on the substrate. Periodic boundary conditions were applied in all directions. The bottom layer of the substrate was fixed, while the remaining atoms were maintained at 1400 K in the canonical (NVT) ensemble using a Nosé–Hoover thermostat[40,41].

To mimic an experimental situation[5], carbon atoms were continuously supplied to the catalyst by inserting atoms at a fixed rate within the Fe particle, representing an effective carbon source pressure. In parallel, unstable carbon atoms were removed stochastically based on their local atomic potential energy. The removal probability was defined by a Boltzmann-type expression,

$$P_{\mathrm{etch}} = A_{\mathrm{etch}} \times \min[1, \exp\left(\frac{E_i - \mu}{\mathrm{k_B}T}\right)], \qquad \cdots (17)$$

where A_{etch} is a kinetic prefactor reflecting the effective pressure of etching agents, E_i is the atomic potential energy, and μ (−6.6 eV) represents the effective chemical potential of the etching environment. These insertion and stochastic removal processes were applied at regular intervals, enabling continuous competition between carbon incorporation and removal at the catalyst interface.

Supplementary Fig. 16a shows a snapshot of the system after attaching an (8,4) CNT segment to the catalyst particle and saturating the catalyst with carbon, which is defined as t = 0. To amplify the effect of substrate interactions within the accessible MD timescale, the van der Waals interaction between the CNT and the substrate was

intentionally set to a large value. As shown in Supplementary Fig. 16b, the CNT with a diameter of 0.84 nm exhibits a pronounced elliptical deformation due to the intentionally strong interaction with the substrate, where the green atoms denote the tube-catalyst interface at t = 0 ns. The magnitude of this deformation is significantly larger than that presented in Supplementary Fig. 15. As a consequence of this deformation, local curvature is strongly enhanced at specific regions of the nanotube. Correspondingly, as highlighted in red in Supplementary Fig. 16c, a pentagon–heptagon (5|7) defect pair is formed at these highly curved regions, accompanied by changes in chirality. At $t \approx 54.86$ ns, the (9,5) CNT segment was steadily elongated (Supplementary Fig. 16d). The spatial correlation between enhanced curvature and defect formation suggests that substrate-induced curvature plays a key role in lowering the kinetic barrier for defect formation, consistent with the thermodynamic tendency discussed in the equilibrium analysis. On the other hand, when a 77.5% weaker LJ potential is applied between a CNT and a substrate, the CNT maintains a circular geometry and preserves its chirality (Supplementary Fig. 16e). Note that the defect formation is a stochastic event, and we rarely observed such defect formation within a timescale of 10 ns when the same simulation was performed using different strengths of LJ interactions.

These observations indicate that substrate interactions can promote chirality changes through kinetic pathways compared to floating-catalyst systems. At the same time, our experiments show that robust chirality preservation is maintained even for CNTs grown on quartz substrates. This suggests that similarly robust structural memory also exists in floating-catalyst growth, supporting the extension of insights obtained from substrate-supported systems to FCCVD conditions. It should be noted, however, that the present simulations do not fully capture other effects such as catalyst immobilization on the substrate or specific interaction between CNT and quartz surface, and thus the quantitative relevance to experimental systems requires further consideration.

Supplementary Table 6 | Lennard-Jones parameters used to include the effects of a substrate composed of artificial atoms X.

Interaction	Fig. panel	Epsilon (eV)	Sigma (Å)
Fe-X	All	0.1	2.25
C-X	a-d	0.025	3.37
	e	0.005625	3.37
X-X	All	0.3	2.25

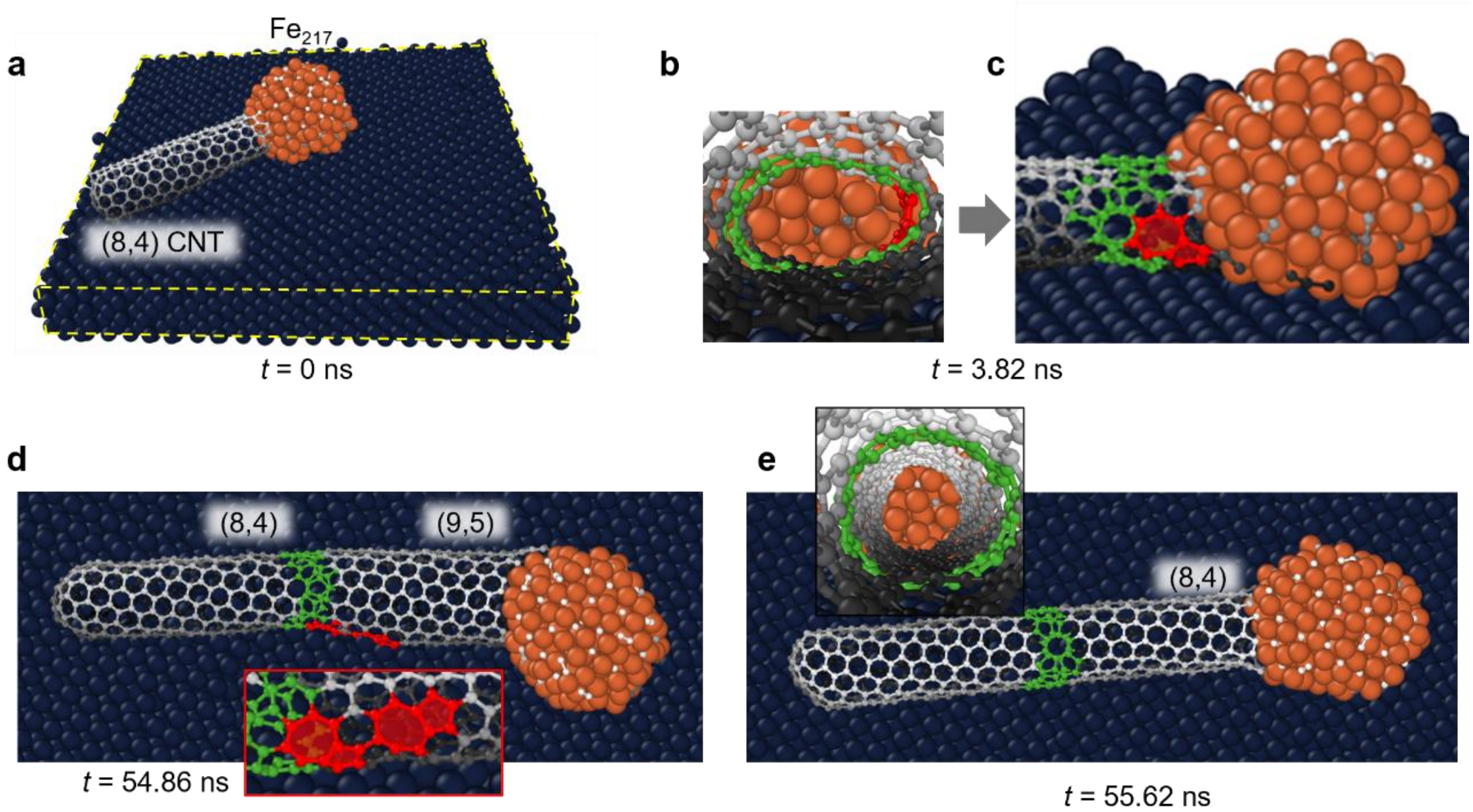

Supplementary Fig. 16 | Molecular dynamics simulations of substrate-induced deformation and defect formation. a Initial configuration at t = 0 ns after reaching quasi-equilibrium for a (8,4) CNT segment connected to an Fe catalyst particle on a substrate. **b** Elliptical deformation of the CNT under strong van der Waals interaction with the substrate; green atoms indicate the tube–catalyst interface. **c** Formation of a pentagon–heptagon defect pair (red) at regions of enhanced curvature, accompanied by a local change in chirality. **d** Subsequent elongation of the transformed (9,5) CNT segment at t = 54.86 ns. **e** CNT grown under weaker substrate interaction, maintaining a circular geometry and preserving its chirality. Simulations were performed at a temperature of T of 1400 K.

Supplementary Note 14: Kinetic defect healing at growth interfaces

To clarify why chirality changes remain rare despite frequent local bond rearrangements at the tube-catalyst interface, we analyzed representative MD trajectories at 1400 K and event statistics (Supplementary Fig. 17). A representative event is shown in Supplementary Fig. 17a,b. A pentagon (red) appears near the interface and may even become temporarily separated from the catalyst by a newly formed row of hexagons. However, rather than continuing forward growth, the system typically undergoes local reverse reactions: the newly formed hexagon row dissociates, the pentagon collapses, and the structure is reconstructed into a defect-free hexagonal network. Only then does elongation resume.

This behavior is reflected in Supplementary Fig. 17c. During the interval between t_2 and t_3, when the pentagon persists (gray region), the number of carbon atoms incorporated into the nanotube (red) remains nearly constant, indicating that net growth is effectively paused. Once the pentagon is removed, nanotube growth resumes. Thus, non-hexagonal defects act as kinetic bottlenecks rather than as structures that are readily buried into the sidewall. A schematic interpretation is shown in Supplementary Fig. 17d: the normal growth pathway proceeds through repeated hexagon incorporation, whereas a defect-bearing pathway is kinetically hindered and tends to return to the normal pathway through reverse reactions, which have lower activation barrier than forward reactions.

To support this picture, the statistical consequence of this filtering is shown in Supplementary Fig. 17e–h. All unique rings that reached layer 2 (green row) from layer 1 (blue row) were tracked to determine whether they advanced into layer 3 or instead collapsed back below layer 1. Hexagons predominantly proceeded deeper into the sidewall, whereas pentagons were preferentially eliminated. At the slowest growth condition (1.7 atoms ns^{-1}), 96.9% (31/32) of observed pentagons in layer 2 collapsed before reaching layer 3, whereas only 6.2% of hexagons did so. At higher growth rates, pentagons still showed much larger collapse probabilities than hexagons: 86.0% at 3.4 atoms ns^{-1} and 60% at 7.5 atoms ns^{-1} compared with only 7.1% and 3.0% for hexagons, respectively.

More generally, the present results point to a partly shared kinetic feature with DNA replication. In both cases, structural rearrangements proceed linearly at a localized

active front—such as an enzyme active site or the tube–catalyst interface—whereas structures that have moved sufficiently far away from that front become effectively frozen because further rearrangement should overcome a high reaction barrier. Under such conditions, if an incorrect local structure is incorporated at the active front, forward propagation becomes kinetically less favorable than its reverse process. As a result, the system acquires a bias toward removing the error before further elongation proceeds, though only biochemical processes can achieve high fidelity surpassing thermodynamic limits by dissipating energy of ATP hydrolysis[42]. Partially consistent with this picture, our experiments show that, although the growth rate adaptively changes in response to environmental modulation, chirality remains robust over the experimentally examined growth rate range.

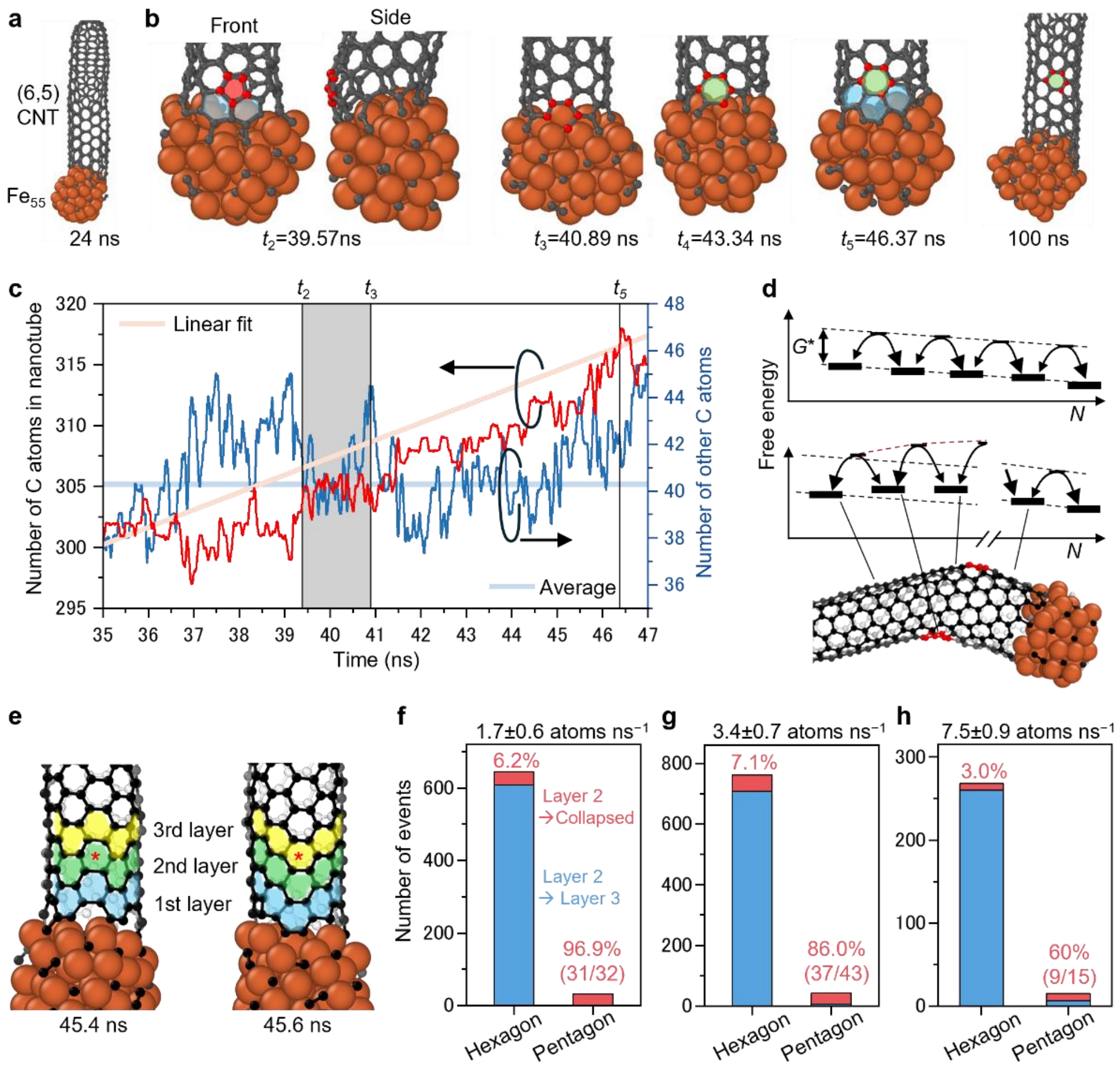

Supplementary Fig. 17 | Kinetic healing of non-hexagonal defects at the tube–catalyst interface. a Snapshot of a CNT grown from an Fe_{55} catalyst. **b** Front and side views of a representative event in which a pentagon (red) forms transiently near the tube edge and is subsequently removed through local reconstruction; additional hexagons formed during the event are highlighted in green and blue. **c** Time evolution of the number of carbon atoms incorporated into the nanotube (red) and other carbon atoms (blue), showing that net elongation is suppressed while a pentagon persists (gray background). **d** Schematic free-energy landscape illustrating normal growth (top) and defect-including pathways (middle), where forward propagation is hindered and reversal becomes favorable. **e** Definition of layer depth from the tube–catalyst interface used for ring-tracking analysis. **f–h** Statistical outcomes for rings that reached layer 2 under different carbon supply rates. To increase the number of pentagon events, simulations were restarted using the same initial configuration that contains a pentagon in layer 2, with 20, 20, and 10 repeated trajectories using different random seeds. T=1400 K.

Supplementary Note 15: Growth simulation from unmatched catalysts

To examine the robustness of CNT structure against catalyst size, we performed MD simulations starting from a deliberately mismatched configuration. A (8,7) CNT was attached to a large Fe catalyst particle. This configuration lies outside the CNT-catalyst size relationship obtained via spontaneous nucleation with the same potential (gray region in Supplementary Fig. 18a). During subsequent growth, the number of carbon atoms in CNT lattice linearly increased, while the carbon concentration within the catalyst remained approximately constant. Supplementary Movie 2 shows the MD trajectory for CNT growth from an Fe_{1389} nanoparticle, where the nanotube axis was adjusted to align parallel to the z-axis through post-growth processing. No structural adaptation of the CNT to the catalyst size is observed: the nanotube retains its initial chirality and diameter throughout elongation, even for substantially larger catalyst particles. These results indicate that, once nucleated, the CNT structure is not redefined by the instantaneous catalyst size during growth. The present behavior partially differs from a previous report[43], which may be attributed to differences in the accuracy of the interatomic potentials employed, such as energy cost for defect formation.

In terms of the carbon throughput into and out of the catalyst nanoparticle, the observed behavior is consistent with our previous kinetic analysis in alcohol CVD growth[5]. Approximately 840 carbon atoms were supplied to the catalyst during the relevant growth interval (panel b, 90 ns), of which about 600 were converted into the CNT, while the remaining carbon atoms were removed from the system after being judged unstable. Experimentally, this removal process corresponds to etching by oxidizing species such as H_2O.

To assess whether the observed structural robustness depends on the specific chirality, we performed additional growth simulations for two different chiral indices under otherwise identical conditions: a zigzag (13,0) tube and a chiral (10,5) tube (Supplementary Fig. 18e,f). In particular, zigzag CNTs have been reported to be highly sensitive to the size of catalyst particles[44]. In both chirality cases, the tubes retained their initial structures throughout the simulated growth while the catalyst enlarged from Fe_{120} to Fe_{420} (t = 0–300 ns), which are consistent with the behavior of the (8,7) tube. The

absence of chirality changes across near-armchair, chiral, and zigzag tubes indicates that the structural memory under catalyst coarsening is not restricted to particular chiral angles.

To distinguish catalyst-size perturbation from catalyst deactivation, we further performed MD simulations of CNT growth under abrupt catalyst coalescence events (Supplementary Fig. 19). As the starting growth catalyst, we used the Fe_{140}-(8,7) CNT configuration extracted at t = 20 ns from the MD simulation shown in Fig. 5e, where the nanotube was actively growing from the catalyst. This configuration was placed near another Fe–C particle, and the incoming particle was given an initial velocity toward the growth catalyst. The simulations were performed at 1400 K.

When the incoming particle retained an active Fe–C surface, coalescence did not immediately disrupt the CNT growth interface. After merging with either an $Fe_{140}C_x$ particle or a much larger $Fe_{1400}C_x$ particle, the CNT remained attached to the merged catalyst and preserved its original tubular structure. These results indicate that even abrupt catalyst enlargement through coalescence does not necessarily change nanotube chirality or lead to catalyst deactivation.

By contrast, coalescence with a carbon-encapsulated, deactivated $Fe_{50}C_x$ particle led to a qualitatively different outcome. In this case, the incoming particle transferred a pre-existing carbon shell or graphene-like nucleus to the active catalyst surface, which promoted passivation of the growth interface. Thus, catalyst coalescence can indeed lead to deactivation, but the decisive factor is not the size increase itself; rather, deactivation is triggered when a graphitic carbon nucleus is introduced onto an otherwise active catalyst surface. This behavior supports our interpretation that the formation of a new graphene-like nucleus on an active catalyst provides a direct pathway to catalyst passivation and growth termination.

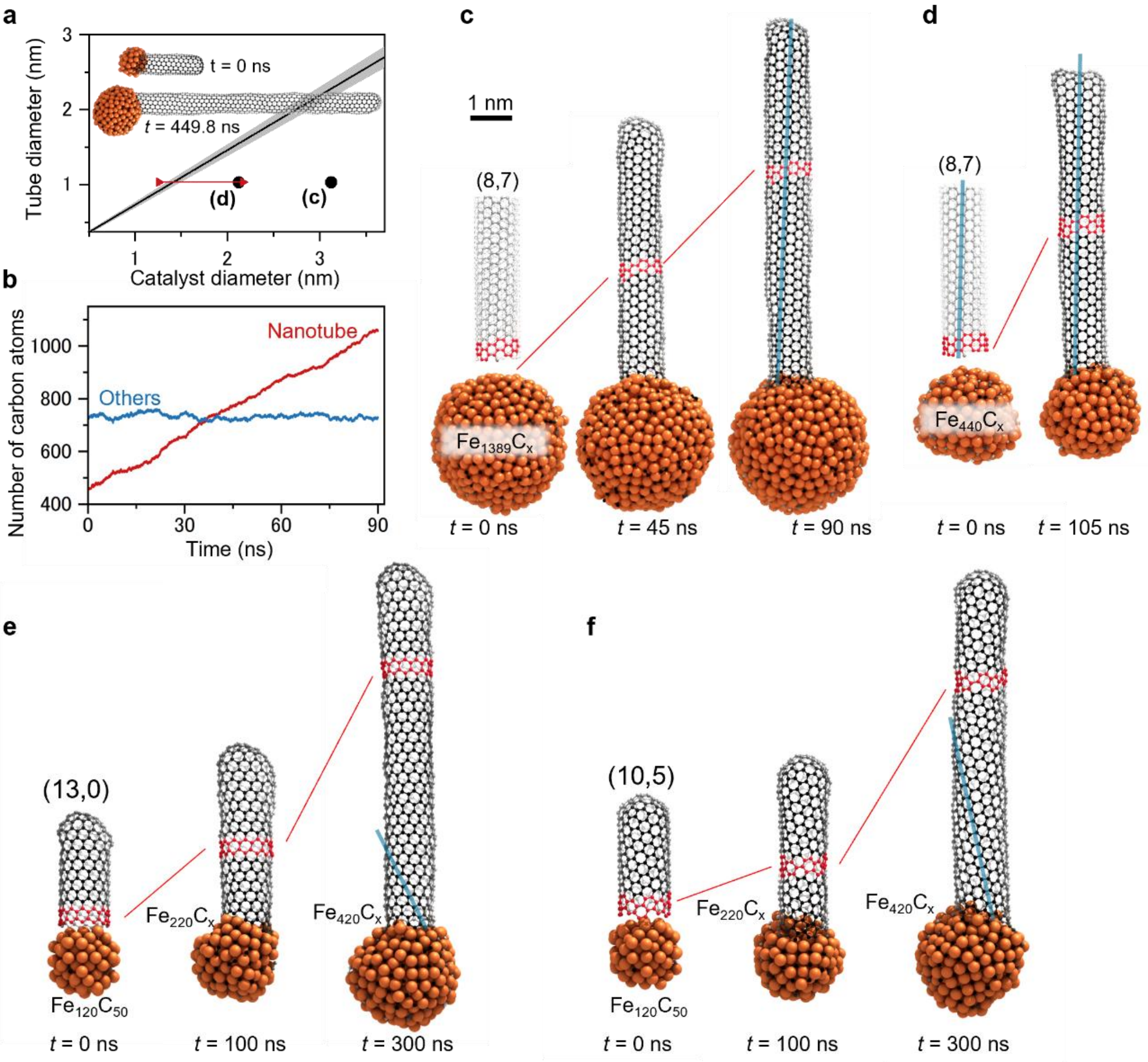

Supplementary Fig. 18 | Growth of a small-diameter CNT attached to an oversized catalyst particle. a CNT-catalyst size relationship obtained from spontaneous nucleation (black line) and that of prepared configuration. The present configuration lies outside the typical range (gray region). Red triangles show catalyst-tube size relationships for the CNT growth simulation shown in the inset, which is extended from the result shown in Fig. 5. **b** Time evolution of carbon atoms in the CNT (red) and in other forms (blue). **c,d** Snapshots of (8,7) CNTs grown from large catalysts (Fe_{1389} and Fe_{440}), showing chirality preservation despite the size mismatch between CNTs and catalysts. Temperature was kept at 1400 K. **e,f** Snapshots of a (13,0) CNT (**e**) and a (10,5) CNT (**f**) growing from an iron nanoparticle that grows in size at a rate of 1 atom ns^{-1}. The same parameters for carbon supply and removal are used as an (8,7) CNT case in Fig. 5e.

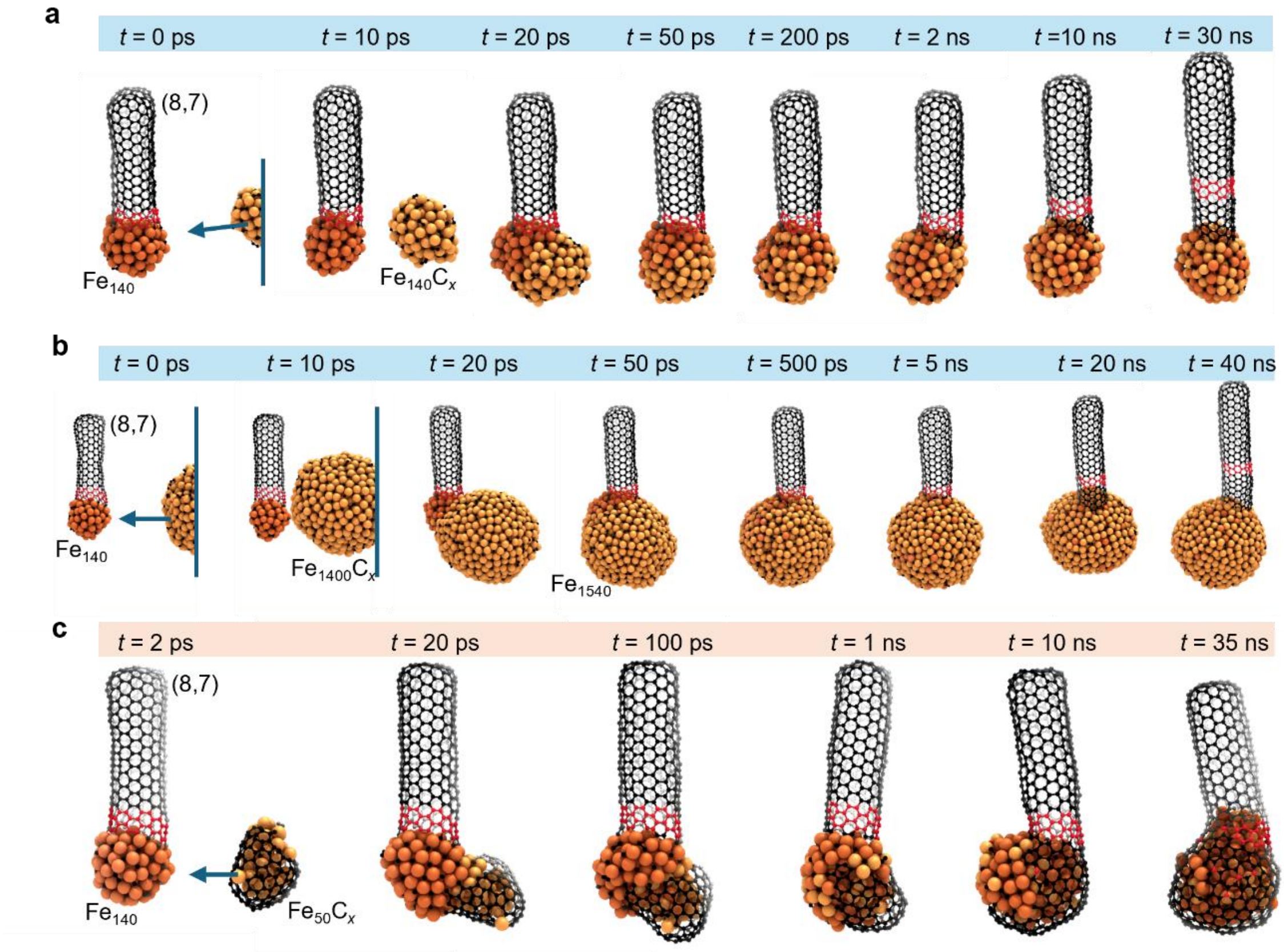

Supplementary Fig. 19 | Catalyst coalescence during CNT growth. a Snapshots of an (8,7) CNT attached to an Fe_{140} catalyst particle before and after coalescence with an active $Fe_{140}C_x$ particle. The CNT grew while maintaining its structure after catalyst coalescence. **b** Snapshots of an (8,7) CNT attached to an Fe_{140} catalyst particle before and after coalescence with a much larger active $Fe_{1400}C_x$ particle, showing chirality preservation despite the large catalyst-size mismatch. **c** Snapshots of an (8,7) CNT attached to an Fe_{140} catalyst particle during coalescence with a carbon-encapsulated, deactivated $Fe_{50}C_x$ particle. The incoming carbon shell/graphene-like nucleus was transferred to the active catalyst surface and induced passivation of the growth interface. These results suggest that catalyst deactivation is governed not simply by catalyst-size evolution, but by whether a new graphitic carbon nucleus is introduced onto the active catalyst surface. Temperature was kept at 1400 K.

Supplementary Table 7 | Similarities and differences between supported- and floating-catalyst CNT growth. For each aspect, the table compares the two platforms, marks whether the present work treats it as common, and recommends how to treat it when relating the systems.

**Item**	**Supported catalysts**	**Floating catalysts**	**Treated as common in this work**	**Recommended treatment**
Catalyst nucleation pathway	Via reduction, dewetting, and surface diffusion[45]	From volatile precursors in the gas phase before/during CNT growth[46,47]	No	Explicitly distinguish
Catalyst evolution pathways	Support-mediated ripening, diffusion into/along the support[48]	Gas-phase coalescence, evaporation, precursor-fed growth[25]	No (assuming catalyst growth in both cases)	Explicitly distinguish
Temperature, residence time, carbon supply path	Spatially stationary local environment; catalytic effect of supports exists[49]	Axial gradients, gas-phase transport only	No	Explicitly distinguish
Growth promoters	H_2O, CO_2, and H_2 are commonly employed to regulate catalyst formation and extend lifetime[50,51]	Sulfur, CO_2, and H_2 are often added[51–53], but their necessity is system-dependent (HiPco does not use S promoter[46])	No	Effects depend on the growth stage at which they act[54]; not easily generalized
CNT nucleation	Following catalyst formation; nucleation density and diameter modulated by nucleation-stage conditions[55,56]	Coupled to gas-phase catalyst formation; Nucleation timing and diameter modulated by promoters[57]	Partly	Fundamental theories apply to both platforms[58–61]; catalyst-tube size usually correlates
Catalyst–CNT interface dynamics	Tip-growth and base-growth coexist[62]	Close to unsupported MD simulations[17,18,63]	Yes	Mechanisms should be shared with caution[64,65]
Structural robustness of CNTs after nucleation	Established for constant growth conditions, but not for evolving growth conditions	Open question for CNT ensembles; influence of sulfur is discussed theoretically[61]	Yes	Should be shared with caution
Elongation rate / kinetics	Measurable at the single-tube level; mostly 0.1–1 $\mu m\ s^{-1}$ at 800–900 °C	Direct measurement is challenging; estimated from length and residence time; influenced by promoters[66]	Mostly (see Suppl. Fig. 1)	Order-of-magnitude comparable at matched temperature; described within the same kinetic model[5]
Substrate-induced deformation and alignment	Often important to achieve isolation and alignment; strong substrate interaction can induce defect formation (Suppl. Note 13 and 14).	Not relevant	No	Specific to supported-growth systems
Is catalyst phase constrained by support epitaxy?	Possible in some systems, but system-dependent[67]; no clear effect has been reported for Fe on quartz	Not relevant	No	Requires caution for supported-catalyst cases

Supplementary references

1. Otsuka, K. et al. Digital isotope coding to trace the growth process of individual single-walled carbon nanotubes. *ACS Nano* **12**, 3994–4001 (2018).
2. Ding, E.-X. et al. Highly conductive and transparent single-walled carbon nanotube thin films from ethanol by floating catalyst chemical vapor deposition. *Nanoscale* **9**, 17601–17609 (2017).
3. Prévot, G. Ostwald ripening of three-dimensional clusters on a surface studied with an ultrafast kinetic Monte Carlo algorithm. *Phys. Rev. B* **84**, 045434 (2011).
4. Pimonov, V. et al. Dynamic instability of individual carbon nanotube growth revealed by in situ homodyne polarization microscopy. *Nano Lett.* **21**, 8495–8502 (2021).
5. Otsuka, K. et al. Universal map of gas-dependent kinetic selectivity in carbon nanotube growth. *ACS Nano* **16**, 5627–5635 (2022).
6. Lee, Y. T. et al. Temperature-dependent growth of carbon nanotubes by pyrolysis of ferrocene and acetylene in the range between 700 and 1000 °C. *Chem. Phys. Lett.* **372**, 853–859 (2003).
7. Zhu, Z. et al. Rate-selected growth of ultrapure semiconducting carbon nanotube arrays. *Nat. Commun.* **10**, 4467 (2019).
8. Chiang, W. H. & Sankaran, R. M. Relating carbon nanotube growth parameters to the size and composition of nanocatalysts. *Diam. Relat. Mater.* **18**, 946–952 (2009).
9. Novikov, I. V. et al. Residence time effect on single-walled carbon nanotube synthesis in an aerosol CVD reactor. *Chem. Eng. J.* **420**, 129869 (2021).
10. Anisimov, A. S. et al. Mechanistic investigations of single-walled carbon nanotube synthesis by ferrocene vapor decomposition in carbon monoxide. *Carbon* **48**, 380–388 (2010).
11. Hussain, S. et al. Measurement of high carbon nanotube growth rate, mass production, agglomeration, and length in a floating catalyst chemical vapor deposition reactor. *ACS Nano* **19**, 8739–8752 (2025).
12. Kim, S. H. & Zachariah, M. R. In-flight kinetic measurements of the aerosol growth of carbon nanotubes by electrical mobility classification. *J. Phys. Chem. B* **110**, 4555–4562 (2006).
13. Ozel, T., Abdula, D., Hwang, E. & Shim, M. Nonuniform compressive strain in

horizontally aligned single-walled carbon nanotubes grown on single crystal quartz. *ACS Nano* **3**, 2217–2224 (2009).

14. Laiho, P., Mustonen, K., Ohno, Y., Maruyama, S. & Kauppinen, E. I. Dry and direct deposition of aerosol-synthesized single-walled carbon nanotubes by thermophoresis. *ACS Appl. Mater. Interfaces* **9**, 20738–20747 (2017).
15. Timans, P. J. Emissivity of silicon at elevated temperatures. *J. Appl. Phys.* **74**, 6353–6364 (1993).
16. Ishimaru, R., Otsuka, K., Inoue, T., Chiashi, S. & Maruyama, S. Carbon dioxide triggers carbon nanotube nucleation: isotope labeling study on the growth process of individual nanotubes. *ECS J. Solid State Sci. Technol.* **11**, 071002 (2022).
17. Yuan, Q., Xu, Z., Yakobson, B. I. & Ding, F. Efficient defect healing in catalytic carbon nanotube growth. *Phys. Rev. Lett.* **108**, 245505 (2012).
18. Hedman, D. et al. Dynamics of growing carbon nanotube interfaces probed by machine learning-enabled molecular simulations. *Nat. Commun.* **15**, 4076 (2024).
19. Navas, H. et al. Unveiling the evolutions of nanotube diameter distribution during the growth of single-walled carbon nanotubes. *ACS Nano* **11**, 3081–3088 (2017).
20. He, M. et al. Linking growth mode to lengths of single-walled carbon nanotubes. *Carbon* **113**, 231–236 (2017).
21. Nasibulin, A. G., Pikhitsa, P. V., Jiang, H. & Kauppinen, E. I. Correlation between catalyst particle and single-walled carbon nanotube diameters. *Carbon* **43**, 2251–2257 (2005).
22. Gao, J. et al. Molecular evolutionary growth of ultralong semiconducting double-walled carbon nanotubes. *Adv. Sci.* **10**, 2205025 (2023).
23. Chai, L. et al. Symmetry and asymmetry in mutations and memory retention during the evolutionary growth of carbon nanotubes. *J. Am. Chem. Soc.* **147**, 19498–19507 (2025).
24. Weller, L. et al. Mapping the parameter space for direct-spun carbon nanotube aerogels. *Carbon* **146**, 789–812 (2019).
25. Hoecker, C., Smail, F., Bajada, M., Pick, M. & Boies, A. Catalyst nanoparticle growth dynamics and their influence on product morphology in a CVD process for continuous carbon nanotube synthesis. *Carbon* **96**, 116–124 (2016).
26. Sebastian, F. L. et al. Absolute quantification of sp^3 defects in semiconducting

single-wall carbon nanotubes by Raman spectroscopy. *J. Phys. Chem. Lett.* **13**, 3542–3548 (2022).

27. Lin, L. et al. Towards super-clean graphene. *Nat. Commun.* **10**, 1912 (2019).
28. Steiner, M. et al. How does the substrate affect the Raman and excited state spectra of a carbon nanotube? *Appl. Phys. A Mater. Sci. Process.* **96**, 271–282 (2009).
29. Feng, X., Matranga, C., Vidic, R. & Borguet, E. A vibrational spectroscopic study of the fate of oxygen-containing functional groups and trapped CO_2 in single-walled carbon nanotubes during thermal treatment. *J. Phys. Chem. B* **108**, 19949–19954 (2004).
30. Thurakitseree, T. et al. Reversible diameter modulation of single-walled carbon nanotubes by acetonitrile-containing feedstock. *ACS Nano* **7**, 2205–2211 (2013).
31. Hasegawa, K. & Noda, S. Millimeter-tall single-walled carbon nanotubes rapidly grown with and without water. *ACS Nano* **5**, 975–984 (2011).
32. Ding, L. et al. Direct observation of the strong interaction between carbon nanotubes and quartz substrate. *Nano Res.* **2**, 903–910 (2009).
33. Yang, J. et al. Radial deformation of single-walled carbon nanotubes on quartz substrates and the resultant anomalous diameter-dependent reaction selectivity. *Nano Res.* **8**, 3054–3065 (2015).
34. Kocabas, C., Shim, M. & Rogers, J. A. Spatially selective guided growth of high-coverage arrays and random networks of single-walled carbon nanotubes and their integration into electronic devices. *J. Am. Chem. Soc.* **128**, 4540–4541 (2006).
35. Bi, J. et al. Near-infrared spectroscopic study and handedness discrimination of large-diameter single-walled carbon nanotubes. *Carbon* **246**, 120910 (2026).
36. Xiao, J. et al. Alignment controlled growth of single-walled carbon nanotubes on quartz substrates. *Nano Lett.* **9**, 4311–4319 (2009).
37. Hertel, T., Walkup, R. E. & Avouris, P. Deformation of carbon nanotubes by surface van der Waals forces. *Phys. Rev. B* **58**, 13870–13873 (1998).
38. Thompson, A. P. et al. LAMMPS - a flexible simulation tool for particle-based materials modeling at the atomic, meso, and continuum scales. *Comput. Phys. Commun.* **271**, 108171 (2022).
39. Wang, H., Zhang, L., Han, J. & E, W. DeePMD-kit: A deep learning package for many-body potential energy representation and molecular dynamics. *Comput.*

Phys. Commun. **228**, 178–184 (2018).

40. Nosé, S. A unified formulation of the constant temperature molecular dynamics methods. *J. Chem. Phys.* **81**, 511–519 (1984).
41. Hoover, W. G. Canonical dynamics: Equilibrium phase-space distributions. *Phys. Rev. A* **31**, 1695–1697 (1985).
42. Hopfield, J. J. Kinetic Proofreading: A new mechanism for reducing errors in biosynthetic processes requiring high specificity. *Proc. Natl. Acad. Sci.* **71**, 4135–4139 (1974).
43. Wu, M. et al. Seeded growth of large single-crystal copper foils with high-index facets. *Nature* **581**, 406–410 (2020).
44. Xu, Z. & Ding, F. Catalyst particle size dependent carbon nanotube cloning. *Carbon* **175**, 69–76 (2021).
45. Bedewy, M. et al. Measurement of the dewetting, nucleation, and deactivation kinetics of carbon nanotube population growth by environmental transmission electron microscopy. *Chem. Mater.* **28**, 3804–3813 (2016).
46. Nikolaev, P. et al. Gas-phase catalytic growth of single-walled carbon nanotubes from carbon monoxide. *Chem. Phys. Lett.* **313**, 91–97 (1999).
47. McLean, B., Kauppinen, E. I. & Page, A. J. Initial competing chemical pathways during floating catalyst chemical vapor deposition carbon nanotube growth. *J. Appl. Phys.* **129**, 044302 (2021).
48. Sakurai, S. et al. Role of subsurface diffusion and Ostwald ripening in catalyst formation for single-walled carbon nanotube forest growth. *J. Am. Chem. Soc.* **134**, 2148–2153 (2012).
49. Wu, Q. et al. Growing single-walled carbon nanotubes from alumina sheet supported catalyst and investigating carrier effects on chirality distribution. *Carbon* **238**, 120279 (2025).
50. Hata, K. et al. Water-assisted highly efficient synthesis of impurity-free single-walled carbon nanotubes. *Science* **306**, 1362–1364 (2004).
51. Magrez, A., Seo, J. W., Kuznetsov, V. L. & Forró, L. Evidence of an equimolar C_2H_2–CO_2 reaction in the synthesis of carbon nanotubes. *Angew. Chem. Int. Ed.* **46**, 441–444 (2007).
52. Bogdanova, A. R., Krasnikov, D. V. & Nasibulin, A. G. The role of sulfur in the

CVD carbon nanotube synthesis. *Carbon* **210**, 118051 (2023).

53. Tian, Y. et al. Growth of single-walled carbon nanotubes with controlled diameters and lengths by an aerosol method. *Carbon* **49**, 4636–4643 (2011).
54. Hoecker, C., Smail, F., Pick, M., Weller, L. & Boies, A. M. The dependence of CNT aerogel ssynthesis on sulfur-driven catalyst nucleation processes and a critical catalyst particle mass concentration. *Sci. Rep.* **7**, 14519 (2017).
55. Sakurai, S., Inaguma, M., Futaba, D. N., Yumura, M. & Hata, K. Diameter and density control of single-walled carbon nanotube forests by modulating ostwald ripening through decoupling the catalyst formation and growth processes. *Small* **9**, 3584–3592 (2013).
56. Lee, J., Abdulhafez, M. & Bedewy, M. Decoupling catalyst dewetting, gas decomposition, and surface reactions in carbon nanotube forest growth reveals dependence of density on nucleation temperature. *J. Phys. Chem. C* **123**, 28726–28738 (2019).
57. Liu, Q. et al. Diameter-selective growth of single-walled carbon nanotubes with high quality by floating catalyst method. *ACS Nano* **2**, 1722–1728 (2008).
58. Artyukhov, V. I., Penev, E. S. & Yakobson, B. I. Why nanotubes grow chiral. *Nat. Commun.* **5**, 4892 (2014).
59. Diaz, M. C., Jiang, H., Kauppinen, E., Sharma, R. & Balbuena, P. B. Can single-walled carbon nanotube diameter be defined by catalyst particle diameter? *J. Phys. Chem. C* **123**, 30305–30317 (2019).
60. McLean, B., Page, A. J. & Ding, F. How oxygen influences the catalytic activity of iron during carbon nanotube nucleation. *J. Appl. Phys.* **139**, 074301 (2026).
61. An, H. et al. The Role of sulfur in single-walled carbon nanotube growth. *ACS Nano* **20**, 11558–11565 (2026).
62. Gohier, A., Ewels, C. P., Minea, T. M. & Djouadi, M. A. Carbon nanotube growth mechanism switches from tip- to base-growth with decreasing catalyst particle size. *Carbon* **46**, 1331–1338 (2008).
63. Förster, G. D., Swinburne, T. D., Jiang, H., Kauppinen, E. & Bichara, C. A semi-grand canonical kinetic Monte Carlo study of single-walled carbon nanotube growth. *AIP Adv.* **11**, 045306 (2021).
64. Jourdain, V. & Bichara, C. Current understanding of the growth of carbon

nanotubes in catalytic chemical vapour deposition. *Carbon* **58**, 2–39 (2013).

65. Magnin, Y., Amara, H., Ducastelle, F., Loiseau, A. & Bichara, C. Entropy-driven stability of chiral single-walled carbon nanotubes. *Science* **362**, 212–215 (2018).

66. Inoue, H., Karakassides, A., Fujimori, T. & Jiang, H. Halogen-promoted long single-walled carbon nanotube growth by floating catalyst chemical vapor deposition for high-performance transparent conductive films. *Carbon* **253**, 121426 (2026).

67. He, M. et al. Chiral-selective growth of single-walled carbon nanotubes on lattice-mismatched epitaxial cobalt nanoparticles. *Sci. Rep.* **3**, 1460 (2013).
