# Supplementary material for: Isotope-labeled growth histories reveal persistent chirality in individual carbon nanotubes despite catalyst evolution
Source: Nat Commun. 2026 Sep 1;17:8898. doi: 10.1038/s41467-026-77101-2 (PMC13534721; doi:10.1038/s41467-026-77101-2)
Supplement: Supplementary file 2 [file 41467_2026_77101_MOESM2_ESM.pdf]

Description of Additional Supplementary Files

File name: Supplementary Movie 1.mp4

Description: Visualization of the growth simulation of a (8,7) CNT growing from an iron nanoparticle that gradually grows in size. Atoms in red represent the hexagons located at the tube edge at t = 0. Snapshots of this growth trajectory are provided in Fig. 5e.

File name: Supplementary Movie 2.mp4

Description: Visualization of the growth simulation of a (8,7) CNT growing from a large iron nanoparticle (Fe1389). Atoms in red represent the hexagons located at the tube edge at t = 0. Snapshots of this growth trajectory are provided in Supplementary Fig. 18c.

File name: Supplementary Movie 3.mp4

Description: Visualization of the growth simulation of a (13,0) CNT growing from an iron nanoparticle that gradually grows in size. Atoms in red represent the hexagons located at the tube edge at t = 0. Snapshots of this growth trajectory are provided in Supplementary Fig. 18e.
