# Supplementary material for: Isotope-labeled growth histories reveal persistent chirality in individual carbon nanotubes despite catalyst evolution
Source: Nat Commun. 2026 Sep 1;17:8898. doi: 10.1038/s41467-026-77101-2 (PMC13534721; doi:10.1038/s41467-026-77101-2)
Supplement: Supplementary file 4 [file 41467_2026_77101_MOESM7_ESM.pdf]

nature portfolio

Peer Review File

Isotope-labeled growth histories reveal persistent chirality in individual carbon nanotubes despite catalyst evolution

Corresponding Author: Professor Keigo Otsuka

This file contains all reviewer reports in order by version, followed by all author rebuttals in order by version.

Version 0:

Reviewer comments:

Reviewer #1

(Remarks to the Author)
In this manuscript, the authors investigated the influence of temperature and catalyst particle size on the growth kinetics and chirality of single-wall carbon nanotubes (SWCNTs), mainly by tracing the growth histories of substrate-supported individual nanotubes using isotope labeling under modulated environments. It is concluded that whereas the growth rate responds sensitively to environmental changes, the diameter and chirality of SWCNTs are robustly preserved during subsequent elongation, and diameter and chirality control is established at the nucleation stage. Although this work was performed carefully, the methodology and main conclusions lack innovation and exciting points for the research community, such as guidance to the chirality control of SWCNTs. In addition, the substrate-supported growth and floating catalyst CVD (FCCVD) growth of SWCNTs are quite different in both growth kinetics and nucleation thermodynamics. For example, the growth rate of the CNTs by FCCVD method and be several order of magnitude faster than that of substrate-supported growth; and sulfur (thiophene as a precursor in this work) is usually used as a growth promoter for FCCVD, thus the sizing effect of these growth routes are substantially different. So, it is nonsense to discuss the growth kinetics and catalyst coarsening of these two methods together.
Therefore, I do not recommend publication of this manuscript in NC.

Reviewer #2

(Remarks to the Author)
The authors investigate the growth of single-walled carbon nanotubes (CNTs) under dynamically evolving environments and trace CNT growth histories using isotope labeling. Based on the observed growth-rate hysteresis induced by temperature variation, the authors conclude that irreversible catalyst coalescence occurs upon increasing the reaction temperature, while the CNT structure remains unchanged throughout the entire growth process. However, the present manuscript does not provide fundamentally new insights into CNT growth mechanisms. The main concerns are outlined below:
1. The authors attempt to generalize their conclusions to both gas-phase growth and substrate-supported processes. However, in typical floating catalyst CVD systems, the actual growth time of an individual CNT is generally shorter than 1 s. As a result, CNT growth may have already terminated before any significant catalyst evolution can be induced by changing the reaction temperature, calling into question the validity of correlating temperature-induced catalyst coalescence with CNT growth behavior in floating catalyst systems.
2. It remains unclear whether the active catalyst particles indeed undergo structural evolution under dynamic reaction conditions. Although AFM characterization was performed on catalysts after CNT removal, this approach cannot distinguish between previously active and inactive particles. Moreover, due to the encapsulation of Fe nanoparticles by CNTs during growth, the active catalysts may be effectively protected from coarsening or diameter changes when the reaction temperature is varied.
3. In addition, several research groups have conducted extensive studies on catalyst evolution during CNT growth using environmental transmission electron microscopy. In their works, significant catalyst coarsening during active CNT growth has rarely been observed, which appears inconsistent with the conclusions drawn in the present manuscript.
4. Another concern relates to the temperature change protocol employed in this work. Although the furnace set temperature is adjusted during the experiment, a finite thermal response time is required for the temperature change to be effectively transmitted to the catalyst nanoparticles. Consequently, the actual reaction time experienced by the catalyst at each

temperature is difficult to define accurately. This uncertainty complicates the precise determination of CNT growth rates and may undermine the reliability of the growth-rate analysis under dynamic temperature conditions.
5. The authors' claim regarding the decoupling of structural memory and growth rate does not represent a new concept within the CNT synthesis community. CNT growth is commonly understood to consist of distinct nucleation and growth stages, during which the CNT structure is primarily determined during nucleation, while the subsequent growth rate can vary without altering the established structure. Moreover, substantial progress has already been made in achieving structure-controlled CNT growth through the regulation of nucleation processes or growth kinetics, as reported by Yan Li's group in Peking University. As such, the novelty and significance of the authors' conclusions in this regard are limited.
6. Moreover, chirality changes during CNT growth are generally considered highly unlikely. According to theoretical studies (for example, Chem, 5, 1-12 (2009)), chirality mutations require the incorporation of pentagon-heptagon defect pairs into the CNT sidewall. The associated energy barriers for such processes are extremely high (larger than 3 eV), which likely accounts for the robust preservation of CNT chirality even under dynamically changing reaction environments. Consequently, the observed retention of CNT structure in this work is consistent with established theoretical understanding rather than providing new mechanistic insight.
7. Some minor comments: i) It is generally not reliable to distinguish metallic and semiconducting CNTs solely based on the G-band features, as the G mode can be influenced by multiple factors and does not provide unambiguous electronic-type assignment. ii) Line 63: the phrase "HiPco-derived samples have revealed" appears to be incorrect and should be revised to "HiPco-derived samples have not revealed".

Reviewer #3

(Remarks to the Author)
In this manuscript, the authors report growth-kinetics measurements of individual single-walled carbon nanotubes (SWCNTs) under a temperature gradient, using an isotope-encoding method. They reveal a clear hysteresis in both the average lifetime and the growth rate (at the ensemble level as well as for individual nanotubes) between temperature ramp-up and ramp-down, manifested by distinct activation energies in the two regimes.
To rationalize these observations, the authors propose a model based on two key assumptions:
(i) under the experimental conditions employed, the CNT growth rate increases with increasing catalyst particle size (i.e. surface area);
(ii) the catalyst particle size increases with temperature as a result of Ostwald ripening.
The calculated trends are compared with the experimental data and show good qualitative agreement. An even better agreement is obtained when considering only large individual catalyst nanoparticles, (although this aspect is described with less precision than the rest of the analysis). The authors further observe that, while the CNT growth rate systematically varies with temperature, the nanotube chirality remains unchanged in most cases. This finding contrasts with previous reports (e.g. Yao et al., 2007).
From these results, the authors conclude that chirality selection occurs at the nucleation stage, whereas the nanotube length is determined during the elongation stage. This decoupling is proposed to explain the relative insensitivity of the diameter distribution to furnace temperature in floating-catalyst CVD growth.
Overall, I am convinced by the experimental methodology, the data interpretation, the modeling approach, the quality and relevance of the arguments, and the main conclusions. This manuscript offers a rigorous and scientifically robust analysis of CNT growth mechanisms, which is highly appreciated.
As detailed below, I have minor disagreements with the interpretation of the Raman data used to assess the degree of chirality preservation (which do not affect the main claims of the authors), as well as many requests for additional information, clarification, and discussion. These points are intended to further strengthen the manuscript and make its conclusions more robust and convincing to a broad readership.

Main points:
1. « growth rate scales with the catalyst surface area » (Otsuka et al., p. 13). The authors should explain what would be the corresponding kinetic regime in the frame of their kinetic model in "Universal Map..." (ACS Nano 2022, 16, 5627–5635) and whether it would only be dependent of the catalyst surface area or also on the CNT chirality.
2. Linear versus non-linear growth: If the proposed scenario is correct, one would also expect to observe nanotubes displaying an increase in growth rate even under constant growth conditions (eg in conditions where the characteristic time of Ostwald ripening is close to the CNT growth lifetime). The authors should comment on whether they observed such cases.
3. The authors should discuss whether their results agree with the mechanisms previously proposed to account for the influence of growth parameters on the CNT diameter distribution (eg Lu et al (J. Phys. Chem. B 2006, 110, 41, 20254–20257), Navas et al (ACS Nano 2017, 11, 3, 3081–3088).
4. Additional AFM data (other samples, other regions) would be welcome to confirm the generality of the Ostwald ripening observation that is key for the main claim: especially in the case of 800-873-800 experiments to demonstrate that the (short) stay at high temperature was sufficient to cause significant Ostwald ripening.
5. « Er of 2.6 eV » (Otsuka et al., p. 15): To be more convincing, this activation energy value should be discussed in comparison with those previously reported in the literature for Ostwald ripening for similar systems.
6. « Distributions of apparent Ea, which are calculated from T ramp-up (red) and ramp down (blue) periods in KMC simulations. » (Otsuka et al., p. 16) In the calculations, the apparent Ea for T ramp-up is around 2 eV, while it is around 3-4 eV experimentally (fig. 3e). I understand that the authors later explain this discrepancy by the hypothesis that « Relatively large ones among simulated particles in panel (f) are assumed to grow CNTs. » (Otsuka et al., p. 16) and « we assume that CNT growth preferentially occurs on relatively larger catalyst particles, » (Otsuka et al., p. 17). The whole section is a bit unclear (is it just a qualitative comment? Or is it actually used in the latest version of the calculations?). The authors should

precise which values or threshold size correspond to "larger particles" or “relatively large ones", and whether and how they integrated such a threshold size in their latest modelling version (eg for figure 4g)”.

7. « Among 158 CNTs analyzed, 139 tubes (88%) maintained their chirality (n,m) over the entire measured length. The total inspected length was 9.39 mm, within which 24 (n,m) change events were observed, corresponding to a length-normalized frequency of 2.6 mm−1 (391 μm). » (Otsuka et al., p. 19): The authors appear to have neglected the most probable scenario: given that there is no a priori reason for the new chirality to maintain the same resonance energy, a chirality change likely results in a complete loss of resonance at the excitation energy used (532 nm). In the Raman map at 532 nm, this would manifest as a short segment—either (i) distinct from the catalyst line (easily identifiable) or (ii) connected to it (more difficult to distinguish from normal tubes). While the few provided Raman maps seem to show few such instances, their potential presence warrants discussion and consideration in the analysis.

8. « As shown in Fig. 5c, Raman mapping measurements further verify the RBM continuity along nanotubes (Supplementary Fig. 2), demonstrating chirality preservation in the FCCVD system. » (Otsuka et al., p. 20): Again, the most plausible explanation is that the chirality change leads to a total loss of resonance at the excitation energy used (532 nm), rendering the tube with the new chirality undetectable, rather than merely causing a discontinuity in the RBM features. To confirm the absence of a chirality change, additional SEM images or Raman maps at other wavelengths of the same region may be provided. This would also require the tubes to be sufficiently dispersed and unbundled for individual resolution, a condition that may be challenging to meet with these samples.

9. The authors should propose an explanation for the discrepancy between their results and those of Yao et al. (Temperature-mediated growth of single-walled carbon-nanotube intramolecular junctions. Nat. Mater. 6, 293–296 (2007)): is it more likely a difference in experimental conditions (if so, which key difference?), or a potential flaw in the methodology used by Yao et al?

Other points:

« Viewed from the standpoint of the growing material, these processes could be regarded as Eulerian and Lagrangian descriptions, respectively. » (Otsuka et al., p. 3) « From a Lagrangian perspective » (Otsuka et al., p. 4): A bit jargon laden. I understand what is meant but it could be stated more simply.

« whereas floating catalysts enlarge mainly through particle coalescence at relatively low temperatures 11 » (Otsuka et al., p. 3): Beside theoretical work (ref. 11), citations of experimental observations would also be welcome to support this assertion.

« have revealed » (Otsuka et al., p. 4): Should this instead be interpreted as "have not revealed"?

« supported by recent MD simulations using machine-learning force fields28,29. » (Otsuka et al., p. 4): It should be more specifically explained in which way these recent MD simulations support "the preservation of chirality over hundreds of micrometers".

« Supplementary Fig. 1 » (Otsuka et al., p. 7): the Raman excitation wavelength should be indicated.

« by extrapolating the growth rate defined between the last two isotope labels down to the catalyst stripe » (Otsuka et al., p. 9): Understood but it could be formulated more clearly.

« the semiconducting-to-metallic tube ratio remains essentially unchanged over the temperature range examined (Supplementary Fig. 3) » (Otsuka et al., p. 10) Figure S3 may more clearly describe the average SC/M ratio (e.g. pondered over the 3 wavelengths) in each of the three cases (800, 875 °C, 800-873-800)

« c) Survival probability p over a 1 s interval for a growing CNT plotted against temperature. Arrows show the direction of temperature modulation. Error bars represent the standard errors. » (Otsuka et al., p. 12): Figure 3c: a table detailing the data used to calculate the probabilities and the error bars should be given in SI. I would suggest plotting the confidence interval (more statistically meaningful), rather than the standard deviation, for the uncertainty bars.

« e) Distributions of apparent Ea obtained by linear fitting of the Arrhenius plots, separately for temperature ramp-up (T up) and ramp-down (T down) periods. » (Otsuka et al., p. 12): Fig. 3e: to evaluate the data quality, all the Arrhenius plots used to build fig 3e should be (more) clearly shown in SI, not only those in Fig. 3d.”

« Note that transition from tangential to perpendicular modes alone cannot explain this behavior because such a transition should involve the catalyst size change as long as the tube chirality remains unchanged. » (Otsuka et al., p. 13) A bit unclear: please reformulate more clearly.

« empirically scales as R^4» (Otsuka et al., p. 14) The authors should provide an additional citation to justify their use of a power-law exponent of 4 in this context, as opposed to the conventional Ostwald ripening law with a power of 3.

« this ensemble model agrees well with the average growth rate for s-CNTs (Fig. 2f) » (Otsuka et al., p. 15): Why mentioning s-SWCNTs only? The authors previously wrote that m-CNTs and s-CNTs have close kinetics.

« Consistently, a comparable hysteretic behavior found in the growth lifetime (Fig. 3c) provides independent evidence for irreversible catalyst coarsening, » (Otsuka et al., p. 18) Does it mean that the authors conclude that Ostwald ripening is the main cause of catalyst deactivation in these conditions? If so, how their model could account (at least qualitatively) for the growth lifetime hysteresis?

« Figure 5a shows Raman profiles acquired along three representative CNTs. » (Otsuka et al., p. 18) The temperature profile experienced by each tube and the instantaneous growth rate (extracted from the digital isotope encoding) should be shown in fig. 5a and S7, to more clearly support the conclusion that the change in temperature does not systematically lead to a change in chirality at the individual CNT level.

« in contrast, tube #7 is a rare example in which an abrupt spectral change is observed for both G-mode and RBM. » (Otsuka et al., p. 18). In the type of case displayed by tube #7, the authors should explain how they distinguish a bundle of two tubes (both emanating from the catalyst line) from a change in chirality.

« see Supplementary Fig. 10 for detailed D-mode analysis » (Otsuka et al., p. 19) What is the explanation of the authors for the disappearance of the D band at 400°C under vacuum? An extrinsic factor?

Sup Info:

- « The fraction of semiconducting CNTs is shown for each excitation wavelength. » (Otsuka et al., p. 10): Please add the

excitation wavelength on each graph. Please precise what is the value expressed in “µm-1“ (linear nanotube density?).
- « Diameter distribution of CNTs grown under for three different temperature profiles. Excitation wavelength of 532 nm is used. Average diameter is indicated by the vertical dashed lines. » (Otsuka et al., p. 10). Why using only the 532-nm data? (the authors have RBM data at other wavelengths for reference growths at constant T).
- « Arrhenius plots of the growth rate for a large number of individual CNTs. » (Otsuka et al., p. 12): the ramp-up and ramp-down data should be shown separately for clarity.
- « Apparent activation energy Ea plotted against the average temperature of each growth segment during the temperature ascent » (Otsuka et al., p. 12) If available, the same data should also be shown for ramp-down.
- « Comparison of apparent activation energies Ea extracted from the T ramp-up and ramp-down periods for CNTs that grew across both stages. » (Otsuka et al., p. 12) The Arrhenius plots should also be shown separately to more clearly assess the data quality.

Reviewer #4

(Remarks to the Author)
The catalytic growth mechanism of SWCNTs, particularly the kinetic aspects and role of catalysts, has long been a focus of interest in the field of nanotubes. Previous work has primarily remained theoretical, with very limited experimental progress. Now, this work ingeniously employs isotopic labeling combined with growth condition fluctuations to reveal the kinetic growth mechanism of SWCNTs. Authors reported that chirality control can be established during the nucleation stage, while subsequent elongation can be optimized independently, this finding is quite important for the structure-controlled synthesis of SWCNTs. The experimental design is smart, and the characterization is comprehensive, providing a clear experimental picture of nanotube growth. This represents a significant breakthrough in the field and holds profound implications for the CNT-based electronic devices. This work will be of interest to researchers in the fields of nanotubes and other related material growth, and is therefore worthy of publication in Nature Communications after suitable minor revision.

1. Considering the strong van der Waals forces and epitaxial alignment between the quartz lattice and the SWCNTs, could this external constraint have enhanced the structural stability of the nanotubes?
2. On line 395, the authors draw an analogy between the chirality-preserving phenomena in SWCNT growth kinetics and DNA replication, which is physically inappropriate. Can authors revise or change this description?
3. The use of the Arrhenius equation to calculate activation energy (Ea) is strictly valid only under steady-state conditions. However, the temperature-programmed growth process in this study is a non-steady-state process. During temperature evolving, the catalyst particles may undergo evolution (e.g., carbon solubility), meaning the pre-exponential factor (A) and the activation energy (Ea) of the system may be changed. For instance, temperature rise increases the carbon solubility in the catalyst, while temperature decrease causes a reduction in solubility and carbon precipitation. Can author provide some discussion or explanation the effect of non-steady-state process on activation energy (Ea)?
4. The work demonstrates that a post-growth snapshot captures a catalyst particle that has coarsened long after the nanotube's diameter was fixed. Therefore, the widely reported 1:1 or 1:2 diameter ratio between the tube and its residual catalyst particle may be largely coincidental. Is it possible to discuss this point by estimating the expected post-growth catalyst size based on their coarsening model and comparing it to the as-grown tube diameters from their own data?

Version 1:

Reviewer comments:

Reviewer #1

(Remarks to the Author)
The authors tried to address the concerns raised by the reviewer, however, I am not convinced by the responses.
1 As for the difference in the growth rates of CNTs by floating catalyst CVD and substrate supported CVD, the authors proposed that "growth rates (in µm/min) of supported-catalyst systems and floating-catalyst systems fall within the same order of magnitude if the temperature difference is taken into account. " Actually, the influence of substrate on the growth kinetics of CNTs must be taken into account, rather than simply comparing the activation energies.
2 Regarding the role of sulfur growth promoter, the authors argued that "its quantitative impact on CNT properties is generally moderate rather than dramatic. " This is absolutely not true. In most FCCVD growth cases, the use of a small amount of sulfur plays a crucial role determining the yield, purity, growth rate and diameter of the CNTs obtained. The example raised by the authors [Chemical Engineering Journal 378, 122010(2019)] is not a representative case.

Except for these above issues relating to some specific conditions, actually, my concern is that the current work does not present a general breakthrough in the understanding of the growth of CNTs. Thus, I think it is suitable for publication in more specialized journals, rather than in NC.

Reviewer #2

(Remarks to the Author)
In their response, the authors devote considerable effort to emphasizing the novelty of their manuscript. After carefully reviewing both the manuscript and the replies, I find myself largely in agreement with the first reviewer: CNT growth via FCCVD differs fundamentally from substrate-supported growth, particularly in terms of nucleation behavior and growth kinetics. Notably, the present work does not introduce any new experimental additives or strategies for FCCVD. In addition,

the authors attempt to decouple structural memory from kinetic adaptability in CNT growth, a concept that is already well established and widely accepted within the CNT research community. On this basis, I am unable to recommend the manuscript for publication. My specific comments on the authors' responses are as follows:

1. The authors state that CNT growth times in FCCVD typically range from several seconds to tens of seconds. However, commercially produced CNTs from the HiPco process are only a few micrometers in length. Considering the high growth rates at elevated temperatures, this suggests that the actual growth time is likely shorter than 1 s.
2. To support their discussion on catalyst nanoparticle coalescence, the authors cite prior studies (ACS Nano 11, 3081 (2017); JPCL 1, 918 (2010)). However, these works also report that catalyst coalescence can lead to catalyst deactivation. In contrast, the authors claim that CNT growth continues even after particle coalescence, which appears inconsistent with the cited literature.
3. Over the past two decades, numerous in situ ETEM studies have examined CNT growth from individual catalyst particles (e.g., Nano Lett. 2008, 8, 2082; Science Advances 2022, 8, abo5686; as well as extensive work from the groups of Renu Sharma at ASU and Stephan Hofmann at the University of Cambridge). Across these studies, particle coalescence is generally not observed after CNT nucleation. Therefore, the authors' assertion that CNTs can continue growing following particle coalescence lacks convincing experimental support.
4. Although AFM images are presented to suggest that catalyst particles increase in size after CVD growth, it remains unclear whether this enlargement occurs before or after CNT nucleation. Furthermore, the formation of amorphous carbon coatings could also contribute to apparent particle growth, particularly after catalyst deactivation.
5. Regarding the previously mentioned work (Chem, 5, 1182–1193 (2019); corrected citation), it is shown that chirality transitions require the incorporation of pentagon-heptagon defect pairs, associated with energy barriers exceeding 3 eV. This supports the conclusion that CNTs are likely to preserve their initial structure once nucleated.
6. In addition, prior theoretical work (Nat. Commun. 2024, 5, 4892) from Yakobson's group has already decoupled nucleation thermodynamics from growth kinetics in CNT formation. This concept has been extensively explored in efforts to achieve chirality-specific synthesis. Therefore, the claimed novelty of the present manuscript is not sufficiently convincing.

Reviewer #3

(Remarks to the Author)
The responses to my comments and the corrections made to the manuscript are generally satisfactory, and I thus recommend publishing the manuscript.

A final suggestion related to the response to my comment 8 on "As shown in Fig. 5c, Raman mapping measurements further verify the RBM continuity along nanotubes (Supplementary Fig. 2), demonstrating chirality preservation in the FCCVD system.": a corrective action should be to moderate this conclusion by mentioning in the text the possible biases related to Raman resonance effects, as recognized by the authors in their response.

Reviewer #4

(Remarks to the Author)
The authors have thoroughly addressed the concerns raised in the previous review. The supporting new results and analyses have improved the manuscript's rigor and clarity. In its revised form, I recommend its acceptance for publication on Nature Communications.

Version 2:

Reviewer comments:

Reviewer #2

(Remarks to the Author)
Compared with the previous version, the manuscript has been improved. However, several important concerns regarding the interpretation of the results and the validity of the conclusions remain unresolved.

1. The authors claim that this work "provides a foundation for practical strategies that integrate structural control with scalable growth." Based on the findings presented here, what specific strategies do the authors envision for achieving both controlled SWCNT structures and scalable production? More concrete discussion would strengthen the practical significance of the work.
2. In Figure 1, the evolution of the carbon chemical potential along the reactor coordinate is illustrated. It is generally accepted that the chemical potential of carbon decreases with increasing temperature. Why, then, does the schematic indicate the highest carbon chemical potential at the reaction zone, where the temperature is also the highest? The physical basis for this representation should be clarified.
3. The authors propose that the previously reported volcano-type bandgap dependence may only emerge under specific growth conditions. What are these conditions? Do they relate primarily to the catalyst composition, growth temperature, carbon precursor, or other parameters? A more explicit discussion is needed.
4. Regarding the temperature dependence of the survival probability, the data points presented in Figure 3c appear highly scattered, making it difficult to establish a clear trend. Furthermore, the large error bars raise concerns about the statistical significance and reliability of the reported correlation. The authors should provide additional evidence to support this conclusion.
5. A central conclusion of the manuscript is that different CNT growth rates observed at the same growth temperature arise

from catalyst coalescence, which increases the catalyst surface area. Several concerns should be addressed: (a) Among the CNTs investigated, what fraction exhibits a growth-rate increase attributable to catalyst coalescence? (b) Ethanol was used as the carbon precursor in this study. Under such conditions, a weak-etching growth regime, where elongation rate scales with catalyst surface area, is generally not expected. Moreover, the group's previous studies using ethanol consistent with models in which the CNT growth rate is primarily governed by chiral angle. How can these observations be reconciled with the mechanism proposed here? (c) Following plasma treatment, the catalyst particles exhibit a bimodal size distribution. However, no direct evidence is provided regarding whether smaller or larger catalyst particles preferentially lead to faster CNT elongation. Such evidence would be important to support the proposed mechanism.
6. Additional information regarding the catalyst nanoparticles is required. What are the average particle diameters before and after coalescence? Are the catalyst particles in the solid or liquid state under the CVD growth conditions? Could the change in particle size alter the catalyst phase state and thereby influence the CNT elongation rate?
7. The authors state that "intentional perturbations of the growth environment at the reactor inlet, including precursor concentration and temperature gradients, could be used to confine the nucleation zone, potentially narrowing the diameter distribution." Given the authors' conclusion regarding robust structural memory during growth, it is unclear how such an approach would effectively narrow the diameter distribution. Additional explanation is needed.
8. Among the CNTs investigated, only 88% retained their chiral indices over the measured growth length. What mechanisms are responsible for the chirality changes observed in the remaining 12% of CNTs? Do these observations challenge the conclusion of robust chirality preservation? The authors should discuss this apparent inconsistency.
9. As supporting evidence, molecular dynamics simulations were performed only for a (8, 7) SWCNT, which is known to possess relatively high structural stability. To more comprehensively evaluate chirality robustness during catalyst coalescence, simulations on less stable nanotubes, such as (n,1) or (n, 0) species, are recommended.

Version 3:

Reviewer comments:

Reviewer #2

(Remarks to the Author)
The authors have well addressed the raised comments, and the revision can be accepted now.

Open Access This Peer Review File is licensed under a Creative Commons Attribution 4.0 International License, which permits use, sharing, adaptation, distribution and reproduction in any medium or format, as long as you give appropriate credit to the original author(s) and the source, provide a link to the Creative Commons license, and indicate if changes were made.
In cases where reviewers are anonymous, credit should be given to 'Anonymous Referee' and the source.
The images or other third party material in this Peer Review File are included in the article's Creative Commons license, unless indicated otherwise in a credit line to the material. If material is not included in the article's Creative Commons license and your intended use is not permitted by statutory regulation or exceeds the permitted use, you will need to obtain permission directly from the copyright holder.
To view a copy of this license, visit https://creativecommons.org/licenses/by/4.0/

Response to reviewer report for manuscript No. NCOMMS-26-008054 by Otsuka et al.

We sincerely thank all the reviewers for taking their time to review the manuscript and providing insightful comments, which are indispensable for the improvement of the manuscript. While the comments from the reviewers are written in ***italic blue***, our point-by-point response and revisions made are provided below in **black** and **red**, respectively. Besides the revisions directly reflecting the reviewers' comments, the manuscript has been professionally edited throughout to improve clarity and readability without altering the original intent. To avoid unnecessary visual clutter, only the passages involving content-related changes are highlighted in yellow in the revised main text. Since Supplementary Information has been extensively expanded with a substantial amount of newly added content, no highlighting has been applied there.

Response to Reviewer #1:

Comment. *In this manuscript, the authors investigated the influence of temperature and catalyst particle size on the growth kinetics and chirality of single-wall carbon nanotubes (SWCNTs), mainly by tracing the growth histories of substrate-supported individual nanotubes using isotope labeling under modulated environments. It is concluded that whereas the growth rate responds sensitively to environmental changes, the diameter and chirality of SWCNTs are robustly preserved during subsequent elongation, and diameter and chirality control is established at the nucleation stage. Although this work was performed carefully, the methodology and main conclusions lack innovation and exciting points for the research community, such as guidance to the chirality control of SWCNTs.*

Response. We sincerely thank the reviewer for concisely summarizing our results and stating that the work was performed carefully. We partly agree that this manuscript does not provide "exciting points" for the community, such as a recipe for chirality-specific growth. After a number of exciting reports on structure control over nanotube chirality and higher-order arrays, we believe that comprehensive understanding of underlying mechanisms behind those studies and then new strategies to scale them up will be increasingly important in next several years. The present work will contribute to the community from such a perspective.

Comment. *In addition, the substrate-supported growth and floating catalyst CVD (FCCVD) growth of SWCNTs are quite different in both growth kinetics and nucleation thermodynamics. For example, the growth rate of the CNTs by FCCVD method and be several order of magnitude faster than that of substrate-supported growth; and sulfur (thiophene as a precursor in this work) is usually used as a growth promoter for FCCVD, thus the sizing effect of these growth routes are substantially different. So, it is nonsense to discuss the growth kinetics and catalyst coarsening of these two methods together.*

Response. We appreciate the important comment on the distinction between substrate-supported growth and FCCVD growth. Through careful literature review, however, we find that growth rates (in μm/min) of supported-catalyst systems and floating-catalyst systems fall within the same order of magnitude if the temperature difference is taken into account. As shown in Supplementary Fig. 1, by plotting growth rates in a form of Arrhenius plot, we can clearly see a linear trend among different growth methods with a slope corresponding to the activation energy of ~1.6 eV. In particular, growth rates around T=1100 K show good agreement between two different catalyst configurations. Here, we should emphasize that a similar map has recently been summarized by Hussain et al. [*ACS Nano* **19**, 8739 (2025)]. They cited the original papers on FCCVD that reported "normalized growth rates [$\mu m \cdot s^{-1}$-atm]" by simply extrapolating measured growth rates to the hypothetical case with 1 atm of carbon source (acetylene etc.). Because the actual partial pressure of acetylene was, for example, ~0.01 atm in the study by Chiang and Sankaran [*Diamond & Related Materials* **18**, 946 (2009)], the summary map by Hussain et al. appears to overestimate the growth rate values in some FCCVD studies by a few orders of magnitude. Such a normalization may unintentionally lead to misunderstanding on the growth kinetics of FCCVD among the research community. <u>We have added a sentence in the main text and Supplementary Fig. 1 to clarify this point, which forms the essential foundation of the discussion in our manuscript.</u> If the reviewer had a different dataset in mind, we would be grateful to examine it and clarify the comparison accordingly.

Next, as for the sizing effect of different growth systems, we emphasized that the central focus of our study is the robustness of nanotube chirality against any kind of catalyst size evolution, irrespective of the specific pathway by which the catalyst size changes. In this context, the essential requirement is that catalyst size changes during growth, rather than the particular mechanism by which it is induced.

Regarding the role of sulfur, we agree that it has often been used as a growth promoter in FCCVD systems. However, its quantitative impact on CNT properties is generally moderate rather than dramatic. For example, a systematic study by Kauppinen et al. [*Chemical Engineering Journal* **378,** 122010(2019)] showed that, when varying the H_2S concentration, the optimized growth yield increased by at most a factor of ~2.5 with a 1–2 ppm level, while the bundle length and average diameter increased by only ~20% compared to sulfur-free conditions. These results indicate that sulfur does not fundamentally alter the growth mechanism or diameter selection, but rather tunes optimal growth windows within a given condition (temperature, type of carbon sources, etc.). In our experiments, the sulfur concentration is extremely low (~0.25 ppm), which is even below the minimum concentration explored in the above study. Therefore, our FCCVD system is expected to be in a regime where the influence of sulfur is minimal. Based on these considerations, we conclude that sulfur addition does not introduce a qualitative difference that would invalidate the comparison between FCCVD and substrate-supported growth in the present work. <u>We have added a paragraph discussing this aspect in Supplementary Note 11.</u>

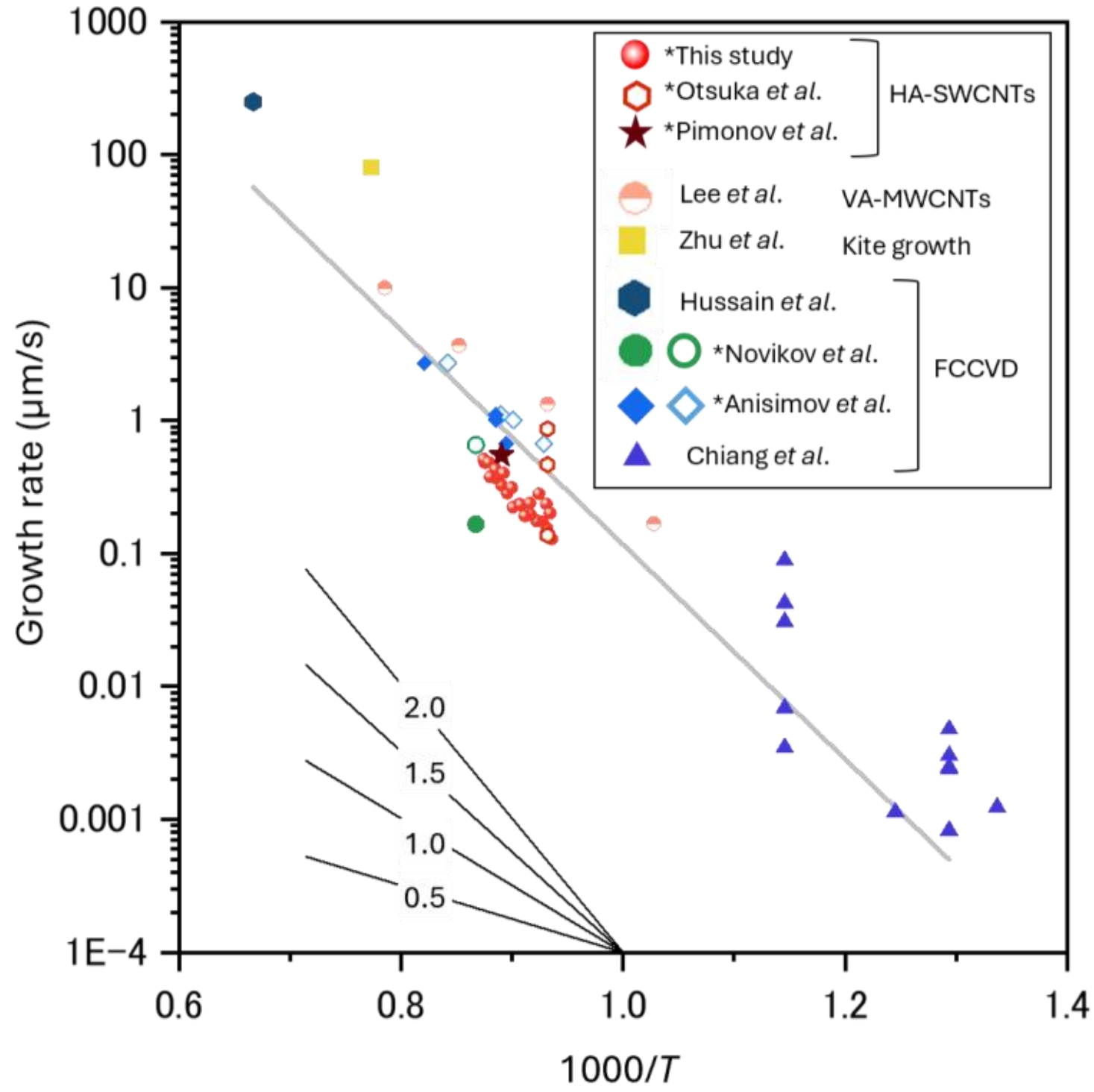

Supplementary Fig 1 | Comparison of reported CNT growth rates across different synthesis methods. Literature denoted with a star (*) reported the growth of single-walled carbon nanotubes (SWCNTs), otherwise multi-walled carbon nanotubes (MWCNTs) were grown. Despite the differences in growth methods, overall growth rates can be fitted with a single slope, suggesting that the observed growth rates fall within a similar kinetic regime. In the study by Otsuka et al.[5] (open hexagons), a small amount of acetylene was gradually added along with a primary carbon source (ethanol), while maintaining the temperature. In the studies by Novikov et al.[9] and Anisimov et al.[10], upper-bound values are also shown in open symbols, because growth rates and temperatures cannot be uniquely determined. In the study by Chiang et al.[8], growth rates were estimated using dynamic mobility analyzer (DMA), instead of directly measuring the length by microscopic means, potentially leading overestimation of growth rates obtained for low-temperature growth, where catalyst size cannot be ignored compared with CNT length.

<u>On Page 7, line 108</u>

We should note that growth rates reported for FCCVD and supported systems fall on a common scaling (Supplementary Fig. 1) despite the differences in configurations, indicating that their kinetics can be compared within a unified framework.

Comment. *Therefore, I do not recommend publication of this manuscript in NC.*

Response. We fully respect the reviewer's evaluation. As clarified above, however, the concerns appear to arise from treating FCCVD and substrate-supported growth as fundamentally distinct

regimes. Our careful survey indicates that these factors do not introduce a qualitative difference in the mechanisms relevant to the present study, and suggests that both systems can be understood within a continuous spectrum of catalyst evolution and growth kinetics. From this perspective, the apparent separation between microscopically resolved growth studies and macroscopic FCCVD systems may reflect a historical gap in experimental accessibility, rather than an intrinsic difference in the underlying physics. We believe that bridging this gap provides a powerful conceptual framework for future studies, particularly in the context of achieving both structural control and scalable production of CNTs. We appreciate the reviewer's comments that highlight the importance of clearly establishing the connection between these different growth systems.

Response to Reviewer #2:

Comment. *The authors investigate the growth of single-walled carbon nanotubes (CNTs) under dynamically evolving environments and trace CNT growth histories using isotope labeling. Based on the observed growth-rate hysteresis induced by temperature variation, the authors conclude that irreversible catalyst coalescence occurs upon increasing the reaction temperature, while the CNT structure remains unchanged throughout the entire growth process. However, the present manuscript does not provide fundamentally new insights into CNT growth mechanisms. The main concerns are outlined below:*

Response. We sincerely thank the reviewer for taking his/her time to review our manuscript and concisely summarizing the key findings of our study. We understand the reviewer's concern, and would like to answer and utilize them to improve our manuscript.

Comment. *1. The authors attempt to generalize their conclusions to both gas-phase growth and substrate-supported processes. However, in typical floating catalyst CVD systems, the actual growth time of an individual CNT is generally shorter than 1 s. As a result, CNT growth may have already terminated before any significant catalyst evolution can be induced by changing the reaction temperature, calling into question the validity of correlating temperature-induced catalyst coalescence with CNT growth behavior in floating catalyst systems.*

Response. We thank the reviewer for raising the important aspect. We believe that this assumption may be too restrictive for FCCVD systems in general. First, sub-second growth or residence times do not appear to be representative across FCCVD systems in general. For example, Weller et al. compiled 32 synthesis papers (55 conditions) on FCCVD/direct-spun systems and found that the bulk residence times span 5–240 s, with a median around 20 s [*Carbon* **146**, 789 (2019)]. We understand that actual catalyst lifetime can be longer or shorter than the residence time in floating-catalyst growth, but the lifetime is in fact much more difficult to determine experimentally. In a recent kinetic study by Novikov et al. [*Carbon* **217**, 118589 (2024)], no clear spontaneous growth termination was observed within the investigated residence-time window of 3.8–12.5 s under CO_2-free conditions, suggesting that catalyst lifetime can exceed the residence-time interval typically accessed in such systems. Taken together, these observations indicate that the reviewer's concern may be relevant to some specific FCCVD configurations, but it does not appear to hold as a general assumption for floating-catalyst CNT growth.

Second, Hoecker et al. [*Carbon* **96**, 116 (2016)] studied an FCCVD reactor operated at furnace set-point temperatures of 1150–1300°C and observed pronounced axial changes in catalyst particle size distribution within the reactor. The temperature range is much higher than the temperature range mainly discussed in our manuscript, and catalyst evolution in the hottest zone is different from our picture (Figure 1). Even in that reactor, however, the feedstock necessarily passes through the 800–900°C range near the furnace entrance, and the local transit time through such a zone is on the order of seconds. Their axial measurements show that catalyst particle populations varied sharply over such short axial intervals, indicating that the reviewer's statement that temperature changes cannot induce significant catalyst evolution is not a general case.

Third, more generally, even a 1-s time scale can be very long from the viewpoint of nanoparticle dynamics. Depending on temperature and the concentration of catalyst precursors (e.g., ferrocene), the characteristic time scale for nanoparticle nucleation, growth, coarsening, or evaporation varies by orders of magnitude. Therefore, the assumption that catalyst evolution must be negligible appears too strong for general FCCVD systems. Rather, when a system is able to grow CNTs to appreciable lengths within a short time, this usually implies high temperature and/or high precursor concentration, conditions that would also be expected to accelerate catalyst formation and evolution.

For these reasons, while the exact growth time in FCCVD is system-dependent and may span a broad range, the assumption that catalyst evolution is absent during the nanotube growth lifetime is not generally warranted. On the contrary, both the literature and physical considerations support the view that catalyst evolution during CNT growth is a plausible and natural scenario. To clarify the above discussion, we have modified the introduction and added a related paragraph in Supplementary Note 11 as follows:

On page 3, line 39
In contrast, in floating-catalyst reactors, catalysts usually nucleate from volatile metal precursors and enlarge predominantly through particle coalescence at relatively low temperatures, while evaporation-driven redistribution also plays a crucial role at higher temperatures[11,12].

Comment. *2. It remains unclear whether the active catalyst particles indeed undergo structural evolution under dynamic reaction conditions. Although AFM characterization was performed on catalysts after CNT removal, this approach cannot distinguish between previously active and inactive particles. Moreover, due to the encapsulation of Fe nanoparticles by CNTs during growth, the active catalysts may be effectively protected from coarsening or diameter changes when the reaction temperature is varied.*

Response. We thank the reviewer for the important comments. We believe that the following additional experiments and discussion sufficiently address the reviewer's concerns. First, our AFM characterization (Fig. 4c) clearly shows the decrease in the number of catalysts at 850°C, which suggests the significant migration of iron atoms on the quartz surface. Regardless of the state of other catalysts, such migrating atoms should be merged into surviving catalysts (*i.e.*, if carbon atoms are supplied to catalysts, iron atoms should also be supplied). Second, we have checked the typical size of active catalysts for CNT growth (Supplementary Fig. 9). For this purpose, we sparsely deposited iron catalysts on a quartz substrate, followed by CNT growth at 800°C. Here, we performed AFM characterization without further treatments, though minimal changes, such as carbon precipitation or oxidation, may occur during cooling and exposure to air. This observation indicates that the active catalysts are much larger than the corresponding tube diameter, clearly excluding the possibility of tangential growth mode (or encapsulation of Fe nanoparticles by CNTs). We have added Supplementary Note 9 and Supplementary Fig. 9 to address this point.

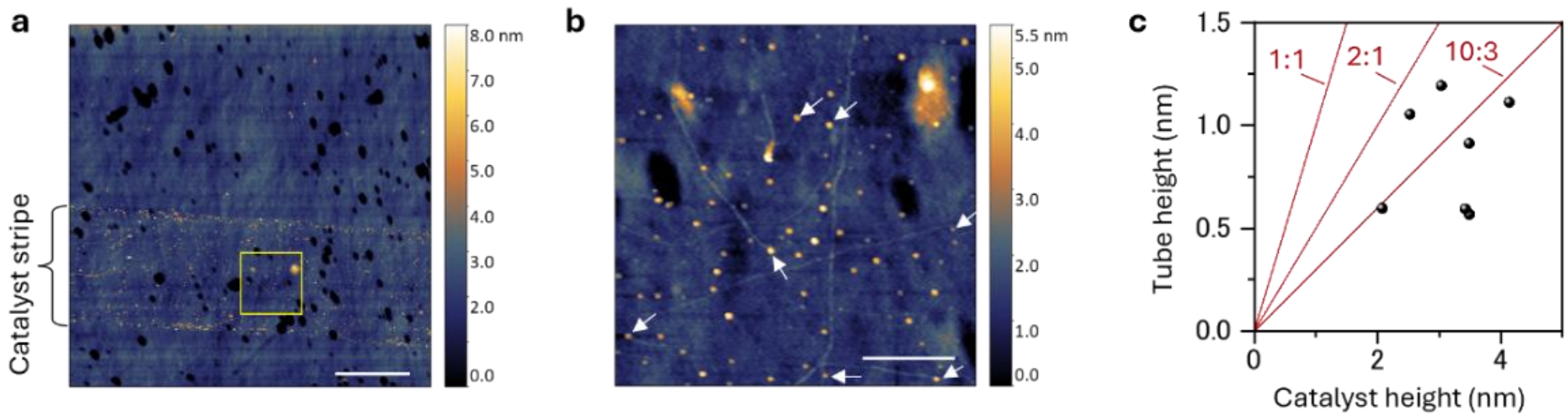

Supplementary Fig. 9 | AFM characterization on quartz. a, b AFM images of catalyst nanoparticles and CNTs grown at 800°C. Panel b was measured within the catalyst stripe (yellow box) in a. Scale bars are 2 μm and 500 nm in panels a and b, respectively. **c** Correlation of the heights of catalyst nanoparticles and the CNTs that grew from the catalysts. Solid lines represent the 1:1, 2:1, and 10:3 catalyst-tube height ratios.

Comment. *3. In addition, several research groups have conducted extensive studies on catalyst evolution during CNT growth using environmental transmission electron microscopy. In their works, significant catalyst coarsening during active CNT growth has rarely been observed, which appears inconsistent with the conclusions drawn in the present manuscript.*

Response. We thank the reviewer for the comments on environmental TEM studies. We have carefully reviewed a number of in situ and ex situ observations of active catalysts during CNT growth, and found that several studies showed a clear evolution (both coarsening and disappearance) of active catalysts. For example, Navas et al. [*ACS Nano* **11**, 3081 (2017)] clearly show the Ostwald ripening, including disappearance, of active catalysts through ex situ TEM studies of different growth conditions (time, temperature, etc.), while in situ study by Kim et al. [*JPCL* **1**, 918 (2010)] stated "Figure 4 shows four frames from one of these videos that captures growth, termination, and Ostwald ripening or subsequent subsurface diffusion of a catalyst particle growing a few-walled CNT". In addition, unless a specific mechanism exists that prevents surface-diffusing atoms from being absorbed by active catalyst particles, the presence of shrinking particles necessarily implies coarsening of others. To share the relevant previous studies, we have rephrased a sentence and added a reference to Kim et al. in the introduction as follows.

On Page 5, line 81

Although *in situ* TEM can probe such dynamic changes of catalyst nanoparticles actively growing nanotubes[35-38], its intrinsic constraints, including the requirement for extremely low pressure and electron-beam-induced damage, render it incompatible with the growth of well-crystallized CNTs under practical conditions.

35. Kim, S. M. *et al.* Evolution in Catalyst Morphology Leads to Carbon Nanotube Growth Termination. *J. Phys. Chem. Lett.* **1**, 918–922 (2010).

Comment. *4. Another concern relates to the temperature change protocol employed in this work. Although the furnace set temperature is adjusted during the experiment, a finite thermal response time is required for the temperature change to be effectively transmitted to the catalyst*

nanoparticles. Consequently, the actual reaction time experienced by the catalyst at each temperature is difficult to define accurately. This uncertainty complicates the precise determination of CNT growth rates and may undermine the reliability of the growth-rate analysis under dynamic temperature conditions.

Response. We sincerely appreciate the insightful comment on the temperature protocol employed in our study. To evaluate the potential discrepancy between the furnace set temperature and the actual temperature experienced by the catalyst, we constructed a simplified heat-transfer model to estimate the *upper bound* of the thermal lag (conservative case). The analysis indicates that the temperature deviation is limited to within a few kelvin and that the characteristic response time is on the order of a few seconds, which is much shorter than both the temperature modulation period and the isotope-labeling interval. In our experiments, silicon substrates that support quartz substrates (see Supplementary Fig. 5a) enhance the absorption of radiation from the furnace, leading to quick thermal response. Furthermore, incorporating this thermal lag into the Arrhenius analysis results in only a minor shift without altering the extracted activation energy or the observed hysteresis. These results confirm that the key conclusions of this study are not affected by the finite thermal response. The detailed analysis has been added as Supplementary Note 5 and Supplementary Fig. 5.

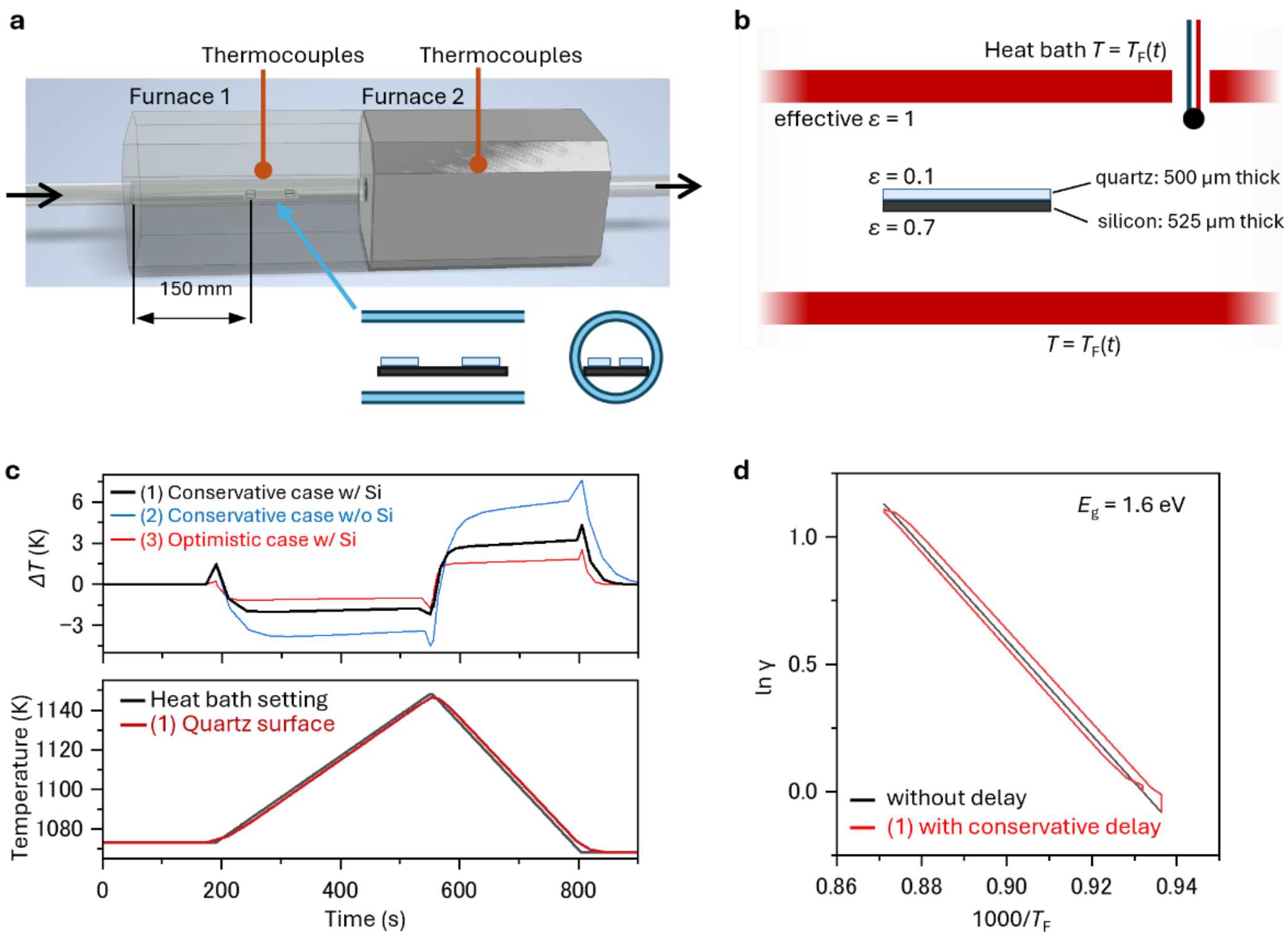

Supplementary Fig. 5 | Thermal response of the substrate under programmed furnace temperature. a Schematic of the two-zone furnace setup and substrate placement inside the quartz tube. **b** Simplified thermal model describing radiative and gas-mediated heat transfer between the furnace and a quartz/Si substrate stack. **c** Simulated temperature deviation (top) between the furnace setpoint and the quartz surface (bottom) for three different assumptions

on heat transfer. **d** The corresponding Arrhenius plots comparing cases with and without thermal delay.

Comment. *5. The authors' claim regarding the decoupling of structural memory and growth rate does not represent a new concept within the CNT synthesis community. CNT growth is commonly understood to consist of distinct nucleation and growth stages, during which the CNT structure is primarily determined during nucleation, while the subsequent growth rate can vary without altering the established structure. Moreover, substantial progress has already been made in achieving structure-controlled CNT growth through the regulation of nucleation processes or growth kinetics, as reported by Yan Li's group in Peking University. As such, the novelty and significance of the authors' conclusions in this regard are limited.*

Response. We appreciate the comment on the relevance of chirality-selective growth studies. We would like to respond to this comment from three points of view listed below. **First**, we completely agree that the general distinction between nucleation and growth stages has been discussed in the CNT community. However, as described in the introduction, there have also been many studies on chirality change upon intended perturbation (e.g., temperature) by both experimental and theoretical approaches, which then leads to the expectation that chirality should vary within the same CNTs in FCCVD processes. If it is the case, despite its high productivity, FCCVD cannot be used to produce chirality-controlled CNTs. In this context, the present work does not aim to propose a new conceptual separation between nucleation and growth. Rather, as reflected in the manuscript title ("growing under evolving environments"), its contribution is to experimentally examine whether the same structural robustness persists under dynamically evolving growth conditions, where catalyst size and temperature change significantly during elongation. This study provides direct evidence for structural robustness beyond steady-growth regimes.

Second, although Yan Li et al. reported a significant breakthrough in the research community (such as *Nature* **510**, 522 (2014)), this does not mean growth processes are fully understood or sufficiently controlled in a large quantity. While their study relies on the chirality determination from the viewpoint of equilibrium thermodynamics (*i.e.*, which structure is most stable after infinitely long time and trial), our study rather focuses on dynamic aspects of structure integrity from a kinetic viewpoint, playing a complementary role in understanding CNT growth. In addition, as Prof. Li pointed out, the use of high-melting-point catalysts achieves structural control at the cost of growth rate (i.e., low yield). As a possible approach for overcoming such a tradeoff relationship, we propose the decoupling of the following elongation processes regardless of how initial structures are established in a controlled manner.

Third, the relationship between such a framework proposed by Li's group and the present observations requires very careful consideration. It is commonly understood that chirality is effectively determined during cap formation, when a sixth pentagon is incorporated [*PRB* **72**, 165423 (2005), *Chem. Sci.* **9**, 3056 (2018)]. At this stage, however, the extended sidewall structure characteristic of a given (n,m) nanotube is not yet fully developed, and the concept of a well-defined chirality-dependent interface is not necessarily straightforward. If chirality is indeed

robust once established—as has been widely observed under steady growth conditions—then the role of tube-catalyst interface thermodynamics in determining chirality becomes less dominant. Instead, if chirality were allowed to change frequently during growth, one could anticipate a scenario in which nanotubes statistically evolve toward thermodynamically favored interface configurations as discussed in [*Sci. Adv.* **2**, e1501729 (2016), *Chem. Sci.* **9**, 3056 (2019)]. Reconciling these two pictures would require additional unverified assumptions, such as enhanced structural flexibility only at the very early stage of cap formation, including lattice-defect formation and backward reaction, or a short lifetime of energetically unfavorable configurations. We have revised the manuscript to clarify this point and to better distinguish our contribution from prior studies on chirality-selective synthesis.

On page 23, line 455

In light of the observed structural robustness, it is also useful to revisit how the thermodynamic stability of the CNT–catalyst interface has been interpreted in relation to chirality-preferential growth. It has been recognized that chirality is effectively determined during cap formation, when six pentagons are formed[63,64]. At this stage, (n,m)-specific sidewall structures are not yet present and therefore may not directly account for chirality determination. Yet, CNTs may approach thermodynamically favored configurations, including diameter and chirality matching with the catalyst, if sufficient opportunities for structural reconfiguration are provided within their growth lifetime. The present results, however, suggest a contrasting picture. Further investigation is required to clarify how this structural robustness depends on catalyst composition and state (*e.g.*, liquid or solid) and how it reconciles with previously proposed thermodynamic perspectives for chirality-specific growth[4,13,65].

Comment. *6. Moreover, chirality changes during CNT growth are generally considered highly unlikely. According to theoretical studies (for example, Chem, 5, 1-12 (2009)), chirality mutations require the incorporation of pentagon-heptagon defect pairs into the CNT sidewall. The associated energy barriers for such processes are extremely high (larger than 3 eV), which likely accounts for the robust preservation of CNT chirality even under dynamically changing reaction environments. Consequently, the observed retention of CNT structure in this work is consistent with established theoretical understanding rather than providing new mechanistic insight.*

Response. We sincerely appreciate the important comment regarding the energy barriers for pentagon-heptagon defect pair formation. Unfortunately, we were not able to identify the specific reference [*Chem* **5**, 1-12 (2009)] despite intensive searching including relevant journals (*Chem* was first published in 2016). Instead, we interpret the reviewer's point by referring previous theoretical studies on defect formation barriers, such as Yuan et al. [*PRL* **108**, 245505, 2012], and have revised the manuscript accordingly. In Yuan *et al.*, the activation barrier for the 5–7 defect formation (not 'formation energy') via the reverse process of defect healing exceeds 4 eV (Fig. 3, Yuan:2012), indicating that the transformation of an already formed 6–6 structure into a 5–7 defect is highly unlikely, seemingly consistent with the reviewer's point. However, during carbon incorporation at the growth interface, alternative pathways with significantly lower barriers are available. Accordingly, they considered a finite fraction (~10%) of 5–7 defects at the growth front

in their calculations to reach their claim (Fig. 4, Yuan:2012). These results highlight that defect formation is strongly pathway-dependent and governed by kinetics, rather than being determined solely by thermal equilibrium such as $\exp(-E/kT)$. We have revised the Introduction to explicitly distinguish between thermodynamic and kinetic contributions in this context. To reflect the intended message in the manuscript more precisely, it would be highly appreciated if the reviewer could provide us with more details (e.g., DOI) on the theoretical studies on defect formation.

On Page 4, line 62
From an equilibrium viewpoint, the density of topological defects is expected to be extremely low under typical growth temperatures, theoretically supporting the growth of macroscopically long CNTs[24]. Even when non-equilibrium kinetic processes are considered, chirality preservation over hundreds of micrometers is also well reported for high-quality CNTs grown under steady conditions[24-27] and is further supported by MD simulations in terms of defect-healing kinetics through the kinetic modeling of defect-healing processes[28,29].

In addition, we have performed molecular dynamics (MD) simulations regarding defect formation kinetics during catalytic CNT growth (Supplementary Fig. 17). When starting with a partially embedded pentagon defect near the tube-catalyst interface and changing the supply rate of carbon atoms to the system, the probability of incorporating this defect in the sidewall highly depends on the supply rate. These results independently indicate that the defect formation is kinetically dominated, beyond the simple equilibrium description using energy barrier and temperature.

We should also note that the study by Yuan et al. considered a model without curvature in CNTs and catalysts, and cannot be directly extended to the chirality robustness under dynamically changing reaction environments. To fill this gap, the same group led by Prof. Ding later reported the effect of size differences between CNTs and catalysts, observing the chirality change events in MD simulations [*Carbon* **175**, 69 (2021)]. In contrast, our MD simulations in Supplementary Fig. 18, where "artificial" CNTs are attached to catalyst particles much larger than the CNT, exhibit defect-free tube elongation. This contradictory result can be attributed to the accuracy of force fields; we used machine-learning force fields developed by Hedman et al., which described Fe-C systems more accurately than other previously reported force fields.

The above finding further led to another important simulation (Fig. 5e,f), where an artificial CNT is attached to a size-matching catalyst particle. Here, iron atoms are added at 1-ns intervals to emulate catalyst coarsening, whreas carbon atoms are supplied at a rate proportional to the surface area of a catalyst particle. Again, the CNT retains its chirality and elongates at a rate reflecting the catalyst coarsening, providing support both for structural memory and adaptive kinetics. We have added the following paragraph to the main text.

On page 19, line 375
As a complementary approach, we performed MD simulations using a machine learning force field (DeepCNT-22[28,50]), in which a CNT with a fixed chirality (8,7) is grown from an iron catalyst particle (initially Fe_{120}) whose size is allowed to evolve during elongation (Fig. 5e,f). In this model, Fe atoms are incrementally added to the catalyst, while carbon atoms are supplied to the catalyst interior at a rate proportional to the catalyst surface area, with unstable carbon atoms being stochastically removed. As a result, the nanotube chirality remains unchanged

throughout the simulation, while the catalyst particle grows in size over time. The time evolution of the system (Fig. 5f) shows that the number of carbon atoms incorporated into the CNT (black line) increases in a convex manner, indicating an acceleration of the growth rate as the catalyst grows in size as assumed earlier. These results provide a mechanistic support that, even under catalyst coarsening, the nanotube diameter does not follow the evolving catalyst size but robustly retains the initially established structure, which is consistent with the experimental observations.

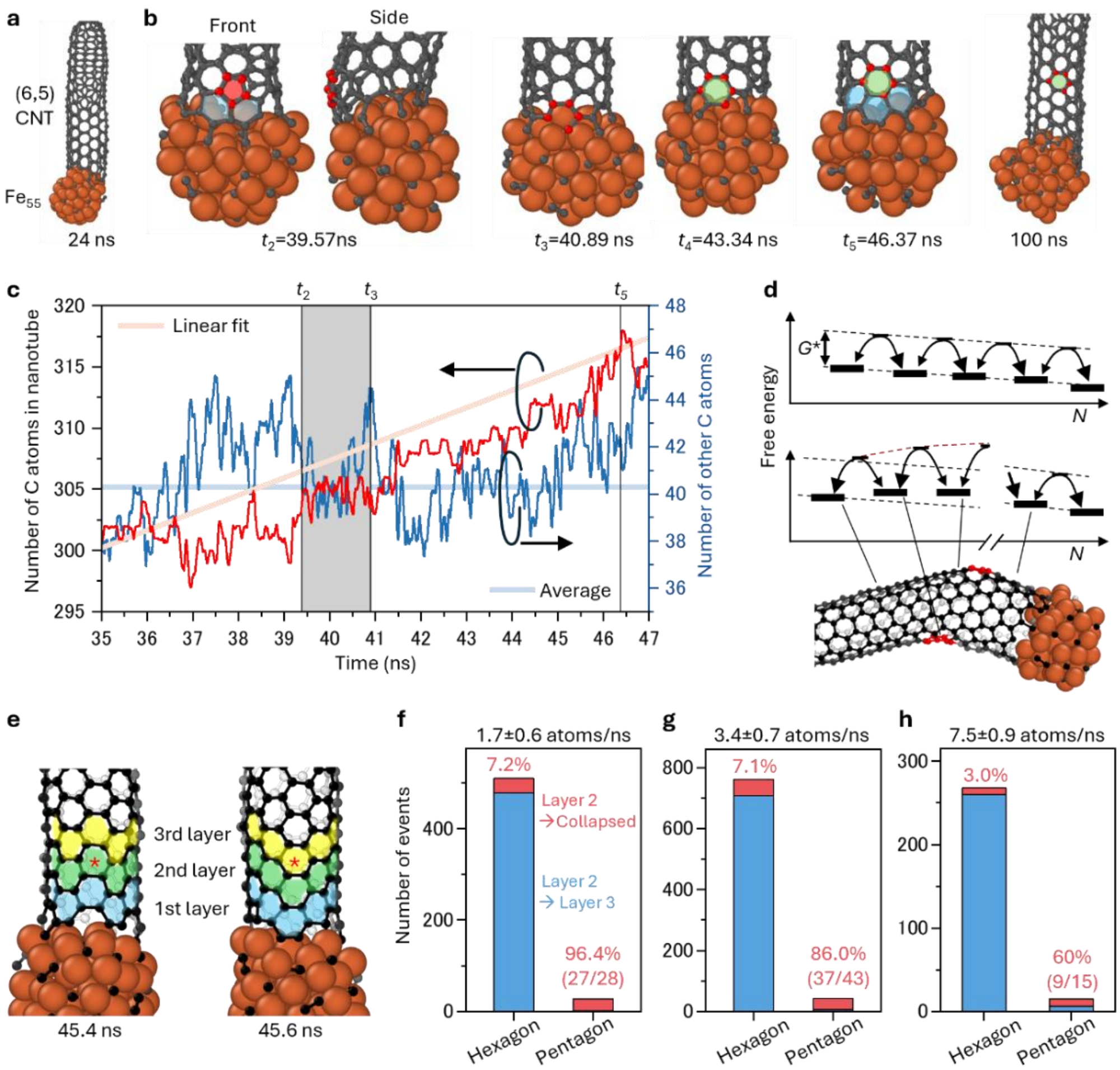

Supplementary Fig. 17 | Kinetic filtering of non-hexagonal defects at the tube–catalyst interface in MD simulations. a Snapshot of a CNT grown from an Fe_{55} catalyst. **b** Front and side views of a representative event in which a pentagon (red) forms transiently near the tube edge and is subsequently removed through local reconstruction; additional hexagons formed during the event are highlighted in green and blue. **c** Time evolution of the number of carbon atoms incorporated into the nanotube (red) and other carbon atoms in the system (blue), showing that net elongation is suppressed while a pentagon persists (gray background). **d** Schematic free-energy landscape illustrating normal growth (top) and a defect-including pathway, where forward propagation is hindered and reversal becomes favorable. **e** Definition of layer depth from the tube–catalyst interface used for ring-tracking analysis. **f–h** Statistical

outcomes for rings that reached layer 2 under different carbon supply rates. To increase the number of pentagon events, simulations were restarted using the same initial configuration that contains a pentagon in layer 2, with 15, 20, and 10 repeated trajectories using different random seeds.

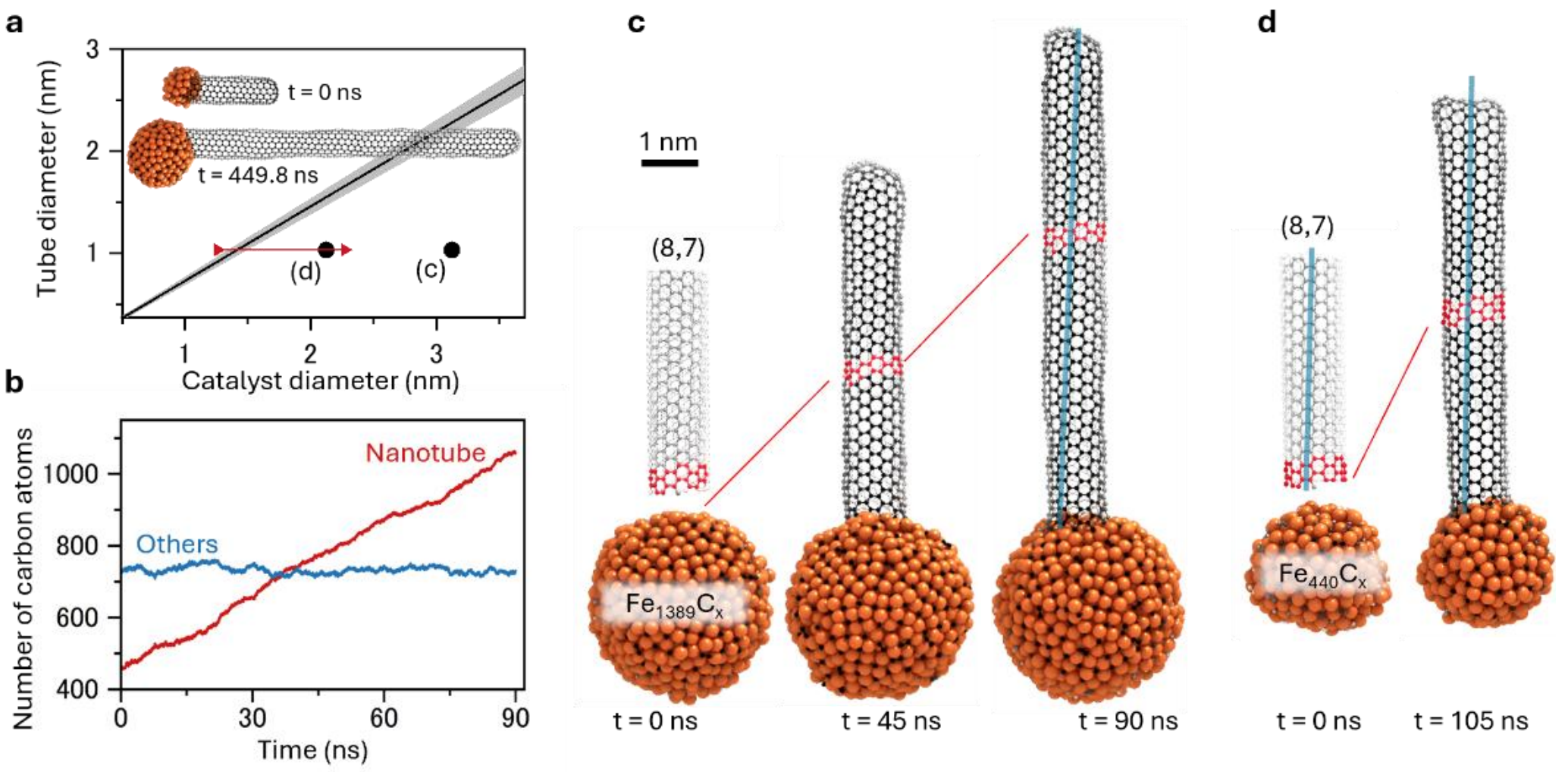

Supplementary Fig. 18 | Growth of a small-diameter CNT attached to an oversized catalyst particle. a CNT-catalyst size relationship obtained from spontaneous nucleation (black line) and that of prepared configuration. The present configuration lies outside the typical range (gray region). Red triangles show catalyst-tube size relationships for the CNT growth simulation shown in the inset, which is extended from the result shown in Fig. 5. **b** Time evolution of carbon atoms in the CNT (red) and in other forms (blue). **c**,**d** Snapshots of (8,7) CNTs grown from large catalysts (Fe_{1389} and Fe_{440}), showing chirality preservation despite the size mismatch between CNTs and catalysts. Temperature was kept at 1400 K.

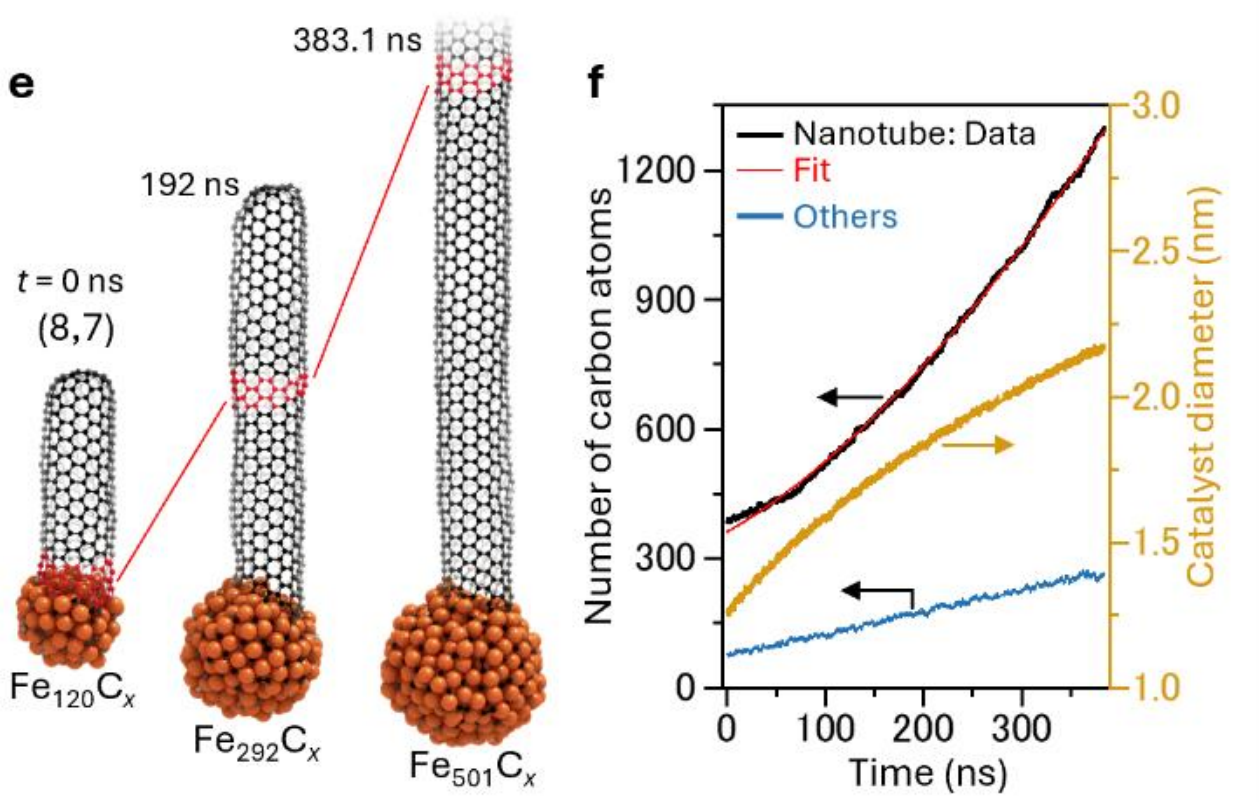

Fig. 5 | Robust chirality preservation under evolving environments. e Snapshots of the growth trajectories in MD simulation for a (8,7) CNT growing from an iron nanoparticle that gradually grows in size. Atoms in red represent the hexagons located at the tube edge at *t* = 0. **f** Time evolution of carbon atoms incorporated into the CNT (black), non-CNT carbon atoms (blue), and catalyst diameter, showing increasing catalyst size and accelerated growth while the CNT structure remains fixed. Red line is a fitting result, which assumes the growth rate proportional to the catalyst surface area.

Comment. *7. Some minor comments: i) It is generally not reliable to distinguish metallic and semiconducting CNTs solely based on the G-band features, as the G mode can be influenced by multiple factors and does not provide unambiguous electronic-type assignment. ii) Line 63: the phrase "HiPco-derived samples have revealed" appears to be incorrect and should be revised to "HiPco-derived samples have not revealed".*

Response. We thank the reviewer for further comments. As for (i), we agree that, in general, G-mode lineshapes alone do not always provide an unambiguous assignment of metallic and semiconducting CNTs, especially on as-grown substrates where local strain, doping, or nanotube–substrate interactions may modify the line shape [*ACS Nano* **20**, 7794 (2026)]. However, our assignment is not based on isolated spectra, but based on spatially continuous Raman mapping along individual nanotubes, typically over >100 positions per tube. In the cases discussed here, metallic-like G-mode features persist over entire segments spanning tens of micrometers, or appear abruptly and then remain spatially consistent over long distances when the chirality changes. Such behavior cannot be explained by random local perturbations. In addition, the nanotubes were transferred from the growth substrate to a Si/SiO_2 substrate for observation, which mostly relieves the growth-induced substrate interaction and residual stress. Together with the consistency between RBM-confirmed metallic spectra and G-mode-only metallic assignments (revised version of Supplementary Fig. 2f), these features make misassignment by random local perturbations unlikely in the present experiments.

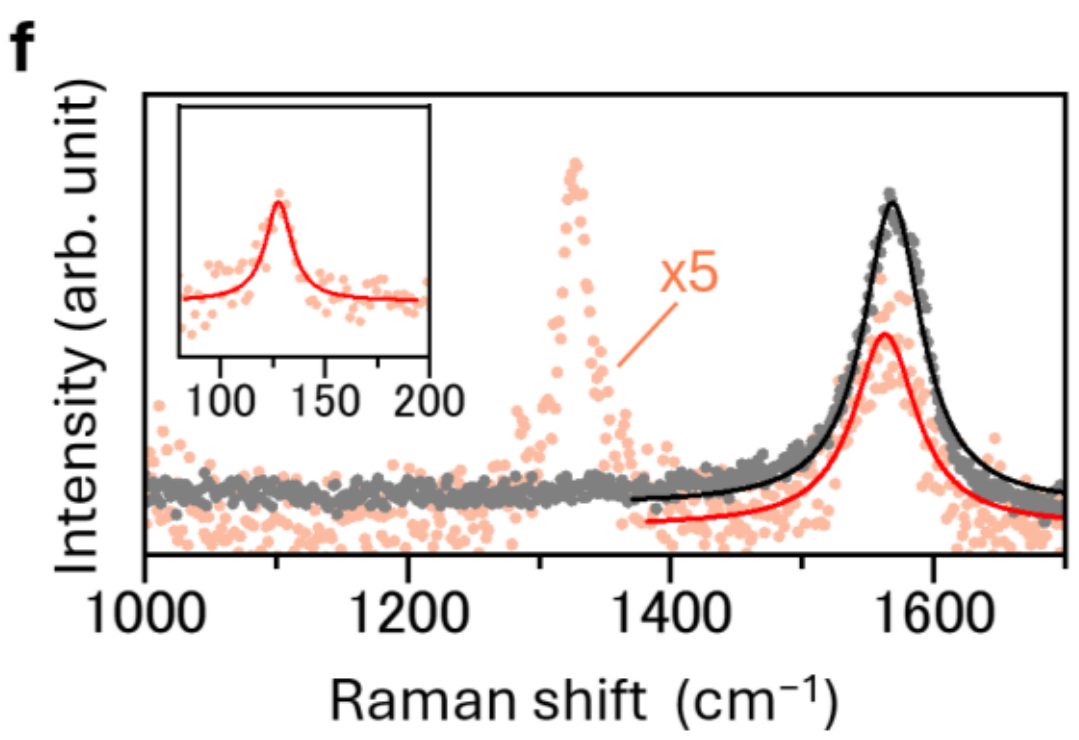

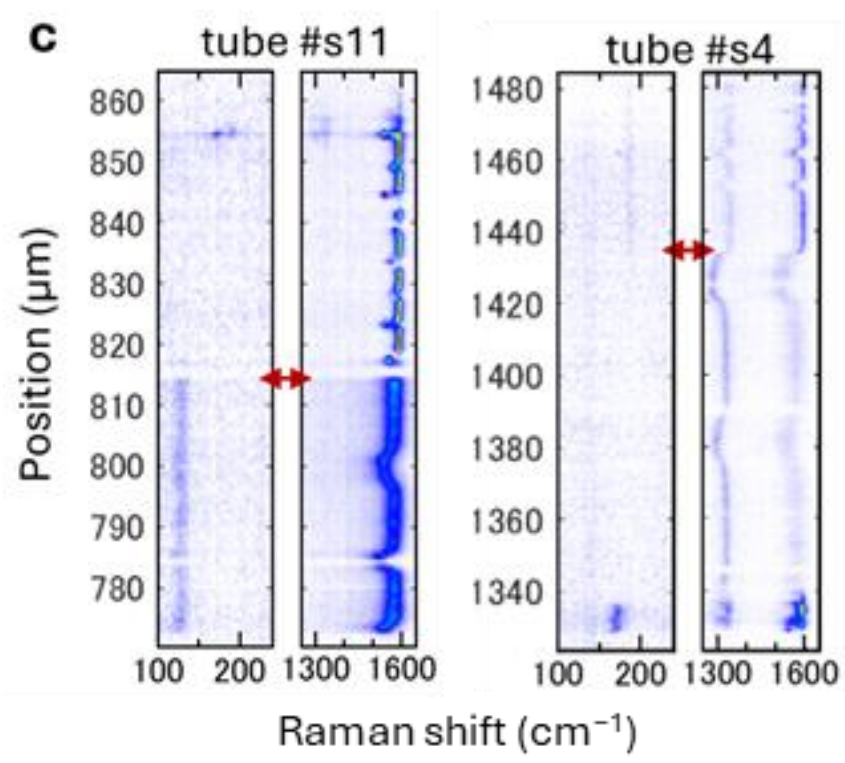

Left: **Supplementary Fig. 2 | f** Raman spectra obtained from a metallic CNT (light red, tube #s11 in Supplementary Fig. 10), which shows a RBM peak at 127 cm^{-1} (diameter of ~2.03 nm), from a metallic-like CNT (gray, tube #s4 in Supplementary Fig. 10) judged from G-mode shape. These spectra are averaged over 4.2 (7 points) and 9 μm (15 points) along their axis, respectively.

Right: **Supplementary Fig. 10**

Regarding the point (ii), we have revised the sentence according to the reviewer's suggestion.

On page 4, line 60
However, HiPco-derived samples have not exhibited clear evidence of intratubular junctions, despite extensive characterization by photoluminescence spectroscopy[21,22] and transistor measurements[23].

Response to Reviewer #3:

Comment. *In this manuscript, the authors report growth-kinetics measurements of individual single-walled carbon nanotubes (SWCNTs) under a temperature gradient, using an isotope-encoding method. They reveal a clear hysteresis in both the average lifetime and the growth rate (at the ensemble level as well as for individual nanotubes) between temperature ramp-up and ramp-down, manifested by distinct activation energies in the two regimes.*

To rationalize these observations, the authors propose a model based on two key assumptions:

(i) under the experimental conditions employed, the CNT growth rate increases with increasing catalyst particle size (i.e. surface area);

(ii) the catalyst particle size increases with temperature as a result of Ostwald ripening.

The calculated trends are compared with the experimental data and show good qualitative agreement. An even better agreement is obtained when considering only large individual catalyst nanoparticles, (although this aspect is described with less precision than the rest of the analysis). The authors further observe that, while the CNT growth rate systematically varies with temperature, the nanotube chirality remains unchanged in most cases. This finding contrasts with previous reports (e.g. Yao et al., 2007).

From these results, the authors conclude that chirality selection occurs at the nucleation stage, whereas the nanotube length is determined during the elongation stage. This decoupling is proposed to explain the relative insensitivity of the diameter distribution to furnace temperature in floating-catalyst CVD growth.

Overall, I am convinced by the experimental methodology, the data interpretation, the modeling approach, the quality and relevance of the arguments, and the main conclusions. This manuscript offers a rigorous and scientifically robust analysis of CNT growth mechanisms, which is highly appreciated.

As detailed below, I have minor disagreements with the interpretation of the Raman data used to assess the degree of chirality preservation (which do not affect the main claims of the authors), as well as many requests for additional information, clarification, and discussion. These points are intended to further strengthen the manuscript and make its conclusions more robust and convincing to a broad readership.

Response. We sincerely thank the reviewer for professional evaluation and suggestions for improvement, and are very delighted to hear that the reviewer is convinced by the experimental methodology, the data interpretation, the modeling approach, the quality and relevance of the arguments, and the main conclusions. We have revised the manuscript and improved the clarity and discussion according to the insightful suggestions.

Main points:

Comment. *1. « growth rate scales with the catalyst surface area » (Otsuka et al., p. 13). The authors should explain what would be the corresponding kinetic regime in the frame of their kinetic model in "Universal Map..." (ACS Nano 2022, 16, 5627−5635) and whether it would only be dependent of the catalyst surface area or also on the CNT chirality.*

Response. We are grateful to the reviewer for referring to our previous work, which provides a basis of the assumption employed in this study. According to the kinetic model, the sensitivity of growth rate to the tube chirality and the catalyst surface can be described as

$$S_{\mathrm{g}} = \frac{\partial \ln \gamma}{\partial \ln k_{\mathrm{g}}} = \frac{Ak_{\mathrm{de}}P_{\mathrm{E}}}{k_{\mathrm{g}}D + Ak_{\mathrm{de}}P_{\mathrm{E}}},$$

$$S_{\mathrm{A}} = \frac{\partial \ln \gamma}{\partial \ln A} = \frac{k_{\mathrm{g}}D}{k_{\mathrm{g}}D + Ak_{\mathrm{de}}P_{\mathrm{E}}},$$

respectively, and we can see $S_{\mathrm{g}}+S_{\mathrm{A}} = 1$. As CNT growth occurs in low-pressure environments, where carbon-source and etching-agent concentrations are low (small P_{E}), corresponding to "randomly dispersed regime", S_{A} is closed to 1, while $S_{\mathrm{g}} \sim 0$. This indicates that the growth rate is nearly proportional to the catalyst surface area, but insensitive to the tube chirality (chiral angle). Numerical results reflecting the present growth conditions are also shown in Supplementary Fig. 7a. This analysis, in turn, suggests that catalyst size has a minor influence on growth rate, if the pressure is high enough (large P_{C} and P_{E}) as in atmospheric-pressure ACCVD, leading to the steady growth tolerant to catalyst coarsening. We have modified the following sentence and Supplementary Note 7 to clarify this point.

> On page 12, line 227
> This behavior can be rationalized if the catalyst size evolves during growth (Fig. 4b), with the elongation rate nearly scaling with catalyst surface area for the weak etching regime at a low pressure[32,43] (see also Supplementary Fig. 7).

Comment. *2. Linear versus non-linear growth: If the proposed scenario is correct, one would also expect to observe nanotubes displaying an increase in growth rate even under constant growth conditions (eg in conditions where the characteristic time of Ostwald ripening is close to the CNT growth lifetime). The authors should comment on whether they observed such cases.*

Response. We appreciate this important comment and agree that growth rate should be changing even under constant growth conditions. Although the results have not been published, a former student in our group has examined the growth profiles at 850°C using both Fe and Co catalysts (Shun Yamamoto, Master thesis at the University of Tokyo (2019)). Gradual increase in growth rates has been observed for some CNTs, while others exhibited constant growth rates (Figure R1), in contrast with the case at 800°C [*ACS Nano* **12**, 3994 (2018)]. If we carefully examine the data obtained for a constant 800°C condition, some CNTs slightly show non-linear growth behavior (acceleration) as shown in nearly added Supplementary Fig. 6f, Relative growth rate change within the same CNTs are analyzed and averaged across all the analyzed CNTs (panel g), we find that the growth rate was increased by 4.6%/min. Although majority of catalysts should shrink

during Ostwald ripening, the observed overall acceleration is consistent with the AFM observations showing that CNTs were grown from relatively large catalyst particles. <u>We have added new panels in Supplementary Fig. 9.</u>

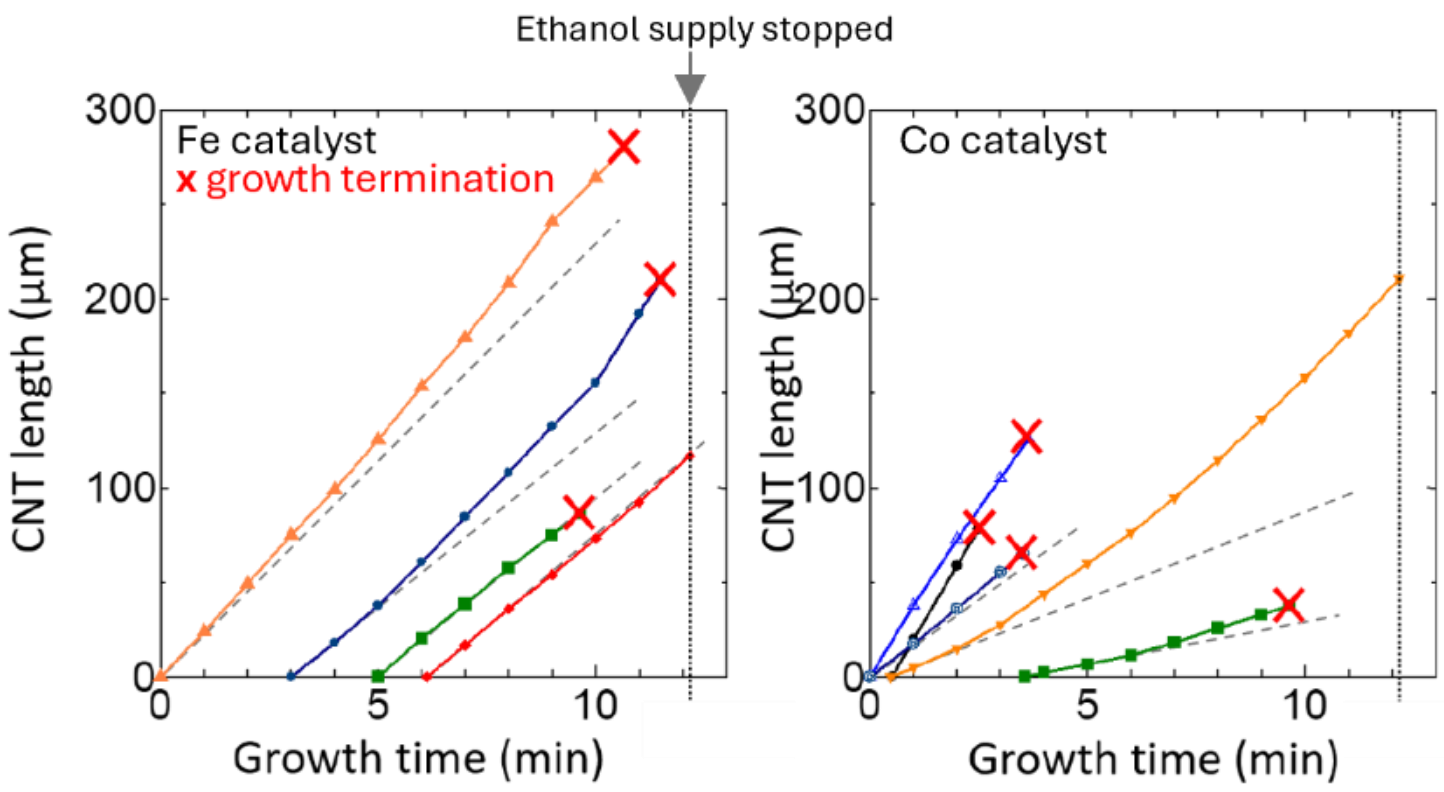

Figure R1 | Representative time evolution of CNT lengths grown from Fe (left) and Co (right) catalysts at 850°C. Dashed lines indicate the extrapolation of initial growth rates, and crosses represent the growth termination before stopping ethanol supply at 12 min. Data are adopted from unpublished work (Shun Yamamoto, Master thesis at the University of Tokyo, 2019).

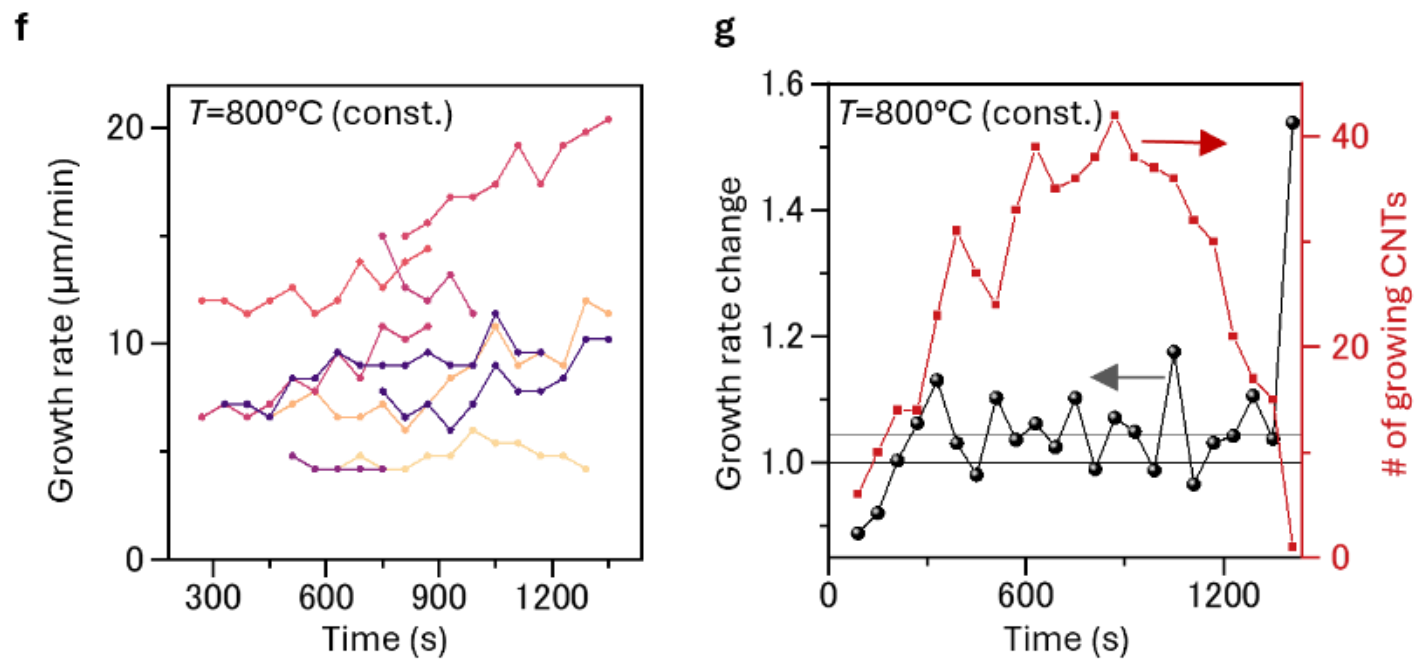

Supplementary Fig. 6 | f Growth rate evolution of representative CNTs grown under a constant temperature[15]. **g** Ensemble average of growth rate change per one minute within the same CNTs[15].

Comment. *3. The authors should discuss whether their results agree with the mechanisms previously proposed to account for the influence of growth parameters on the CNT diameter distribution (eg Lu et al (J. Phys. Chem. B 2006, 110, 41, 20254–20257), Navas et al (ACS Nano 2017, 11, 3, 3081–3088).*

Response. We sincerely thank the reviewer for suggesting highly related papers and fully agree that these studies provide an informative description in which the nanotube diameter distribution reflects the size distribution of active catalyst particles dynamically reshaped by Ostwald ripening. In this sense, our interpretation is fully consistent with the underlying picture proposed in these studies, especially with the study by Navas et al. Importantly, those studies primarily focused on the evolution of diameter distributions and did not explicitly address whether the chirality of

individual nanotubes is preserved once growth is initiated. While the assumption of chirality preservation was reasonable, it has not been directly verified at the level of individual nanotubes. In our study, by tracking the growth histories of individual CNTs, we explicitly demonstrate that the chirality (and thus diameter) selected at nucleation is robustly preserved, even under dynamically evolving conditions where catalyst particles undergo substantial coarsening. Therefore, our results provide direct experimental support for the underlying assumption of these frameworks and clarify that catalyst size distribution at the nucleation stage governs the CNT diameter. We have added a short discussion on the relevance of the study by Navas et al.

On page 22, line 433
This view is also consistent with the framework of Navas *et al*., where the diameter distribution of growing nanotubes reflects the active catalyst size distribution as Ostwald ripening continuously reshapes the catalyst population under a given carbon feeding condition[57].

Comment. *4. Additional AFM data (other samples, other regions) would be welcome to confirm the generality of the Ostwald ripening observation that is key for the main claim: especially in the case of 800-873-800 experiments to demonstrate that the (short) stay at high temperature was sufficient to cause significant Ostwald ripening.*

According to the suggestion by the reviewer, we have measured two different types of AFM data for CNTs on quartz, where no post-growth treatment was performed to retain the original configuration. Note that during the transfer of CNT arrays onto Si/SiO_2 substrates, catalyst nanoparticles were not accompanied and absent in the analyzed samples (Supplementary Fig. 2). First, we grew CNTs at 800°C from sparsely deposited iron catalysts within stripe patterns, and then performed AFM measurements in the catalyst regions. We find the height of catalysts are much larger than that of corresponding CNTs. To further correlate the catalyst size with growth profiles reconstructed from isotope decoding, we synthesized CNTs under temperature ramp up (800–875°C in 5 min) and acquired AFM images at tube-catalyst connection regions, showing the catalyst-tube diameter ratio to be ~2 the CNT with an apparent E_a of 2.3 eV. Probably because the thin iron was deposited for the correlation of catalysts and tubes, Ostwald ripening may be suppressed compared to the experiment shown in the main text, which explains E_a lower than the average (3.2 eV) value in Fig. 3e for T ramp-up. Although these results provide limited support for Ostwald ripening during short stay at high temperature, we have added Supplementary Fig. 9.

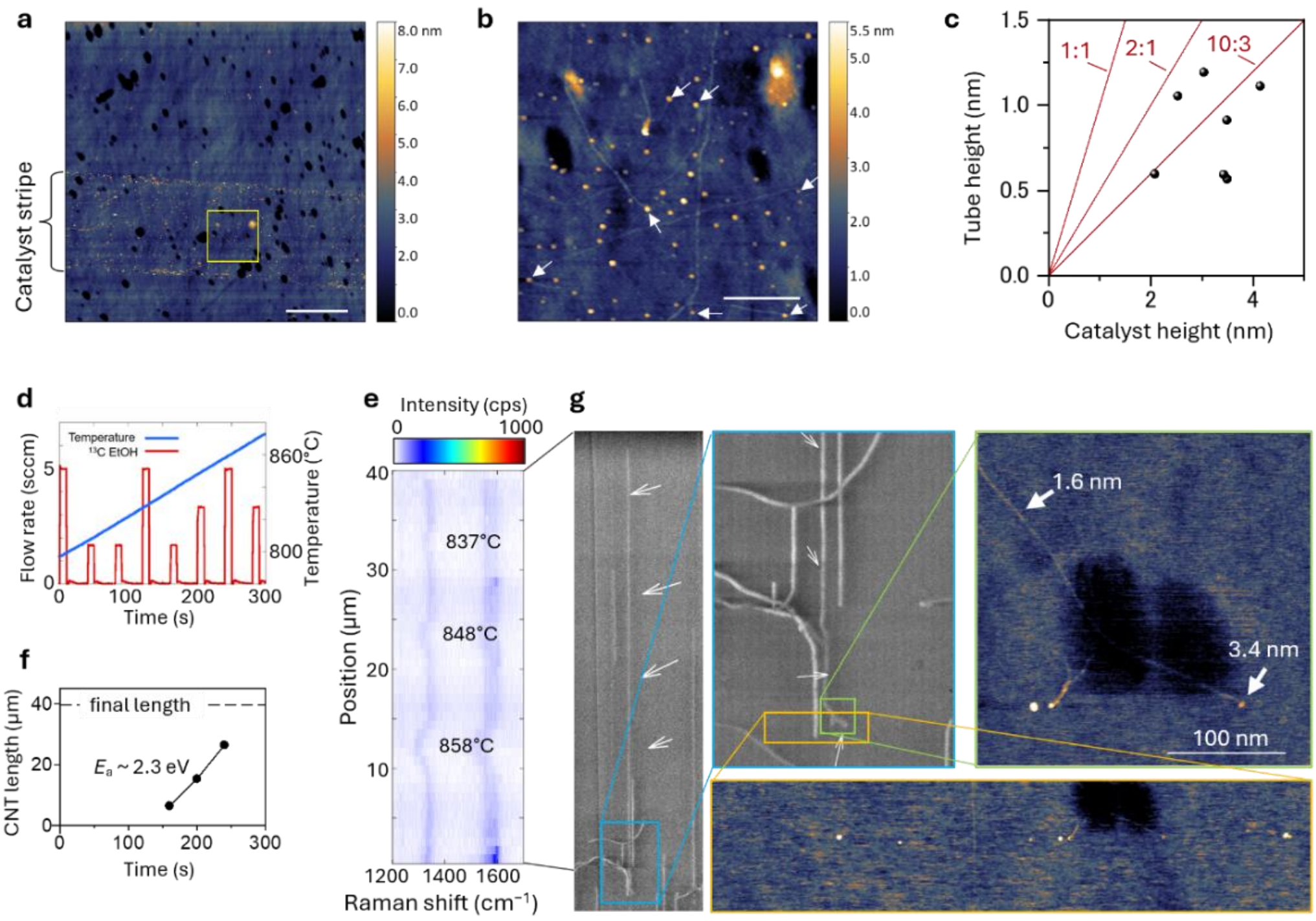

Supplementary Fig. 9 | AFM characterization on quartz. a, b AFM images of catalyst nanoparticles and CNTs grown at 800°C. Panel b was measured within the catalyst stripe (yellow box) in a. Scale bars are 2 μm and 500 nm in panels a and b, respectively. **c** Correlation of the heights of catalyst nanoparticles and the CNTs that grew from the catalysts. Solid lines represent the 1:1, 2:1, and 10:3 catalyst-tube height ratios. **d** Temperature and isotope labeling pattern for the growth analysis on a quartz substrate. **e** Raman spectra along a metallic CNT. Temperatures at which each isotope label were incorporated are shown. **f** Reconstructed length evolution of the CNT, indicating the extracted activation energy to be ~2.3 eV for this particular case. **g** SEM and AFM images to trace the catalyst nanoparticle responsible for the growth of the m-CNT.

Comment. *5. « Er of 2.6 eV » (Otsuka et al., p. 15): To be more convincing, this activation energy value should be discussed in comparison with those previously reported in the literature for Ostwald ripening for similar systems.*

We thank the reviewer for the important comment. First, we are not able to find experimental activation energies for Ostwald ripening of the exact same system (iron nanoparticles on r-cut quartz) or similar. In our study, the *E*r of 2.6 eV was determined from the hysteretic behavior of CNT growth rates, and the same value is used for kinetic Monte Carlo simulations. The latter was practically achieved by reducing the adatom adsorption energy E_{ad} by 0.3 eV compared to the simulations by Prévot [*PRB* **84**, 045434 (2011)] (Co on graphite). A first-principle kinetic study shows that the total activation energies of ripening on TiO_2 are 2.34 eV for Ni. Although data for Fe is missing, we can reasonably expect a similar value for Fe. We have added a comparison with this theoretical study as follows:

On page 14, line 265

Notably, this ensemble model reproduces the average growth rate of s-CNTs (Fig. 2f) when E_r = 2.6 eV is used, which reasonably agrees with a first-principle kinetic study for Ni on TiO_2[48].

Comment. *6. « Distributions of apparent Ea, which are calculated from T ramp-up (red) and ramp down (blue) periods in KMC simulations. » (Otsuka et al., p. 16) In the calculations, the apparent Ea for T ramp-up is around 2 eV, while it is around 3-4 eV experimentally (fig. 3e). I understand that the authors later explain this discrepancy by the hypothesis that « Relatively large ones among simulated particles in panel (f) are assumed to grow CNTs. » (Otsuka et al., p. 16) and « we assume that CNT growth preferentially occurs on relatively larger catalyst particles, » (Otsuka et al., p. 17). The whole section is a bit unclear (is it just a qualitative comment? Or is it actually used in the latest version of the calculations?). The authors should precise which values or threshold size correspond to "larger particles" or "relatively large ones", and whether and how they integrated such a threshold size in their latest modelling version (eg for figure 4g)".*

We thank the reviewer for pointing out that this part was not sufficiently clear. We actually used a simple assumption that only the particles larger than a threshold are responsible for CNT growth in the latest version of the calculations. As already shown in Supplementary Fig. 9, CNT growth in the experiments is observed only from a subset of catalyst particles spanning a broad size distribution, and the active particles are biased toward relatively larger ones. To obtain a *qualitative* understanding of the experimental trend, we introduced a simple threshold in the CNT-growth simulation. Specifically, the KMC simulations were performed for 36 independent runs with 100 particles each (3600 particles in total). Among them, 388 particles survived until the final simulation time (900 s), and we assumed that the 185 larger ones contributed to CNT growth, whereas the remaining particles were excluded because they predominantly disappeared during the ripening process. For this selected subset, CNT growth was then sampled repeatedly by assigning random growth-onset times and growth lifetimes.

We stress that this thresholding was used only in the simulation for CNT length evolution and was not incorporated into the KMC calculation of nanoparticle ripening itself. We also note that the experimental distribution of apparent E_a is broader than that obtained in the KMC-based estimate. This is reasonable because, in the experiments, additional sources of variability are present, including uncertainty in estimating the growth rate, tube-to-tube variations in chirality and intrinsic activation barriers, and spatial inhomogeneity of the substrate surface. To keep the main text concise, we made only a minimal clarification there and added the detailed description to Supplementary Note 8.

On page 16, line 301

To gain further insight into stochastic CNT elongation, we simulated CNT growth from the KMC-derived nanoparticles. Since our measurements resolve only CNTs whose length and growth rate exceed detection thresholds, we selected a relatively large subset of catalysts to calculate an instantaneous growth rate γ described by Eq. (3) (Supplementary Note 8 for details).

Comment. *7. « Among 158 CNTs analyzed, 139 tubes (88%) maintained their chirality (n,m) over the entire measured length. The total inspected length was 9.39 mm, within which 24 (n,m) change events were observed, corresponding to a length-normalized frequency of 2.6 mm−1 (391 µm). » (Otsuka et al., p. 19): The authors appear to have neglected the most probable scenario: given that there is no a priori reason for the new chirality to maintain the same resonance energy, a chirality change likely results in a complete loss of resonance at the excitation energy used (532 nm). In the Raman map at 532 nm, this would manifest as a short segment—either (i) distinct from the catalyst line (easily identifiable) or (ii) connected to it (more difficult to distinguish from normal tubes). While the few provided Raman maps seem to show few such instances, their potential presence warrants discussion and consideration in the analysis.*

We agree that the present analysis may overlook shifts in resonance energy upon chirality change. Combining other excitation wavelengths for Raman analysis would be the best practice, but it is not feasible considering the limited efficiency of a Raman spectrometer for the current CNT sample (long and low density) compared to our custom-built Raman system equipped with a green laser. Instead, we used SEM to compare Raman mapping images and corresponding SEM images as shown in newly added Supplementary Fig. 12.

Although Raman and SEM images are consistent with each other for most cases, we rarely found the CNTs that appear longer in SEM than Raman mapping, suggesting changes in resonance window, as the reviewer concerned. Roughly speaking, this probability is not high compared to the chirality change events judged from Raman spectra alone, which can be qualitatively rationalized by the study by Chai et al. [*JACS* **147**, 19498 (2025)]. They observed a number of chirality change events and found that (*n*,*m*)->(*n*+*i*,*m*-*i*) changes were majority while preserving semiconducting features. Since (2*n*+*m*) families before and after such chirality changes are different, we suspect that resonance energy changes were non-negligible but moderate enough so that most CNTs remain somewhat resonant to the same excitation laser before and after chirality changes, rather than fully disappear in Raman mapping measurements. Nevertheless, our initial assessment is very likely to overestimate chirality preservation, and we have added a note on this point in the main text and Supplementary Note 10, while retaining the same estimated values in Fig. 5 for comparison with previous studies.

On page 18, line 340

We note that changes in Raman resonant conditions can be overlooked and may lead to overestimation of these values(Supplementary Note 10); however, the overall level of chirality preservation is comparable—although classification may be modestly influenced by CNT array density—indicating that dynamically evolving environments do not measurably increase the incidence of chirality transitions.

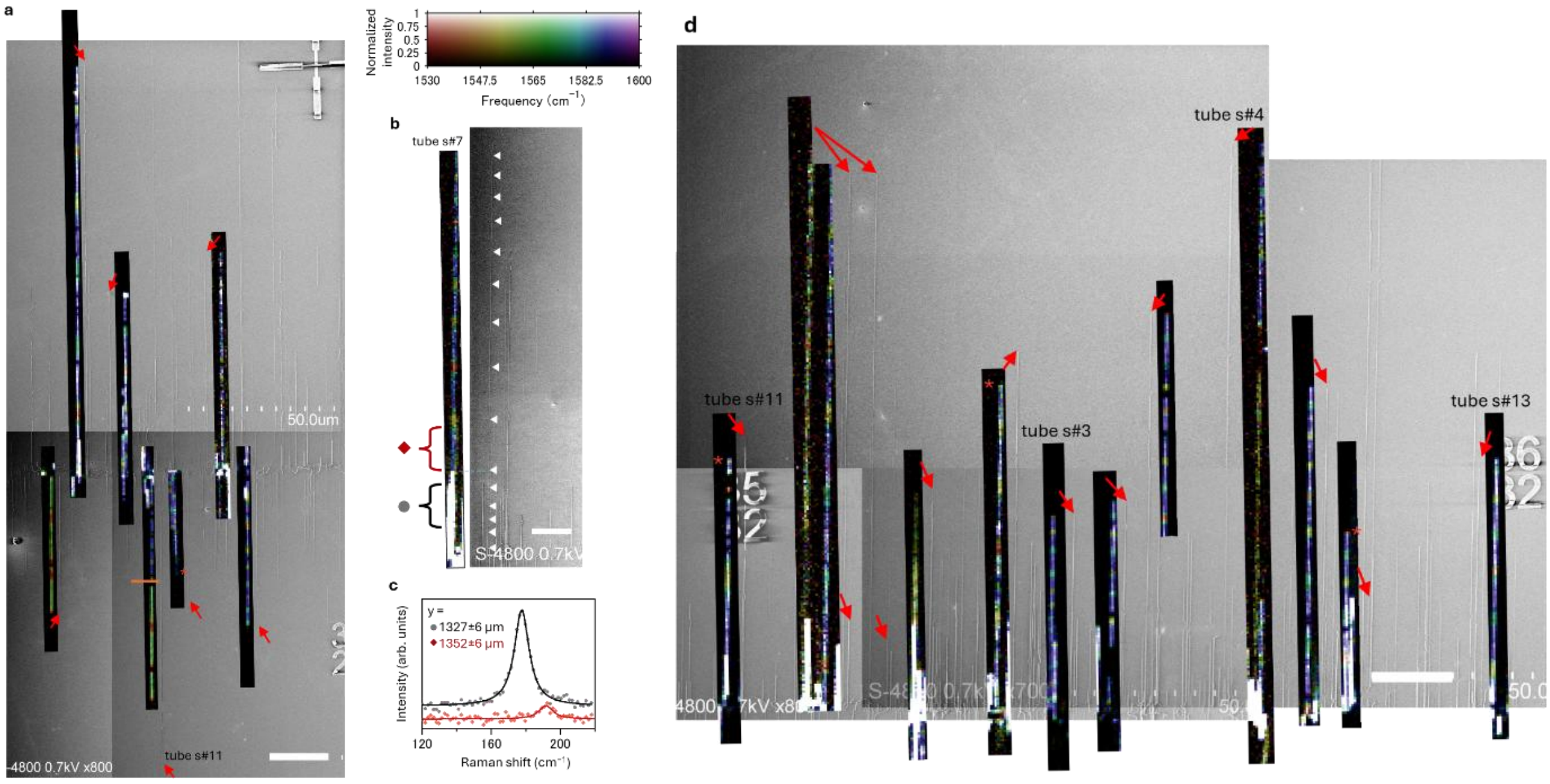

Supplementary Fig. 12 | Comparison of Raman mapping and SEM images. a SEM images of CNT transferred onto a Si/SiO_2 substrate for Raman measurements. After roughly conducting Raman mapping measurements over a very large area, CNTs indicated by red arrows (resonant to a 532 nm laser) were characterized by Raman spectroscopy, and the corresponding Raman mapping images are shown adjacent to the SEM image. Horizontal orange lines indicate the chirality change positions. Stars (*) indicate the inconsistency between SEM and Raman images, which suggests some changes in Resonance condition. **b** SEM and Raman mapping images of tube #7 in Fig. 5a (tube #s7 in Supplementary Fig. 10). White triangles indicate the isotope labels, and a horizontal dashed line in light blue indicate the junction where the Raman features abruptly change. **c** RBM spectra of the CNT before (y = 1352±6 μm in Supplementary Fig. 10) and after (y = 1327±6 μm) the chirality change event. **d** SEM and Raman mapping images of CNTs in other regions. Scale bars are 20, 10, and 20 μm in panels (a), (b), and (d), respectively.

** Larger figures are available in Supplementary Information

Comment. *8. « As shown in Fig. 5c, Raman mapping measurements further verify the RBM continuity along nanotubes (Supplementary Fig. 2), demonstrating chirality preservation in the FCCVD system. » (Otsuka et al., p. 20): Again, the most plausible explanation is that the chirality change leads to a total loss of resonance at the excitation energy used (532 nm), rendering the tube with the new chirality undetectable, rather than merely causing a discontinuity in the RBM features. To confirm the absence of a chirality change, additional SEM images or Raman maps at other wavelengths of the same region may be provided. This would also require the tubes to be sufficiently dispersed and unbundled for individual resolution, a condition that may be challenging to meet with these samples.*

We agree with the reviewer's comment. We have already included Supplementary Fig. 3, which presents SEM and Raman mapping of as-grown FCCVD-derived CNTs directly deposited on a substrate. Assuming that CNTs are approximately visualized in SEM, the correspondence between the SEM morphology and the Raman mapping confirms consistency between the two measurements for two CNTs long enough for RBM-based evaluation. On the other hand, as panel d shows, continuity analysis is substantially more difficult in FCCVD-derived samples than in quartz-grown CNTs, because short CNTs can be deposited close to longer CNTs and may also form bundles. Therefore, while the present data demonstrate that chirality can be preserved over ~10 µm length scales even in the FCCVD system, they do not establish the average frequency of chirality-change events in FCCVD growth. We regard that question as beyond the scope of the present study and an important subject for future investigation.

Comment. *9. The authors should propose an explanation for the discrepancy between their results and those of Yao et al. (Temperature-mediated growth of single-walled carbon-nanotube intramolecular junctions. Nat. Mater. 6, 293–296 (2007)): is it more likely a difference in experimental conditions (if so, which key difference?), or a potential flaw in the methodology used by Yao et al?*

Response. We thank the reviewer for raising an important question on the contradiction with previous studies. One of the possible explanations would be difference in growth rates, as the growth rate reported by Yao et al is an order of magnitude larger than that in our experiments. Although we could not judge from the provided information whether their methodology has a potential flaw, our study did try to avoid the ambiguity in assigning nanotube structures (i.e., chirality) by selecting appropriate substrates and environments (CNTs were transferred onto Si/SiO_2), as well as the protocol for reconstructing time evolution of CNT growth (isotope labeling). For example, differences of thermal expansion coefficients between as-grown CNTs and substrates, or interactions with substrates induce strain and doping to CNTs, which can significantly change the lineshape, intensity, and frequency of Raman features [*ACS Nano* **20**, 7794 (2026)], leading to the misassignment of structural changes in CNTs. If tensile strain persists, both G-mode and RBM exhibit a downshift [*Chem. Commun.* 2009, 6902], possibly explain the phenomena observed in the literature, where six intramolecular junctions were reported under a temperature oscillating condition, while retaining its metallic electronic structure (Figure 2 and Figure S6, Yao:2007).

To minimize such ambiguity, we transferred CNTs using PMMA thin films to release strain and doping, and then acquired (typically) more than 100 spectra from single CNTs. We have added a figure to show the spatial uniformity of the measured samples in Supplementary Fig. 2 and related discussion on this point.

On page 8, line 10, in Supplementary information

Supplementary Fig. 2e shows the G^+-mode peak frequencies extracted from more than 100 points along the same CNT (tube #1 in Fig. 2c). Except for obvious isotope-induced downshifts, each tube segment shows highly uniform peak frequencies and linewidths owing to the transfer

process and a minimum doping effect from Si/SiO_2 substrates. These features enable more reliable and spatially resolved identification of chirality preservation or change. In contrast, conventional measurements on as-grown substrates are often affected by residual thermal stress and limited spatial sampling, making it difficult to distinguish extrinsic spectral variations from intrinsic structural changes.

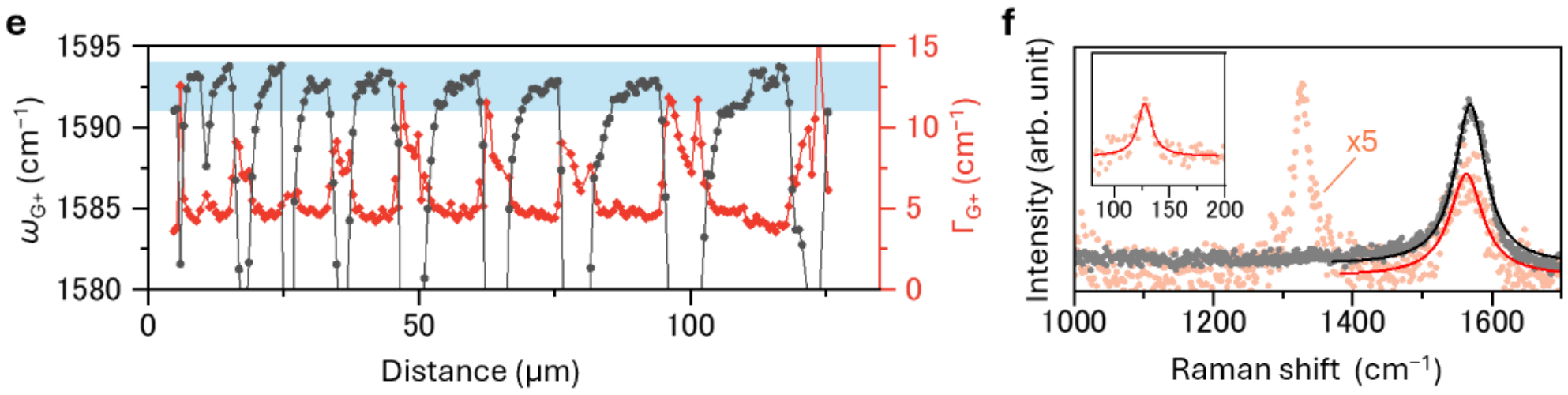

Supplementary Fig. 2 | Aligned, isolated CNTs for isotope labeling analysis. e G^+-mode peak frequencies ω_{G+} and linewidths Γ_{G+} acquired along tube #1 in the main text (Fig. 2). Despite the mixed supply of ^{13}C-nriched ethanol, most of measured spectra peak within the blue shared region, which indicates a 3 cm^{-1} width. **f** Raman spectra obtained from a metallic CNT (light red, tube #s11 in Supplementary Fig. S10), which shows a RBM peak at 127 cm^{-1} (diameter of ~2.03 nm), from a metallic-like CNT (gray, tube #s4 in Supplementary Fig. S10) judged from G-mode shape. These spectra are averaged over 4.2 (7 points) and 9 μm (15 points) along their axis, respectively.

Other points:

Comment. *« Viewed from the standpoint of the growing material, these processes could be regarded as Eulerian and Lagrangian descriptions, respectively. » (Otsuka et al., p. 3) « From a Lagrangian perspective » (Otsuka et al., p. 4): A bit jargon laden. I understand what is meant but it could be stated more simply.*

Response. We thank the reviewer for the comment for improving the readability. We have removed the corresponding description and simplified the introduction.

Comment. *« whereas floating catalysts enlarge mainly through particle coalescence at relatively low temperatures 11 » (Otsuka et al., p. 3): Beside theoretical work (ref. 11), citations of experimental observations would also be welcome to support this assertion.*

We thank the reviewer for the important aspect to be added to the introduction. Although direct measurements of catalyst size evolution are limited and absent for the exact temperature range of the present study (<1000 K), we believe that the experimental work by Hoecker et al. [*Carbon* **96,** 116 (2016)] adequately supports the statement, where catalyst nanoparticle dynamics along the reactor were investigated at temperatures of 1150–1300°C. They observed disappearance of

particles due to evaporation in the hottest zone, but importantly, clear coarsening of catalyst particles at the entrance (~700°C) until they reached the hottest zone, and CNT growth was observed at such a low-temperature region. We have added experimental support for the assertion as follows:

> On page 3, line 39
> In contrast, in floating-catalyst reactors, catalysts usually nucleate from volatile metal precursors and enlarge predominantly through particle coalescence at relatively low temperatures, while evaporation-driven redistribution also plays a crucial role at higher temperatures[11,12.]

Comment. *« have revealed » (Otsuka et al., p. 4): Should this instead be interpreted as "have not revealed"?*

Response. We appreciate the careful reading of the manuscript. The comment is correct, and we have revised this point.

Comment. *« supported by recent MD simulations using machine-learning force fields28,29. » (Otsuka et al., p. 4): It should be more specifically explained in which way these recent MD simulations support "the preservation of chirality over hundreds of micrometers".*

Response. We thank the reviewer for carefully reviewing the statement. We fully agree that it requires more specific explanations on how MD simulations support such a long-scale preservation of nanotube chirality. Hedman et al. modeled defect healing kinetics based on the observation of MD simulations of time scale up to 1 μs and draw a relationship between temperature, growth rate, and expected lengths without lattice defects. We have revised the corresponding sentence to clarify this point.

> One page 4, line 64
> Even when non-equilibrium kinetic processes are considered, chirality preservation over hundreds of micrometers is also well reported for high-quality CNTs grown under steady conditions[24–27] and is further supported by MD simulations through the kinetic modeling of defect-healing processes [28,29].

Comment. *« Supplementary Fig. 1 » (Otsuka et al., p. 7): the Raman excitation wavelength should be indicated.*

Response. We thank the reviewer for pointing out the important information missing. We have added the excitation wavelengths for all the figure panels.

***Comment.** « by extrapolating the growth rate defined between the last two isotope labels down to the catalyst stripe » (Otsuka et al., p. 9): Understood but it could be formulated more clearly.*

We thank the reviewer for pointing out the unclear formulation of how the growth termination timing is estimated. <u>We have rephrased the sentence so the assumption of linearly growth rate after the last isotope labels is clearly delivered</u>.

> <u>On page 9, line 156</u>
> To quantify the temperature dependence of growth termination, we approximated the termination time for each CNT by linearly extrapolating the growth rate defined between the last two isotope labels to the catalyst positions (Fig. 3a).

***Comment.** « the semiconducting-to-metallic tube ratio remains essentially unchanged over the temperature range examined (Supplementary Fig. 3) » (Otsuka et al., p. 10) Figure S3 may more clearly describe the average SC/M ratio (e.g. pondered over the 3 wavelengths) in each of the three cases (800, 875 °C, 800-873-800)*

Response. We agree that Supplementary Fig. 3 (Supp. Fig. 4 in the revised version) needs more clear description of average ratio of semiconducting and metallic CNTs. <u>We have added description in Supplementary Note 4.</u> Unfortunately, although we performed additional measurements using other excitation wavelengths, because efficiency and machine time of the available Raman spectrometer (Renishaw inVia) are quite limited to obtain sufficient statistics for sparse samples (see Supplementary Fig. 12), we are not able to show a meaningful SC/M ratio for the 800-873-800 case.

***Comment.** « c) Survival probability p over a 1 s interval for a growing CNT plotted against temperature. Arrows show the direction of temperature modulation. Error bars represent the standard errors. » (Otsuka et al., p. 12): Figure 3c: a table detailing the data used to calculate the probabilities and the error bars should be given in SI. I would suggest plotting the confidence interval (more statistically meaningful), rather than the standard deviation, for the uncertainty bars.*

Response. We thank the reviewer for this suggestion. We have added Supplementary Table 2, which lists the data used for Fig. 3c (*T*, *D*, and *N* for each time bin). Regarding Fig. 3c and Supplementary Fig. 6e, we have also replaced the standard-error bars with 95% confidence intervals assuming Poisson statistics, and revised the figure caption accordingly.

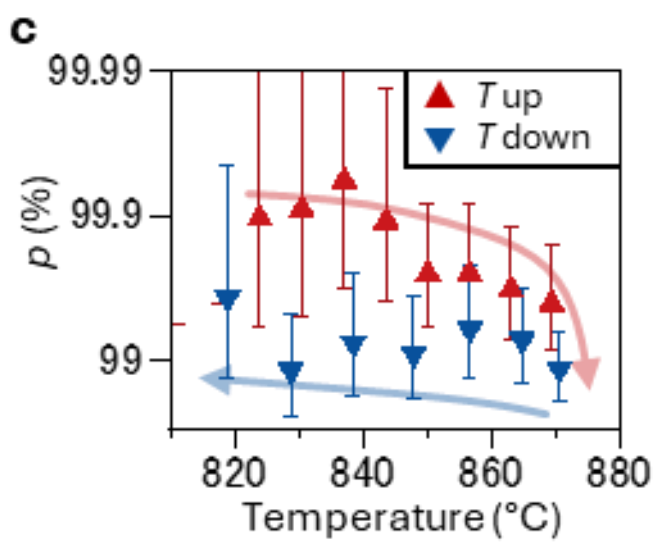

Fig. 3 | Growth termination and kinetic hysteresis under thermal modulation. c Survival probability (*p*) over a 1-s interval for growing CNTs plotted as a function of temperature. Arrows indicate the direction of temperature modulation. Error bars represent 95% confidence intervals assuming Poisson statistics.

Comment. *« e) Distributions of apparent Ea obtained by linear fitting of the Arrhenius plots, separately for temperature ramp-up (T up) and ramp-down (T down) periods. » (Otsuka et al., p. 12): Fig. 3e: to evaluate the data quality, all the Arrhenius plots used to build fig 3e should be (more) clearly shown in SI, not only those in Fig. 3d."*

Response. We sincerely thank the reviewer for careful evaluation including Supplementary Figures. We have separated the Arrhenius plots for s-CNTs and m-CNTS, as well as those of temperature ramp-up and ramp down periods for clarity.

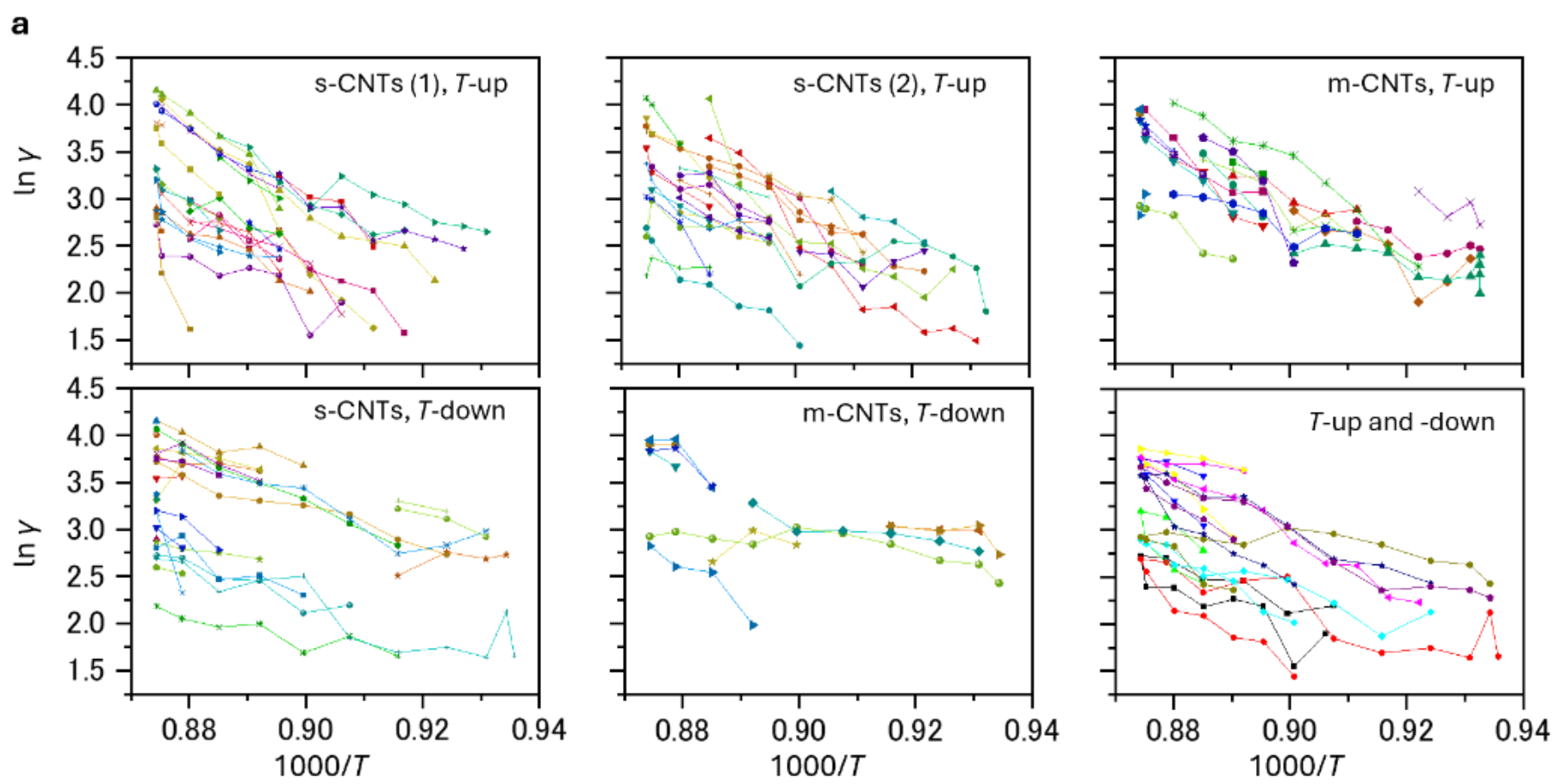

Supplementary Fig. 6 | Arrhenius and survival analyses supporting kinetic interpretations. a Arrhenius plots of the growth rate for a large number of individual CNTs. For clarity, panels are separated for s-CNTs and m-CNTs, as well as for *T* ramp-up and *T* ramp-

down. All CNTs whose apparent E_a for both T ramp-up and ramp-down are evaluated are plotted in the bottom right panel.

Comment. *« Note that transition from tangential to perpendicular modes alone cannot explain this behavior because such a transition should involve the catalyst size change as long as the tube chirality remains unchanged. » (Otsuka et al., p. 13) A bit unclear: please reformulate more clearly.*

Response. We appreciate the comments on this insufficient description. We have revised the corresponding sentence as follows.

On page 13, line 232
We note that a transition from tangential to perpendicular growth modes[17] alone cannot account for the growth rate change, because such a transition presupposes a corresponding change in catalyst size as long as tube chirality remains unchanged.

Comment. *« empirically scales as R^4» (Otsuka et al., p. 14) The authors should provide an additional citation to justify their use of a power-law exponent of 4 in this context, as opposed to the conventional Ostwald ripening law with a power of 3.*

Response. As the reviewer pointed out, for the diffusion-limited coarsening, the rate exponent is equal to 1/3 in three-dimensional clusters in bulk (3D/3D). When the model extended to ripening of islands on surfaces (3D/2D), analytical solutions were given by Chakraverty [*J. Phys. Chem. Solids* **28**, 2401 (1967)] with a growth exponent of 1/4 for diffusion-limited coarsening, while a rate exponent of 1/3 for attachment-limited coarsening. Although this 1/4 exponent is reasonable approximation for sufficiently sparse particles and used in the analytical model in the present study, the actual power-law exponent may be even smaller as the KMC simulation results suggested. To clarify the 3D/2D modeling, we have modified the introduction of LSW-type description as follows:

On page 13, line 244
To estimate the impact of catalyst coarsening on CNT growth rates, we adopted a Lifshitz–Slyozov–Wagner (LSW)-type description of Ostwald ripening, extended to sparsely distributed, surface-supported nanoparticles. In this framework, the average particle radius $\bar{R}(t)$ for diffusion-limited coarsening empirically follows[45,46]

Comment. *« this ensemble model agrees well with the average growth rate for s-CNTs (Fig. 2f) » (Otsuka et al., p. 15): Why mentioning s-SWCNTs only? The authors previously wrote that m-CNTs and s-CNTs have close kinetics.*

Response. We thank the reviewer for pointing out the inconsistency in our manuscript. Although the temperature dependence of growth rates between s-CNTs and m-CNTs is quite similar, there is a slight but systematic difference: growth rates of m-CNTs are faster by ~20%, which is consistent with our previous study [*ACS Nano* **16**, 5627 (2022)]. Since the number of growing CNTs is small at the initial and final stages of growth, the ensemble growth rate is highly influenced by the number of m- and s-CNTs, leading to a large error. To avoid this issue, we only took s-CNTs for discussing ensemble trends.

Comment. *« Consistently, a comparable hysteretic behavior found in the growth lifetime (Fig. 3c) provides independent evidence for irreversible catalyst coarsening, » (Otsuka et al., p. 18) Does it mean that the authors conclude that Ostwald ripening is the main cause of catalyst deactivation in these conditions? If so, how their model could account (at least qualitatively) for the growth lifetime hysteresis?*

Response. As the reviewer stated, we attribute Ostwald ripening to the observed hysteretic behavior of growth lifetime. This is based on the model described in Supplementary Note 6 and Fig. 7. Briefly, carbon chemical potential becomes higher for the larger catalysts according to the model we proposed [*ACS Nano* **16**, 5627 (2022)], resulting in higher growth rate at the same time. This premise plays a key role in the present study. On the other hand, it is widely recognized that one of the major mechanisms for growth termination is encapsulation of catalysts by carbon, which requires nucleation of carbon layers on catalysts. As such nucleation processes can be approximated by graphene nucleation on metal surface, we derive a relationship between the chemical potential and the nucleation frequency (inverse of lifetime) as in Supplementary Eq. 5. We should note that catalyst deactivation is mostly a stochastic event and can occur without Ostwald ripening, but rather accelerated by catalyst coarsening. The following sentence has been modified to clarify the role of chemical potential and to adjust the strength of the claim.

> On page 17, line 313
>
> The comparable hysteresis in growth lifetime (Fig. 3c) may reflect the increase in chemical potential, providing independent support for irreversible catalyst coarsening, rather than transient environmental fluctuations (see Supplementary Note 7 for modeling).

The correlation between growth rate and lifetime (likely through chemical potential) can actually be observed in a constant temperature condition in Supplementary Fig. 6e,f. Furthermore, this is qualitatively consistent with the findings by Navas et al., stating "At short times, most catalyst particles are uncovered, whereas at intermediate and long times, many particles, **mostly large ones, are encapsulated by carbon layers or decorated by carbon structures** (Figure 5c). This suggests that carbon poisoning is a source of growth termination, especially at high precursor pressures and low synthesis temperatures." This consistency with Navas's study is now mentioned in SI.

***Comment.** « Figure 5a shows Raman profiles acquired along three representative CNTs. » (Otsuka et al., p. 18) The temperature profile experienced by each tube and the instantaneous growth rate (extracted from the digital isotope encoding) should be shown in fig. 5a and S7, to more clearly support the conclusion that the change in temperature does not systematically lead to a change in chirality at the individual CNT level.*

Response. We thank the reviewer for the comment regarding the correlation between temperature and chirality change. We believe that Supplementary Fig. 11c (previously Fig. 8c) answers half of the reviewer's question, which shows the individual timing of chirality change events and apparent E_a before these events (if definable). Although the chirality change was provoked near the highest temperature (instantaneous temperature change is minimum though) for tube #7 in Fig. 5, there is no clear trend observed for the chirality change timing. Note that analysis in Fig. 5 was performed more or less independently of that shown in Figs. 2-4 (different regions of the same substrate) to avoid bias arising from the capability of digital isotope encoding, where growth rates higher than a certain threshold are required. Therefore, all the cases that experienced chirality changes with definable growth profiles are displayed in Supplementary Fig. 11c, among 19 cases presented in Fig. 5b. We have added instantaneous growth rates to the same figure panel to support the conclusion as suggested by the reviewer.

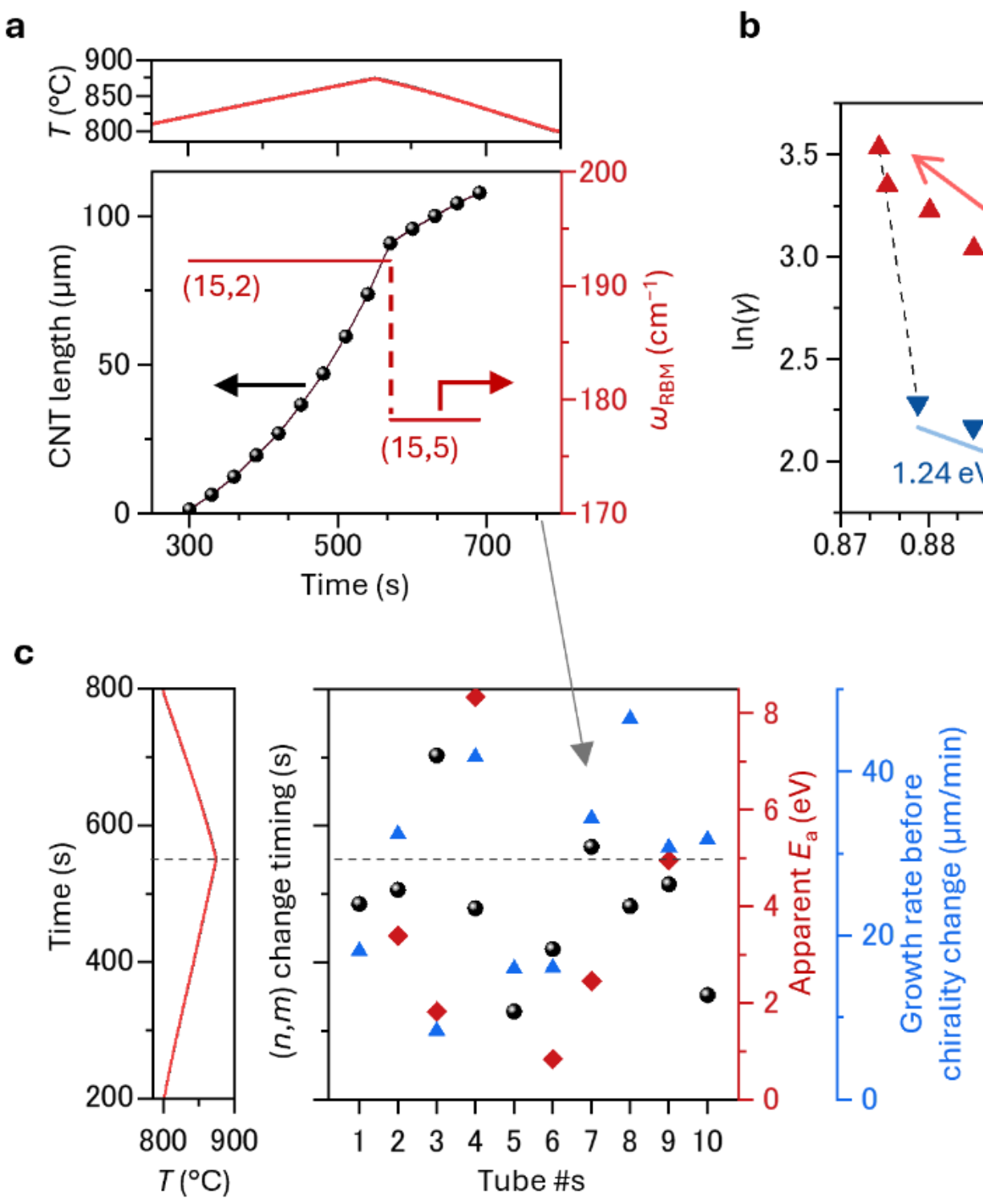

Supplementary Fig. 11 | Timing and kinetics of rare chirality-change events. c Chirality-change timing (black), apparent activation energy (red), and instantaneous growth rate (blue) before the transition for ten more CNTs.

Comment. *« in contrast, tube #7 is a rare example in which an abrupt spectral change is observed for both G-mode and RBM. » (Otsuka et al., p. 18). In the type of case displayed by tube #7, the authors should explain how they distinguish a bundle of two tubes (both emanating from the catalyst line) from a change in chirality.*

Response. We appreciate the important comment on the possibility of bundling multiple CNTs. First, we designed the experiment to avoid this issue by reducing the catalyst amont and CNT density as shown in Supplementary Fig. 2 the probability of detecting multiple CNTs in a laser spot. We have obtained additional SEM images for a part of CNTs analyzed by Raman mapping (Supplementary Fig. 12) as displayed earlier in the response document. However, in the case of tube #7, as we found a few more CNTs lying closely, we also carefully analyzed the average RBM spectra before and after the tentative chirality changing point (Supplementary Fig 12c). RBM component peaked at 192 cm^{-1} (observed at the upper region) is not detected from the lower segment (gray plots), excluding the possibility of bundling for this particular case. Similar processes were performed for all CNTs in the form of Supplementary Fig. 10 and isolated spectra derived from them.

Comment. *« see Supplementary Fig. 10 for detailed D-mode analysis » (Otsuka et al., p. 19) What is the explanation of the authors for the disappearance of the D band at 400°C under vacuum? An extrinsic factor?*

Response. As the reviewer suspect, we also attribute the disappearance of the D band at 400°C to extrinsic factors. More specifically, the reduction of the D band upon heating to 200–400°C under vacuum is attributed mainly to the removal of surface functional groups [*J. Phys. Chem. B* **108**, 19949 (2004)] introduced during/after growth as discussed in Supplementary Note 12 and Fig. 14. The small residual D band after annealing is tentatively ascribed to substrate-induced symmetry breaking and/or dielectric inhomogeneity, rather than irreversible lattice defects. We have added the above reference to Supplementary Note 12.

Sup Info:

Comment. *- « The fraction of semiconducting CNTs is shown for each excitation wavelength. » (Otsuka et al., p. 10): Please add the excitation wavelength on each graph. Please precise what is the value expressed in "µm-1" (linear nanotube density?).*

Response. We thank the reviewer for pointing out this important information. We have described the excitation wavelengths for all figures.

Comment. *- « Diameter distribution of CNTs grown under for three different temperature profiles. Excitation wavelength of 532 nm is used. Average diameter is indicated by the vertical dashed lines. » (Otsuka et al., p. 10). Why using only the 532-nm data? (the authors have RBM data at other wavelengths for reference growths at constant T).*

Response. We agree that diameter evaluation with multiple excitation wavelengths is also required for the samples with temperature-modulation. As discussed earlier, we intentionally reduced the CNT density for this particular experiment. Custom-made Raman system equipped with a green laser (532 nm) can automatically acquire hundreds of Raman mapping images (such in Supplementary Fig. 12) based on rough positioning of CNTs, and was predominantly used to obtain the data provided in the manuscript. On the other hand, although we have tried to obtain diameter distribution using other excitation wavelengths (633 and 785 nm), due to limited efficiency and machine time of commercial Raman spectrometer (shared facility), we found the same analysis with sufficient statistics was not feasible. <u>We have added the specific reasons for the choice of excitation wavelengths in Supplementary Note 1.</u>

Comment. *- « Arrhenius plots of the growth rate for a large number of individual CNTs. » (Otsuka et al., p. 12): the ramp-up and ramp-down data should be shown separately for clarity.*

Response. We thank the reviewer for the important suggestion. <u>We have added Arrhenius plots separately for ramp-up and ramp-down periods in Supplementary Fig. 6a, as displayed earlier in this response document.</u>

Comment. *- « Apparent activation energy Ea plotted against the average temperature of each growth segment during the temperature ascent » (Otsuka et al., p. 12) If available, the same data should also be shown for ramp-down.*

Response. We appreciate the insightful suggestion. <u>We have added the apparent Ea plots in Supplementary Fig. 6b.</u>

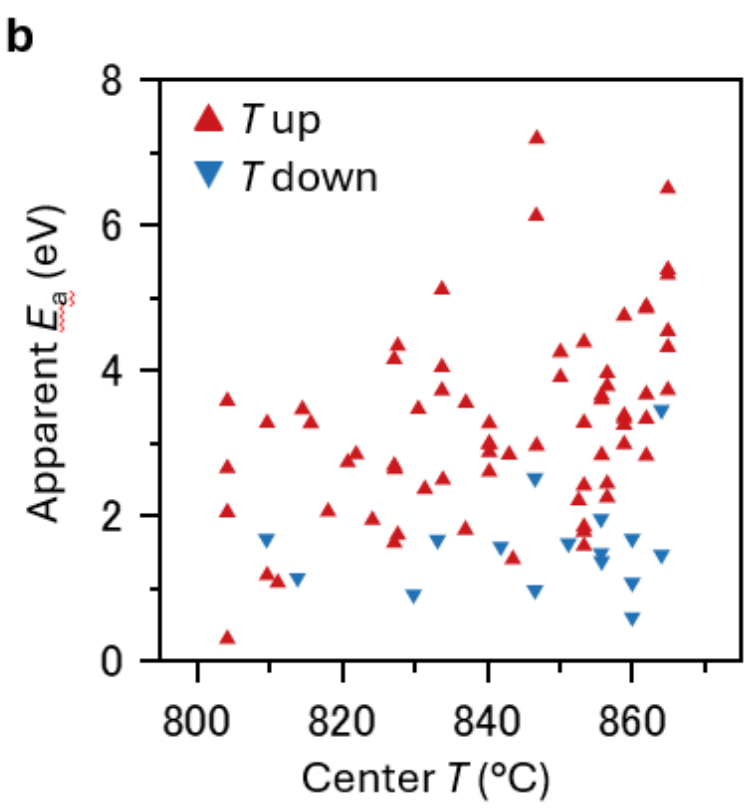

Supplementary Fig. 6 | b Apparent activation energy E_a plotted against the average temperature of each growth segment during *T* ramp-up (red) and ramp-down (blue) periods.

Comment. *- « Comparison of apparent activation energies Ea extracted from the T ramp-up and ramp-down periods for CNTs that grew across both stages. » (Otsuka et al., p. 12) The Arrhenius plots should also be shown separately to more clearly assess the data quality.*

We thank the reviewer for suggestion. We have added Arrhenius plots that show both temperature ramp-up and ramp-down periods in Supplementary Fig. 6a, as shown earlier in this document (page 27).

Response to Reviewer #4:

Comment. *The catalytic growth mechanism of SWCNTs, particularly the kinetic aspects and role of catalysts, has long been a focus of interest in the field of nanotubes. Previous work has primarily remained theoretical, with very limited experimental progress. Now, this work ingeniously employs isotopic labeling combined with growth condition fluctuations to reveal the kinetic growth mechanism of SWCNTs. Authors reported that chirality control can be established during the nucleation stage, while subsequent elongation can be optimized independently, this finding is quite important for the structure-controlled synthesis of SWCNTs. The experimental design is smart, and the characterization is comprehensive, providing a clear experimental picture of nanotube growth. This represents a significant breakthrough in the field and holds profound implications for the CNT-based electronic devices. This work will be of interest to researchers in the fields of nanotubes and other related material growth, and is therefore worthy of publication in Nature Communications after suitable minor revision.*

Response. We are delighted to hear that the reviewer stated that our work will be of interest to researchers in the field of nanotubes and other related materials. We believe that this study would be a solid basis for future progress in the research field by providing guidelines to achieving structure control and large-scale production. We found all the comments from the reviewer insightful and used them to improve the manuscript.

Comment. *1. Considering the strong van der Waals forces and epitaxial alignment between the quartz lattice and the SWCNTs, could this external constraint have enhanced the structural stability of the nanotubes?*

Response. We thank the reviewer for this important suggestion. We believe it is unlikely that the quartz substrate substantially enhanced chirality preservation through epitaxial stabilization of the nanotube lattice. First, this situation is fundamentally different from other epitaxial systems, such as bulk semiconductor growth and CNT alignment on hexagonal substrates such as hBN or graphite. In the latter case, crystallographic matching between the hexagonal lattice of the CNT and that of the substrate can lead to chirality-angle-dependent alignment [*Carbon* **246**, 120910 (2026)]. In contrast, alignment on quartz has been successfully reproduced by analytical models in which the CNT is treated simply as a continuous cylinder with different diameters [*Nano Lett.* **9**, 4311 (2009)]. These studies indicate that the alignment on quartz is governed primarily by anisotropic van der Waals interactions rather than by hexagon-by-hexagon epitaxial matching.

Second, robust chirality preservation is also observed in our kite-growth experiments under steady conditions, where the argument based on substrate-induced directional alignment does not provide a specific explanation. This further suggests that structural robustness is intrinsic to the nanotube growth process itself, rather than being primarily imposed by the quartz substrate.

Finally, our supplementary modeling and simulations suggest that substrate interactions may even have the opposite tendency in some cases: by inducing local deformation and curvature concentration, they can slightly promote defect formation and hence facilitate chirality change,

rather than suppress it. We added this point and the corresponding discussion in Supplementary Fig. 16 and 17.

On page 19, line 356

It is worth noting that, although we observed robust structural memory in the substrate-supported system, tube-substrate interactions can enhance local curvature[39] that lowers the kinetic barrier for defect formation[24] (see Supplementary Figs. 15 and 16). Chirality preservation in floating-catalyst systems is therefore expected to be at least comparably robust.

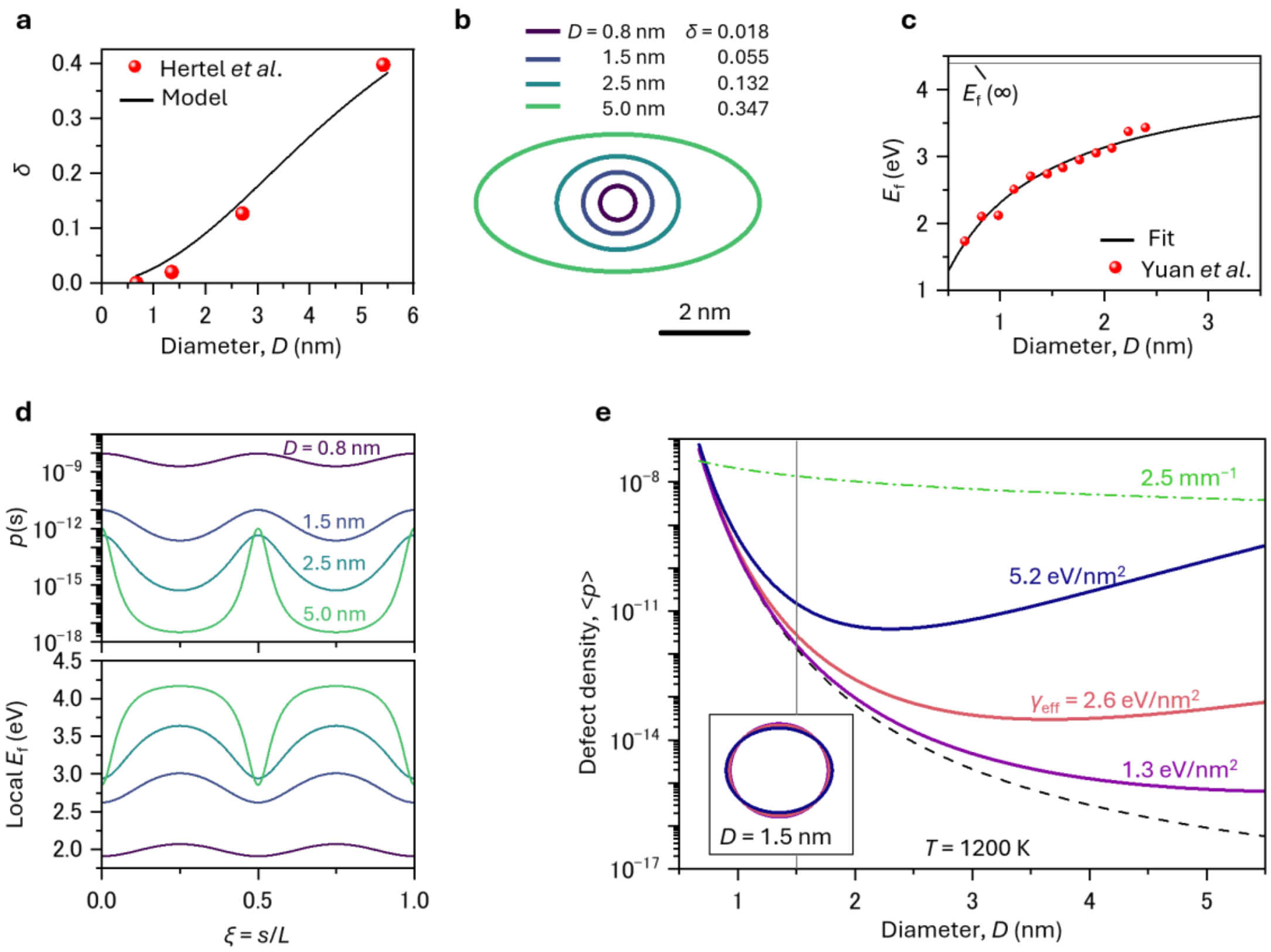

Supplementary Fig. 15 | Substrate-induced deformation and its impact on defect formation under thermal equilibrium. a Diameter dependence of the ellipticity δ obtained from the continuum model, with molecular mechanics results[36] shown for reference. **b** Cross-sectional shapes of nanotubes with different diameters. **c** Curvature-dependent defect formation energy based on the calculated data of Yuan *et al.*[16] and the fitted curve used in this work. **d** Spatial variation of the local defect formation energy and the corresponding Boltzmann factor along the tube circumference for selected diameters. **e** Circumferentially averaged defect density as a function of nanotube diameter at T = 1200 K for different substrate interaction strengths. The black dashed line indicates the undeformed case, the vertical line marks a representative diameter (D = 1.5 nm). The inset compares the cross-sectional chapes of a nanotube with different interaction strengths.

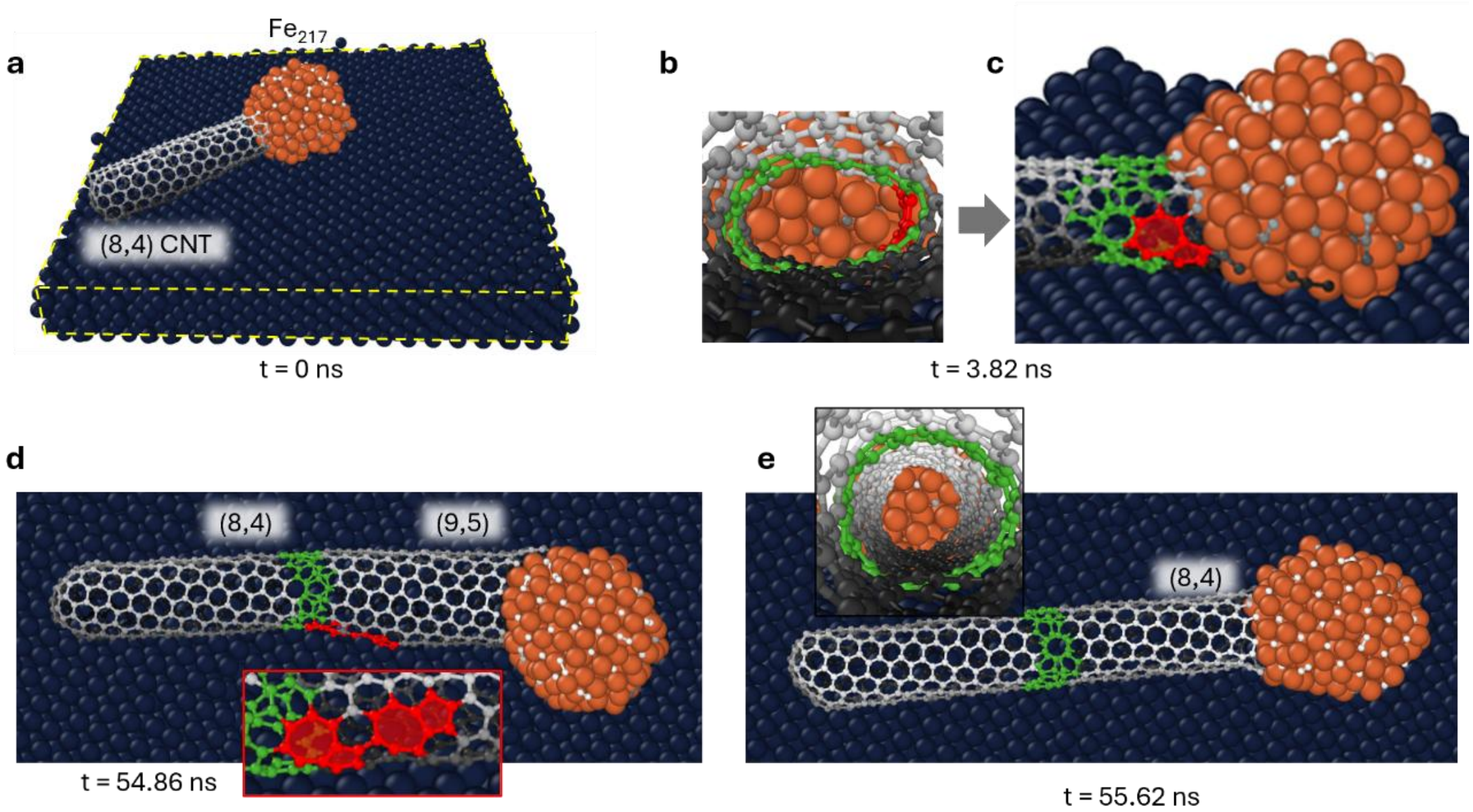

Supplementary Fig. 16 | Molecular dynamics simulations of substrate-induced deformation and defect formation. a Initial configuration at t = 0 ns after reaching quai-equilibrium for a (8,4) CNT segment connected to an Fe catalyst particle on a substrate. **b** Elliptical deformation of the CNT under strong van der Waals interaction with the substrate; green atoms indicate the tube–catalyst interface. **c** Formation of a pentagon–heptagon defect pair (red) at regions of enhanced curvature, accompanied by a local change in chirality. **d** Subsequent elongation of the transformed (9,5) CNT segment at t = 54.86 ns. **e** CNT grown under weaker substrate interaction, maintaining a circular geometry and preserving its chirality.

Comment. *2. On line 395, the authors draw an analogy between the chirality-preserving phenomena in SWCNT growth kinetics and DNA replication, which is physically inappropriate. Can authors revise or change this description?*

Response. We thank the reviewer for pointing out the inappropriate analogy to DNA replication. We agree that the term "proofreading" implies a specific energy-dissipation mechanism that is not applicable to the present system. We have therefore removed the original statement suggesting that catalytic CNT growth intrinsically involves a proofreading-like mechanism analogous to DNA replication. In the revised manuscript, we retained only two limited points of comparison. First, both processes involve rapid one-dimensional elongation localized at a reaction front or interface. Second, in both cases structural fidelity is maintained locally before irreversible extension proceeds. We have revised the paragraph accordingly to avoid implying a direct mechanistic equivalence between the two systems.

On page 21, line 413:
In this sense, CNT growth may share a few features with biochemical processes. For example, during DNA replication, rapid one-dimensional elongation proceeds strictly at the enzyme-substrate interface, where structural fidelity is maintained because incorrect intermediates are

removed before irreversible extension occurs[53,54]. In catalytic CNT growth, elongation occurs at a dynamically reconfigurable CNT–catalyst interface, where transient non-hexagonal defects increase the barrier of subsequent reactions and can be eliminated before being permanently incorporated into the tube wall[24,28,55] (see Supplementary Note 17 for MD simulations). Such dynamic behavior may account for efficient defect healing during one-dimensional crystal growth of CNTs.

In addition, through the consideration of the above issue, we performed MD simulations. Although these simulations did not justify retaining the original proofreading analogy, they yielded a useful mechanistic observation that we chose to keep as supplementary information. The simulations show a dynamic defect-removal behavior at the CNT–catalyst interface that is broadly consistent with previous studies[24,28], while suggesting an additional kinetic aspect whereby transient defect formation can temporarily hinder forward elongation and favor local reconstruction before permanent incorporation into the tube wall. We therefore added Supplementary Fig. 17 as supporting context for the revised discussion.

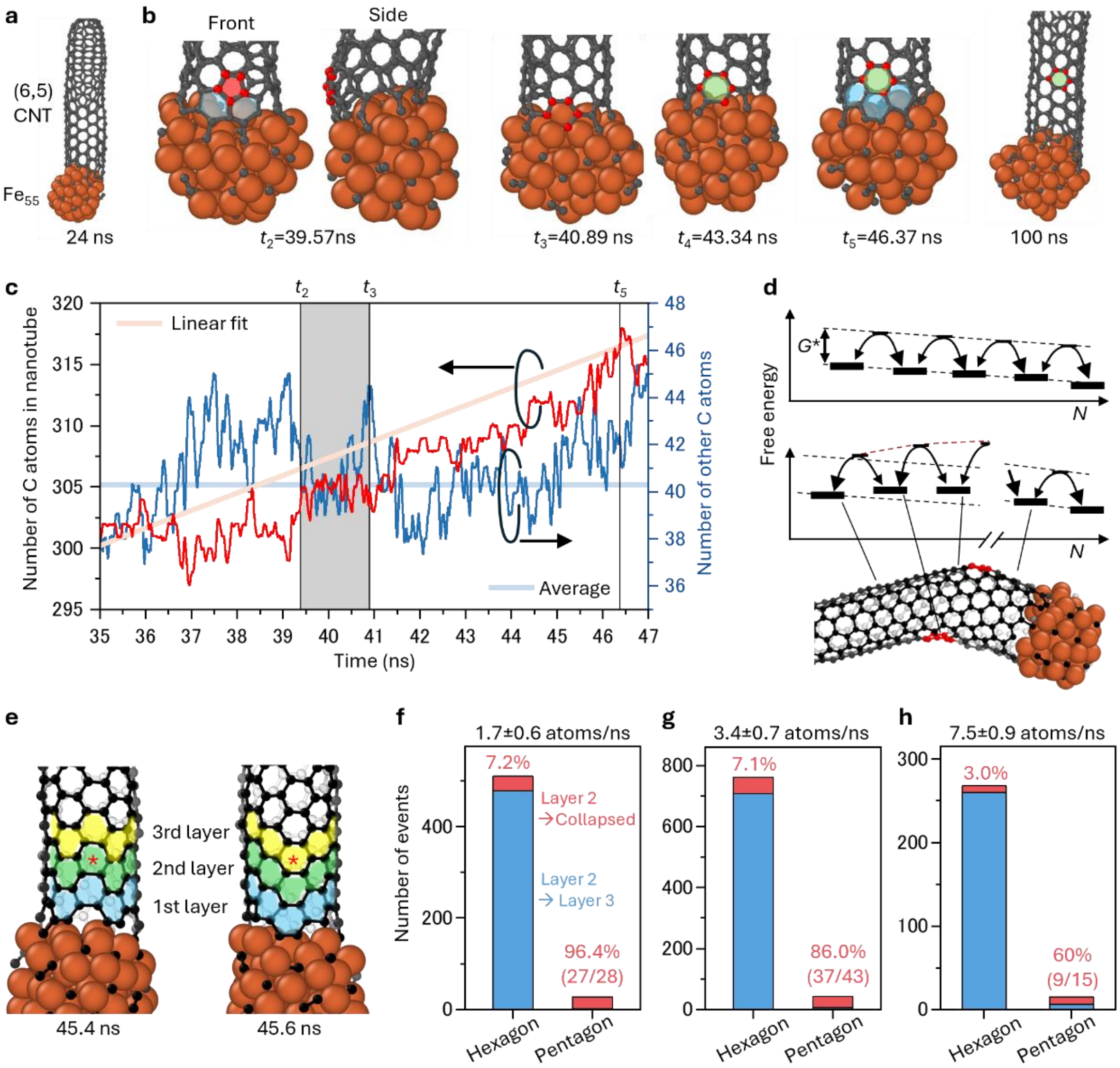

Supplementary Fig. 17 | Kinetic filtering of non-hexagonal defects at the tube–catalyst interface in MD simulations. a Snapshot of a CNT grown from an Fe_{55} catalyst. **b** Front and

side views of a representative event in which a pentagon (red) forms transiently near the tube edge and is subsequently removed through local reconstruction; additional hexagons formed during the event are highlighted in green and blue. **c** Time evolution of the number of carbon atoms incorporated into the nanotube (red) and other carbon atoms in the system (blue), showing that net elongation is suppressed while a pentagon persists (gray background). **d** Schematic free-energy landscape illustrating normal growth (top) and a defect-including pathway, where forward propagation is hindered and reversal becomes favorable. **e** Definition of layer depth from the tube–catalyst interface used for ring-tracking analysis. **f–h** Statistical outcomes for rings that reached layer 2 under different carbon supply rates. To increase the number of pentagon events, simulations were restarted using the same initial configuration that contains a pentagon in layer 2, with 15, 20, and 10 repeated trajectories using different random seeds.

Comment. *3. The use of the Arrhenius equation to calculate activation energy (Ea) is strictly valid only under steady-state conditions. However, the temperature-programmed growth process in this study is a non-steady-state process. During temperature evolving, the catalyst particles may undergo evolution (e.g., carbon solubility), meaning the pre-exponential factor (A) and the activation energy (Ea) of the system may be changed. For instance, temperature rise increases the carbon solubility in the catalyst, while temperature decrease causes a reduction in solubility and carbon precipitation. Can author provide some discussion or explanation the effect of non-steady-state process on activation energy (Ea)?*

Response. We completely agree that the extraction of activation energy is valid only under steady-state conditions. As our central focus is to experimentally support that the catalysts underwent coarsening at high temperatures (Figure 4), which explains the hysteresis in the "Arrhenius plots" of growth rates, we pay a careful attention to this point. Accordingly, we refer to the slope in Arrhenius plots as "Apparent activation energy (E_a). To avoid potential confusion for readers, we have added the following sentence in the main text.

On page 11, line 196

In such a case, projecting the growth rate evolution onto an Arrhenius plot requires caution, and we therefore refer to the extracted E_a values as apparent activation energies.

Comment. *4. The work demonstrates that a post-growth snapshot captures a catalyst particle that has coarsened long after the nanotube's diameter was fixed. Therefore, the widely reported 1:1 or 1:2 diameter ratio between the tube and its residual catalyst particle may be largely coincidental. Is it possible to discuss this point by estimating the expected post-growth catalyst size based on their coarsening model and comparing it to the as-grown tube diameters from their own data?*

Response. We appreciate the reviewer's insightful suggestion. In our system, post-growth AFM observations show that the catalyst particle size does not fall within the commonly reported 1:1

or 1:2 ratio relative to the nanotube diameter; instead, catalyst particles are often several times larger than the corresponding CNTs (see nearly added Supplementary Fig. 9). This behavior can be attributed to catalyst coarsening under our experimental conditions. For example, Navas et al. reported that, in post-growth TEM observations, many CNT termini lack both a cap and a catalyst particle, indicating that catalyst particles can shrink and eventually disappear during growth [*ACS Nano* **11**, 3081 (2017)]. Our system can be viewed as a complementary situation, in which catalyst particles instead evolve toward larger sizes during elongation.

In contrast, Nasibulin et al. reported a diameter ratio of approximately 1.6 under FCCVD conditions using a specific catalyst generation method that suppresses catalyst size evolution [*Carbon* **43**, 2251 (2005)]. This size ratio may reflect the intrinsic correlation that is determined at the nucleation as in a recent MD study by Hedman et al [*Nat. Commun.* **15**, 4076 (2024)]. However, their analysis is based on ensemble-averaged values of catalyst and CNT diameters, rather than one-to-one correspondence between individual nanotubes and catalyst particles. An example of individual tube-catalyst pairing is provided by He et al., where the diameter ratio is not confined to a narrow range such as 1:1 or 1:2, but instead exhibits a broad distribution [*Carbon* **113**, 231 (2017)]. While this variation was interpreted in terms of different growth modes, the analysis is based on post-growth snapshots and does not directly track the growth history of either the catalyst or the CNT. Therefore, the observed correlations do not uniquely determine the underlying mechanism. Based on our experimental observations, the relationship between catalyst size and CNT diameter might instead reflect growth histories after nucleation, including variations in growth onset, growth rate, and catalyst coarsening or disappearance.

Based on these considerations, we have revised the manuscript and Supplementary information to clarify that, in our system, the post-growth tube-catalyst diameter ratio does not converge to a single characteristic value, but is strongly influenced by dynamic processes during growth.

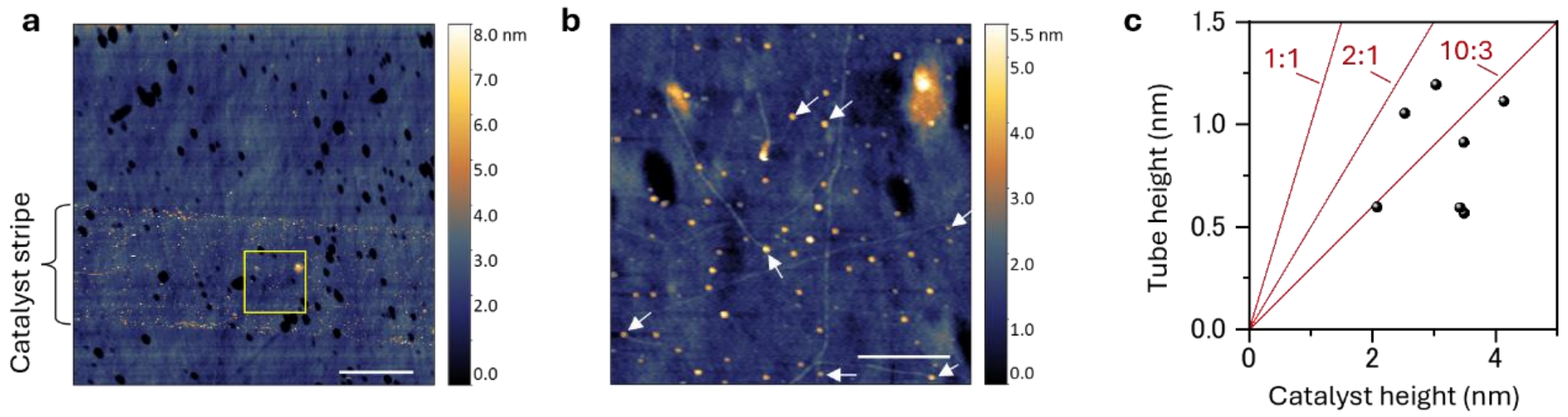

Supplementary Fig. 9 | AFM characterization on quartz. a, b AFM images of catalyst nanoparticles and CNTs grown at 800°C. Panel b was measured within the catalyst stripe (yellow box) in a. Scale bars are 2 μm and 500 nm in panels a and b, respectively. **c** Correlation of the heights of catalyst nanoparticles and the CNTs that grew from the catalysts. Solid lines represent the 1:1, 2:1, and 10:3 catalyst-tube height ratios.

Response to reviewer report for manuscript No. NCOMMS-26-008054A-Z by Otsuka et al.

We sincerely thank all the reviewers for taking their time to again review the manuscript and provide insightful comments. While the comments from the reviewers are written in ***italic blue***, our point-by-point response and revisions made are provided below in **black** and **red**, respectively.

Response to Reviewer #1:

Comment. *The authors tried to address the concerns raised by the reviewer, however, I am not convinced by the responses.*

Response. We sincerely thank the reviewer for taking time to evaluate our manuscript. Recognizing that our earlier framing might have been read as an attempt to replicate FCCVD conditions on a substrate, we have revised Abstract and Results to clarify our actual scope. The present study addresses two specific questions: (i) whether the chirality of an already nucleated CNT follows the catalyst size as the catalyst evolves, and (ii) whether chirality changes when the growth temperature is varied during elongation. Our intent is not to reproduce FCCVD conditions on a substrate, but to isolate these two well-defined questions in a controlled environment.

On page 6, line 103:
Among various key distinctions between supported- and floating-catalyst systems, we focus on a few variables specific to floating-catalyst systems—temperature and chemical potential—and impose them in a controlled manner on substrate-supported CNTs.

Comment. *1 As for the difference in the growth rates of CNTs by floating catalyst CVD and substrate supported CVD, the authors proposed that "growth rates (in µm/min) of supported-catalyst systems and floating-catalyst systems fall within the same order of magnitude if the temperature difference is taken into account. " Actually, the influence of substrate on the growth kinetics of CNTs must be taken into account, rather than simply comparing the activation energies.*

Response. We thank the reviewer for raising the role of substrate effects. The central question of our study —chirality robustness during elongation— requires careful consideration of substrate-induced perturbations, which we have therefore examined in detail (Supplementary Figs. 15 and 16). Continuum modeling and MD simulations both show that strong substrate–CNT van der Waals interactions can induce elliptical deformation and rather promote defect formation. The chirality robustness observed in our experiments arises despite conditions where substrate-induced perturbations could in principle have driven chirality changes; by implication, FCCVD systems should exhibit at least comparably robust preservation. We have also added Supplementary Table 5 to summarize the aspects common to, or distinct between supported and floating-catalyst growth.

Supplementary Table 5 | Similarities and differences between supported- and floating-catalyst CNT growth. For each aspect, the table compares the two platforms, marks whether the present work treats it as common, and recommends how to treat it when relating the systems.

Item	Supported catalysts	Floating catalysts	Treated as common in this work	Recommended treatment
Catalyst nucleation pathway	Via reduction, dewetting, and surface diffusion[44]	From volatile precursors in the gas phase before/during CNT growth[45,46]	No	Explicitly distinguis
Catalyst evolution pathways	Support-mediated ripening, diffusion into/along the support[47]	Gas-phase coalescence, evaporation, precursor-fed growth[25]	No (assuming catalyst growth in both cases)	Explicitly distinguis
Temperature, residence time, carbon supply path	Spatially stationary local environment; catalytic effect of supports exists[48]	Axial gradients, gas-phase transport only	No	Explicitly distinguis
Growth promoters	H_2O, CO_2, and H_2 are commonly employed to regulate catalyst formation and extend lifetime[49,50]	Sulfur, CO_2, and H_2 are often added[50–52], but their necessity is system-dependent (HiPco does not use S promoter[45])	No	Effects depend on tl growth stage at which they act[53]; no easily generalized
CNT nucleation	Following catalyst formation; nucleation density and diameter modulated by nucleation-stage conditions[54,55]	Coupled to gas-phase catalyst formation; Nucleation timing and diameter modulated by promoters[56]	Partly	Fundamental theori apply to both platforms[57–60]; catalyst-tube size usually correlates
Catalyst–CNT interface dynamics	Tip-growth and base-growth coexist[61]	Close to unsupported MD simulations[17,18,62]	Yes	Mechanisms should be shared with caution[63,64]
Structural robustness of CNTs after nucleation	Established for constant growth conditions, but not for evolving growth conditions	Open question for CNT ensembles; influence of sulfur is discussed theoretically[60]	Yes	Should be shared with caution
Elongation rate / kinetics	Measurable at the single-tube level; mostly 0.1–1 µm/s at 800–900°C	Direct measurement is challenging; estimated from length and residence time; influenced by promoters[65]	Mostly (Supp. Fig. 1)	Order-of-magnitude comparable at matched temperatur described within the same kinetic model
Substrate-induced deformation and alignment	Often important to achieve isolation and alignment; strong substrate interaction can induce defect formation (Suppl. Note 13 and 14).	Not relevant	No	Specific to supported-growth systems
Is catalyst phase constrained by support epitaxy?	Possible in some systems, but system-dependent[66]; no clear effect has been reported for Fe on quartz	Not relevant	No	Requires caution fo supported-catalyst cases

While other substrate effects (catalyst immobilization, catalytic roles of support itself) also matter for growth-rate kinetics, **our focus is *not how* catalyst particles change size in each synthesis platform**, but **whether catalyst-size changes redefine the structure of an already nucleated CNT** during elongation. At the tube–catalyst growth front —where carbon incorporation, precipitation, interfacial reconstruction, and defect formation/healing take place— the same elementary processes operate in both systems. Reflecting this, MD and DFT studies of CNT growth are routinely cited in interpreting both supported and floating-catalyst experiments, and

the experimental literature on the two platforms actively cross-references each other [*Carbon* **219**, 118747 (2024); *Carbon* **195**, 92 (2022); *Nano Research* **16**, 12720 (2023)]. The empirical agreement in growth rates across the two platforms at a given temperature —despite differences in pressure, carbon source, and growth promoters— further indicates **that the effective carbon supply and precipitation kinetics at the growth interface fall within a comparable kinetic window**.

Comment. 2 Regarding the role of sulfur growth promoter, the authors argued that "its quantitative impact on CNT properties is generally moderate rather than dramatic. " This is absolutely not true. In most FCCVD growth cases, the use of a small amount of sulfur plays a crucial role determining the yield, purity, growth rate and diameter of the CNTs obtained. The example raised by the authors [Chemical Engineering Journal 378, 122010(2019)] is not a representative case.

Response. We agree with the reviewer that sulfur-containing species, including thiophene, can have a substantial impact on CNT growth in many FCCVD systems. We have removed the discussion referring to [*Chemical Engineering Journal* **378**, 122010 (2019)] and added a sentence in the Introduction. In addition, excessive sulfur addition tends to enlarge nucleated tube diameter and favor multi-walled structures [*Carbon* **210**, 118051 (2023)], which falls outside the scope of our discussion on nanotube chirality. As our discussion targets single-walled CNTs primarily for semiconducting applications, we now use "SWCNTs" wherever the single-walled nature is important and apparent, including the second revision below.

> On page 3, line 44:
> Growth promoters, such as sulfur[17] and CO_2[18], commonly employed in these systems may also influence the catalyst evolution processes.
>
> On page 3, line 33:
> Consequently, single-walled CNTs (SWCNTs) are commercially produced primarily via floating-catalyst CVD (FCCVD), such as HiPco and eDIPS[9,10], providing source materials for semiconductor applications including transistors[11] and single photon emitters[12].

However, the presence of sulfur does not by itself make FCCVD mechanistically incomparable with substrate-supported CVD. Growth promoters and mild additives are also widely used in substrate-supported CNT growth; in some conditions, oxygen, water, and CO_2 can strongly enhance CNT yield, catalyst lifetime, and the accessible growth window [*Science* **306**, 1362 (2004)]. Thus, strong sensitivity to a small amount of additive is not unique to FCCVD, but reflects a broader feature of CNT growth: catalyst activity, carbon activity, and deactivation pathways must be kept within a suitable window.

Moreover, sulfur is not a universal prerequisite for FCCVD growth. Not a few FCCVD systems, including CO-based processes (e.g., HiPco or *Chemical Engineering Journal* **476**, 146528 (2023)), ethylene-based aerosol CVD [*Nanoscale* **10**, 9752 (2018)], and other systems [*Scientific Reports* **4**, 3903 (2014)] can produce high-quality CNTs without sulfur addition. To clarify this point experimentally, we have also added new control data obtained from our sulfur-free FCCVD

process to the Supporting Fig. 13 and Supplementary Note 10, showing that high-quality CNTs can be obtained under appropriate precursor chemistry. The weak dependence of average diameter on furnace set temperatures is also confirmed for the sulfur-free FCCVD.

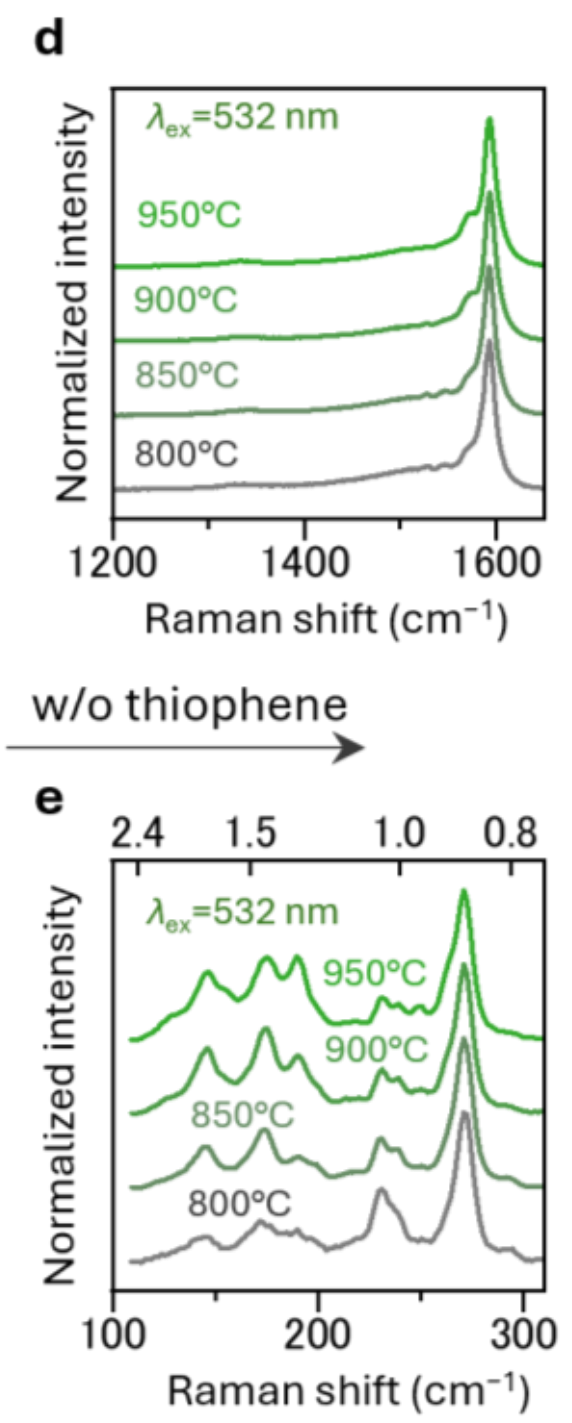

In addition, we also synthesized CNTs without thiophene promoter at four different furnace set temperatures (Supplementary Fig. 13d,e). Although diameter distribution is different from the case with thiophene, which might be influenced by the total pressure and the condition of quartz tube wall, high-quality CNTs with G/D ratio of ~50 were obtained, exhibiting a weak temperature dependence of average diameter (1.1 nm and 1.2 nm for T_{set}=800°C and 950°C cases, respectively).

Supplementary Fig. 13 | d,e Raman spectra of CNTs grown without thiophene using an excitation wavelength of 532 nm.

Most importantly, the sulfur-related effects discussed in the literature mainly concern catalyst-particle formation, catalyst activation, carbon encapsulation, cap lift-off, wall-number selection, and yield [*Carbon* **210**, 118051 (2023)]. These effects do not demonstrate that, after a CNT has nucleated, its diameter or chirality continuously follows subsequent changes in catalyst size. Therefore, they do not invalidate the central implication of our study: once CNT diameter and chirality are established at nucleation, they can remain robust during subsequent catalyst evolution, while growth kinetics adapt. This distinction is particularly relevant to FCCVD, where catalyst particles evolve continuously in nonuniform temperature and chemical-potential fields. Our results therefore suggest that FCCVD diameter distributions should be interpreted in terms of the catalyst-state distribution at the moment and location of nucleation, rather than only from post-growth catalyst snapshots.

Comments. Except for these above issues relating to some specific conditions, actually, my concern is that the current work does not present a general breakthrough in the understanding of the growth of CNTs. Thus, I think it is suitable for publication in more specialized journals, rather than in NC.

Response. We understand that the reviewer's concern is the lack of a general breakthrough in the understanding of the growth of CNTs, and this point is main concern for the reviewer rather than these above issues. We address this directly, as it is central to the value of our work. We agree at the outset that this work proposes no new recipe for chirality-controlled synthesis; its advance lies in understanding. We summarize this advance along three lines.

1. A quantitative framework that separates structure determination from elongation. Our central finding is not merely that chirality is preserved after nucleation, which is a basis for understanding diameter determination in FCCVD systems, but that it is preserved *while* the growth rate and lifetime respond systematically to the evolving catalyst —a response we measure directly on individual tubes. Because we resolve both the robust structure and the adaptive kinetics on the same nanotubes, and integrate them with the kinetic model we established [*ACS Nano* **16**, 5627 (2022)], the separation is not a qualitative statement that "structure and yield can be optimized independently," but a kinetic framework specific enough to prescribe *how* to maximize yield: for example, catalyst coarsening shortens the growth lifetime, and our quantitative analysis indicates how the precursor/promoter supplies can be designed to make growth robust against such changes and to match the intrinsic lifetime to the reactor residence time. Separating structure determination from elongation is therefore an actionable principle, not a slogan.

2. A re-examination of the catalyst–CNT structural correspondence. The robustness we observe forces a reconsideration of how catalyst and tube structures are related. The diameter and chirality of a tube are commonly read from ex situ catalyst–tube correlations, but if the structure is a frozen record of nucleation and does not follow the subsequent catalyst evolution, such static snapshots can be systematically misleading. A rigorous account of chirality determination—and ultimately its control—therefore requires a dynamic view: observation and understanding of the growth interface as it evolves, rather than post-growth snapshots.

3. A trigger for a more general discussion of one-dimensional crystal growth. That a quasi-1D crystal retains its structure so robustly is itself informative for related materials. In VLS nanowires, the diameter readily follows fluctuations in catalyst size [*Nat. Commun.* **7**, 12271 (2016)]; in CNTs, the closed graphitic lattice keeps the structure fixed. Identifying these as opposite regimes—set by the structure of the growth interface—reframes a question beyond CNTs: why catalyst-templated growth tracks the catalyst in some crystals and remembers it in others. Our work provides an experimental entry point to that general question.

We have revised the entire Abstract, as well as the following paragraphs in Introduction and Conclusion to make these three points explicit.

> On page 4, line 63:
> Since any change in nanotube diameter requires topological defect incorporation, consistency with the literature on high-crystallinity SWCNT growth warrants examination. Defect formation is in practice governed by kinetics rather than equilibrium thermodynamics[27]. Under

steady growth conditions, chirality preservation along SWCNTs over hundreds of micrometers has been well reported[28–30], further supported by recent MD simulations through the modeling of defect-healing kinetics[31,32]. These supports for robustness, however, do not rule out structural adaptation under dynamically evolving conditions: these modeling assume planar interfaces[27] or fixed interfaces[31], leaving the effects of tube–catalyst mutual curvature and contact geometry unresolved.

Whether the structure of an individual SWCNT is adaptive or robust as temperature and catalyst size vary during elongation therefore calls for direct experimental resolution.

On page 5, line 81:

Here, we combine digital isotope labeling[38] with controlled thermal cycling to investigate chirality preservation in individual SWCNTs under dynamic environments, with the growth rate history serving as a probe of catalyst-size evolution over hundreds of micrometers. The experimental observations are integrated with kinetic Monte Carlo simulations of stochastic catalyst coarsening and MD simulations of SWCNT growth, providing complementary perspectives across length and time scales. Our results reveal robust structural memory alongside adaptive kinetics, establishing a rigorous baseline for mechanistic discussion of chirality determination and a foundation for practical strategies that integrate structural control with scalable growth.

One page 24, line 463:

In conclusion, by tracking individual CNTs under dynamically evolving environments, we have established that diameter and chirality, set at nucleation, are robustly preserved during subsequent elongation, while growth rate responds sensitively to environmental fluctuations. The pronounced temperature dependence and hysteresis of growth rates reflect irreversible catalyst Ostwald ripening rather than intrinsic changes in the growth mechanism, as supported by MD simulations of SWCNT growth from enlarging catalysts. This contrast between structural robustness and adaptive kinetics carries three consequences: it provides a design principle that decouples chirality control from yield optimization across separate growth stages; it requires reinterpretation of ex situ CNT–catalyst diameter ratios in terms of the catalyst's temporal evolution rather than static post-growth snapshots; and it suggests a strategy for catalyst-mediated 1D crystal growth: preserving structural fidelity at a reconfigurable interface while allowing kinetic flexibility during elongation. These consequences establish a framework for connecting atomic-scale structural understanding to scalable strategies for structure-controlled synthesis.

On page 21, line 410:

Even vapor-liquid-solid 1D nanowires adapt their diameter to the evolving catalyst geometry[55], indicating the importance of interface structure.

Response to Reviewer #2:

Comment. *In their response, the authors devote considerable effort to emphasizing the novelty of their manuscript. After carefully reviewing both the manuscript and the replies, I find myself largely in agreement with the first reviewer: CNT growth via FCCVD differs fundamentally from substrate-supported growth, particularly in terms of nucleation behavior and growth kinetics.*

Response. We sincerely thank the reviewer for reviewing our revised manuscript and reading through our response. In response to the comments, we have revised the manuscript to convey our claims more clearly. As for the additional viewpoint, please refer to our responses to Reviewer #1. Briefly, the two systems share both common and distinct features, which we have organized in Supplementary Table 5. The structural robustness concerns the stage *after* nucleation, so the difference in nucleation behavior does not affect our conclusions, while the kinetics at the CNT–catalyst interface are shown to be common to both systems.

Comment. *Notably, the present work does not introduce any new experimental additives or strategies for FCCVD.*

We agree that this work does not propose a new additive molecule for FCCVD. We would argue, however, that the structural-memory framework itself prescribes a concrete design strategy for FCCVD growth, including how known additives such as CO_2 should be deployed. Because the diameter and chirality are fixed at nucleation and then preserved during elongation, the gas-phase carbon activity a_C can be tailored spatially along the reactor in a stage-specific manner: a high a_C near the nucleation zone favors small-diameter nucleation, while a a_C-lowering species (CO_2) introduced downstream during elongation lower the growth rate while extending lifetime. Importantly, FCCVD imposes a finite residence time absent in substrate-supported growth, so length is maximized by designing the a_C profile such that the intrinsic growth lifetime matches this residence time. Influence of catalyst size change (larger particles reach higher μ at a fixed a_C) should also be considered to design optimal a_C profile. Such stage-resolved control, which builds on the rate–lifetime relationship of the kinetic model in Supplementary Note 7, directly follows from our finding and is not available from ensemble abundance arguments that contain no time variable. We have modified the Discussion part and added the following paragraph to propose potential strategies derived from the present study.

On page 23, line 434:

These considerations suggest that intentional perturbations of the growth environment at the reactor inlet, including precursor concentration and temperature gradients, could be used to confine the nucleation zone, potentially narrowing the diameter distribution. Complementarily, the same separation of structural memory from elongation allows the gas-phase carbon activity[61] along the reactor to be designed independently to maximize length. We have seen that the catalyst chemical potential μ, which depends on its size and the carbon activity, governs both the growth rate γ and the intrinsic growth lifetime in opposite directions (Fig. 3, Supplementary Note 7). Final CNT length is then γ integrated over this lifetime, also limited by the extrinsic residence time. An optimal carbon-activity profile therefore depends on the reactor geometry, as well as

temperature profile and catalyst evolution along the flow, reflecting a design constraint distinct from that in substrate-supported growth.

Comment. *In addition, the authors attempt to decouple structural memory from kinetic adaptability in CNT growth, a concept that is already well established and widely accepted within the CNT research community. On this basis, I am unable to recommend the manuscript for publication. My specific comments on the authors' responses are as follows:*

We wish to clarify that **the concept of our study —the structural robustness of individual nanotubes under dynamic conditions— is fundamentally distinct from the ensemble chirality-abundance framework of Yakobson et al.** (see our response to Comment 6 for a detailed comparison).

Comment 1. The authors state that CNT growth times in FCCVD typically range from several seconds to tens of seconds. However, commercially produced CNTs from the HiPco process are only a few micrometers in length. Considering the high growth rates at elevated temperatures, this suggests that the actual growth time is likely shorter than 1 s.

Response. We thank the reviewer for highlighting the HiPco process as a specific test case. We agree that the actual growth time in HiPco is likely shorter than 1 s, given the few-micrometer length of HiPco-derived CNTs and high growth temperatures.

However, sub-second growth times do not by themselves imply negligible catalyst evolution. As we have already mentioned in the previous response, catalyst-particle growth and CNT elongation are both governed by temperature and precursor availability. Indeed, Hoecker et al. [*Carbon* **96**, 116 (2016)] directly measured catalyst nanoparticle size distributions along an FCCVD reactor and showed (Fig. 4 therein) that the particle size distribution and concentration change substantially over short axial distances, corresponding to residence-time differences on the order of ~1 s under their flow conditions. Catalyst particles thus evolve noticeably on sub-second-to-second timescales even at moderate precursor loadings.

This point is even more relevant to HiPco. While Hoecker et al. used approximately 0.5 mass% ferrocene at atmospheric pressure, corresponding to a nominal ferrocene partial pressure of ~ 0.04 Torr, the preferred catalyst-precursor partial pressure in the HiPco patent is 1–10 Torr, which is ~**25–250 times higher** than the case of Hoecker et al. Since precursor impingement flux scales approximately with partial pressure, catalyst-particle growth, coalescence, and restructuring in HiPco are expected to proceed even faster. The expected short growth time in HiPco therefore does not rule out significant catalyst evolution during that interval.

Comment 2. To support their discussion on catalyst nanoparticle coalescence, the authors cite prior studies (ACS Nano 11, 3081 (2017); JPCL 1, 918 (2010)). However, these works also report that catalyst coalescence can lead to catalyst deactivation. In contrast, the authors claim that CNT growth continues even after particle coalescence, which appears inconsistent with the cited literature.

Response. We thank the reviewer for raising the important point, which prompted us to perform additional analysis specifically addressing coalescence (Supplementary Fig. 19, detailed below). Here, we interpret the Reviewer's comments (Comments 2 and 3) as follows:

(a) Particle coalescence has often been associated with catalyst deactivation.

(b) Direct ETEM evidence for post-nucleation coalescence of active catalysts is limited.

First, we would like to clarify that in our experiment for supported-catalyst growth, **Ostwald ripening, rather than particle coalescence, is assumed to be the primary mechanism behind catalyst coarsening**. In relation to point (a), we agree that particle coarsening —whether through coalescence or Ostwald ripening— can eventually lead to catalyst deactivation through encapsulation by carbon. Importantly, our experiments and interpretation are **actually consistent** with the observation by Navas et al. (ACS Nano:2017), where large catalyst particles were encapsulated by carbon layers at intermediate and long growth durations. Therefore, we have stated in the previous submission: "*In addition, this framework is consistent with the TEM observation by Navas et al.*[19]*, where a significant evolution of the particle size distribution and encapsulation of large catalyst particles by carbon layers were observed*" (in Supplementary Note 7). **The apparent inconsistency arises from the difference between snapshot observations and time-resolved tracking using isotope labeling**.

In our model, catalyst coarsening has two coupled consequences: it increases the CNT growth rate by increasing the effective carbon supply, but at the same time it increases the probability of catalyst deactivation through carbon overlayer formation due to increased chemical potential. This point is discussed in Supplementary Note 7 in detail, where growth termination is modeled in terms of catalyst surface passivation by a graphene-like overlayer, and the same catalyst-coarsening picture is used to explain both the growth-rate hysteresis and the lifetime hysteresis.

Experimentally, this interpretation is supported by the data shown in Fig. 3: after the catalyst has evolved at higher temperature, the CNT growth rate becomes higher, whereas the survival probability becomes lower. Consequently, the growth of characterized CNTs was mostly terminated before 900 s, when ethanol supply was stopped. Thus, our emphasis is not that CNT growth continues after catalyst coarsening, but that **catalyst coarsening is a continuous process that can transiently accelerate CNT elongation speed while progressively increasing the stochastic probability of catalyst deactivation**. <u>We have revised the text to clarify this point and to avoid the impression that catalyst coarsening does not influence the lifetime of catalysts</u>.

On page 10, line 172:
Consistently, most CNTs had already terminated growth before the carbon feed was stopped (Fig. 2e), indicating that accelerated elongation is accompanied by shortened growth lifetime.

To further address the reviewer's concern about particle *coalescence*, we additionally performed MD simulations as a stronger catalyst-size perturbation than continuous coarsening. When a carbon-dissolved catalyst particle merged with an active catalyst, CNT growth continued while preserving the original nanotube structure. By contrast, when the incoming particle was encapsulated by a carbon shell, excessive carbon supply induced secondary graphitic nucleation and terminated the original CNT growth. As the reviewer pointed out, particle coalescence can therefore lead to deactivation, although it does not inevitably terminate CNT growth in all cases. Details have been added in Supplementary Fig. 19.

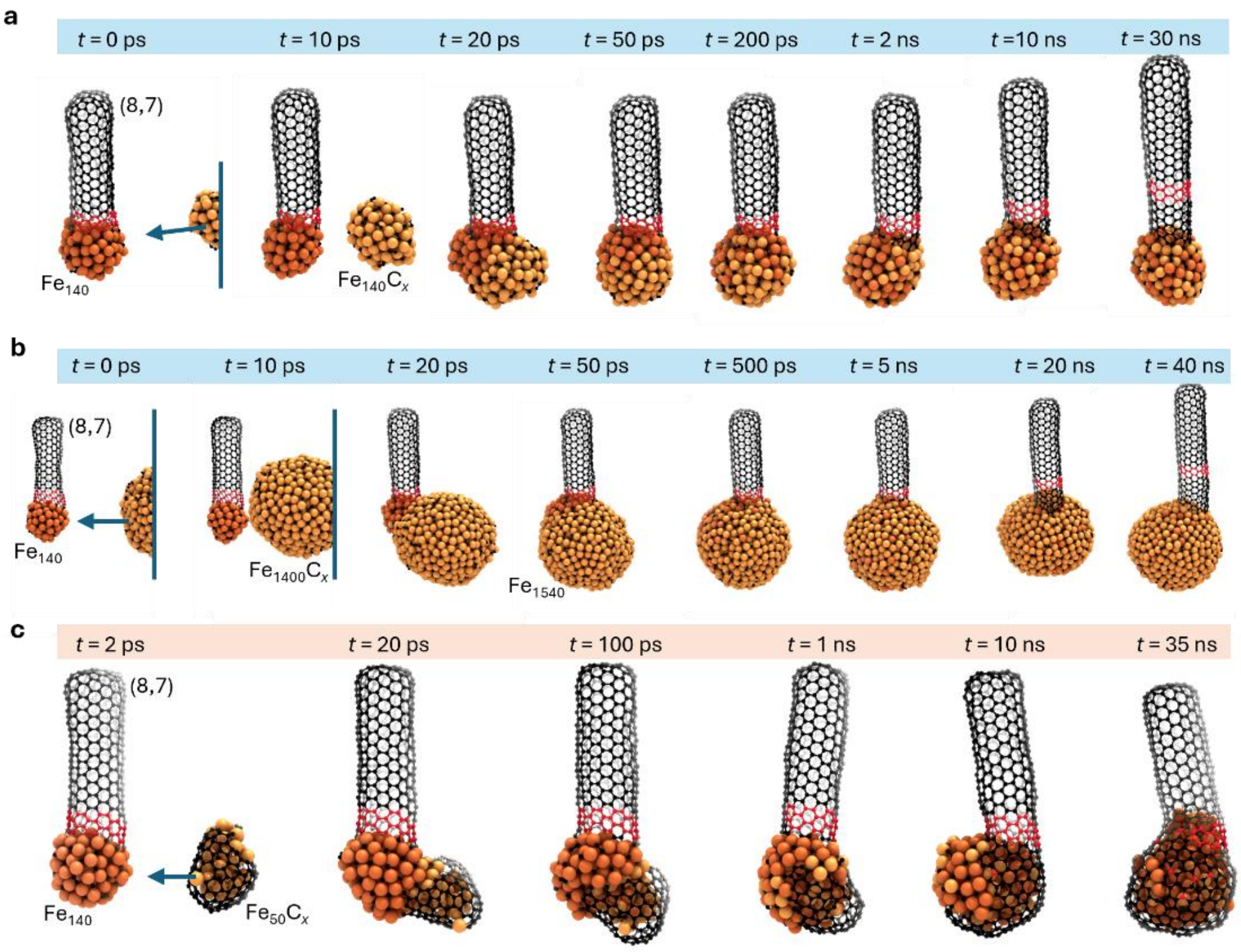

Supplementary Fig. 19 | Catalyst coalescence during CNT growth. a Snapshots of an (8,7) CNT attached to an Fe_{140} catalyst particle before and after coalescence with an active $Fe_{140}C_x$ particle. The CNT grew whhile maintaining its structure after catalyst coalescence. **b** Snapshots of an (8,7) CNT attached to an Fe_{140} catalyst particle before and after coalescence with a much larger active $Fe_{1400}C_x$ particle, showing chirality preservation despite the large catalyst-size mismatch. **c** Snapshots of an (8,7) CNT attached to an Fe_{140} catalyst particle during coalescence with a carbon-encapsulated, deactivated $Fe_{50}C_x$ particle. The incoming carbon shell/graphene-like nucleus was transferred to the active catalyst surface and induces passivation of the growth interface. These results suggest that catalyst deactivation is governed not simply by catalyst-size evolution, but by whether a new graphitic carbon nucleus is introduced onto the active catalyst surface. Temperature was kept at 1400 K.

Comment 3. Over the past two decades, numerous in situ ETEM studies have examined CNT growth from individual catalyst particles (e.g., Nano Lett. 2008, 8, 2082; Science Advances 2022, 8, abo5686; as well as extensive work from the groups of Renu Sharma at ASU and Stephan Hofmann at the University of Cambridge). Across these studies, particle coalescence is generally not observed after CNT nucleation. Therefore, the authors' assertion that CNTs can continue growing following particle coalescence lacks convincing experimental support.

Response. We thank the reviewer for the references to the important ETEM literature, including the work of the Sharma and Hofmann groups. This motivated us to systematically map where these studies and our isotope-encoded growth analysis access different temperature regimes (see Table R1 below).

We interpret this comment to be associated with the point (b). We agree that post-nucleation *coalescence* of active catalyst particles has rarely been reported in single-CNT in situ ETEM observations. However, the absence of such observations should be distinguished from evidence that the process cannot occur. ETEM and our approach access complementary regimes —atomic-scale dynamics under low temperature/pressure *versus* 100-µm-scale growth under practical growth conditions— so the absence of observation in ETEM does not directly contradict our findings. For example, **extensive ETEM studies including those by Shama et al. and Hofmann et al. were usually performed at 450–650°C** (Table R1), which are much lower than our experiment (800–873°C). When the same KMC simulation is applied to the low-temperature case (600°C), catalyst coarsening is negligible over few-hour time scale (Figure R1), in good contrast with the temperature range of our experiment. A brief comment on a temperature constraint of *in situ* TEM and mentioned references have been added in the Introduction.

> On page 5, line 76:
> Although in situ TEM can probe catalyst nanoparticles during active nanotube growth[33–37], its intrinsic constraints, including the general requirement for low pressure/temperature and …

In addition, as noted in our previous response, we further emphasize that, if most of the active catalyst surface remains exposed, as suggested by our AFM observations (catalysts are much larger than CNTs), there is no general mechanism by which the active particle would be protected from metal-atom flux generated by restructuring or Ostwald ripening of neighboring particles. Carbon encapsulation or strong CNT–catalyst interfacial pinning could suppress metal uptake, but such mechanisms require substantial surface coverage or passivation, which is inconsistent with the growth of CNTs. Thus, while the exact timing of coarsening cannot be determined from post-growth AFM alone, assuming an invariant active catalyst size is also not justified.

Table R1 | Representative in situ TEM/ETEM studies of CNT growth and catalyst dynamics. The table summarizes representative single-particle in situ TEM/ETEM studies, highlighting the catalyst, carbon source, temperature, and support. These studies demonstrate the power of in situ observation for resolving CNT nucleation, growth, and catalyst dynamics, but also show that each observation is tied to a specific catalyst composition, support geometry, temperature, pressure, and imaging window.
† Catalyst splitting and Ostwald-ripening-associated growth-termination events were observed during near-ambient-pressure MWCNT growth.
‡ W-based high-melting-point alloy catalyst.

**Catalyst**	**Carbon source**	**Temperature**	**Support**	**Authors (First/ Last)**
Ni/SiO_2	C_2H_2	475–480°C	SiO_2	R. Sharma / M. M. J. Treacy [i]
Ni, Fe	C_2H_2; NH_3/C_2H_2 for CNF	360–615°C depending on observation	SiO_x membrane	S. Hofmann / J. Robertson [ii]
Fe_3C / Fe on SiO_2	C_2H_2	600°C	SiO_2	H. Yoshida / Y. Homma [iii]
Ni	NH_3/C_2H_2	477°C	SiO_x membrane / SiO_x-coated TEM grid	M. Moseler / A. C. Ferrari [iv]
Fe_3C active / Fe_5C_2 inactive	C_2H_2	630°C	perforated SiO_x thin film	S. Mazzucco / R. Sharma [v]
Au-doped Ni / Ni_3C	C_2H_2	520°C	not specified here	R. Rao / B. Maruyama [vi]
Fe catalyst	C_2H_4/H_2/He	800°C †	SiN membrane	J. Huang / M.-G Willinger [vii]
Co	acetate-derived carbon species	600°C	TEM grid / thin support	F. Yang / Y. Li [viii]
Co–W–C / η-carbide alloy ‡	$C_2H_4 + H_2$	850°C	SiN_x membrane	Y. Wang / H.-M. Cheng [ix]

[i] Sharma, R., Rez, P. & Treacy, M. M. J. Direct Observations of the Growth of Carbon Nanotubes using in situ Transmission Electron Microscopy. *e-Journal Surf. Sci. Nanotechnol.* **4**, 460–463 (2006).
[ii] Hofmann, S. *et al.* In situ Observations of Catalyst Dynamics during Surface-Bound Carbon Nanotube Nucleation. *Nano Lett.* **7**, 602–608 (2007).

[iii] Yoshida, H., Takeda, S., Uchiyama, T., Kohno, H. & Homma, Y. Atomic-Scale In-situ Observation of Carbon Nanotube Growth from Solid State Iron Carbide Nanoparticles. *Nano Lett.* **8**, 2082–2086 (2008).
[iv] Moseler, M., Cervantes-Sodi, F., Hofmann, S., Csányi, G. & Ferrari, A. C. Dynamic Catalyst Restructuring during Carbon Nanotube Growth. *ACS Nano* **4**, 7587–7595 (2010).
[v] Mazzucco, S. *et al.* Direct evidence of active and inactive phases of Fe catalyst nanoparticles for carbon nanotube formation. *J. Catal.* **319**, 54–60 (2014).
[vi] Rao, R., Sharma, R., Abild-Pedersen, F., Nørskov, J. K. & Harutyunyan, A. R. Insights into carbon nanotube nucleation: Cap formation governed by catalyst interfacial step flow. *Sci. Rep.* **4**, 6510 (2014).
[vii] Huang, X., Farra, R., Schlögl, R. & Willinger, M.-G. Growth and Termination Dynamics of Multiwalled Carbon Nanotubes at Near Ambient Pressure: An in Situ Transmission Electron Microscopy Study. *Nano Lett.* **19**, 5380–5387 (2019).
[viii] Yang, F. *et al.* Growth modes of single-walled carbon nanotubes on catalysts. *Sci. Adv.* **8**, eabq0794 (2022).
[ix] Wang, Y. *et al.* Growth mechanism of carbon nanotubes from Co-W-C alloy catalyst revealed by atmospheric environmental transmission electron microscopy. *Sci. Adv.* **8**, eabo5686 (2022).

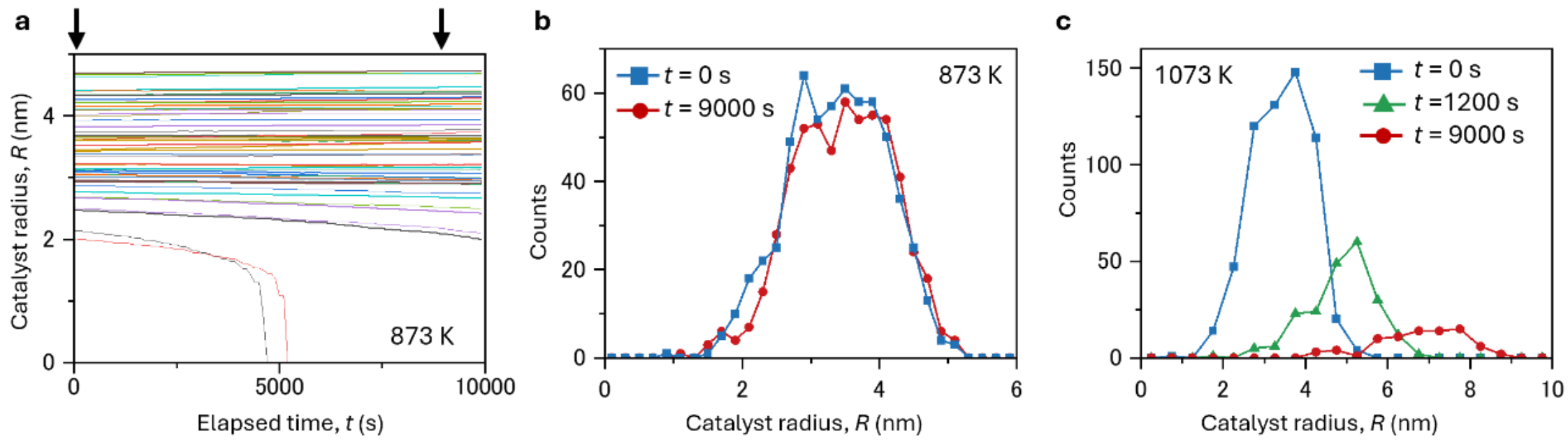

Figure R1 | Kinetic Monte Carlo simulations at low temperatures. **a**, Evolution of individual catalyst sizes obtained from KMC simulations at 873 K. All parameters except for temperature are the same as the simulation shown in Fig. 4. **b**, Corresponding histograms of catalyst radii R at $t = 0$ and 9000 s, showing only a slight change in size and number. **c**, Histograms of catalyst size at $t = 0$, 1200, and 9000 s after KMC simulations at 1073 K.

Comment 4. Although AFM images are presented to suggest that catalyst particles increase in size after CVD growth, it remains unclear whether this enlargement occurs before or after CNT nucleation. Furthermore, the formation of amorphous carbon coatings could also contribute to apparent particle growth, particularly after catalyst deactivation.

Response. We fully agree that post-growth AFM observations alone cannot determine whether catalyst enlargement occurred before or after CNT nucleation, and that amorphous carbon coating may contribute to the apparent particle size after deactivation. For this reason, we did not use AFM as the primary evidence for catalyst evolution during growth. Our major evidence instead comes from isotope-encoded growth histories of individual CNTs, which reconstruct the temporal evolution of elongation rate together with structural information along the same nanotubes. The connection between observed growth rate change and catalyst coarsening is supported from (1) kinetic model established in [*ACS Nano* **16**, 5627 (2022)], (2) LSW-type Ostwald ripening model, (3) kinetic Monte Carlo simulations for Ostwald ripening, and (4) MD simulations of CNT growth from growing catalyst; the AFM data are presented only as additional evidence that catalyst particles can evolve under relevant CVD conditions. Direct in situ catalyst imaging under the same temperature, substrate, and pressure conditions would be valuable, but is beyond the scope of the present study. This limitation is already explicitly stated in Supplementary Note 9.

Independently of these models, the irreversibility of the growth itself supports catalyst evolution. At nearly identical temperatures, individual SWCNTs grow faster during ramp-down than ramp-up, and this hysteresis quantified for each individual tube grown on the same substrate in Table R2 (acceleration factors of ~1.1–2.1 at ~849°C). Because this hysteresis cannot arise from a reversible temperature dependence, and its magnitude varies markedly from tube to tube under identical furnace conditions, it cannot originate from system-wide factors (e.g., furnace drift or temperature error) and instead reflects irreversible, catalyst-specific evolution along each nanotube. While this is not the only conceivable interpretation, we are aware of no alternative mechanism that accounts for the hysteresis and its tube-to-tube variation, and would welcome one.

Table R2 | Growth rate hysteresis within individual SWCNTs. Instantaneous growth rates of SWCNTs, which grew across the maximum temperature and show hysteresis (Supplementary Fig. 6a, bottom right), and the acceleration factors of each nanotube at nearly identical temperatures during *T* ramp-up and ramp-down periods.

**Center *T* (°C)**<br>**Center *t* (s)**	**837.1 (up)**<br>**375**	**850.1 (up)**<br>**435**	**847.9 (down)**<br>**645**	**838.5 (down)**<br>**675**	**Ratio (~849°C)**	**Ratio (~838°C)**
**#r1**	4.7	9.6	11.7	8.3	1.21	1.76
**#r2**	4.2	6.4	11.7	12.2	1.83	2.89
**#r3**	7.5	11.6	12.9	11.9	1.11	1.58
**#r4**	17.5	28.4	37.6		1.32	
**#r5**		18.4	38.0		2.06	
**#r6**		10.6	17.1	20.5	1.62	
**#r7**	11.2	15.6	28.6	21.1	1.83	1.88
**#r8**		18.2	27.2	20.8	1.50	

Comment 5. Regarding the previously mentioned work (Chem, 5, 1182–1193 (2019); corrected citation), it is shown that chirality transitions require the incorporation of pentagon-heptagon defect pairs, associated with energy barriers exceeding 3 eV. This supports the conclusion that CNTs are likely to preserve their initial structure once nucleated.

Response. We thank the reviewer for providing us with information on the related work. We have carefully read through the paper, and found that this mentioned work does not, in fact, support structural preservation of CNTs. Rather, the authors synthesized the (10,9) tubes with 80% abundance and proposed a mechanism in which **the chirality of (*n*,*n*) tubes tends to change to (*n*,*n*-1)** compared to changes from (*n*,*n*) to (*n*,*n*-2) or (*n*+1,*n*), based on the "energy barriers" among several chirality pairs. The reported barriers characterize the relative ordering of chirality-transition pathways, not absolute chirality-change frequencies or defect densities; their magnitude, even >3 eV, only indicates which transitions are favored if they occur.

More fundamentally, chirality-change events cannot be explained from the viewpoint of equilibrium thermodynamics alone. **Defect formation that induces chirality change is governed by kinetics**, including the CNT growth rate: chirality can change more easily at higher growth rates, consistent with the existence of defective CNTs reported under certain synthesis conditions. The theoretical analysis by Yuan et al. [*PRL* **108**, 245505 (2012)] addresses this regime, but does not incorporate the effects of CNT–catalyst mutual curvature and contact geometry, as well as their temporal change, which are central to our study through both experimental observations and MD simulations.

Comment 6. In addition, prior theoretical work (Nat. Commun. 2024, 5, 4892) from Yakobson's group has already decoupled nucleation thermodynamics from growth kinetics in CNT formation. This concept has been extensively explored in efforts to achieve chirality-specific synthesis. Therefore, the claimed novelty of the present manuscript is not sufficiently convincing.

Response. We thank the reviewer for the important prior theoretical work by Yakobson and co-workers [*Nat. Commun*. **5**, 4892 (2014)]. With this reference, we now understand that the reviewer is concerned that our discussion of structural memory and growth kinetics may appear to re-present the established concept that nucleation thermodynamics and growth kinetics can be treated as distinct factors in CNT chirality selection.

We would like to clarify that **the concept addressed in the prior work and that addressed in our manuscript are fundamentally different**; whether one concept holds does not determine whether the other holds. Yakobson et al. considered **the ensemble abundance of CNT chirality**, in which the abundance $A(\chi)$ of CNTs with a given chiral angle χ is expressed as the product of the chirality-dependent nucleation probability $N(\chi)$ and the chirality-dependent growth rate $R(\chi)$. Their framework therefore explains which chiral indices are statistically enriched in a CNT population ($A(\chi)=N(\chi)R(\chi)$). *By contrast*, our study addresses **a post-nucleation question at the level of individual nanotubes**: once a CNT with a given diameter and chirality has nucleated, whether that structural identity is preserved during subsequent elongation *under dynamically evolving growth environments*. Using isotope labeling, we directly reconstructed the growth history of individual CNTs and compared, within the same nanotube, the structural continuity

with the response of growth rate to temperature and catalyst evolution. Another important distinction is that the prior abundance model is essentially a chirality-dependent statistical model and does not consider **time-dependent growth histories**; *i.e*, their model does not explicitly contain a time variable *t*.

Thus, the contrast between structural robustness and kinetic adaptability in our manuscript is not a re-presentation of the established theory of chirality selection. The novelty of our work therefore lies not in separating nucleation and growth as factors controlling ensemble chirality distribution, but in experimentally showing that nanotube chirality was robustly retained along the length under dynamic growth conditions. Since some of wording is misleading, we have revised the Abstract, Discussion, and Conclusion to distinguish our claims and the concept established by Yakobson et al.

> Abstract, line 19:
> **The contrast between structural memory and adaptive growth kinetics** further provides a mechanistic design principle of multiple-stage strategies, toward overcoming the conventional quality-quantity trade-off.

Other minor rephrasing is highlighted in the revised manuscript.

Response to Reviewer #3:

Comment. *The responses to my comments and the corrections made to the manuscript are generally satisfactory, and I thus recommend publishing the manuscript.*

Response. We are delighted to hear that the reviewer recommends publishing the manuscript.

Comments. A final suggestion related to the response to my comment 8 on "As shown in Fig. 5c, Raman mapping measurements further verify the RBM continuity along nanotubes (Supplementary Fig. 2), demonstrating chirality preservation in the FCCVD system.": a corrective action should be to moderate this conclusion by mentioning in the text the possible biases related to Raman resonance effects, as recognized by the authors in their response.

Response. We appreciate the important comment on the possible bias related to Raman resonance effects. We agree with the reviewer's comment and have moderated the statement accordingly.

> On page 18, line 335:
> We note that changes in Raman resonant conditions can be overlooked and may lead to overestimation of these values (Supplementary Note 10); …
>
> On page 19, line 357:
> Raman spectral mapping (Fig. 5c and Supplementary Fig. 3) shows RBM continuity along individual nanotubes, suggesting chirality preservation in the FCCVD system.

Response to Reviewer #4:

***Comment**. The authors have thoroughly addressed the concerns raised in the previous review. The supporting new results and analyses have improved the manuscript's rigor and clarity. In its revised form, I recommend its acceptance for publication on Nature Communications.*

Response. We are delighted to hear that the reviewer recommends the acceptance of our manuscript for publication on Nature Communications. We appreciate the insightful comments from the reviewer.

Other modifications note mentioned in the point-by-point responses.

- As we noticed that we interpreted first-round Comment 5 by Reviewer #2 differently, we have removed the paragraph starting with "In light of the observed structural robustness" (page 23, line 455, previous version) to improve readability and avoid potential reader confusion with the work by Yakobson et al. (Nat. Commun. 2014).
- Fig. 1 has been revised to emphasize that supported- and floating-catalyst systems share similar tube-catalyst interface dynamics, despite significant differences in other aspects.
- In Fig. 2, we added a conceptual illustration (panel b) to convey how isotope-labeled growth histories provide an indirect readout of catalyst-state evolution during growth; this point is also clarified in the corresponding main-text description.
- Since our MD simulations do not provide universal support for chirality preservation across a wide range of growth rate, tube chirality, catalyst elements, and temperature, we have added a sentence to indicate the limitation of our study (page 20, line 383).

Further systematic simulations across different chiralities, carbon-supply rates, and temperature are needed to define the quantitative limits of this structural robustness.

- In Supplementary Fig. 7e, estimated CNT lengths based on the analytical model have been added for extrinsic lifetime τ_2 of 10 s, assuming floating-catalyst system, showing the different impact of intrinsic lifetime on final lengths.

Response to reviewer report for manuscript No. NCOMMS-26-008054B-Z by Otsuka et al.

We sincerely thank the reviewer for taking his/her time to again review the manuscript and provide insightful comments. While the comments from the reviewer are written in ***italic blue***, our point-by-point response and revisions made are provided below in **black** and **red**, respectively. Changes made in response to the reviewer's comments and other minor modifications are highlighted in yellow and light blue, respectively, in the main text.

Response to Reviewer #2:

Comment. *Compared with the previous version, the manuscript has been improved. However, several important concerns regarding the interpretation of the results and the validity of the conclusions remain unresolved.*

Response. We sincerely thank the reviewer for taking time to evaluate our manuscript. We are glad to hear that the reviewer stated, "Compared with the previous version, the manuscript has been improved".

Comment. 1. The authors claim that this work "provides a foundation for practical strategies that integrate structural control with scalable growth." Based on the findings presented here, what specific strategies do the authors envision for achieving both controlled SWCNT structures and scalable production? More concrete discussion would strengthen the practical significance of the work.

Response. We thank the reviewer for a helpful suggestion to make the practical implication concrete. The foundation our results provide is the decoupling itself: because chirality and diameter are fixed at nucleation and preserved through elongation even as the catalyst coarsens and the temperature changes, the requirement for structural control and the requirement for scalable (long, high-yield) growth act at *different stages* of the process and can be optimized independently. This would not be the case if the diameter were re-set whenever the environment is modulated to raise throughput (higher temperature, increased carbon activity). Then, structural control and scale-up would be in direct competition; it is the structural memory that removes this competition.

In particular, this defines a multiple-stage strategy. (i) Structure is set only at nucleation: reactor geometry, precursor supply, and the inlet temperature profile are chosen so that tubes nucleate with the intended diameter/chirality, and confining nucleation to a narrow inlet window (please see our response to Comment 7) narrows the resulting distribution. (ii) Length is then maximized over the elongation stage within a finite reactor. Because the catalyst chemical potential μ_{cat}, which is set by catalyst size and gas-phase carbon activity, raises the growth rate γ but shortens the intrinsic growth lifetime (Fig. 3c, Supplementary Note 7), and because the final length is γ integrated over that lifetime but capped by the extrinsic residence time, the optimal carbon-

activity profile depends on the reactor. When the residence time is much shorter than the intrinsic lifetime, as in some practical reactors, length is residence-time-limited and a higher carbon activity that raises γ is favorable; a mild etchant downstream that suppresses catalyst poisoning further extends the usable lifetime (Supplementary Note 7). In the opposite, long-reactor limit where the intrinsic poisoning-limited lifetime dominates (including supported-catalyst growth), a lower carbon activity that grows tubes slowly but keeps the catalyst alive is preferable. Spatial (temporal) control of carbon activity along a reactor is an established route to uniform, large-yield growth [Sato *et al.*, *Carbon* **136**, 143 (2018), Yasuda *et al.*, *Nano Lett.* **11**, 3617, 2011], supporting the implementation of these multiple stages as distinct zones of a single reactor.

Such a modulation of carbon activity may be achieved by deep injection of additional carbon sources and etching agents, as well as by designing furnace temperature profile. This is only a design principle derived from our results; its full experimental realization is beyond the present scope. We have sharpened the corresponding Discussion paragraph to state the two-stage logic explicitly.

Revision: On page 23, line 437
These considerations suggest that intentional perturbations of the growth environment at the reactor inlet, including precursor concentration and temperature gradients, could be used to confine the nucleation zone, thereby limiting the range of catalyst sizes sampled at nucleation and potentially narrowing the diameter distribution. Complementarily, the robust structural memory allows for independent optimization of gas-phase carbon activity[21] at the subsequent elongation zone to maximize CNT length without disturbing the established diameter. We have seen that the catalyst chemical potential μ, which depends on….

Comment. 2. In Figure 1, the evolution of the carbon chemical potential along the reactor coordinate is illustrated. It is generally accepted that the chemical potential of carbon decreases with increasing temperature. Why, then, does the schematic indicate the highest carbon chemical potential at the reaction zone, where the temperature is also the highest? The physical basis for this representation should be clarified.

Response. We appreciate the very insightful comment. Our original intention was to indicate that carbon sources and etching agents undergo thermal decomposition and depletion due to reactions with catalyst nanoparticles or a reactor wall. The influence of thermal decomposition is, for example, evident from different growth rates of CNTs placed upstream or downstream of the furnace [e.g., Otsuka *et al.*, *ACS Nano* **16**, 5627 (2022)-Fig. S2], as ethylene and acetylene are generated from ethanol at high temperatures [Sugime *et al.*, *Carbon* **50**, 2953 (2012)]. At the same time, highly reactive chemicals (e.g., acetylene) progressively consumed downstream and deplete at the downstream [Sato *et al.*, *Carbon* **136**, 143 (2018)], which may lead to a peak in the carbon activity rather than a monotonic profile.

Nevertheless, we agree that chemical potential generally decreases with increasing temperature. After carefully examining the use of gas-phase chemical potential as a representative of such feature, we have modified the term to gas-phase carbon activity a_C, defined relative to graphite

(a_C=1 at equilibrium with graphite), $a_C = \exp[(\mu_{gas} - \mu_{gr})/kT]$. Because a_C is referenced to graphite *at each temperature*, the temperature dependence of the reference potential, which the reviewer correctly notes, is divided out, so the profile in Fig. 1 reflects the local gas composition rather than the absolute $\mu(T)$. It is the net thermodynamic driving force for carbon transfer from the gas to the catalyst, which is raised by carbon sources (e.g., acetylene) and lowered by etching agents (e.g., water). Note that a_C corresponds to the carbon chemical potential μ_{cat} that the catalyst would attain when equilibrated with the gas in the absence of the growing tube; during growth, continuous carbon drain into the nanotube holds the catalyst below gas-phase a_C. We have modified Fig. 1b and the corresponding text.

Revision: On page 4, line 53 (only representative one shown below)
Thermal decomposition and depletion of feedstock species in CVD reactors generate spatial variations in gas-phase composition and carbon activity a_C[21], leading to position-dependent growth behavior[22,23].

Comment. 3. The authors propose that the previously reported volcano-type bandgap dependence may only emerge under specific growth conditions. What are these conditions? Do they relate primarily to the catalyst composition, growth temperature, carbon precursor, or other parameters? A more explicit discussion is needed.

Response. We thank the reviewer for this comment. To clarify our position, our data are limited to our own growth conditions, and within these conditions we did not observe any metallicity-dependent difference. Determining the specific conditions (catalyst composition, temperature, precursor, or others) under which a bandgap dependence would appear would require a dedicated comparative study across these parameters, which is far beyond the scope of the present work. After carefully reflecting on this point, we have decided not to discuss a mechanism that our own data cannot verify because pursuing this comparison in depth would risk obscuring the central message of the manuscript. Accordingly, we have removed the previous expression "volcano-type bandgap dependence" from the main text and now describe only what we observed: no metallicity-dependent difference in growth behavior under the present conditions. We believe that the comment has helped us improve the clarity of the manuscript.

Revision: On page 8, line 130
We briefly compared the growth-rate distributions of each type within individual temperature segments. Across the examined temperature range, no significant metallicity dependence was observed (Fig. 2g).

Nevertheless, we speculate that the difference may come down to a few factors such as growth temperature, catalyst charging, or nanotube diameter. In particular, Fe-catalyzed CVD growth has been shown to involve charge generation and transfer, with the growing nanotube becoming negatively charged [Wang *et al*., *Nano Lett.* **16**, 4102 (2016)]. In a substrate-supported geometry this charge can dissipate through the substrate, whereas in a kite-growth geometry it may need to

be removed through the nanotube itself, in which case the kinetics could depend on the electronic type. Confirming which factors dominate would require a dedicated study.

Comment. 4. Regarding the temperature dependence of the survival probability, the data points presented in Figure 3c appear highly scattered, making it difficult to establish a clear trend. Furthermore, the large error bars raise concerns about the statistical significance and reliability of the reported correlation. The authors should provide additional evidence to support this conclusion.

Response. We thank the reviewer for raising the statistical reliability of the survival probability. We fully agree that the confidence interval (CI) of any *individual* 30-s bin is too wide to establish a trend on its own. **Our claim, however, does not rest on individual bins but on the *systematic* offset between the ramp-up and ramp-down branches**. To test this within the same Poisson framework, we pooled the growing-CNT statistics over a common temperature band (818.9–850.1°C), separately for the ramp-up and ramp-down stages, and recomputed the mean survival probability p with an exact 95% CI on the pooled termination count.

The pooled survival probability is p_{up} = 99.87% (95% CI 99.76–99.94%) during ramp-up and p_{down} = 99.19% (95% CI 98.76–99.50%) during ramp-down, and the two intervals do not overlap. Equivalently, the pooled termination probability is 6.2× higher on the ramp-down branch than on the ramp-up branch at the same temperature range. This is not an artifact of the chosen band: restricting the analysis to the 823.9–847.9°C interval gives p_{up} = 99.91% (99.79–99.97%) and p_{down} = 99.07% (98.56–99.44%), a 10.5× difference. Because the two branches include the comparable set of temperatures visited at different times, it evidences an irreversible change accumulated during high-temperature exposure that shortens the growth lifetime, consistent with the catalyst coarsening independently supported by our AFM and KMC results (Fig. 4). We have added this pooled analysis in Supplementary Note 6.

Revision: On page 18in Supplementary Information
Because the confidence interval of any single 30-s bin is too wide to establish a trend on its own, we instead compared the systematic difference in the mean survival probability p between the ramp-up and ramp-down stages by pooling the termination statistics over a common temperature band. For each stage, we summed the termination events D and the numbers of growing nanotubes N over the bins, evaluated a pooled rate $q_{pool} = \Sigma D/(\Delta t \cdot \Sigma N)$, and obtained the mean survival probability $p = \exp(-q_{pool} \cdot \tau)$ with an exact 95% confidence interval (CI) on the Poisson count ΣD. The two branches give clearly non-overlapping intervals, p_{up} = 99.87% (95% CI 99.76–99.94%) and p_{down} = 99.19% (95% CI 98.76–99.50%) over 818.9–850.1°C, and the separation is preserved when the band is narrowed (Supplementary Table 3). This significant difference in growth lifetime indicates that an irreversible change occurred between the two stages; together with the concurrent hysteretic increase in growth rate, it is most reasonably attributed to catalyst coarsening.

Supplementary Table 3 | Pooled growth-survival statistics for the ramp-up and ramp-down stages.

Temperature band	branch	ΣD	ΣN	Mean p	95% CI
818.9–850.1°C	Ramp-up	10	254	99.87%	99.76–99.94%
	Ramp-down	21	86	99.19%	98.76–99.50%
823.9–847.9°C	Ramp-up	5	188	99.91%	99.79–99.97%
	Ramp-down	19	68	99.07%	98.56–99.44%

Comment. 5. A central conclusion of the manuscript is that different CNT growth rates observed at the same growth temperature arise from catalyst coalescence, which increases the catalyst surface area. Several concerns should be addressed: (a) Among the CNTs investigated, what fraction exhibits a growth-rate increase attributable to catalyst coalescence?

Response. We thank the reviewer for the insightful comment. First, we clarify again that in our supported-catalyst experiments, **the catalyst coarsening behind the growth-rate variation is attributed primarily to Ostwald ripening rather than to particle coalescence**. Taking question (a) as the fraction of CNTs whose growth-rate increase is attributable to catalyst coarsening, the most direct evidence comes from comparing the growth rate of the same nanotube at nearly identical temperatures but at different stages of catalyst evolution. Table R2 in our previous response letter (reproduced below) lists the SWCNTs that continued to grow across the maximum temperature: **all among eight investigated CNTs grew faster during ramp-down than during ramp-up** at ~849°C and ~838°C. These tubes provide the clearest comparison, but they represent a biased subset, as their relatively long lifetimes are precisely what allow the same-temperature comparison.

To estimate the fraction across the broader population, we use the apparent activation energy E_a (Fig. 3e). Since intrinsic elongation is characterized by E_a of 1–2 eV—both in the ramp-down stage here and in previous studies—we take $E_a > 2$ eV during ramp-up as a signature of coarsening-assisted acceleration, which is met by 84% of the ramp-up population. We note that this value somewhat overestimates the true fraction, as ~25% of the ramp-down population also exceeds 2 eV owing to statistical fluctuation and measurement uncertainty. We have added the following sentences in Supplementary Information.

Revision: On page 17 in Supplementary Information (Note 6)
First, all the CNTs displayed in the bottom right panel grew faster during T ramp-down than during ramp-up when compared at comparable temperatures, indicating the coarsening of the corresponding catalyst. Since the intrinsic E_a is estimated to be ~1.6 eV (Fig. 4e), more than 80% of CNTs in the upper three panels suggest the coarsening of catalysts (apparent $E_a > 2$ eV).

Table R2 (reproduced from the previous response letter) | **Growth rate hysteresis within individual SWCNTs.** Instantaneous growth rates of SWCNTs, which grew across the maximum temperature and show hysteresis (Supplementary Fig. 6a, bottom right), and the acceleration factors of each nanotube at nearly identical temperatures during T ramp-up and ramp-down periods.

**Center *T* (°C)**	**837.1 (up)**	**850.1 (up)**	**847.9 (down)**	**838.5 (down)**	**Ratio (~849°C)**	**Ratio (~838°C)**
**Center *t* (s)**	**375**	**435**	**645**	**675**		
**#r1**	4.7	9.6	11.7	8.3	1.21	1.76
**#r2**	4.2	6.4	11.7	12.2	1.83	2.89
**#r3**	7.5	11.6	12.9	11.9	1.11	1.58
**#r4**	17.5	28.4	37.6		1.32	
**#r5**		18.4	38.0		2.06	
**#r6**		10.6	17.1	20.5	1.62	
**#r7**	11.2	15.6	28.6	21.1	1.83	1.88
**#r8**		18.2	27.2	20.8	1.50	

Comment. 5(b) Ethanol was used as the carbon precursor in this study. Under such conditions, a weak-etching growth regime, where elongation rate scales with catalyst surface area, is generally not expected. Moreover, the group's previous studies using ethanol consistent with models in which the CNT growth rate is primarily governed by chiral angle. How can these observations be reconciled with the mechanism proposed here?

Response. We thank the reviewer for this insightful comment, which prompts us to clarify how the elongation rate depends on tube chirality versus catalyst surface area within our kinetic framework.

In our model the growth rate is expressed as

$$\gamma = \frac{Ak_{\mathrm{g}}(k_{\mathrm{ad}}P_{\mathrm{C}} - k_{\mathrm{de}}P_{\mathrm{E}}N_{\mathrm{eq}})}{k_{\mathrm{g}}D + Ak_{\mathrm{de}}P_{\mathrm{E}}}$$

where the term $k_{\mathrm{g}}D$ carries the chirality dependence (through the diameter D and the chirality-dependent precipitation constant k_{g}) and $Ak_{\mathrm{de}}P_{\mathrm{E}}$ represents the ethanol-derived etching. The logarithmic sensitivities of γ to these two quantities are

$$S_{\mathrm{g}} \equiv \frac{\partial \ln \gamma}{\partial \ln k_{\mathrm{g}}} = \frac{Ak_{\mathrm{de}}P_{\mathrm{E}}}{k_{\mathrm{g}}D + Ak_{\mathrm{de}}\mathrm{P}_{\mathrm{E}}}$$

$$S_{\mathrm{A}} \equiv \frac{\partial \ln \gamma}{\partial \ln A} = \frac{k_{\mathrm{g}}D}{k_{\mathrm{g}}D + Ak_{\mathrm{de}}\mathrm{P}_{\mathrm{E}}}$$

Introducing the dimensionless ratio $y \equiv Ak_{\mathrm{de}}P_{\mathrm{E}}/k_{\mathrm{g}}D$, these reduce to $S_{\mathrm{g}} = y/(1+y)$ and $S_{\mathrm{A}} = 1/(1+y)$, so that $S_{\mathrm{g}}+S_{\mathrm{A}} = 1$. The sensitivity to catalyst surface area and the sensitivity to chirality are thus strictly complementary, and their balance is set by a single parameter y — that is, by the magnitude

of the etching term relative to the precipitation term, not by the type of the carbon precursor or etching agent.

The reviewer is correct that ethanol supplies an etching agent ($P_E \neq 0$). However, the critical factor is its magnitude. Using parameters experimentally calibrated for low-pressure ethanol CVD in our previous study [*ACS Nano* **16**, 5627 (2022)] we obtain $S_A \approx 0.89$ and $S_g \approx 0.11$. The elongation rate is therefore nearly proportional to the catalyst surface area and only weakly sensitive to chirality. This is fully consistent with the same study, in which low-pressure ethanol CVD yields a chirality-insensitive growth rate (*ACS Nano* 2022, Figs. 1a and 4a).

The chirality-governed behavior to which the reviewer refers emerges only when the ethanol partial pressure is raised by roughly 10–20× (atmospheric-pressure CVD). In that regime, S_A decreases to ~0.46–0.31 and the growth rate acquires the pronounced chiral-angle dependence reported in *ACS Nano* 2022 (Figs. 1b and 4h). The two observations are therefore not contradictory: they correspond to opposite ends of the same $S_g + S_A = 1$ relationship, selected by *the absolute etching-agent pressure*. We emphasize that this strong area-sensitivity at low pressure (large S_A) is precisely what makes the elongation rate a sensitive probe of catalyst coarsening in the present study; at high pressure, the rate would instead become robust against such size changes (small S_A), a tendency also apparent in Supplementary Fig. 7. To improve the clarity of this point, we have revised the main text and Supplementary Information as follows:

> **Revision:** On page 12, line 218
> This behavior can be rationalized if the elongation rate scales with catalyst surface area, so that catalyst size evolution (Fig. 4b) produces different rates at the same temperature. Such scaling nearly holds in the weak-etching regime at the low ethanol pressure (~135 Pa) used here[40,45] (see Supplementary Fig. 7).

> **Revision:** On page 25 in Supplementary Information (Note 7)
> Note that when the sensitivity to k_g is defined ($S_g \equiv \partial \ln\gamma / \partial \ln k_g$), $S_A + S_g = 1$, indicating the complementary features that depend only on dimensionless ratio $Ak_{de}P_E/k_gD$.

Comment. 5(c) Following plasma treatment, the catalyst particles exhibit a bimodal size distribution. However, no direct evidence is provided regarding whether smaller or larger catalyst particles preferentially lead to faster CNT elongation. Such evidence would be important to support the proposed mechanism.

Response. We thank the reviewer for this question. We would like to clarify both the role of this measurement and how the size dependence of the elongation rate is established. First, the bimodal distribution comes from a separate, ensemble-level experiment whose sole purpose is to show that coarsening becomes pronounced at elevated temperature; the particles were analyzed "irrespective of whether they nucleated CNTs," with the CNTs intentionally removed by oxygen plasma to resolve the catalyst-size distribution by AFM (Fig. 4c). In this dense and unaligned sample, an individual CNT cannot be assigned to its parent catalyst by construction, and such analysis is not the purpose of this measurement.

Instead, the size dependence of the rate itself is directly established by He et al. [*Sci. Adv.* **5**, eaav9668 (2019)] through in situ ETEM: the CNT growth rate scales linearly with the active catalyst surface-area-to-tube-diameter ratio A/D_T (their Fig. 2D). Because the tube diameter is fixed at nucleation in our system, this reduces to a proportionality between elongation rate and active catalyst surface area, so a coarsening particle elongates its tube faster, which is consistent with the scaling of Eq. 3 ($\gamma \propto R^2$). Despite the difference of growth conditions, our response to Comment 5(b) quantifies that this low-pressure ethanol condition lies in the surface-area-dominated (weak-etching) regime ($S_A \sim 0.89$), validating the adoption of their observation in our experiments. We have added the values in the main text.

Revision: On page 13, line 231

In contrast, growth at 850°C resulted in the emergence of larger particles (~10–15 nm) via Ostwald ripening, yielding a bimodal size distribution[46], while main population remained near 4–5 nm.

Comment. 6. Additional information regarding the catalyst nanoparticles is required. What are the average particle diameters before and after coalescence? Are the catalyst particles in the solid or liquid state under the CVD growth conditions? Could the change in particle size alter the catalyst phase state and thereby influence the CNT elongation rate?

Response. We appreciate these insightful questions. First, we note that, in our supported-catalyst system, the catalyst enlarges through Ostwald ripening rather than through discrete particle coalescence. There is therefore no well-defined pair of "before/after" states to compare; the size distribution evolves gradually and monotonically during growth. What we can report is this continuous evolution, characterized by AFM after growth at two temperatures: at 800°C, the catalyst size distribution peaks near 5 nm, whereas at 850°C it shows a peak near 4 nm together with a minor population of large particles at ~10–15 nm (see Figure R1 below).

Second, as a reference point, Rao et al. [*ACS Nano* **7**, 1100 (2013)] located the Fe solid-to-liquid transition at 1000–1100°C for ~2.5-nm particles, a value that already includes a Gibbs-Thomson melting-point depression of ΔT~125°C. Our growth temperatures (800–873°C) lie below this range, and since the Gibbs-Thomson depression scales inversely with particle radius, our larger catalysts would experience an even smaller effect. For this reason, active catalyst nanoparticles are likely to be solid under our conditions, although we refrain from an explicit categorical assignment in the main text, as the size of the individual *active* catalysts was not directly measured. We would like to directly address this important point in future work, when appropriate methods become available.

Third, even if our conditions happened to lie near such a phase boundary, our conclusion would be robust: Rao *et al*. report no pronounced discontinuity in the initial growth rate across the Fe solid-to-liquid transition, so a phase change would not by itself account for the observed rate variation. Together with our experimental observations, where no abrupt jump in Arrhenius plots is observed (Supplementary Fig. 6a), the growth rate change is therefore most naturally attributed to temperature and the gradual increase in catalyst surface area ($\gamma \propto R^2$, Eq. 3).

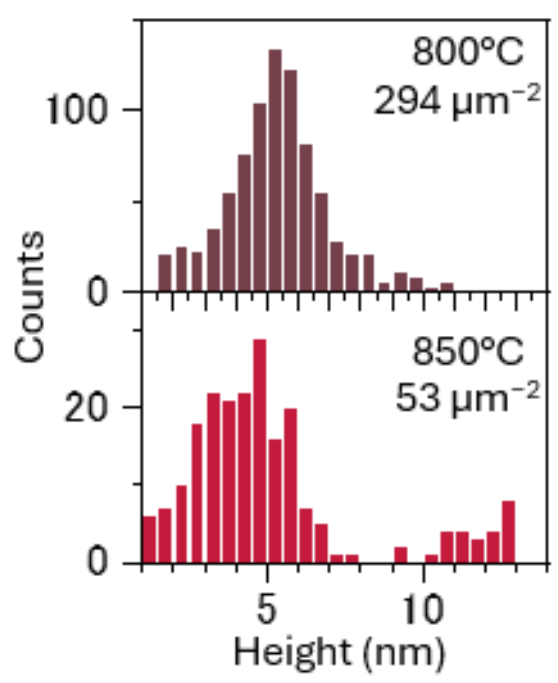

Figure R1 | Catalyst height histograms extracted from Fig. 4c for the catalyst nanoparticles after CNT growth at 800°C (top) and 850°C (bottom) for 20 min.

Comment. 7. The authors state that "intentional perturbations of the growth environment at the reactor inlet, including precursor concentration and temperature gradients, could be used to confine the nucleation zone, potentially narrowing the diameter distribution." Given the authors' conclusion regarding robust structural memory during growth, it is unclear how such an approach would effectively narrow the diameter distribution. Additional explanation is needed.

Response. We thank the reviewer for this comment. Here, we would like to provide a more detailed explanation below. Based on the present finding, we suppose that the width of the diameter distribution originates from the variation of the catalyst size. Specifically, different tubes reach the nucleation threshold at different times and positions along the reactor, and a tube that nucleates later (downstream) grows from a coarsened catalyst and therefore has a larger diameter. Because the diameter is determined at nucleation and preserved afterward, the final distribution corresponds to the catalyst-size distribution sampled over these various nucleation events.

For example, a sharp increase in the carbon activity over a short section of the reactor promotes the nucleation of many tubes simultaneously at that position, whereas a decrease in the carbon activity immediately downstream suppresses further nucleation from the catalysts that have coarsened by then. This suppression removes the large-diameter tail that late nucleation would otherwise produce. In the limit where the activity profile confines the nucleation to a single position, the diameter distribution of the CNTs coincides with the catalyst-size distribution at this single position. In this limit, the contribution from gradual coarsening is eliminated, and only the instantaneous polydispersity at nucleation timing remains. In this way, the perturbations at the inlet could narrow the diameter distribution. <u>We have modified the main text to clarify the above scenario, which is included in the revision related to Comment 1</u>.

Comment. 8. Among the CNTs investigated, only 88% retained their chiral indices over the measured growth length. What mechanisms are responsible for the chirality changes observed in the remaining 12% of CNTs? Do these observations challenge the conclusion of robust chirality preservation? The authors should discuss this apparent inconsistency.

Response. We thank the reviewer for this question. By "robust", we mean that the per-length frequency of chirality-change events is not pushed beyond the range already spanned by our previous studies by dynamic environmental modulation, not that such changes never occur. Our value, 2.6 mm^{-1} (24 events over ~9 mm), lies within that range: ~1.3 mm^{-1} at steady 800°C [39] and ~2.9 mm^{-1} under an interrupted-etching protocol [41] (Fig. 5b). Given the additional uncertainty in comparing these datasets, where CNTs are harder to trace back to corresponding catalyst at the higher densities of the previous studies, dynamic modulation and the accompanying catalyst coarsening therefore do not measurably increase the transition frequency. The 88%/12% split is, by contrast, a length-dependent fraction: at a fixed event rate, shorter tubes contain fewer transitions, so the frequency per unit length, not the fraction of tubes containing such an event, is the physically meaningful quantity.

Mechanistically, these residual transitions arise from the same stochastic process that operates even under steady growth: transient non-hexagonal rings form at the dynamic tube–catalyst edge

and, in the great majority of cases, are healed before the sidewall buries them [Yuan et al., *Phys. Rev. Lett.* **108**, 245505 (2012)]. A chirality change is the residual outcome when healing loses this kinetic race to hexagon incorporation, so its per-length frequency is set by the carbon-incorporation rate rather than by qualitatively new pathway. Two factors could in principle raise this (*n*,*m*) transition rate in the present system.

First, a platform-specific one: strong tube-substrate interaction increases curvature and locally lowers the barrier for 5|7-pair formation (Supplementary Fig. 16). Free-standing CNTs in FCCVD lack this effect. Second, catalyst coarsening, which raises the growth rate at fixed temperature and thereby shifts the healing-versus-incorporation balance; the fixed defect fraction increases with carbon-supply rate (Supplementary Fig. 17) [27]. Coarsening therefore acts only indirectly, through this elevated rate, and is not a direct cause of (*n*,*m*) change. Indeed, MD simulations with oversized or abruptly coalesced catalysts preserves chirality on an active Fe surface (Supplementary Note 16). Consistent with a rate-mediated rather than coarsening-specific origin, within the sampled range we find no systematic correlation between growth-rate acceleration and chirality transitions—tube #3 preserves its chirality through a ~3.5-fold rate increase over a ~30°C rise (Fig. 5a)—and this kinetic channel should become significant only at carbon activities well above those used here. To clarify this point, we have added a sentence in the main text as follows.

Revision: On page 18, line 335

However, the overall level of chirality preservation is comparable—though classification may be modestly influenced by CNT array density—indicating that dynamically evolving environments do not measurably increase the incidence of chirality transitions. The residual transitions are consistent with the stochastic, kinetically governed defect formation that persists even under steady growth.

Comment. 9. As supporting evidence, molecular dynamics simulations were performed only for a (8, 7) SWCNT, which is known to possess relatively high structural stability. To more comprehensively evaluate chirality robustness during catalyst coalescence, simulations on less stable nanotubes, such as (n,1) or (n, 0) species, are recommended.

Response. We thank the reviewer for the very insightful suggestion. We agree that according to the previous study by Xu et al. [*Carbon* **175**, 69 (2021)], (n,1) or (n,0) species may be less stable and worth investigating for their chirality robustness. We therefore performed the same MD simulation for a (13,0) CNT, which has the exact same diameter but much different chiral angle from a (8,7) CNT, and found that the tube chirality is robustly preserved over ~5.2 nm within ~300 ns, despite the intended catalyst coarsening. In addition, the case with a (10,5) CNT also showed chirality preservation during the same catalyst coarsening. We therefore believe that the observed structural stability is not specific to near-armchair species but applies to a wide range of chiral species. <u>We have added MD simulation results in Supplementary Fig. 18 and Supplementary Movie 3.</u>

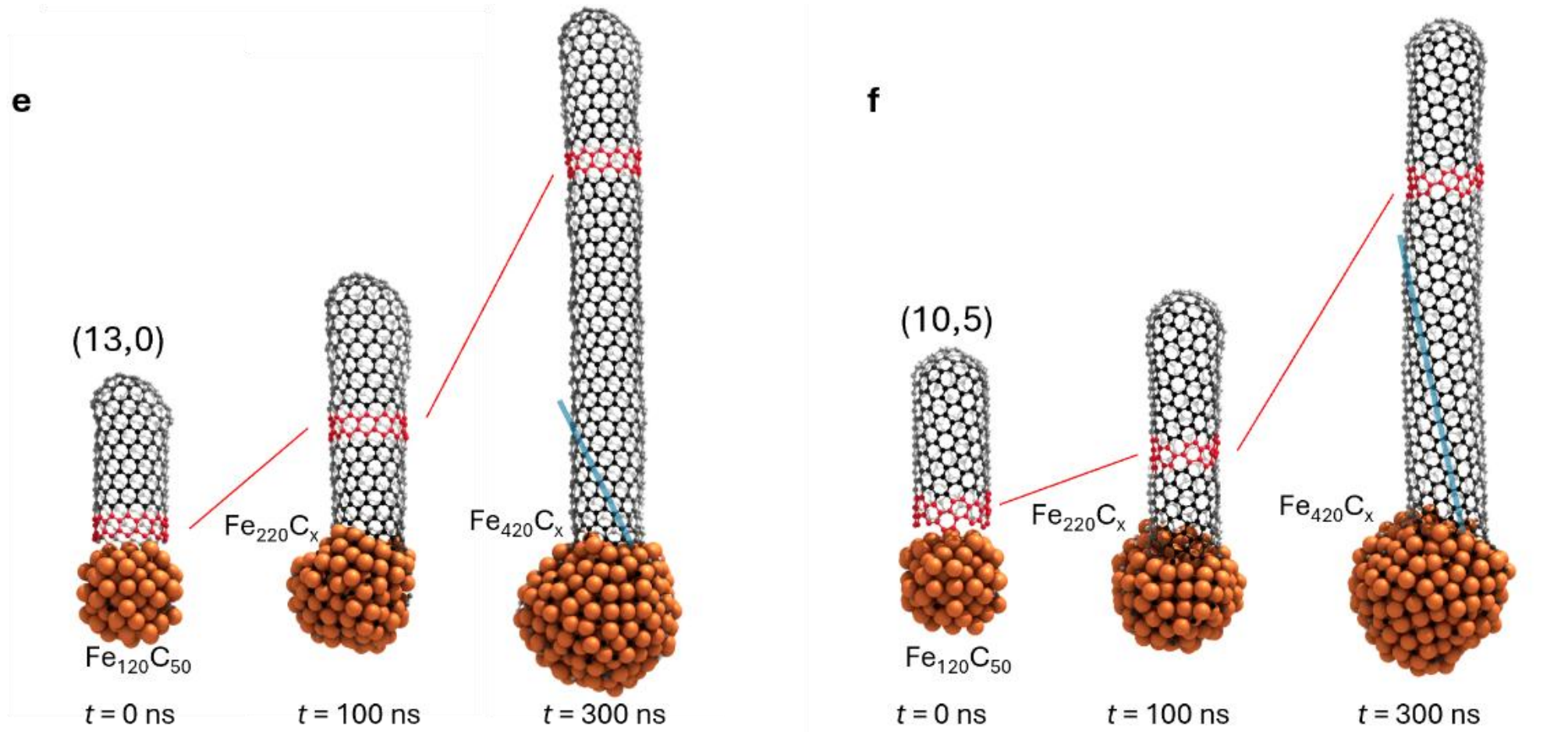

Supplementary Fig. 18 | e,f Snapshots of a (13,0) CNT (e) and a (10,5) CNT (f) growing from an iron nanoparticle that grows in size at a rate of 1 atom/ns. The same parameters for carbon supply and removal are used as an (8,7) CNT case in Fig. 5e.

Revision: On page 20, line 378
Consistent results are obtained for the cases of a (13,0) CNT and a (10,5) CNT under otherwise identical conditions (Supplementary Fig. 18).

Revision: On page 54 in Supplementary Information (Note 16)
To assess whether the observed structural robustness depends on the specific chirality, we performed additional growth simulations for two different chiral indices under otherwise identical conditions: a zigzag (13,0) tube and a chiral (10,5) tube (Supplementary Fig. 18e,f). In particular, zigzag CNTs have been reported to be highly sensitive to the size of catalyst particles[44]. In both chirality cases, the tubes retained their initial structures throughout the simulated growth while the catalyst enlarged from Fe_{120} to Fe_{420} (t = 0–300 ns), which are consistent with the behavior of the (8,7) tube. The absence of chirality changes across near-

armchair, chiral, and zigzag tubes indicates that the structural memory under catalyst coarsening is not restricted to particular chiral angles.

Other revisions:

- In Supplementary Information (on page 25), "Note that when the sensitivity to k_g is defined …" has been added.
- On page 20, line 383, "Further systematic simulation across different diameters, carbon-supply rates, and temperature are needed…" because we have added MD simulations for other chiral angles.
- The numbers of analyzed CNTs in Supplementary Fig. 4b have been corrected.
- Other minor typos, grammatical errors, inaccurate phrases are corrected and highlighted in the main text.
- Duplicate of the reference (Liao et al., [18] and [59] in the previous manuscript) has been resolved.
